# Exercise therapy and self-management support for individuals with multimorbidity: a randomized and controlled trial

**DOI:** 10.1038/s41591-025-03779-4

**Published:** 2025-06-30

**Authors:** Søren T. Skou, Mette Nyberg, Mette Dideriksen, Hanne Rasmussen, Jan Arnholtz Overgaard, Christine Bodilsen, Anne Merete B. Soja, Amir Pasha Attarzadeh, Manuel J. Bieder, Nadia P. Dridi, Andreas Heltberg, Peter H. Gæde, Johan L. Reventlow, Sidse Arnfred, Uffe Bodtger, Jan C. Brønd, Lau C. Thygesen, Sanne P. Møller, Madalina Jäger, Alessio Bricca

**Affiliations:** 1 The Research and Implementation Unit PROgrez, Department of Physiotherapy and Occupational Therapy, Næstved-Slagelse-Ringsted Hospitals, Slagelse, Denmark; 2 Center for Muscle and Joint Health, Department of Sports Science and Clinical Biomechanics, University of Southern Denmark, Odense, Denmark; 3 Department of Rehabilitation, Lolland Municipality, Maribo, Denmark; 4 Department of Exercise and Health, Roskilde Municipality, Roskilde, Denmark; 5 Department of Internal Medicine 1, Section of Cardiology, Holbæk Hospital, Holbæk, Denmark; 6 Centre for Evidence-Based Orthopaedics, Department of Orthopedic Surgery, Zealand University Hospital, Køge, Denmark; 7 Department of Orthopaedic Surgery, Næstved Hospital, Næstved, Denmark; 8 Department of Cardiology, Zealand University Hospital, Roskilde, Denmark; 9 Department of General Practice, Institute of Public Health, University of Copenhagen, Copenhagen, Denmark; 10 Department of Cardiology and Endocrinology, Slagelse Hospital, Slagelse, Denmark; 11 Institute for Regional Health Research, University of Southern Denmark, Odense M, Denmark; 12 General Practitioners Reventlow and Wolfhagen, Slagelse, Denmark; 13 Psychiatric Research Unit, Copenhagen University Hospital – Psychiatry Region Zealand, Slagelse, Denmark; 14 Department of Clinical Medicine, University of Copenhagen, Copenhagen, Denmark; 15 Pulmonary Research Unit Region Zealand (PLUZ), Department of Respiratory Medicine, Zealand University Hospital Næstved, Næstved, Denmark; 16 The Research Unit for Exercise Epidemiology, Centre of Research in Childhood Health, Department of Sports Science and Clinical Biomechanics, University of Southern Denmark, Odense, Denmark; 17 National Institute of Public Health, University of Southern Denmark, Copenhagen, Denmark; 18 Danish Centre for Motivation and Behaviour Science, University of Southern Denmark, Odense, Denmark

**Keywords:** Rehabilitation

## Abstract

Despite increasing individual and societal burden, evidence for effective management strategies of multimorbidity is missing. Exercise therapy and self-management support are promising interventions, but their effect has not been evaluated. We hypothesized that exercise therapy and self-management support were superior to usual care alone in improving health-related quality of life (HRQoL) in individuals with multimorbidity. In this pragmatic multicenter, assessor-blinded randomized controlled trial (MOBILIZE), we enrolled 228 adult patients with two or more selected long-term conditions that limited their daily activities, but who were able to walk at least 3 meters without assistance, and who did not have unstable health conditions, life expectancy less than 12 months, or selected psychiatric conditions. Patients were randomized (1:1) to a 12 week personalized exercise therapy and self-management support program in addition to usual care or usual care alone. The primary outcome was HRQoL (using the EQ-5D-5L (European Quality of Life 5-dimensions 5-level version), ranging from −0.758 to 1, with higher scores being better) at 12 months, while secondary outcomes included functional performance (6 minute walk test and the 30 second chair-stand test), serious adverse events (SAEs), physical activity level (steps per day and minutes per day of at least light intensity measured with accelerometers), disease burden (Bayliss burden of illness measure), depression (Personal Health Questionnaire Depression Scale-8), anxiety (General Anxiety Disorder-7), self-efficacy (Self-Efficacy for Managing Chronic Disease scale), disability (12 item WHO Disability Assessment Schedule) and self-rated health (EQ-VAS (EuroQoL Visual Analog Scale)). In total, 197 of 228 participants (86%) completed the 12 month follow-up. On intention-to-treat analysis the exercise therapy and self-management support program had a statistically significantly greater effect on HRQoL than usual care alone (0.050 versus −0.014; adjusted mean difference, 0.064 points; 95% CI: 0.014–0.115). There were 36 and 48 SAEs in the exercise therapy and self-management group and usual care group, respectively (*P* = 0.388). Among the other secondary outcomes, only self-rated health was statistically significantly different between the groups (adjusted mean difference, 6.9 points; 95% CI: 1.8–12.1), in favor of the intervention group. In conclusion, this trial suggests that personalized exercise therapy and self-management support are more effective than usual care alone in improving health-related quality of life at 12 months in adults with multimorbidity, without compromising safety. The clinical relevance of the results remains unclear. ClinicalTrials.gov registration: NCT04645732.

## Abstract (web summary)

The authors report findings from the MOBILIZE trial, which found that exercise therapy and self-management support improved patient-reported quality of life in individuals with multiple chronic conditions.

## Main

Multimorbidity is commonly defined as the presence of two or more coexisting long-term conditions in the same individual^1^. It affects more than one-third of the adult population worldwide^2^, with a projected increase of 84% by 2049 (ref. ^3^). It occurs 10–15 years earlier in deprived areas^4^ and the inequality is expected to widen further in the future, especially in the working-age population^3^.

Multimorbidity is associated with reduced quality of life, physical and cognitive function, and premature death^1,5^. There is an almost exponential relationship between the number of long-term conditions and associated costs because of greater healthcare utilization, sick leave and so on^6^. As such, individuals living with multimorbidity account for three out of four of all primary care consultations^7^ and are at increasingly greater risk of hospitalization and longer hospital stays with more long-term conditions^8^.

Multimorbidity is a complex problem for research and clinical practice owing to the large heterogeneity in the measurement, presentation and severity of included long-term conditions^1,9^. Despite the massive individual and societal burden and the projected steep increase in prevalence in the future, there remains a lack of evidence on effective management strategies^10^. This is reflected in clinical guidelines and in the silo-based healthcare system specialized in the individual conditions that focus on treating each condition in isolation, rather than adopting person-centered, multimorbidity care models^1^. However, evidence suggests that a single-disease approach leads to inadequate, fragmented and even contradictory care that is inefficient and unsatisfactory to the patient and healthcare provider, and which increases the treatment burden^1,11–14^.

Recommendations on future interventional research suggest focusing on patient health behaviors such as exercise therapy^10^, which is also among the key research priorities identified by patients, carers and health professionals^15^. Exercise therapy has been identified as an effective and safe intervention for at least 25 long-term conditions^16^, including knee and hip osteoarthritis, chronic obstructive pulmonary disease, heart failure and coronary heart disease, hypertension, type 2 diabetes mellitus and depression, which are among the leading causes of global disability and which frequently co-occur^17^. In parallel, self-management support has gained recognition as a crucial element in improving health-related quality of life and reducing healthcare utilization in individuals with long-term conditions^18^. This is because self-management support is essential to ensure long-term adherence to exercise and other health behaviors, slow down the progression of long-term conditions, and improve health^18^.

Overall, while exercise therapy and self-management support appear to be promising treatment options for individuals with multimorbidity, the current evidence is of low quality, underscoring the need for high-quality randomized controlled trials (RCTs) to evaluate their effectiveness across different combinations of long-term conditions^10,19,20^.

In this pragmatic multicenter RCT (MOBILIZE), we investigated whether a 12 week personalized exercise therapy and self-management support program in addition to usual care was superior to usual care alone in improving health-related quality of life at 12 months in individuals with multimorbidity.

## Results

### Patient disposition

**Fig. 1:**
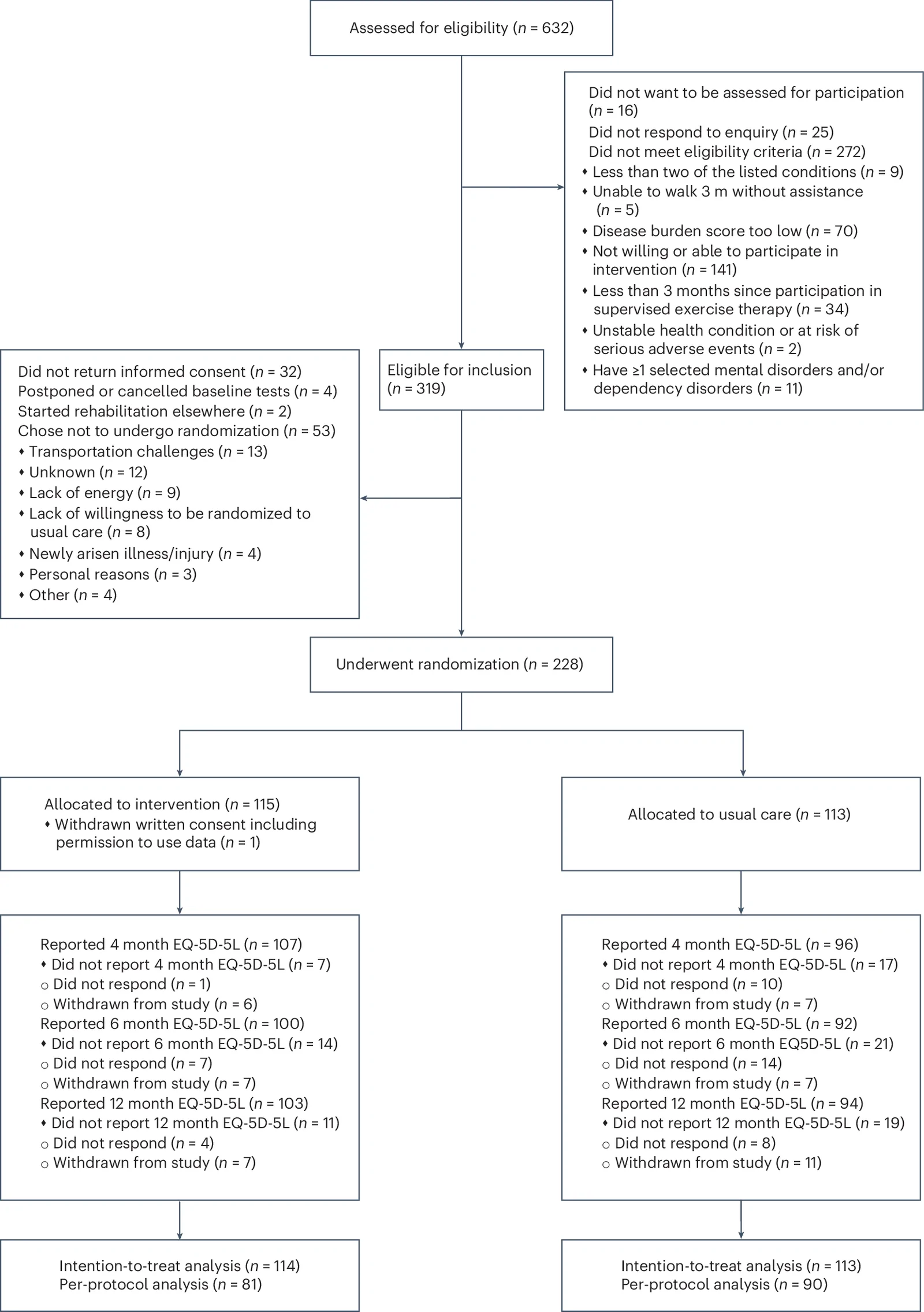
Flow of study patients.

From 18 January 2022 through to 30 May 2023, we assessed 632 patients with multimorbidity. Ultimately, 228 patients were randomized (36% recruitment rate): 115 to the exercise therapy and self-management support intervention group and 113 to the usual care group (Fig. 1). At the 12 month follow-up assessment, 197 participants provided primary outcome data (86% retention rate): 90% in the exercise and self-management intervention group and 83% in the usual care group. Reasons for not providing outcome data at the follow-up assessment are given in Supplementary Appendix 1.

In the exercise therapy and self-management support intervention group, 76% and 75% attended 18 or more exercise therapy and self-management sessions, respectively. Reasons for non-attending the exercise and self-management sessions included illness, vacation and appointments with other healthcare professionals (for example, planned hospital visits).

The characteristics of the participants in the exercise therapy and self-management group and usual care group were comparable (Table 1). Patients had a mean age of 69.8 years (s.d. 8.4), mean BMI of 30.9 kg m^−2^ (s.d. 5.7), a total of 98 (43%) were female, and patients had on average seven long-term conditions (s.d. 3, range 2–19). A list of long-term conditions that participants in both groups had at baseline is given in Supplementary Appendix 2.

**Table 1:** Baseline characteristics

	Exercise and self-management	Usual care
Age (years)	70.0 ± 8.7 (*n* = 114)	69.6 ± 8.1 (*n* = 113)
Body mass index (kg m^−2^)	31.6 ± 6.0 (*n* = 114)	30.3 ± 5.5 (*n* = 113)
Sex, n (%)		
Female	45 (39)	53 (47)
Male	69 (61)	60 (53)
Smoking status, n (%)		
Current smoker	15 (13)	9 (8)
Former smoker	64 (56)	65 (58)
Never smoked	35 (31)	39 (34)
Demographic status, n (%)		
Working/student	10 (9)	10 (9)
Unemployed	3 (3)	0 (0)
Sick leave full-time	2 (1)	4 (4)
Sick leave part-time	3 (3)	5 (4)
Disability pensioner	15 (13)	10 (9)
Early retirement	2 (1)	4 (4)
Retired	79 (69)	80 (70)
Number of chronic conditions per individual	7 ± 2.9 (*n* = 114)	7 ± 2.8 (*n* = 113)
Steps per day, median (IQR)	4,140 (2,019–5,821) (*n* = 106)	3,798 (2,025–6,008) (*n* = 104)
Time spent being physically active with at least light intensity (min per day)	27 ± 9.8 (*n* = 106)	26 ± 9.8 (*n* = 103)
6 min walk test (m)	401 ± 95 (*n* = 111)	393 ± 114 (*n* = 111)
30 s chair-stand test (number of chair stands in 30 s)	10.7 ± 3.7 (*n* = 113)	10.9 ± 4.0 (*n* = 113)
Instruments		
Self-Efficacy for Managing Chronic Disease 6-item Scale (SEMCD6)	6.2 ± 1.8 (*n* = 114)	6.2 ± 1.9 (*n* = 113)
Personal Health Questionnaire Depression Scale (PHQ-8), median (IQR)	5 (3–7) (*n* = 114)	5 (2–8) (*n* = 113)
General Anxiety Disorder-7 (GAD-7), median (IQR)	2.5 (0–4) (*n* = 114)	1 (0–4) (*n* = 113)
Bayliss burden of illness measure	17 ± 8.6 (*n* = 114)	17 ± 8.2 (*n* = 113)
European Quality of Life 5-dimensions 5-level version (EQ-5D-5L), descriptive index	0.709 ± 0.226 (*n* = 114)	0.724 ± 0.211 (*n* = 113)
Overall functioning and disability		
WHO Disability Assessment Schedule (WHODAS 2.0, 12 items)	23 ± 16 (*n* = 114)	23 ± 15 (*n* = 112)
EuroQoL Visual Analog Scale (EQ-VAS)	53.0 ± 18.0 (*n* = 114)	56.9 ± 18.4 (*n* = 113)

Data are given as mean ± s.d. unless otherwise stated.

### Primary outcome

#### Between-group differences from the intention-to-treat analysis of the primary outcome

**Fig. 2:**
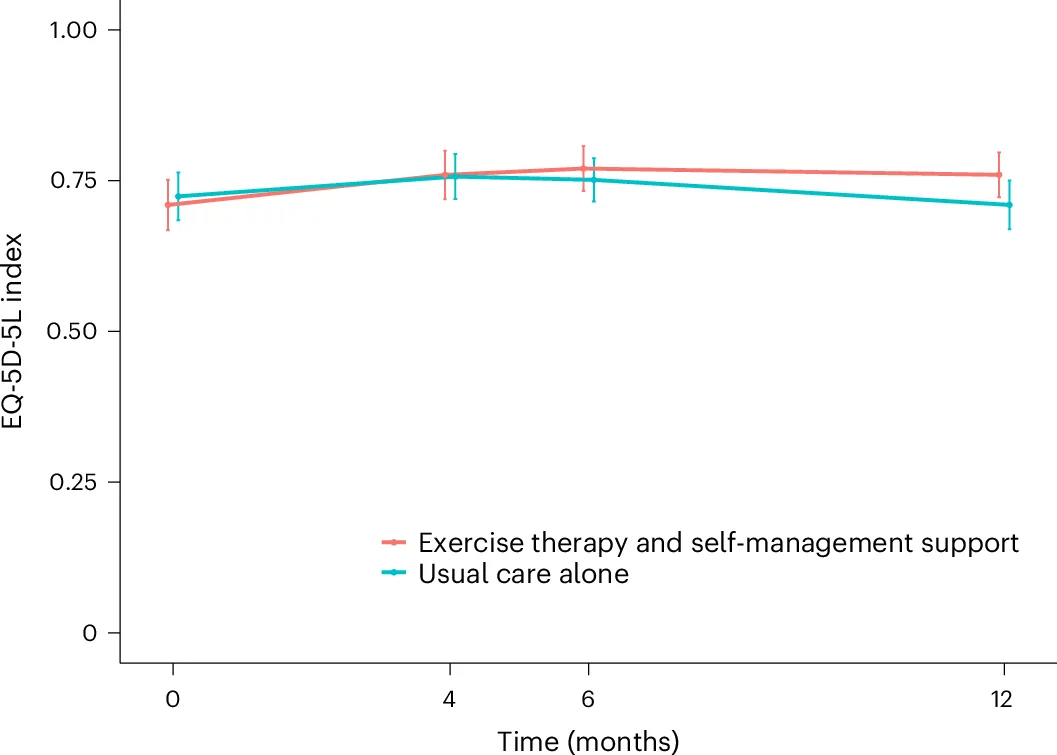
Mean unadjusted EQ-5D-5L index over the 12 month follow-up in the intention-to-treat analysis. Error bars indicate 95% CI. In the intention-to-treat analysis, 114 individuals from the exercise therapy and self-management support group and 113 from the usual care alone group were included. The between-group difference in adjusted mean change from baseline to 12 months was 0.064 points (95% CI: 0.014–0.115).

The between-group analysis identified a statistically significant difference in change between the groups from baseline to 12 months in the primary outcome, the descriptive index of the European Quality of Life 5-dimensions 5-level version (EQ-5D-5L) questionnaire. The mean difference in change was 0.064 points (95% CI: 0.014–0.115) in favor of the exercise therapy and self-management group in both the crude and adjusted analyses (Fig. 2 and Table 2).

**Table 2:** Primary and secondary outcomes at 12 month follow-up (intention-to-treat analysis)

	Total no. of assessments	Mean score at 12 months		Change from baseline to 12 months, mean (95% CI)		Between-group difference in mean change (95% CI)	
		Exercise therapy and self-management group	Usual care group	Exercise therapy and self-management group	Usual care group	Crude	Adjusted^a^
Primary outcome							
EQ-5D-5L, descriptive index	908	0.760	0.710	0.050 (0.012; 0.089)	−0.014 (−0.048; 0.020)	0.064 (0.014; 0.115)	0.064 (0.014; 0.115)
Secondary outcomes							
6 min walk test (m)	583	416.2	412.4	14.5 (3.4; 25.7)	8.3 (−3.9; 20.5)	5.3 (−10.9; 21.5)	5.2 (−11.1; 21.4)
30 s chair-stand test (no. of chair stands in 30 s)	595	12.2	12.0	1.2 (0.7; 1.8)	0.6 (0.1; 1.1)	0.6 (−0.2; 1.3)	0.6 (−0.2; 1.3)
Minutes per day spent being physically active with at least light intensity	557	188.0	185.7	−8.0 (−16.8; 0.7)	1.2 (−10.6; 12.9)	−7.7 (−21.9; 6.4)	−7.6 (−21.8; 6.5)
Steps per day	559	3514	3466	−1029 (−1366; −692)	−1134 (−1524; −744)	177 (−303; 657)	181 (−301; 663)
Bayliss burden of illness measure	815	6.7	6.9	−0.6 (−1.0; −0.2)	−0.5 (−−0.9; 0.005)	−0.15 (−0.8; 0.5)	−0.2 (−0.8; 0.5)
PHQ-8	805	4.2	4.7	−1.0 (−1.7; −0.3)	−0.3 (−1.0; 0.4)	−0.7 (−1.6; 0.3)	−0.7 (−1.6; 0.3)
GAD-7	809	2.1	2.2	−0.4 (−1.0; 0.2)	−0.2 (−0.8; 0.4)	−0.2 (−1.0; 0.7)	−0.2 (−1.0; 0.7)
SEMCD6	810	6.5	6.2	0.2 (−0.2; 0.6)	−0.1 (−0.6; 0.3)	0.3 (−0.3; 0.9)	0.3 (−0.3; 0.9)
WHODAS 2.0, 12 items	905	20.4	22.9	−2.9 (−5.0;-0.8)	−0.3 (−2.4; 1.8)	−2.6 (−5.5; 0.3)	−2.6 (−5.5; 0.3)
EQ-VAS	818	60.8	57.5	7.7 (3.8; 11.7)	0.0 (−3.6; 3.7)	6.9 (1.7; 12.1)	6.9 (1.8; 12.1)

^a^The model was adjusted for the randomization stratification factors (number of chronic conditions (2 or 3+) and recruitment center (hospitals, general practitioners and self-referrals)) by including them as fixed effects.

#### Within-group differences from the intention-to-treat analysis of the primary outcome

While the exercise therapy and self-management group improved by 0.050 points (95% CI: 0.012–0.089) in the descriptive index of the EQ-5D-5L, the usual care group declined by 0.014 points (95% CI: −0.048 to 0.020) from baseline to 12 months (Table 2). In the exercise therapy and self-management group, 32.5% of the participants reached a minimum important difference of 0.074 for people with varying comorbidities^21^, while in the usual care group the corresponding number was 27.4%. At 12 months, 56 (55%) and 36 (40%) reported a patient acceptable symptom state in the exercise and self-management support and the usual care groups, respectively. Of these, 10 (18%) and 16 (44%) reported that they considered their treatment to have failed.

### Secondary outcomes

#### Between-group differences from the intention-to-treat analysis of secondary outcomes

The exercise therapy and self-management group had a statistically significant greater improvement in the EuroQoL Visual Analog Scale (EQ-VAS) than the usual care group at 12 months with a mean adjusted difference of 6.9 points (95% CI: 1.8–12.1). While most of the other secondary outcomes favored the exercise therapy and self-management group, no other statistically significant between-group differences in change from baseline to 12 months were found (*P* > 0.05; Table 2).

#### Within-group differences from the intention-to-treat analysis of secondary outcomes

For the secondary outcomes, both groups reported a statistically significant improvement in the 30 second chair-stand test, while only the exercise therapy and self-management group improved in the 6 minute walk test, Bayliss burden of illness measure, Personal Health Questionnaire Depression Scale (PHQ-8), World Health Organization (WHO) Disability Assessment Schedule and EQ-VAS (*P* < 0.05; Table 2).

### Per-protocol analysis

In the per-protocol analysis (*n* = 171; *n* = 81 in the exercise therapy and self-management support group and *n* = 90 in the usual care group), the between-group difference in change from baseline to 12 months in the primary outcome, the descriptive index of the EQ-5D-5L questionnaire, was not statistically significant. The mean difference in change was 0.032 points (95% CI: −0.032 to 0.088) in favor of the exercise therapy and self-management group in both the crude and adjusted analyses. There was no between-group difference in the secondary outcomes in the per-protocol analysis. Within-group changes were in line with within-group change in the intention-to-treat analysis (Supplementary Appendices 3 and 4).

### Safety

There were 36 and 48 serious adverse events (SAEs) in the exercise therapy and self-management group and in the usual care group, respectively, with no statistically significant between-group difference (*P* = 0.388; Table 3). There were 58 and 50 non-SAEs in the exercise therapy and self-management group and in the usual care group, respectively, with no statistically significant between-group difference (*P* = 0.317; Supplementary Appendix 5).

**Table 3:** Serious adverse events over the 12 month follow-up

	Exercise therapy and self-management group	Usual care group	*P* value^a^
No. of participants affected	30	34	0.528
	Number of events		
Overall	36	48	0.388
Mortality	0	2	0.154
Mental	0	0	1.000
Pulmonary	6	9	0.742
Musculoskeletal	5	8	0.537
Endocrine	1	1	1.000
Cancer	1	6	0.171
Neurological	0	1	0.319
Gastrointestinal	2	7	0.088
Cardiovascular	14	7	0.229
Genitourinary	1	2	0.560
Sensory organs	0	0	1.000
Injury	5	1	0.102
Other	1	4	0.310

^a^Chi-squared test or Wilcoxon signed-rank test.

## Discussion

We have demonstrated a statistically significantly greater improvement in health-related quality of life at 12 months (adjusted mean difference, 0.064 points; 95% CI: 0.014–0.115) in adults with multimorbidity randomized to personalized exercise therapy and self-management support in addition to usual care as compared with usual care alone without increasing the risk of SAEs. This supports our pre-defined hypothesis that personalized exercise therapy and self-management support is superior to usual care alone in improving health-related quality of life at 12 months. However, the clinical relevance of the difference remains unclear, and although the personalized exercise therapy and self-management support group improved in six of the secondary outcomes, only the improvement on self-rated health was statistically significantly greater than that in the usual care alone group (adjusted mean difference, 6.9 points; 95% CI: 1.8–12.1).

The most recent Cochrane review^10^ on interventions for improving outcomes in patients with multimorbidity and an adapted version of the same review excluding studies targeting comorbidity^22^ found little to no effect of a variety of interventions on health-related quality of life and a range of other outcomes. Both reviews concluded that further evidence was needed to guide clinical practice, in particular larger well-conducted trials^10,22^. One of the largest and most well-conducted RCTs on multimorbidity to date, the 3D trial^23^, found no difference in effect on health-related quality of life (assessed with the EQ-5D-5L as in our trial) at 15 months when comparing a 6 monthly patient-centered review by a nurse, a general practitioner and a pharmacist to usual care when focusing on several concepts such as medication adherence, depression symptoms and health management. In contrast, we found a statistically significant greater improvement in health-related quality of life at 12 months (adjusted mean difference, 0.064 points; 95% CI: 0.014–0.115) in adults with multimorbidity randomized to personalized exercise therapy and self-management support as compared to usual care alone. The between-group difference of 0.064 points in our study did not reach the 0.074 minimum important difference identified in a study of people with varying comorbidities^21^. However, the minimum important difference for people with multimorbidity undergoing exercise therapy and self-management support is yet to be defined and is likely to vary greatly due to the heterogeneity of the population^1^. Interestingly, the usual care group seemed to decline in health-related quality of life towards 12 months, while the exercise therapy and self-management support group maintained their effect at 12 months. Extending the findings to a follow-up at, for example, 5 years would enable us to see whether this decline continues, thereby increasing the between-group differences over time. Although based on the within-group analysis (that is, without comparing it to the usual care group), the group randomized to exercise therapy and self-management support improved statistically significantly in six of the secondary outcomes, including chair stands, walking distance, disease burden, depression, disability and self-rated health (*P* < 0.05). However, only the improvement in self-rated health was greater than that in the group randomized to usual care alone. Given the complexity of multimorbidity and its care^1^, and the fact that we included an older population with a range of long-term conditions and a high disease burden, an improvement in health-related quality of life while maintaining other health parameters might be what can be expected from an intervention like this one. Future trials investigating the effects of similar interventions will help to shed light on this and inform future practice.

Our findings add considerably to the current literature and improve the credibility of the findings from two meta-analyses of individuals with an index long-term condition and comorbidity demonstrating a small effect of exercise therapy^19^ and in-person behavioral interventions^24^, respectively, on health-related quality of life immediately after the intervention but negligible results at long-term follow-up. In the current trial we found that the results of the exercise therapy and self-management support intervention at 4 months were maintained at the 12 month follow-up, while the health-related quality of life of the usual care group gradually declined, suggesting that the intervention participants were able to maintain effects over time. The meta-analysis on exercise therapy^19^ and, to some extent, the meta-analysis on in-person behavioral interventions^24^ demonstrated improvements on physical function, one of the expected effects from exercise therapy^25^, and short-term effects on depression and anxiety. However, although we did find statistically significant within-group improvements from personalized exercise therapy and self-management support on objectively measured physical function, self-reported disability and depression (*P* < 0.05), the effects were not greater than that of usual care alone. There may be several explanations for this. First, although not statistically significant (*P* > 0.05), the usual care group also improved at 12 months, potentially owing to natural fluctuations in symptoms, motivation to change lifestyle among those agreeing to be included, and the Hawthorne effect, in which individuals modify their behavior when being studied^26^. All of the above could, however, also affect the results in the intervention group. Second, the baseline values of, for example, the physical function outcomes were better than those of the exercise therapy meta-analysis (397 meters versus 368 meters on the 6 minute walk test)^19^, suggesting that we included individuals with multimorbidity with less disability and thereby less room for improvement in the disability. This is also supported by the fact that the baseline value of health-related quality of life was higher than that of the participants in the 3D trial^23^. In contrast, our participants had on average seven long-term conditions (range 2–19) with a disease burden of 17, while the corresponding numbers were seven (median number of conditions) and 18–19.5 (disease burden) in the 3D trial^23^. This suggests that our population was in fact affected by their long-term conditions at a level similar to that of patients seen in primary care. Importantly, although people with multimorbidity may have several adverse events due to their conditions regardless of the treatment received, our intervention did not increase the risk of adverse events, including SAEs, highlighting the safety of exercise also in this population^27^. Finally, the large heterogeneity in terms of the included conditions and severity of conditions, as well as that in the evaluated interventions in previous RCTs, precludes any direct comparisons^19,24^.

We expect our study to have clinical implications worldwide. It is a large-scale RCT demonstrating an effect on health-related quality of life among a defined population of people with multimorbidity, suggesting exercise therapy and self-management as viable interventions among individuals with multimorbidity. Although our RCT has demonstrated significant results (*P* < 0.05) only in the within-group comparison and not when compared with usual care alone, previous systematic reviews have, as described above, also demonstrated positive physical function outcomes from exercise therapy in a range of long-term conditions and on depression (and anxiety) in the short-term^16,19^. Importantly, the provision of exercise therapy was not associated with an increased risk of SAEs in our RCT. This confirms a previous meta-analysis identifying a reduced risk of SAEs among individuals with an index long-term condition and comorbidity undergoing exercise therapy^19^. Altogether, this supports the recommendation of exercise as medicine for long-term conditions and multimorbidity. However, further high-quality RCTs on exercise therapy in people with multimorbidity are needed to confirm and extend our findings.

By defining multimorbidity as the presence of two or more of the six long-term conditions in our RCT, we also limit generalizability to this population. However, we did not restrict the number or type of other conditions that the individual could have, as shown by the relatively high mean number of long-term conditions among participants. Another potential limitation is the extent of the intervention, which consisted of 24 supervised sessions of 90 min. This might have led to an increased treatment burden, as well as making the intervention less applicable in some healthcare systems and for those with a high treatment burden. In contrast, some might argue that a greater dose (for example, intensity, duration and number of times per week) would be needed to lead to greater changes in the objective measures. The lack of blinding of participants may be associated with a risk of bias, especially given that the primary outcome was self-reported. Future planned analyses from the MOBILIZE study include an evaluation of changes in treatment burden, stress, sleep quality and fatigue and a range of objectively measured outcomes such as inflammation, cholesterol, blood pressure, HbA1c and sleep quantity, as well as a cost-effectiveness analysis. These will be important contributions to gain a deeper understanding of the mechanisms, sustainability and relevance of the MOBILIZE intervention around the world and potentially extend the findings on the self-reported outcomes of health-related quality of life and self-rated health, which were the only outcomes with statistically significantly greater effects (*P* < 0.05) from exercise therapy and self-management support in the current report. Finally, the difference between interventions, with one including 24 additional supervised sessions, which provided greater attention, and the possibility to seek help, from a healthcare professional, could partially explain the between-group difference in effect in health-related quality of life.

A major strength of our study is the pragmatic design, embedded in clinical practice across healthcare sectors, which increases the possibility to implement the intervention after the trial. Furthermore, the extensive development and feasibility phase of MOBILIZE, which follows the Medical Research Council framework for complex interventions^28^, and the inclusion of all relevant stakeholders, as well as the rigorous co-design and methodology of the RCT, ensure the relevance of the intervention for patients and other stakeholders and increase the validity of the study.

In conclusion, our results suggest that personalized exercise therapy and self-management support are more effective than usual care alone in improving health-related quality of life at 12 months in adults with multimorbidity, without compromising safety. However, the clinical relevance of the difference remains unclear, highlighting the need for further research, including confirmatory trials.

## Methods

### Study design

This was a pragmatic, assessor-blinded, multicenter, parallel-group RCT (1:1 treatment allocation) with follow-up assessments at 4, 6 and 12 months conforming to the CONSORT Statement^29^. The CONSORT checklist is given in Supplementary Appendix 6. The study was approved by the Regional Committees on Health Research Ethics for Region Zealand (SJ-857), the Danish Data Protection Agency (Region Zealand, Denmark, REG-015-2020) and pre-registered at ClinicalTrials.gov (NCT04645732). Details of the study, including detailed description of recruitment, treatment and outcomes, have been published in a protocol paper^30^.

The RCT is part of the MOBILIZE study, a 5 year study funded by the European Research Council (https://www.mobilize-project.dk/?lang=en), following the Medical Research Council framework for complex interventions^28^.

### Patients

We enrolled adult patients (aged 18 years or older) with multimorbidity, defined as a diagnosis of at least two of the following conditions: knee or hip osteoarthritis, chronic obstructive pulmonary disease, heart disease (heart failure or coronary heart disease), hypertension, type 2 diabetes mellitus and depression. Patients were not excluded if they had other comorbidities. Furthermore, the patients had to fulfill a range of eligibility criteria.

#### Inclusion criteria

These are as follows: ability to walk 3 m without assistance; a score of ≥3 on the Bayliss Disease Burden: Morbidity Assessment by Self-Report scale^31^ for at least one of the conditions listed in the section above and a score of ≥2 for at least one of the other conditions listed in the section 'Patients' above; and a willingness and ability to participate in a 12 week supervised exercise therapy and self-management program twice a week.

#### Exclusion criteria

These are as follows: participation in supervised systematic exercise for one of their diseases within the last 3 months; presence of an unstable health condition or a risk of SAEs as assessed by a medical specialist; having a terminal condition or a life expectancy of less than 12 months; a categorization of class IV on the New York Heart Association (NYHA) Functional Classification scale (given that the benefits and harms of exercise in this population are uncertain^32^); psychosis disorders, post-traumatic stress disorder, obsessive compulsive disorder, attention deficit hyperactivity disorder, autism, anorexia nervosa/bulimia nervosa and/or dependency disorders; and other reasons for exclusion (unable to understand Danish, mentally unable to participate).

### Recruitment and retention

Participants were recruited from four general practitioners, two psychiatric facilities and six hospital departments in the Region of Zealand, Denmark, as well as by self-referral. Recruitment methods included direct consultations, Facebook ads, local newspaper articles, and other forms of advertising such as posters and handouts.

Individuals visiting one of the recruitment sites who met the eligibility criteria were invited to participate in the RCT. Patient records were also reviewed to identify eligible participants, who were then contacted by phone. Interested individuals were referred to the MOBILIZE project team, and a team member followed up to finalize their inclusion. For self-referrals, a project team member provided detailed information about the study and assessed their eligibility for enrollment by phone. A MOBILIZE-affiliated medical specialist evaluated self-referrals to ensure that they complied with the eligibility criteria on being diagnosed with the listed conditions, and did not have unstable health conditions or were at risk of SAEs.

Once the patients verbally agreed to participate, written informed consent was obtained by the study personnel before they were enrolled in the study.

Based on the results from a systematic review conducted as part of the MOBILIZE project, which aimed to quantify recruitment and retention rates in exercise therapy trials for individuals with multimorbidity^33^, as well as the two most recent Cochrane systematic reviews on recruitment and retention practices^34,35^, a strategy to optimize recruitment and retention was developed. All members of the study team who had direct contact with participants were instructed on when, how, and how often to contact participants to ensure optimal retention throughout the project.

All patients adhering to the eligibility criteria, regardless of sex and gender, were included. We report on the prevalence (that is, n (%)) of the male and female sex (determined by the civil registration number in Denmark) in Table 1, but did not plan or conduct formal analyses related to sex or gender.

Participants received reimbursement for transportation to the outcome assessments and the study treatment.

### Blinding

The outcome assessors, the research assistant handling the data, and the statisticians were blinded to the randomization.

### Randomization

Participants who met the eligibility criteria and signed the informed consent form were randomized in a 1:1 allocation ratio following baseline assessment. The statistician had previously prepared a computer-generated randomization schedule using permuted blocks of four or six individuals, stratified by the number of chronic conditions (2 or 3+) and by recruitment center. Allocation numbers were concealed in opaque sealed envelopes, which were accessible to a study coordinator only after the informed consent and baseline assessment were completed.

### Study treatment

Participants were randomized to one of two groups: a personalized exercise therapy and self-management support program alongside usual care, or usual care alone. All participants continued their current treatment, including any prescribed medications.

#### Exercise therapy and self-management support program

Those assigned to the exercise therapy and self-management support program participated in a 12 week program tailored for individuals with multimorbidity. Prior to initiating the program, each participant had a 60 min one-to-one session with a physiotherapist to introduce the exercise program and set the starting level of the exercises. The program consisted of 24 self-management support sessions (30 min each) followed by 24 supervised exercise sessions (60 min each). The program was co-developed in close collaboration with stakeholders and patient partners as described in full elsewhere^25^. In brief, the research team introduced an initial program based on collected evidence to physiotherapists, patient advocates, carers and medical doctors. We discussed the program’s structure, including proposed exercises, progression and regression levels, and self-management themes. This collaborative approach was maintained throughout the intervention’s development, including the feasibility study, and contributed to shaping the final version tested in this RCT. The program was found feasible and acceptable in people with multimorbidity adhering to the eligibility criteria^25,36^.

Each exercise session included warm-up (8 min), balance (5 min), strengthening (20 min), participant’s choice (additional strengthening exercises, aerobic or functional exercises; 20 min) and cool-down (7 min). The strengthening exercises started with two sets of 10 repetitions in the first week and progressed up to three sets of 12 repetitions in week 11 and 12, in line with the American College of Sports Medicine recommendations^37^. All exercises were personalized across 4–5 difficulty levels and progressed or regressed based on their rate of perceived exertion. The participants were guided by the physiotherapists to achieve the optimal exercise intensity to promote health benefits by the WHO during both the aerobic (that is, levels 12–14 in the BORG scale) and strengthening or functional exercises (that is, levels 5–7 in the OMNI scale). After each set, the participant rated how hard the exercise was, on the OMNI or BORG scale, and if the optimal intensity was not reached or the intensity was rated as too high, the physiotherapists suggested a higher or lower level, respectively. If a participant could not perform level 1 (with full range of motion), a shorter range of motion (as level 0) was recommended (Supplementary Appendix 7). The self-management support sessions combined individual and group sessions and home assignments with one theme per session and aimed to improve self-management skills and motivation to maintain an active lifestyle and better quality of life after the program (Supplementary Appendix 8).

The exercise therapy and self-management support program was delivered at the hospitals in Næstved and Slagelse, at a private practice physiotherapy clinic in Holbæk and at rehabilitation centers in the municipalities of Roskilde and Lolland, by physiotherapists completing a 1 day certification course to deliver the treatment. During the trial, three members of the study team (A.B., M.D. and M.J.) visited, at least once, all of the centers where the MOBILIZE intervention was delivered. The purpose of the visit was to observe whether the exercise therapy and self-management sessions were delivered as intended and to problem-solve any issue that might have occurred during their delivery.

Attendance was tracked, and satisfactory attendance required at least 18 out of 24 exercise therapy and self-management sessions (75%). Participants with lower attendance were included in the intention-to-treat analysis but were excluded from the per-protocol analysis.

#### Usual care

Usual care involved the standard care that participants received outside the study, including any relevant ongoing or additional treatments as determined by their general practitioner or specialist. No study-specific treatment was provided as part of the usual care, nor was there any restrictions on what treatment could be provided, if considered necessary by the treating general practitioner or specialist.

Details of the exercise therapy and self-management support program are given in Supplementary Appendices 7 and 8 and in the published protocol^30^, and further information on the development and feasibility is available in previous publications^25,36^.

### Data collection and outcomes

Self-reported outcomes were collected using electronic or paper-based self-reported questionnaires completed at home (EasyTrial ApS) at baseline, 4 months (approximately 16 weeks, immediately after the treatment program), 6 months and 12 months. If a participant was either unable to access the questionnaire electronically or did not wish to complete it electronically, he or she would receive a paper version by mail along with a prepaid return envelope and would complete it at home. Objectively measured outcomes were collected at baseline, 4 months and 12 months at the intervention sites by blinded assessors who had undergone specific training in the test protocol during a 1 day course. The outcomes were selected to reflect the anticipated impact of the intervention and to include most of the recommended core outcomes for multimorbidity trials^38^.

#### Primary outcome measure

The primary outcome was the descriptive index of the self-reported, EQ-5D-5L questionnaire (5-level version, ranging from −0.758 to 1, higher is better) at 12 months. The EQ-5D-5L is a reliable and valid measure of health-related quality of life^39^. The descriptive index consists of five dimensions (mobility, self-care, usual activities, pain and/or discomfort, and anxiety and/or depression), which each has five levels. The participants self-reported their problems for each of the dimensions, which was then calculated into an overall index value using the Danish EQ-5D-5L value set^40^.

#### Secondary outcome measures

All secondary outcomes were evaluated in all participants.

Functional performance was assessed at baseline and at follow-up at 4 and 12 months using the 6 min walk test and the 30 s chair-stand test, which are commonly used, valid and reliable measures of functional capacity, lower extremity strength and endurance in older adults^41,42^. Steps per day and minutes per day of at least light intensity were measured at the same time points using two Axivity AX3 accelerometers (Axivity Ltd) worn on the right thigh and the wrist of the non-dominant hand. Participants wore them for 7 consecutive days, and valid data required at least 22 hours of wear per day on 3 weekdays and 1 weekend day. The measurement followed a protocol previously found valid and reliable^43,44^.

Self-reported outcomes included the Bayliss burden of illness measure (on a 1–5 scale for each individual condition, summed to a total score for all conditions, higher representing more severe disease burden)^31^, the Personal Health Questionnaire Depression Scale (PHQ-8, range 0–24 points, higher indicating more severe depression)^45,46^, the General Anxiety Disorder-7 (GAD-7, range 0–21, higher indicating more severe anxiety)^45^, the Self-Efficacy for Managing Chronic Disease scale (range 1–10, higher scores indicating higher self-efficacy)^47^, the 12-item WHO Disability Assessment Schedule (WHODAS 2.0; ranging from 0 (no disability) to 100 (full disability)^48,49^, and the EQ-VAS of the EQ-5D-5L questionnaire (range 0–100, higher indicating better self-rated health)^39^. Finally, self-reported patient acceptable symptom state for quality of life was assessed (yes/no)^50^, and in those responding no, treatment failure was assessed (yes/no)^51,52^. The self-reported outcomes instruments have previously been found to be valid and reliable. The Bayliss burden of illness measure was translated into Danish for this study.

Furthermore, the number of adverse events (AEs) and SAEs was self-reported or identified by reviewing medical records during follow-up. AEs and SAEs were defined as any undesirable experience during follow-up leading to contact with the healthcare system. They were categorized according to body system or mortality, and assessed for severity by an adjudication committee (U.B. and P.H.G.) experienced in evaluating AEs (for example, such as pain, falls and fatigue) and SAEs (for example, hospitalization, disability or permanent damage) based on definitions of SAEs from the US Food and Drug Administration^53^.

### Patient and public involvement

Patient and public involvement has been central to all phases of the MOBILIZE project. Throughout, a group of up to eight patients with multimorbidity and carers were involved in key meetings and decisions. They shared their experiences, needs and preferences, and helped shape the intervention, recruit participants and co-develop, feature in and ensure the clarity of the information communicated from the MOBILIZE project. Our approach followed the 'Collaborate' level on the IAP2 Spectrum of Public Participation, emphasizing active partnership^54^. Patient and public involvement was reported according to the GRIPP2 reporting checklist^55^, available in Supplementary Appendix 9.

### Statistical analysis

The statistical analysis plan was made publicly available before data unblinding and analyses^56^. The only deviations from the statistical analyses plan was that AEs and SAEs were compared between groups using the chi-squared test and Wilcoxon signed-rank test and that the per-protocol analyses also excluded patients in both groups who had been hospitalized for more than 7 days or died during follow-up because this would be likely to affect outcomes. Two statisticians blinded to group allocation performed the analyses independently, and the author group followed published procedures for blinded interpretation of the intention-to-treat analyses^57^. The blinded interpretation was made available online prior to unblinding the data^58^. AE, SAE and per-protocol analyses were conducted after breaking the randomization code.

#### Sample size

The RCT was powered to detect a difference of 0.074 points between the two groups in the primary outcome (EQ-5D) from baseline to the 12 month follow-up. While a minimum important difference is yet to be defined for multimorbidity, this difference has previously been identified as the minimum important difference in individuals with various comorbidities^21^. To detect this difference in change, 95 participants per group were required, assuming a common standard deviation of 0.156, with 90% power and an alpha level of 0.05. A total of 228 participants were recruited to account for a potential 20% loss to follow-up.

#### Primary and secondary analyses

Primary and secondary outcomes were analyzed according to the intention-to-treat principle (that is, all patients randomized were included and analyzed according to the group they were randomized to) followed by a per-protocol analysis. The primary intention-to-treat analysis included all patients randomized to the two treatment arms, except for one patient who withdrew written consent and permission to use data. In the per-protocol analysis, participants randomized to exercise therapy and self-management support but who attended fewer than 18 of the 24 sessions, participants in the usual care group who participated in 12 or more supervised exercise therapy sessions for one of their conditions during follow-up, and participants in both groups who underwent major surgery or were hospitalized for more than 7 days during follow-up, were excluded.

Continuous outcomes (including the primary outcome) were analyzed using a repeated measures mixed-effects linear model with participants as random effect, which accounts for missing data^59^. Visit (baseline, 4, 6 and 12 months), treatment arm (Exercise therapy and self-management support program, Usual care) and interaction between visit at time point 12 months and treatment arm were included as fixed effects. The interaction term is the main test of effect. The model was adjusted for the randomization stratification factors (number of chronic conditions (2 or 3+) and recruitment center (hospitals, general practitioners, and self-referrals)) by including them as fixed effects. Missing values were handled according to the guidelines for each specific outcome. If no guideline was available, conditional mean imputation was used. No adjustments for multiplicity were needed^60^.

The number of AEs and SAEs per patient during the 12 month follow-up was compared between groups using the chi-squared test for mortality and the number of persons affected, and the Wilcoxon signed-rank test for all other AEs and SAEs. All analyses were performed in SAS v9.4 (SAS Institute Inc.).

### Reporting summary

Further information on research design is available in the Nature Portfolio Reporting Summary linked to this article.

## Online content

Any methods, additional references, Nature Portfolio reporting summaries, source data, extended data, supplementary information, acknowledgements, peer review information; details of author contributions and competing interests; and statements of data and code availability are available at https://doi.org/10.1038/s41591-025-03779-4.

## Supplementary information

Supplementary Information Supplementary appendices 1–9.

Reporting Summary

## Data Availability

De-identified data and data dictionaries from the MOBILIZE study are available from the principal investigator (Prof. Søren T. Skou, stskou@health.sdu.dk) after publication of the primary publications and until 5 years after the publication of this manuscript. However, restrictions apply to the availability of the de-identified data due to GDPR and study-specific regulations, and access requires a data sharing agreement and a research proposal that will be evaluated by the study group. Requests to access data can expect to be answered within 3 months.

## Code availability

No novel code was used for the current study.

## References

[1] Skou, S. T. et al. Multimorbidity. *Nat. Rev. Dis. Primers* **8**, 48 (2022). 35835758 10.1038/s41572-022-00376-4 PMC7613517

[2] Chowdhury, S. R., Chandra Das, D., Sunna, T. C., Beyene, J. & Hossain, A. Global and regional prevalence of multimorbidity in the adult population in community settings: a systematic review and meta-analysis. *eClinicalMedicine* **57**, 101860 (2023). 36864977 10.1016/j.eclinm.2023.101860 PMC9971315

[3] Head, A., Birkett, M., Fleming, K., Kypridemos, C. & O’Flaherty, M. Socioeconomic inequalities in accumulation of multimorbidity in England from 2019 to 2049: a microsimulation projection study. *Lancet Public Health* **9**, e231–e239 (2024). 38553142 10.1016/S2468-2667(24)00028-8

[4] Barnett, K. et al. Epidemiology of multimorbidity and implications for health care, research, and medical education: a cross-sectional study. *Lancet* **380**, 37–43 (2012). 22579043 10.1016/S0140-6736(12)60240-2

[5] Jani, B. D. et al. Relationship between multimorbidity, demographic factors and mortality: findings from the UK Biobank cohort. *BMC Med.* **17**, 74 (2019). 30967141 10.1186/s12916-019-1305-x PMC6456941

[6] Lehnert, T. et al. Review: health care utilization and costs of elderly persons with multiple chronic conditions. *Med. Care Res. Rev.* **68**, 387–420 (2011). 21813576 10.1177/1077558711399580

[7] Salisbury, C., Johnson, L., Purdy, S., Valderas, J. M. & Montgomery, A. A. Epidemiology and impact of multimorbidity in primary care: a retrospective cohort study. *Br. J. Gen. Pract.* **61**(582), e12–e21 (2011). 21401985 10.3399/bjgp11X548929 PMC3020068

[8] Frølich, A., Ghith, N., Schiøtz, M., Jacobsen, R. & Stockmarr, A. Multimorbidity, healthcare utilization and socioeconomic status: a register-based study in Denmark. *PLoS One* **14**, e0214183 (2019). 31369580 10.1371/journal.pone.0214183 PMC6675513

[9] Ho, I. S. S. et al. Examining variation in the measurement of multimorbidity in research: a systematic review of 566 studies. *Lancet Public Health* **6**, e587–e597 (2021). 34166630 10.1016/S2468-2667(21)00107-9

[10] Smith, S. M., Wallace, E., O'Dowd, T. & Fortin, M. Interventions for improving outcomes in patients with multimorbidity in primary care and community settings. *Cochrane Database Syst. Rev.* **1**, CD006560 (2021). 33448337 10.1002/14651858.CD006560.pub4 PMC8092473

[11] Mair, F. S. & May, C. R. Thinking about the burden of treatment. *BMJ* **349**, g6680 (2014). 25385748 10.1136/bmj.g6680

[12] Noel, P. H., Frueh, B. C., Larme, A. C. & Pugh, J. A. Collaborative care needs and preferences of primary care patients with multimorbidity. *Health Expect.* **8**, 54–63 (2005). 15713171 10.1111/j.1369-7625.2004.00312.x PMC5060269

[13] Bower, P. et al. Multimorbidity, service organization and clinical decision making in primary care: a qualitative study. *Fam. Pract.* **28**, 579–587 (2011). 21613378 10.1093/fampra/cmr018

[14] Smith, S. M., O’Kelly, S. & O’Dowd, T. GPs’ and pharmacists’ experiences of managing multimorbidity: a ‘Pandora’s box’. *Br. J. Gen. Pract.* **60**, 285–294 (2010). 20594430 10.3399/bjgp10X514756 PMC2894403

[15] Parker, S. G. et al. Priorities for research in multiple conditions in later life (multi-morbidity): findings from a James Lind Alliance Priority Setting Partnership. *Age Ageing* **48**, 401–406 (2019). 30892604 10.1093/ageing/afz014

[16] Dibben, G. O. et al. Evidence for exercise-based interventions across 45 different long-term conditions: an overview of systematic reviews. *eClinicalMedicine* **72**, 102599 (2024). 39010975 10.1016/j.eclinm.2024.102599 PMC11247153

[17] Ferrari, A. J. et al. Global incidence, prevalence, years lived with disability (YLDs), disability-adjusted life-years (DALYs), and healthy life expectancy (HALE) for 371 diseases and injuries in 204 countries and territories and 811 subnational locations, 1990–2021: a systematic analysis for the Global Burden of Disease Study 2021. *Lancet* **403**, 2133–2161 (2024). 38642570 10.1016/S0140-6736(24)00757-8 PMC11122111

[18] Allegrante, J. P., Wells, M. T. & Peterson, J. C. Interventions to support behavioral self-management of chronic diseases. *Annu. Rev. Public Health* **40**, 127–146 (2019). 30601717 10.1146/annurev-publhealth-040218-044008 PMC6684026

[19] Bricca, A. et al. Benefits and harms of exercise therapy in people with multimorbidity: a systematic review and meta-analysis of randomised controlled trials. *Ageing Res. Rev.* **63**, 101166 (2020). 32896665 10.1016/j.arr.2020.101166 PMC7116122

[20] Bricca, A., Smith, S. M. & Skou, S. T. Management of multimorbidity. *J. Multimorb. Comorb.* **13**, 26335565231156693 (2023). 36911182 10.1177/26335565231156693 PMC9996704

[21] Walters, S. J., & Brazier, J. E. Comparison of the minimally important difference for two health state utility measures: EQ-5D and SF-6D. *Qual. Life Res.* **14**, 1523–1532 (2005). 16110932 10.1007/s11136-004-7713-0

[22] Smith, S. M., Wallace, E., Clyne, B., Boland, F. & Fortin, M. Interventions for improving outcomes in patients with multimorbidity in primary care and community setting: a systematic review. *Syst. Rev.* **10**, 271 (2021). 34666828 10.1186/s13643-021-01817-z PMC8527775

[23] Salisbury, C. et al. Management of multimorbidity using a patient-centred care model: a pragmatic cluster-randomised trial of the 3D approach. *Lancet* **392**, 41–50 (2018). 29961638 10.1016/S0140-6736(18)31308-4 PMC6041724

[24] Bricca, A. et al. Effect of in-person delivered behavioural interventions in people with multimorbidity: systematic review and meta-analysis. *Int. J. Behav. Med.* **30**, 167–189 (2023). 35484462 10.1007/s12529-022-10092-8 PMC10036283

[25] Bricca, A. et al. Personalised exercise therapy and self-management support for people with multimorbidity: development of the MOBILIZE intervention. *Pilot Feasibility Stud.* **8**, 244 (2022). 36461048 10.1186/s40814-022-01204-y PMC9717541

[26] Berkhout, C. et al. Defining and evaluating the Hawthorne effect in primary care, a systematic review and meta-analysis. *Front. Med.* **9**, 1033486 (2022). 10.3389/fmed.2022.1033486 PMC9679018 36425097

[27] Niemeijer, A. et al. Adverse events of exercise therapy in randomised controlled trials: a systematic review and meta-analysis. *Br. J. Sports Med.* **54**, 1073–1080 (2020). 31563884 10.1136/bjsports-2018-100461

[28] Skivington, K. et al. A new framework for developing and evaluating complex interventions: update of Medical Research Council guidance. *BMJ* **374**, n2061 (2021). 34593508 10.1136/bmj.n2061 PMC8482308

[29] Moher, D. et al. CONSORT 2010 explanation and elaboration: updated guidelines for reporting parallel group randomised trials. *BMJ* **340**, c869 (2010). 20332511 10.1136/bmj.c869 PMC2844943

[30] Skou, S. T. et al. Study protocol for a multicenter randomized controlled trial of personalized exercise therapy and self-management support for people with multimorbidity: the MOBILIZE study. *J. Multimorb. Comorb.* **13**, 26335565231154447 (2023). 36762033 10.1177/26335565231154447 PMC9903016

[31] Bayliss, E. A., Ellis, J. L. & Steiner, J. F. Seniors’ self-reported multimorbidity captured biopsychosocial factors not incorporated into two other data-based morbidity measures. *J. Clin. Epidemiol.* **62**, 550–557 (2009). 18757178 10.1016/j.jclinepi.2008.05.002 PMC2743235

[32] Pelliccia, A. et al. 2020 ESC Guidelines on sports cardiology and exercise in patients with cardiovascular disease: the Task Force on sports cardiology and exercise in patients with cardiovascular disease of the European Society of Cardiology (ESC). *Eur. Heart J.* **42**, 17–96 (2021). 32860412 10.1093/eurheartj/ehaa605

[33] Harris, L. K., Skou, S. T., Juhl, C. B., Jäger, M. & Bricca, A. Recruitment and retention rates in randomised controlled trials of exercise therapy in people with multimorbidity: a systematic review and meta-analysis. *Trials* **22**, 396 (2021). 34127042 10.1186/s13063-021-05346-x PMC8204443

[34] Treweek, S. et al. Strategies to improve recruitment to randomised trials. *Cochrane Database Syst. Rev.* **2**, MR000013 (2018). 29468635 10.1002/14651858.MR000013.pub6 PMC7078793

[35] Gillies, K. et al. Strategies to improve retention in randomised trials. *Cochrane Database Syst. Rev.* **3**, MR000032 (2021). 33675536 10.1002/14651858.MR000032.pub3 PMC8092429

[36] Skou, S. T. et al. Personalised exercise therapy and self-management support for people with multimorbidity: feasibility of the MOBILIZE intervention. *Pilot Feasibility Stud.* **9**, 12 (2023). 36653858 10.1186/s40814-023-01242-0 PMC9847074

[37] Garber, C. E. et al. American College of Sports Medicine position stand. Quantity and quality of exercise for developing and maintaining cardiorespiratory, musculoskeletal, and neuromotor fitness in apparently healthy adults: guidance for prescribing exercise. *Med. Sci. Sports Exerc.* **43**, 1334–1359 (2011). 21694556 10.1249/MSS.0b013e318213fefb

[38] Smith, S. M. et al. A core outcome set for multimorbidity research (COSmm). *Ann. Fam. Med.* **16**, 132–138 (2018). 29531104 10.1370/afm.2178 PMC5847351

[39] Janssen, M. F. et al. Measurement properties of the EQ-5D-5L compared to the EQ-5D-3L across eight patient groups: a multi-country study. *Qual. Life Res.* **22**, 1717–1727 (2013). 23184421 10.1007/s11136-012-0322-4 PMC3764313

[40] Jensen, C. E. et al. The Danish EQ-5D-5L value set: a hybrid model using cTTO and DCE data. *Appl. Health Econ. Health Policy* **19**, 579–591 (2021). 33527304 10.1007/s40258-021-00639-3 PMC8270796

[41] Du, H., Newton, P. J., Salamonson, Y., Carrieri-Kohlman, V. L. & Davidson, P. M. A review of the six-minute walk test: its implication as a self-administered assessment tool. *Eur. J. Cardiovasc. Nurs.* **8**, 2–8 (2009). 18694656 10.1016/j.ejcnurse.2008.07.001

[42] Jones, C. J., Rikli, R. E. & Beam, W. C. A 30-s chair-stand test as a measure of lower body strength in community-residing older adults. *Res. Q. Exerc. Sport* **70**, 113–119 (1999). 10380242 10.1080/02701367.1999.10608028

[43] Donaldson, S. C., Montoye, A. H. K., Tuttle, M. S. & Kaminsky, L. A. Variability of objectively measured sedentary behavior. *Med. Sci. Sports Exerc.* **48**, 755–761 (2016). 26606270 10.1249/MSS.0000000000000828

[44] Migueles, J. H. et al. Accelerometer data collection and processing criteria to assess physical activity and other outcomes: a systematic review and practical considerations. *Sports Med.* **47**, 1821–1845 (2017). 28303543 10.1007/s40279-017-0716-0 PMC6231536

[45] Kroenke, K., Spitzer, R. L., Williams, J. B. & Löwe, B. The Patient Health Questionnaire Somatic, Anxiety, and Depressive Symptom Scales: a systematic review. *Gen. Hosp. Psychiatry* **32**, 345–359 (2010). 20633738 10.1016/j.genhosppsych.2010.03.006

[46] Kroenke, K. et al. The PHQ-8 as a measure of current depression in the general population. *J. Affect. Disord.* **114**, 163–173 (2009). 18752852 10.1016/j.jad.2008.06.026

[47] Ritter, P. L. & Lorig, K. The English and Spanish Self-Efficacy to Manage Chronic Disease Scale measures were validated using multiple studies. *J. Clin. Epidemiol.* **67**, 1265–1273 (2014). 25091546 10.1016/j.jclinepi.2014.06.009

[48] Federici, S., Bracalenti, M., Meloni, F. & Luciano, J. V. World Health Organization disability assessment schedule 2.0: an international systematic review. *Disabil. Rehabil.* **39**, 2347–2380 (2017). 27820966 10.1080/09638288.2016.1223177

[49] Saltychev, M., Katajapuu, N., Bärlund, E. & Laimi, K. Psychometric properties of 12-item self-administered World Health Organization disability assessment schedule 2.0 (WHODAS 2.0) among general population and people with non-acute physical causes of disability: systematic review. *Disabil. Rehabil.* **43**, 789–794 (2021). 31335215 10.1080/09638288.2019.1643416

[50] Pham, T. & Tubach, F. Patient acceptable symptomatic state (PASS). *Joint Bone Spine* **76**, 321–323 (2009). 19525136 10.1016/j.jbspin.2009.03.008

[51] Ingelsrud, L. H., Granan, L.-P., Terwee, C. B., Engebretsen, L. & Roos, E. M. Proportion of patients reporting acceptable symptoms or treatment failure and their associated KOOS values at 6 to 24 months after anterior cruciate ligament reconstruction: a study from the Norwegian Knee Ligament Registry. *Am. J. Sports Med.* **43**, 1902–1907 (2015). 25977523 10.1177/0363546515584041

[52] Skou, S. T. et al. Study protocol for a randomised controlled trial of meniscal surgery compared with exercise and patient education for treatment of meniscal tears in young adults. *BMJ Open* **7**, e017436 (2017). 28827270 10.1136/bmjopen-2017-017436 PMC5724132

[53] US Food & Drug Administration. What is a Serious Adverse Event? 2017. https://www.fda.gov/safety/reporting-serious-problems-fda/what-serious-adverse-event (Silver Spring, 2014).

[54] International Association for Public Participation. IAP2 Spectrum of Public Participation. 2022. https://iap2.org.au/resources/spectrum/ (2018).

[55] Staniszewska, S. et al. GRIPP2 reporting checklists: tools to improve reporting of patient and public involvement in research. *BMJ* **358**, j3453 (2017). 28768629 10.1136/bmj.j3453 PMC5539518

[56] Skou, S. T. et al. Statistical analysis plan for MOBILIZE: a randomized controlled trial of personalized exercise therapy and self-management support for people with multimorbidity (University of Southern Denmark, 2024). Available from: https://portal.findresearcher.sdu.dk/en/publications/statistical-analysis-plan-for-mobilize-a-randomized-controlled-tr

[57] Jarvinen, T. L. et al. Blinded interpretation of study results can feasibly and effectively diminish interpretation bias. *J. Clin. Epidemiol.* **67**, 769–772 (2014). 24560088 10.1016/j.jclinepi.2013.11.011

[58] Skou, S. T. et al. Blinded interpretation of the primary endpoint results from the study: MOBILIZE – a randomized controlled trial of personalized exercise therapy and self-management support for people with multimorbidity (University of Southern Denmark, 2024). Available from: https://portal.findresearcher.sdu.dk/en/publications/blinded-interpretation-of-the-primary-endpoint-results-from-the-s-4

[59] Ranstam, J. et al. Alternative analyses for handling incomplete follow-up in the intention-to-treat analysis: the randomized controlled trial of balloon kyphoplasty versus non-surgical care for vertebral compression fracture (FREE). *BMC Med. Res. Methodol.* **12**, 35 (2012). 22443312 10.1186/1471-2288-12-35 PMC3323461

[60] European Medicines Agency. *Guideline on multiplicity issues in clinical trials* (2017); https://www.ema.europa.eu/en/documents/scientific-guideline/draft-guideline-multiplicity-issues-clinical-trials_en.pdf

