# Supplementary material for: Exercise therapy and self-management support for individuals with multimorbidity: a randomized and controlled trial
Source: Nat Med. 2025 Jun 30;31(9):3176–82. doi: 10.1038/s41591-025-03779-4 (PMC12443619; doi:10.1038/s41591-025-03779-4)
Supplement: Supplementary file 1 — Supplementary appendices 1–9. [file 41591_2025_3779_MOESM1_ESM.pdf]

nature medicine

Supplementary information

https://doi.org/10.1038/s41591-025-03779-4

Exercise therapy and self-management support for individuals with multimorbidity: a randomized and controlled trial

In the format provided by the authors and unedited

Supplementary Appendix 1: Reasons for not providing outcome data

Reasons for not receiving the allocated intervention (exercise therapy and self-management support)

1 Asked to be withdrawn from the study and have all data removed due to other examinations at the doctor

1 Dissatisfied that he would not receive individual treatment for his shoulder issue

1 Could not transport himself to training

1 Could not cope with the idea of participating

1 Did not show up and subsequently did not respond to numerous attempts of contact

Reasons for not receiving the allocated intervention (usual care)

1 Dissatisfied with not being placed in the intervention group

Reasons for not providing primary outcome data at 4-month follow up in the exercise therapy and self-management support group

2 Stopped intervention, feeling mentally unwell

1 Stopped due to chemotherapy treatment

Reasons for not providing primary outcome data at 4-month follow up in the usual care group

1 Hospitalized at the time of testing

8 Reason not specified

3 Dissatisfied with the lack of intervention, therefore, did not attend follow-ups

3 Lack of energy

1 Stress

Reasons for not providing primary outcome data at 6-month follow up in the exercise therapy and self-management support group

3 Also did not complete the 4-month EQ5D

4 Reason not specified

2 Illness and hospitalizations

1 Feeling mentally unwell

Reasons for not providing primary outcome data at 6-month follow up in the usual care group

13 Also did not complete the 4-month follow-up

4 Reason not specified

1 Feeling mentally unwell

2 Lack of energy

Reasons for not providing primary outcome data at 12-month follow up in the exercise therapy and self-management support group

3 Also did not complete the 4- and 6-month EQ5D

2 Also did not complete the 6-month EQ5D

1 Wife hospitalized and weakened

1 Hospitalized and weakened

Reasons for not providing primary outcome data at 12-month follow up in the usual care group

12 Also did not complete the 4- and 6-month EQ5D

2 Fatalities

4 Also did not complete the 6-month EQ5D

1 Reason not specified

1 Lung cancer

Supplementary Appendix 2: Long-term conditions at baseline

	Exercise therapy and self-management group	Usual care group
Hypertension	81	93
Diabetes type 2	54	45
Hip osteoarthritis	44	42
Knee osteoarthritis	62	58
Depression	26	24
Chronic obstructive pulmonary disease	32	33
Chronic heart failure	20	24
Ischemic heart disease	16	17
Other heart diseases	40	36
Anxiety	15	15
Respiratory allergies	13	12
High cholesterol (LDL)	60	68
High triglycerides	10	13
Bronchitis	8	7
Asthma	16	17
Diabetes type 1	1	1
Osteoporosis	11	14
Chronic wounds	2	1
Metabolic disorders	11	14
Chronic back pain or sciatica	53	37
Rheumatoid arthritis	19	13
Rheumatological diseases such as fibromyalgia or lupus	4	8
Stomach problems such as ulcers or gastritis	21	25
Intestinal problems	12	19
Poor blood circulation in the legs	20	29
Hearing loss	47	46
Vision problems	51	44
Lung emphysema	5	5
Cancer in the last 5 years	8	9
Stroke	13	11
Parkinson's disease	0	0
Dementia	0	2
Migraine	11	8
Other neurological diseases	8	6
Anemia	2	3
Number of participants reporting other long-term conditions not mentioned above	26	34

Supplementary Appendix 3: Per protocol results

	Total no. of assessments	Mean score at 12 months in the exercise and self-management group	Mean score at 12 months in the usual care group	Change from baseline to 12 months in the exercise and self-management group	Change from baseline to 12 months in the usual care group	Between-Group difference in mean improvement (crude) (95% CI)	Between-Group difference in mean improvement (adjusted)[2] (95% CI)
Primary outcome							
EQ-5D-5L, the descriptive index	684	0.775	0.731	0.046 (0.002;0.091)	0.014 (-0.020;0.048)	0.032 (-0.023; 0.088)	0.032 (-0.023; 0.088)
Secondary Outcomes							
6-minute walk test (meters)	458	418.0	419.0	12.6 (-0.0;25.3)	15.0 (4.8;25.1)	-2.6 (-18.6;13.4)	-2.5 (-18.5;13.5)
30-second chair-stand test (number of chair stands in 30 seconds)	464	12.4	12.2	1.4 (1.0;1.9)	0.9 (0.3;1.4)	0.6 (-0.1;1.3)	0.6 (-0.1;1.3)
Minutes/day spent being physically active with at least light intensity	436	190.3	187.0	-5.3 (-15.0;4.5)	2.8 (-11.9;17.4)	-5.3 (-22.3;11.7)	-4.8 (-21.74;12.2)
Steps/day	437	3530	3622	-991 (-1354; -627)	-1017 (-1431; -602)	126 (-384; 635)	150 (-359;659)
The Bayliss burden of illness measure	627	6.6	6.8	-0.6 (-1.0; -0.2)	-0.6 (-1.0;-0.03)	-0.08 (-0.7;0.6)	-0.08 (-0.7;0.6)
Personal Health Questionnaire Depression Scale (PHQ-8)	620	3.8	4.4	-1.1 (-1.8; -0.3)	-0.6 (-1.3;0.2)	-0.4 (-1.4;0.6)	-0.4 (-1.4;0.6)
General Anxiety Disorder-7 (GAD-7)	622	2.1	2.0	-0.23 (-1.0;0.4)	-0.5 (-1.1;0.2)	0.2 (-0.7;1.1)	0.2 (-0.7;1.1)
Self-Efficacy for Managing Chronic Disease 6-item Scale (SEMCD6)	622	6.6	6.1	0.1 (-0.3;0.6)	-0.2 (-0.7;0.3)	0.2 (-0.4;0.9)	0.2 (-0.4;0.9)

WHO Disability Assessment Schedule (WHODAS 2.0, 12 items)	681	18.1	21.7	-2.5 (-4.9;-0.1)	-1.0 (-3.3;1.4)	-1.5)-4.8;1.7)	-1.5 (-4.8;1.7)
EQ VAS	629	62.5	59.0	8.1 (3.6;12.6)	2.3 (-1.8;6.3)	5.6 (-0.3;11.4)	5.6 (-0.2;11.5)

[2] The model was adjusted for the randomization stratification factors (number of chronic conditions (2 or 3+) and recruitment center (hospitals, general practitioners, and self-referrals)) by including them as fixed effects.

Supplementary Appendix 4: Figure per protocol results

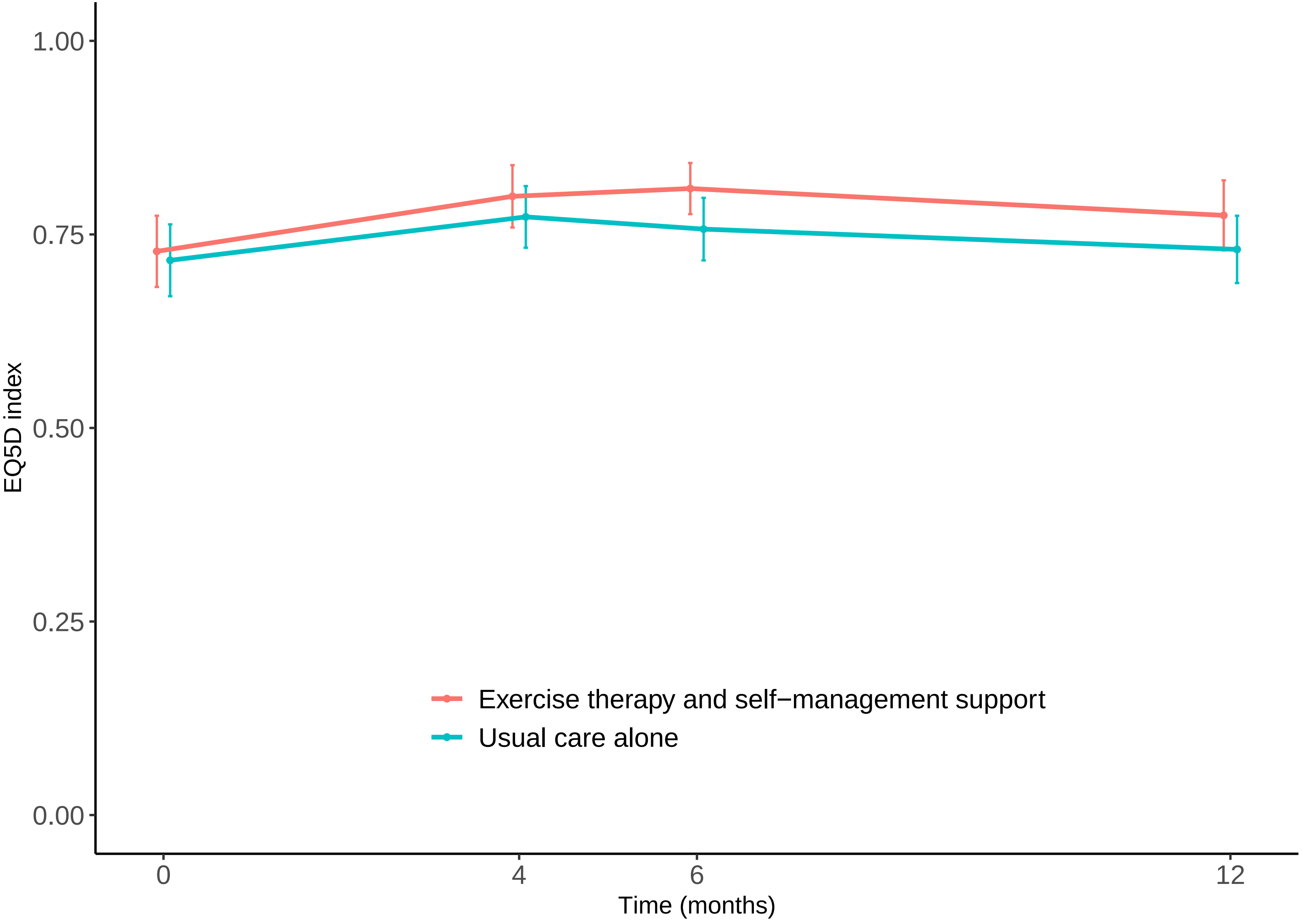

Supplementary Appendix 5: Adverse events (non-serious) over the 12-month follow-up

	Exercise therapy and self-management and group	Usual care group	P Value[1]
Number of participants affected	58	50	0.317
	Number of events		
Overall	79	81	0.543
Mental	1	0	0.324
Pulmonary	5	5	0.738
Musculoskeletal	25	30	0.507
Endocrine	3	3	0.994
Cancer	0	0	1.000
Neurological	4	4	0.723
Gastrointestinal	7	8	0.778
Cardiovascular	9	6	0.407
Genitourinary	1	5	0.156
Sensory organs	0	2	0.156
Injury	13	9	0.366
Other	11	9	0.502

Chi^2-test or Wilcoxon signed-rank test

Supplementary Appendix 6

CONSORT 2010 checklist of information to include when reporting a randomised trial*

Section/Topic	Item No	Checklist item	Reported on page No
**Title and abstract**			
	1a	Identification as a randomised trial in the title	1
	1b	Structured summary of trial design, methods, results, and conclusions (for specific guidance see CONSORT for abstracts)	3-4
**Introduction**			
Background and objectives	2a	Scientific background and explanation of rationale	5-6
	2b	Specific objectives or hypotheses	6
**Methods**			
Trial design	3a	Description of trial design (such as parallel, factorial) including allocation ratio	24
	3b	Important changes to methods after trial commencement (such as eligibility criteria), with reasons	N/A
Participants	4a	Eligibility criteria for participants	24-25
	4b	Settings and locations where the data were collected	29-30
Interventions	5	The interventions for each group with sufficient details to allow replication, including how and when they were actually administered	27-29 (Supp 7 and 8)
Outcomes	6a	Completely defined pre-specified primary and secondary outcome measures, including how and when they were assessed	29-31
	6b	Any changes to trial outcomes after the trial commenced, with reasons	N/A
Sample size	7a	How sample size was determined	32-33
	7b	When applicable, explanation of any interim analyses and stopping guidelines	N/A
Randomisation:			
Sequence generation	8a	Method used to generate the random allocation sequence	27
	8b	Type of randomisation; details of any restriction (such as blocking and block size)	27
Allocation concealment mechanism	9	Mechanism used to implement the random allocation sequence (such as sequentially numbered containers), describing any steps taken to conceal the sequence until interventions were assigned	27
Implementation	10	Who generated the random allocation sequence, who enrolled participants, and who assigned participants to interventions	25-27
Blinding	11a	If done, who was blinded after assignment to interventions (for example, participants, care providers, those	26

		assessing outcomes) and how	
	11b	If relevant, description of the similarity of interventions	N/A
Statistical methods	12a	Statistical methods used to compare groups for primary and secondary outcomes	32-34
	12b	Methods for additional analyses, such as subgroup analyses and adjusted analyses	N/A
**Results**			
Participant flow (a diagram is strongly recommended)	13a	For each group, the numbers of participants who were randomly assigned, received intended treatment, and were analysed for the primary outcome	7 + Fig. 1
	13b	For each group, losses and exclusions after randomisation, together with reasons	7 (Suppl. 1)
Recruitment	14a	Dates defining the periods of recruitment and follow-up	7
	14b	Why the trial ended or was stopped	N/A
Baseline data	15	A table showing baseline demographic and clinical characteristics for each group	Table 1
Numbers analysed	16	For each group, number of participants (denominator) included in each analysis and whether the analysis was by original assigned groups	7 + Fig. 1
Outcomes and estimation	17a	For each primary and secondary outcome, results for each group, and the estimated effect size and its precision (such as 95% confidence interval)	8-10, table 2
	17b	For binary outcomes, presentation of both absolute and relative effect sizes is recommended	9-10, table 3 (Suppl. 5)
Ancillary analyses	18	Results of any other analyses performed, including subgroup analyses and adjusted analyses, distinguishing pre-specified from exploratory	21 (Suppl. 3+4)
Harms	19	All important harms or unintended effects in each group (for specific guidance see CONSORT for harms)	10, table 3 (Suppl. 5)
**Discussion**			
Limitations	20	Trial limitations, addressing sources of potential bias, imprecision, and, if relevant, multiplicity of analyses	14-15
Generalisability	21	Generalisability (external validity, applicability) of the trial findings	14-15
Interpretation	22	Interpretation consistent with results, balancing benefits and harms, and considering other relevant evidence	11-15
**Other information**			
Registration	23	Registration number and name of trial registry	4+24
Protocol	24	Where the full trial protocol can be accessed, if available	24
Funding	25	Sources of funding and other support (such as supply of drugs), role of funders	16

Citation: Schulz KF, Altman DG, Moher D, for the CONSORT Group. CONSORT 2010 Statement: updated guidelines for reporting parallel group randomised trials. BMC Medicine. 2010;8:18.
© 2010 Schulz et al. This is an Open Access article distributed under the terms of the Creative Commons Attribution License (http://creativecommons.org/licenses/by/2.0), which permits unrestricted use, distribution, and reproduction in any medium, provided the original work is properly cited.

*We strongly recommend reading this statement in conjunction with the CONSORT 2010 Explanation and Elaboration for important clarifications on all the items. If relevant, we also recommend reading CONSORT extensions for cluster randomised trials, non-inferiority and equivalence trials, non-pharmacological treatments, herbal interventions, and pragmatic trials. Additional extensions are forthcoming: for those and for up-to-date references relevant to this checklist, see www.consort-statement.org.

Supplementary Appendix 7

MOBILIZE

Training programme manual for people with multiple chronic diseases (multimorbidity)

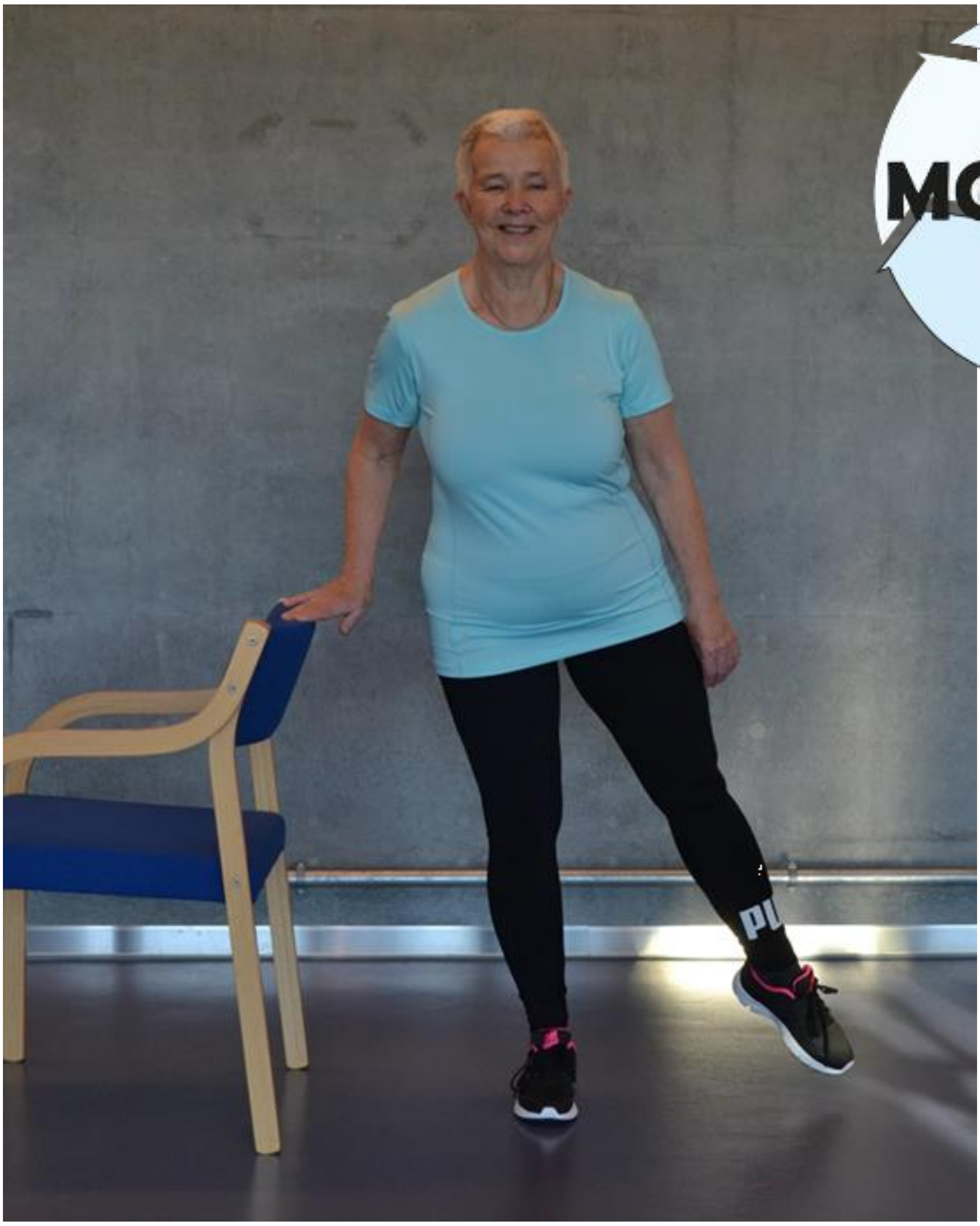

Photo: MOBILIZE

BETTER HEALTH FOR PEOPLE WITH MULTIPLE DISEASES
THROUGH INDIVIDUALISED TRAINING AND PATIENT EDUCATION

Contents

Overview of training programme 4
Level of effort and progression 6
Strength training and functional exercises 6
Cardio training 7
Balance training 8
Pain 8
Serious adverse effects or events 9
Training diary 10
Retention in training and self-management 12
The training exercises in MOBILIZE 14
Warm-up (8 min.) 14
Balance exercises (5 min.) 15
Exercise 1. From heel to toe 15
Exercise 2. 'Walking a tightrope' 17
Strength exercises (20 min.) 18
Exercise 3. Get-up-and-sit-down exercises/squats 18
Exercise 4. Back exercise 20
Exercise 5. Sliding exercise/step forward or backward 22
Option A, Fitness (20 minutes) 24
Option B, Strength training (20 minutes) 25
Exercise 1B. Arm stretching 25
Exercise 2B. Pelvic lift 27
Exercise 3B. Abdominal exercise 29
Alternative abdominal exercise 30
Option C, Functional exercises (20 minutes) 32
Exercise 1C. Stairway 32
Exercise 2C. Grandchild lifting 34
Exercise 3C. Carrying shopping bags 35
Cool-down (7 minutes) 36
Exercise 1. Child's position 36
Exercise 2. Standing forward bend 36
Exercise 3. Stretching upper body 37

Alternative cool-down exercises 37
Contraindications to exercise 38

Overview of training programme

This training programme contains exercises that should be performed under the supervision of a physiotherapist. The goal of this training programme is to improve the participant's physical and psychosocial health.

The programme consists of two parts:

All participants perform these elements – warm-up, balance exercises, strength exercises for the lower and upper body and cool-down.

Each participant can also choose between three different training options: cardio training, strength training or functional exercises.

The programme is a circuit training programme in which each exercise, including breaks, is completed before the participant moves on to the next exercise. This is important to remember for the sake of completing the participant's training diary.

Part 1

Warm-up (8 minutes) *and*

Balance exercises (5 minutes) *and*

Strength exercises (20 minutes)

Part 2 – Participant's Choice

Cardio training (option A – 20 minutes) *or*

Strength training (option B – 20 minutes) *or*

Functional exercises (option C – 20 minutes) *and*

Cool-down (7 minutes)

Each exercise has different levels (ranging from 1 to 4 or 5), and the physiotherapist helps the participants find their ideal starting point. If a participant cannot perform level 1 (with full range of motion), a shorter range of motion (as level 0) is used, and this is noted in the notes in the training diary.

After each set, the participant rates how hard the exercise was, and based on this, the individual level is determined.

Throughout the course, make sure to tailor the training to the individual participant's needs. For example, it may be necessary to reduce the number of repetitions or the load if the participant experiences pain. Conversely, it may also be relevant, for example, to combine multiple resistance bands in order to challenge the participant sufficiently.

In addition to training in MOBILIZE, participants are encouraged to comply with the Danish Health Authority's recommendations on physical activity.

Level of effort and progression

Strength training and functional exercises

The OMNI scale, which ranges from 0 (extremely easy) to 10 (extremely hard), is used to assess how strenuous a strength exercise is perceived (Figure 1).

The participant must feel that the level was 'somewhat hard' (between 5 and 7 on the scale) after each completed set. Progression is achieved by increasing the number of repetitions and sets or using a stronger resistance band or heavier weight. As a guide, the participant will be able to increase the level every two weeks (Table 1). However, always assess progression and possible regression individually for each participant.

Set	Repetitions	Week
2	10	1 and 2
2	12–15	3 and 4
3	12	5 and 6
**2**	**10**	**7 and 8 (increased level)**
2	12–15	9 and 10
3	12	11 and 12

Table 1. The American College of Sports Medicine's recommended progression for strength training.

Focus on appropriate joint position and full range of motion (ROM). If a participant is unable to perform level 1, shorter movements are used.

The participant should use 3 sec. in the concentric phase + 3 sec. in the eccentric phase.

The functional exercises are progressed according to the same principles as the strength exercises – except for the 'Carrying shopping bags' exercise, which follows the guidelines below (Table 2). However, always evaluate each participant individually:

Set	Walking distance (metres)	Week
2	20	1 and 2
2	25	3 and 4
3	30	5 and 6
**2**	**20**	**7 and 8 (increased level)**
2	25	9 and 10
3	30	11 and 12

Table 2. Progression in connection with the functional exercise 'Carrying shopping bags'.

Cardio training

To provide guidance as to when a participant should progress their fitness training, level 14–15 (the talking limit) on the Borg scale is used. The intensity corresponds to moderate physical activity such as brisk walking or other forms of physical activity that require moderate effort, increase the heart rate and make the participant somewhat short of breath. At this intensity, the participant can typically speak,but not sing (Table 3).

Rate of Perceived Exertion

Point	Effort	Description
6	No Exertion	Little to no movement, very relaxed
7	Extremely Light	Able to maintain pace
8		
9	Very Light	Comfortable and breathing harder
10		
11	Light	Minimal sweating, can talk easily
12		
13	Somewhat Hard	Slight breathlessness, can talk
14		Increased sweating, still able to hold conversation but with difficulty
15	Hard	Sweating, able to push an still maintain proper form
16		
17	Very Hard	Can keep a fast pace for a short time period
18		
19	Extremely Hard	Difficult breathing, near muscle exhaustion
20	Maximally Hard	STOP, exercising, total exhaustion

Table 3. Borg's 6–20 Rate of Perceived Exertion scale.

Balance training

Perform 3 sets of up to 12 repetitions in each set of exercise 1 (from heel to toe).

Walk 3 sets of up to 5 metres in exercise 2 (‘walking a tightrope’).

Rest for approx. 30 seconds between each set.

The balance exercises, which aim to improve balance and sensorimotor control, are progressed according to the following guidelines:

When the participant can complete all three sets safely and with good control, they are promoted to the next level.

Level 1: Perform the exercise with support

Level 2: Perform the exercise without support

Level 3: Perform the exercise on an uneven surface

Level 4: Perform the exercise with your eyes closed

Pain

The pain management model below goes from 0–10 (where 0 equals no pain and 10 the worst pain imaginable) and can be used to assess the intensity of pain in patients affected by pain after each training session. Pain up to level 2 is considered safe (green); pain up to level 5 is considered acceptable (yellow); while pain above level 5 carries a risk of getting worse.

Pain management model

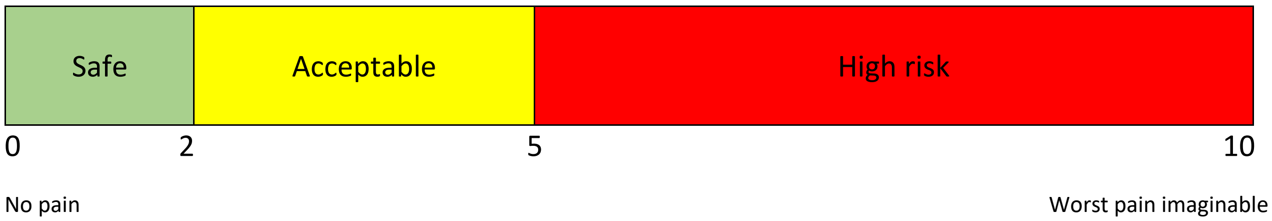

Plan the training based on the individual participant’s pain level. If necessary, reduce the number of repetitions, load etc.

Serious adverse effects or events

If any serious adverse effects or events occur for a participant during the project, the physiotherapist must notify MOBILIZE immediately.

According to the FDA, a serious adverse effect or event includes*: An adverse event that is fatal, life-threatening, requires hospitalisation or prolongation of previous hospitalisation, or results in persistent or significant impairment or incapacity for work.

All side effects must be recorded in the form on page 2 of the training diary.

* https://www.fda.gov/safety/reporting-serious-problems-fda/what-serious-adverse-event

Training diary

During the individual introduction, the physiotherapist introduces the training diary, which must be completed at each training session.

Fill in the date and the participant's attendance at training and patient education here. The reason for absence or if the participant experiences adverse effects can also be noted here (see also 'Serious adverse effects or events' above).

The training data below is recorded.

Note:
For exercises that involve training both legs/sides, only data for the weakest side must be recorded.

Balance exercises

- Level (1–4)
- Number of sets (1–3)
- For 'from heel to toe': number of repetitions (1–12)
- When 'walking a tightrope': number of metres walked
- Performed safely and with good control

Strength exercises

- Level (1–5)
- Number of sets (1–3)
- Number of repetitions (1–15)
- Level of exertion according to the OMNI scale (0–10)

Fitness

- Level (1–4)
- Duration (minutes)
- Activity
- Level of exertion according to the Borg scale (6–20)

Functional exercises

- Level (1–4)
- Number of sets (1–3)
- For 'stair climbing' and 'grandchild lifting': number of repetitions (1–15)
- For 'carrying shopping bags': number of metres walked
- Level of exertion according to the OMNI scale (0–10)

When a participant has completed their last training session, their training diary is scanned and saved locally on the therapist's computer. The document is then uploaded to MOBILIZE SharePoint under the folder Træning / Udfyldte træningsdagbøger / Udfyldte træningsdagbøger_Slagelse (or _Holbæk, _Roskilde, _Næstved, _Lolland).

Use the participant's ID no. to name the scanned diary: [participant ID no.]_diary. The scanned document is subsequently deleted from the therapist's computer. The paper version is kept in a locked cabinet and destroyed when the study is completed (July 2024).

Retention in training and self-management

Patients with multiple diseases face many challenges in their lives. Upon inclusion in the project, the MOBILIZE team talks to each individual participant to clarify any potential barriers to training. Even so, participating in a long-term training and education programme can seem overwhelming, and it is therefore important to support and follow the participants closely.

A participant is contacted by telephone by the physiotherapist if:

- The person concerned does not show up without cancellation
- In the event of three consecutive no-shows (even if a cancellation has been reported)

Ask whether the no-shows are due to reasons where the problem can be remedied. If necessary, contact the MOBILIZE team for advice (see also: Tool for use in the event of no-show(s) in the MOBILIZE project).

If a participant experiences pain or other physical challenges, the physiotherapist will initially perform an assessment and adapt the training accordingly. If this does not help, contact MOBILIZE, who will then contact the participant regarding further participation.

The MOBILIZE programme is an individualised process for each participant. Keep your focus on this throughout the course. The teams run on a rolling intake basis. It is therefore important to do a quick round of names when a new participant arrives. This helps them get off to a good start on the team.

Strategies that help you meet your participants' expectations and retain them:

- Be authentic and empathetic when communicating with participants
- Assess and address participants' concerns, questions and expectations
- Provide clear and correct information about diseases, diagnoses and treatments
- Be proactive and ask follow-up questions (ask participants to summarise the information you have given them) to avoid misunderstandings
- Strengthen positive associations and minimise negative associations between the intervention and contextual factors
- Inform participants about the evidence for the effect of exercise on pain and general health
- Think about how you describe the desired treatment results. In particular, think about how you describe possible adverse events, etc. to minimise the nocebo effect
- Work on strategies for handling adverse events
- Refer to our patient education materials, which contain evidence-based information, and avoid unsubstantiated comments that can increase anxiety and uncertainty
- Show you are a professional, e.g. by wearing your workplace's uniform

- Touching during the exercises should always be accompanied by verbal guidance – for example, put a hand behind the participant's back during the back exercise to remind them which muscles need to be activated.

A good group dynamic is also important to retain the participants in the MOBILIZE project.<br><br>Feel free to facilitate communities among the participants that can help them to continue training together after completion of the project.<br>This can be accomplished by, for example, identifying one or more participants who can arrange/be responsible for training or group formation among interested participants after the MOBILIZE course.

At the last training session for a participant, there is a brief discussion about how to ensure the participant can take things further after MOBILIZE. Review the points below. Have a laminated version of the sheet 'At the last training session' ready, which can be presented and reused.

- How have you fared with the goal you set for yourself at the start of the MOBILIZE project?
- Did you get any good ideas from the thematic module with advice on how to progress and incorporate physical activity into your everyday life after MOBILIZE?
- Do you have a concrete plan for how to proceed with this?
- Do you have someone who can help you follow this plan and someone who can motivate you?
- Finally, do you have any questions?
- Feel free to call the MOBILIZE team if you have any questions or need advice.

The training exercises in MOBILIZE

Below you will find the training programme with pictures and descriptions/instructions.

If necessary, it can be printed out and given to the participant to support their training diary.

Warm-up (8 min.)

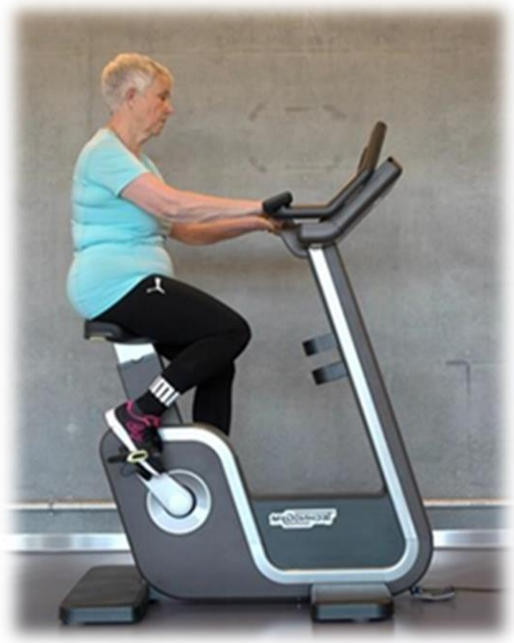

Cycling on an exercise bike, walking or other physical activity (e.g. rowing) depending on available training equipment and your wishes.

The degree of exertion is assessed subjectively and is gradually increased over the 8 minutes, until the level 'somewhat hard' on the Borg scale is reached – i.e. you can talk but not sing.

Balance exercises (5 min.)

Exercise 1. From heel to toe

Level 1. Stand on the floor with your feet slightly apart. Place a hand on the chair for support. Slowly lift your heels off the floor so that you are standing on your toes. Return to the starting position and then lift your toes so that you are standing on the back of your foot.

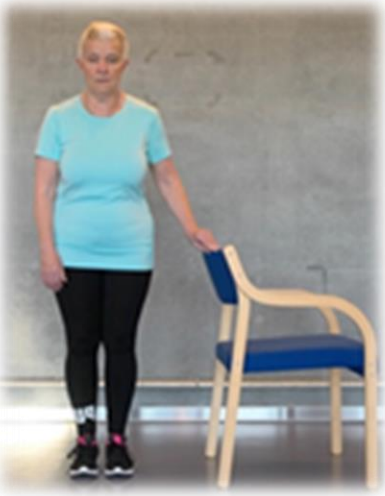
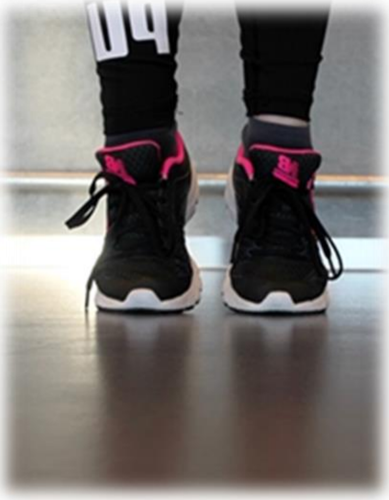
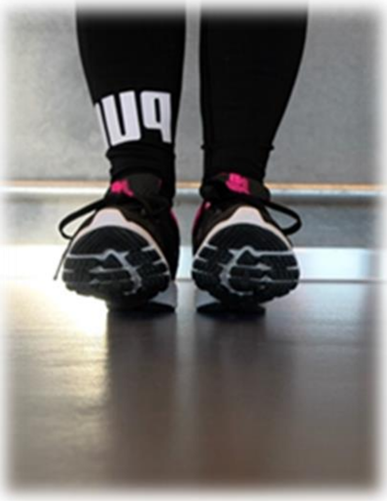

Level 2. Stand on the floor with your feet slightly apart and without support from the chair. Slowly lift your heels off the floor so that you are standing on your toes. Return to the starting position and then lift your toes so that you are standing on the back of your foot.

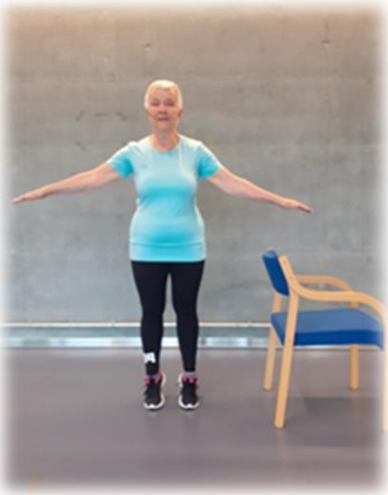
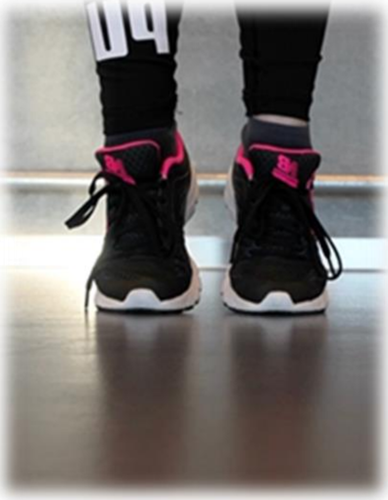
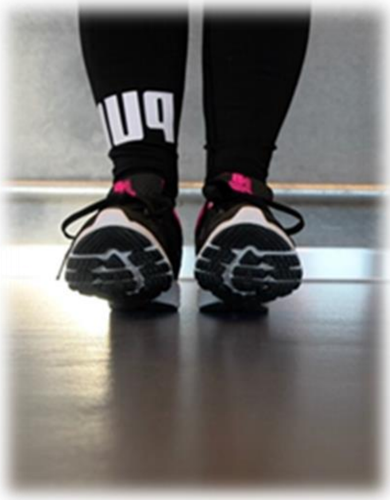

Level 3. Stand on an uneven surface (e.g. a pillow or a folded towel) with your feet slightly apart and without support from the chair. Slowly lift your heels off the floor so that you are standing on your toes. Return to the starting position and then lift your toes so that you are standing on the back of your foot.

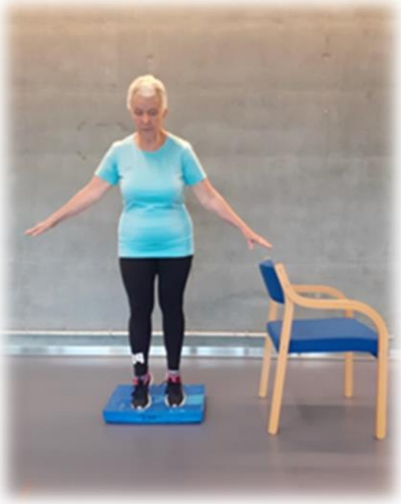
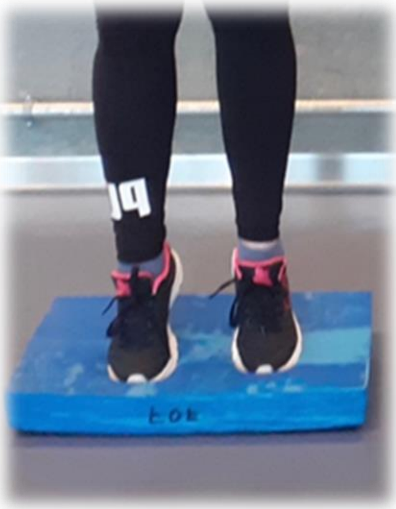
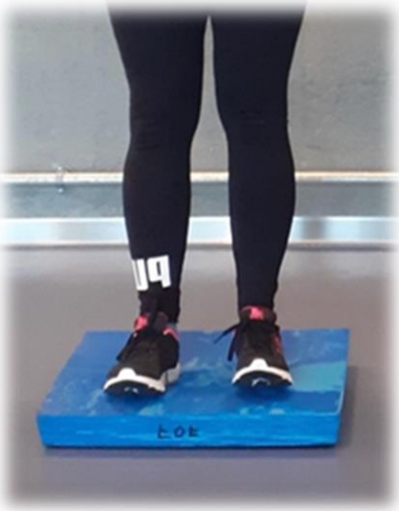

Level 4. Stand on the floor with your feet slightly apart. Place a hand on the chair for support and close your eyes. Slowly lift your heels off the floor so that you are standing on your toes. Return to the starting position and then lift your toes so that you are standing on the back of your foot.

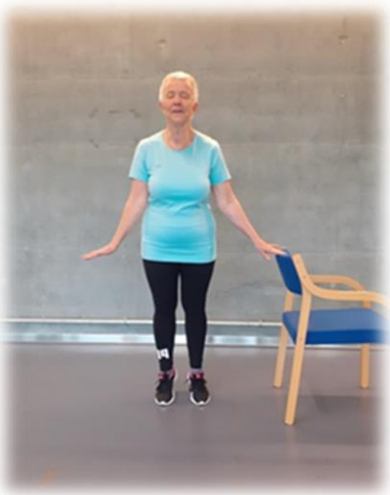
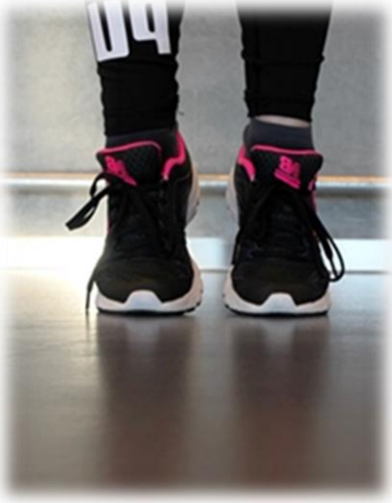
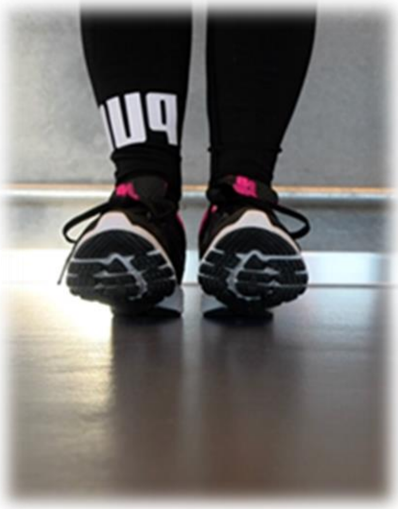

Exercise 2. 'Walking a tightrope'

Level 1. Using the wall for support. Extend your arms out to the side. Stand straight and look straight ahead. Place one foot directly in front of the other (heel to toe) as if you are walking a tightrope.

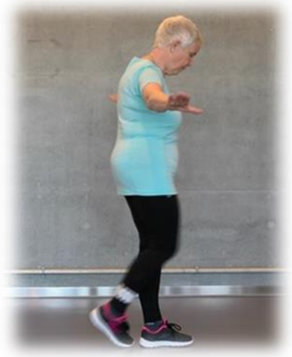

Level 2. Without using the wall for support. Extend your arms out to the side. Stand straight and look straight ahead. Place one foot directly in front of the other (heel to toe) as if you are walking a tightrope.

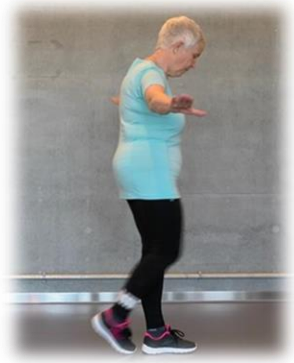

Level 3. With your eyes closed. Use the wall to support yourself if necessary. Extend your arms out to the side. Stand straight and look straight ahead. Place one foot directly in front of the other (heel to toe) as if you are walking a tightrope.

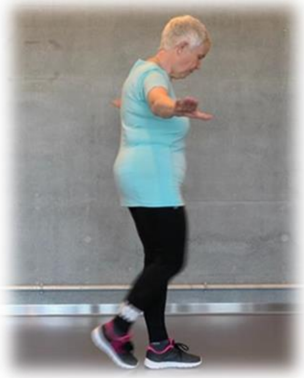

Strength exercises (20 min.)

Exercise 3. Get-up-and-sit-down exercises/squats

Level 1. With arm support. Start in a seated position with your feet parallel and hip-width apart. Put equal weight on both legs and use light hand support from the seat or armrest of the chair. Get up from the chair and then sit down slowly again. Avoid tipping back into the chair. Stay focused on the appropriate positioning of your hip, knee and ankle joints.

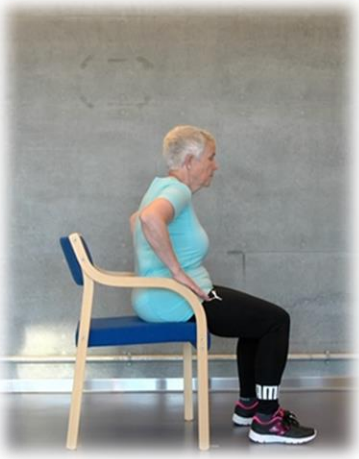
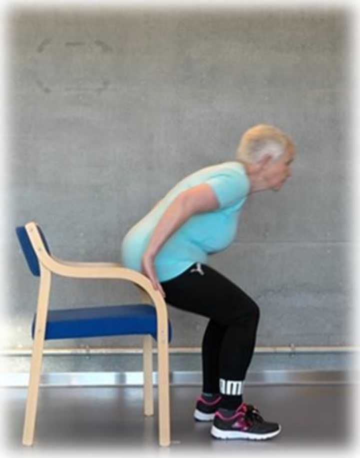
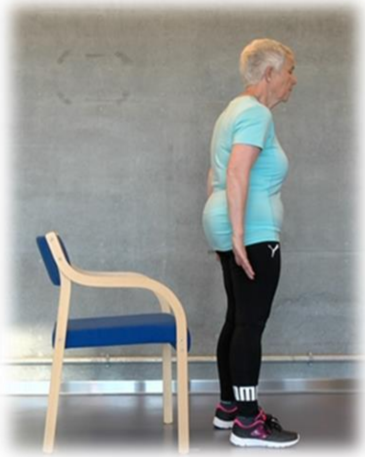

Level 2. Without arm support. Start in a seated position with your arms crossed in front of your chest and your feet parallel, hip-width apart. Put equal weight on both legs. Get up from the chair and then sit down slowly again. Avoid tipping back into the chair. Stay focused on the appropriate positioning of your hip, knee and ankle joints.

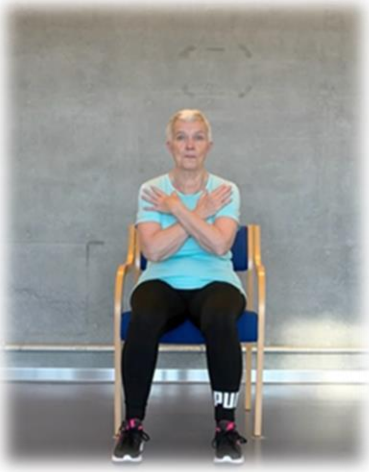
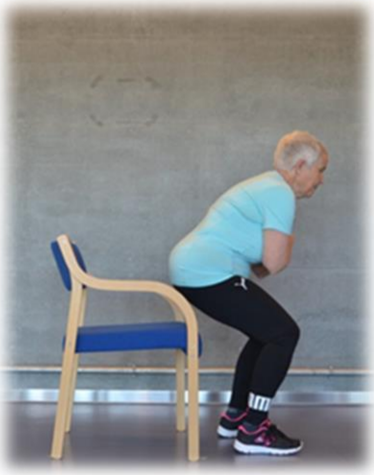
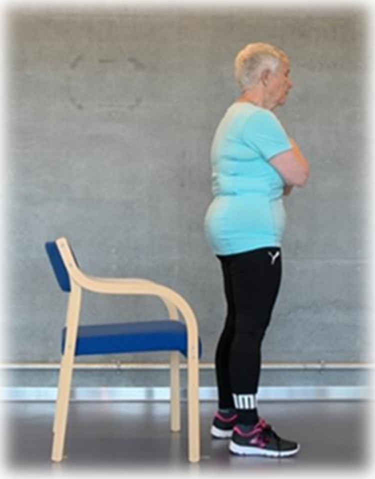

Level 3. Without arm support. Start in a seated position with one leg placed slightly in front of the other. Keep the weight and focus on the back leg. Get up from the chair and then sit down slowly again. Avoid tipping back into the chair. Stay focused on the appropriate positioning of your hip, knee and ankle joints. Switch legs and repeat the exercise.

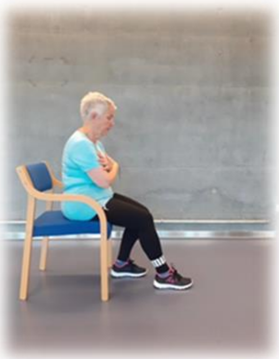 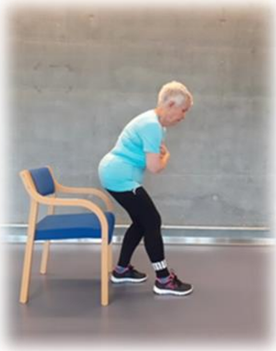 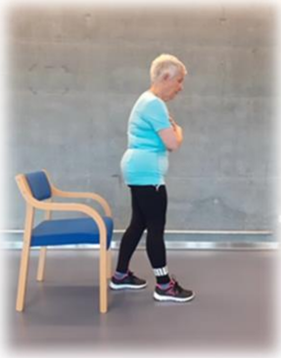

Level 4. Squat. Stand on the floor with your feet slightly apart. Put equal weight on both legs. Stay focused on the appropriate positioning of your hip, knee and ankle joints. Bend your hips and knees until your knees bend about 90 degrees and stand back up. Keep your back straight and look straight ahead.

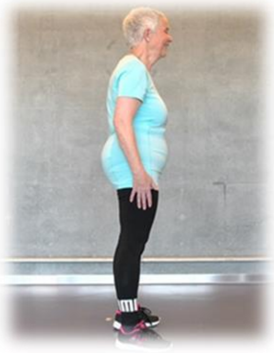 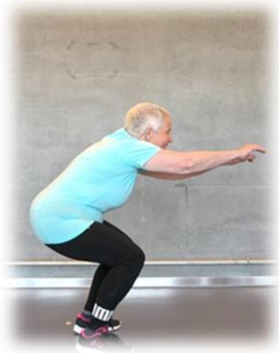 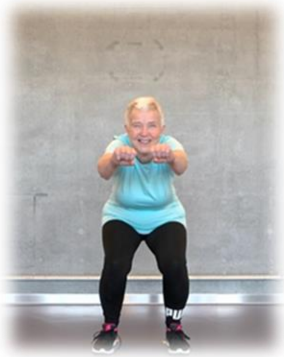

Level 5. Squat with resistance band. Stand on the floor with your feet slightly apart. Put equal weight on both legs. Place a resistance band under your feet. Grasp the left end of the band with your left hand and the right end with your right hand. Stay focused on the appropriate positioning of your hip, knee and ankle joints. Bend your hips and knees until your knees bend about 90 degrees and stand back up. Keep your back straight and look straight ahead.

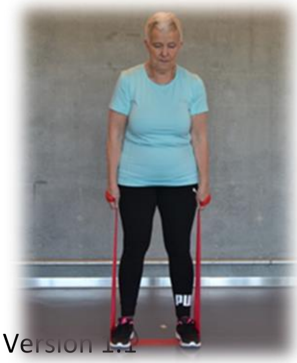 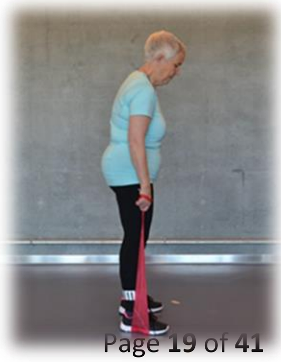 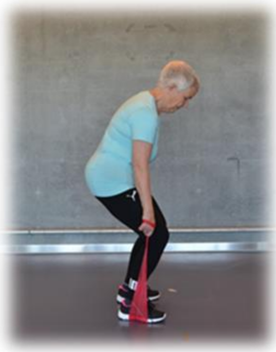

Exercise 4. Back exercise

Level 1. Start in a seated position with your feet parallel and hip-width apart. Stretch your arms and hold the resistance band. Move your shoulders back and down and tense your abdomen. Pull your elbows back in a rowing motion. Keep your back straight.

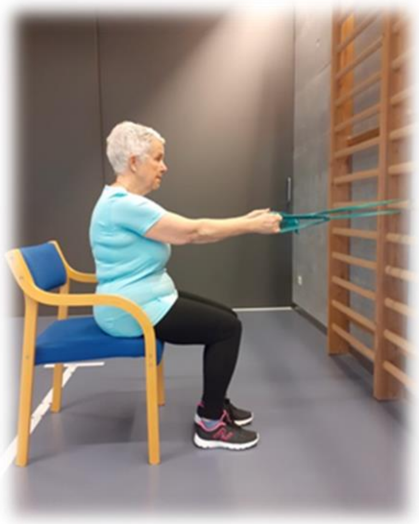

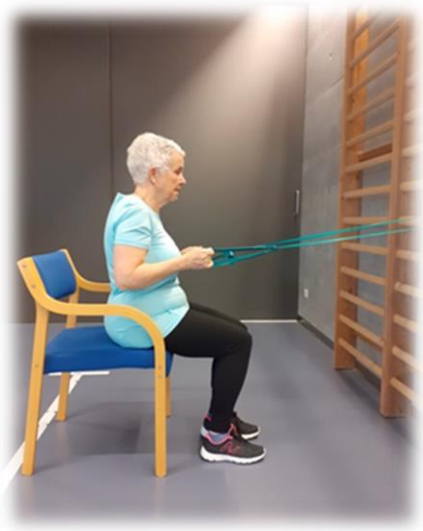

Level 2. Start in a seated position on a large exercise ball with your feet parallel and hip-width apart. Stretch your arms and hold the resistance band. Move your shoulders back and down and tense your abdomen. Pull your elbows back in a rowing motion. Keep your back straight.

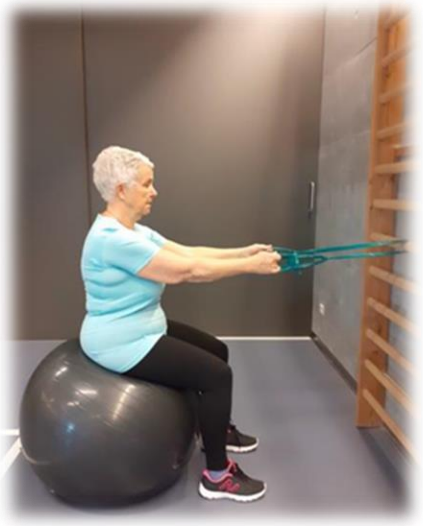

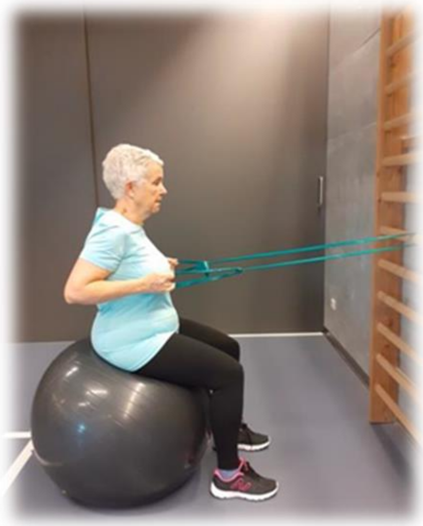

Level 3. Stand on the floor with your feet slightly apart. Put equal weight on both legs. Stretch your arms and hold the resistance band. Move your shoulders back and down and tense your abdomen. Pull your elbows back in a rowing motion. Keep your back straight.

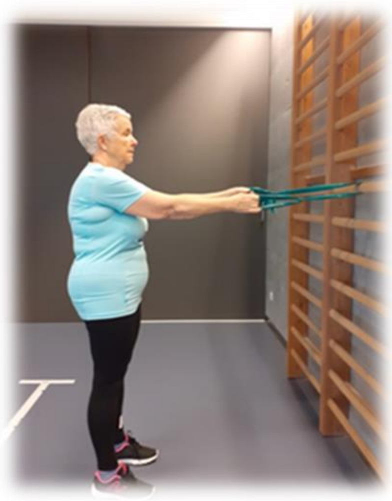

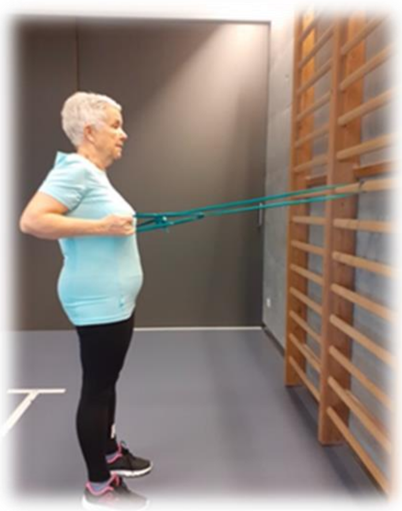

Level 4. Like level 3, but with a resistance band with greater resistance.

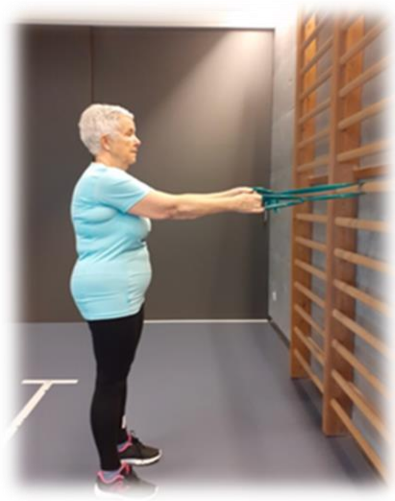

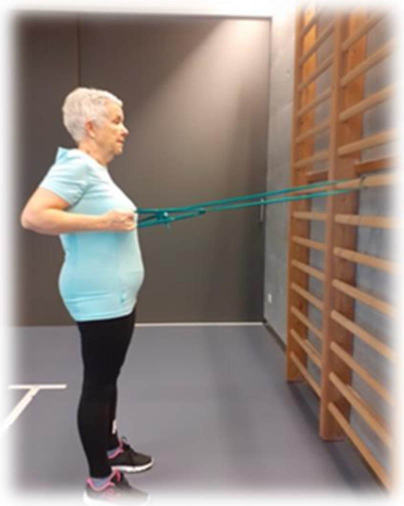

Exercise 5. Sliding exercise/step forward or backward

Level 1. Stand up, put your weight on one leg and place your other leg on a smooth surface. Move your leg on the smooth surface backwards and then return to the starting position. Bend at the knees and hips on your weight-bearing leg. Focus on the appropriate positioning of your hip, knee and ankle joints. If necessary, use a chair for support. Switch legs and repeat the exercise.

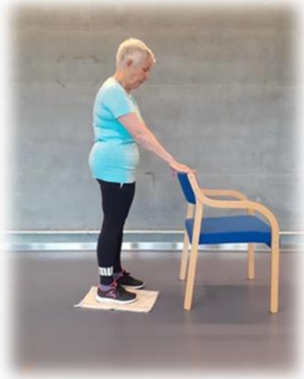
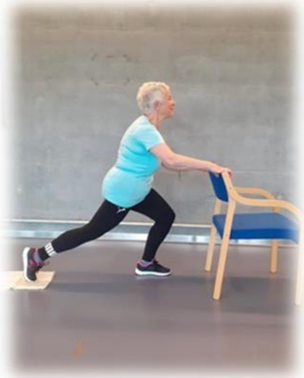

Level 2. As in level 1, but with your weight-bearing foot on an uneven surface. If necessary, use a chair for support. Switch legs and repeat the exercise.

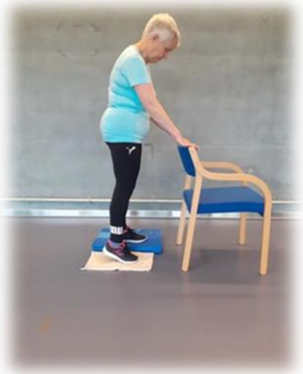
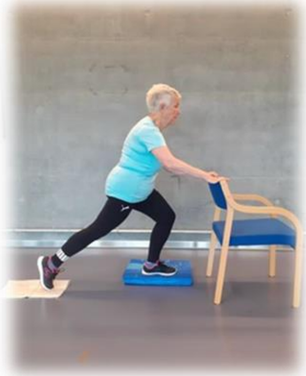

Level 3. Take a large step forward or backward and give way in the knee and hip joints of your front leg, so that your knees and hips bend. Focus on the appropriate positioning of your hip, knee and ankle joints. Push yourself back to the starting position. Switch legs and repeat the exercise.

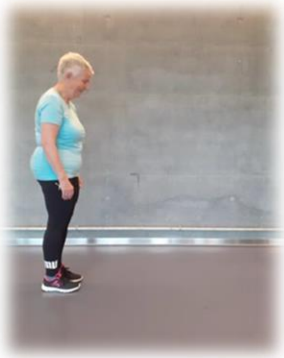
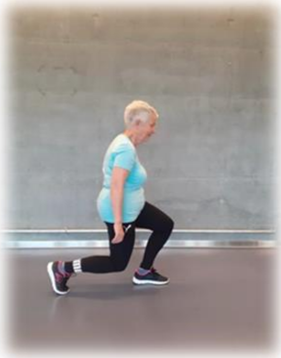

Level 4. With a dumbbell in each hand. Take a large step forward or backward and give way in the knee and hip joints of your front leg, so that your knees and hips bend. Focus on the appropriate positioning of your hip, knee and ankle joints. Push yourself back to the starting position. Switch legs and repeat the exercise.

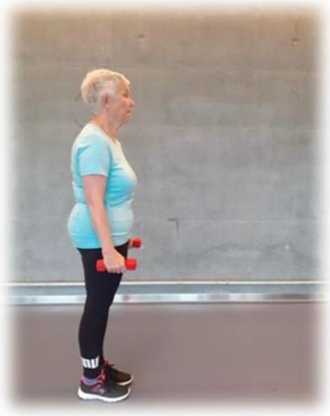

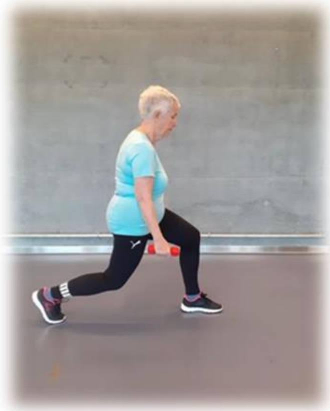

Exercises of the participant's choice

Option A, Fitness (20 minutes)

**Level 1**	Activity that improves your fitness (e.g. walking or cycling on an exercise bike).<br>**20 minutes at moderate intensity**
**Level 2**	Activity that improves your fitness (e.g. walking or cycling on an exercise bike).<br>**20 minutes: 1 minute fast and 2 minutes slow**
**Level 3**	Activity that improves your fitness (e.g. walking or cycling on an exercise bike).<br>**20 minutes: 1 minute fast and 1 minute slow**
**Level 4**	Activity that improves your fitness (e.g. walking or cycling on an exercise bike).<br>**20 minutes: 2 minutes fast and 1 minute slow**

Option B, Strength training (20 minutes)

Exercise 1B. Arm stretching

Level 1. Stand facing a wall. Place your hands against the wall and place your feet slightly back. Tense your buttocks and abdomen and keep your body in a straight line. It is important that you maintain your posture throughout the exercise. Now lean against the wall so that your arms form an angle of approximately 90 degrees and slowly push outward again.

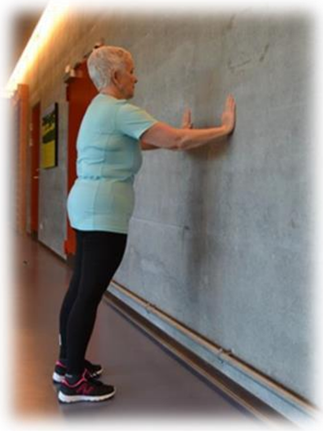

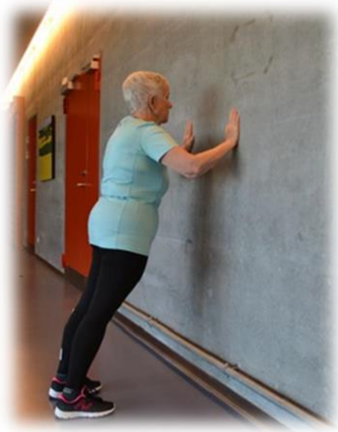

Level 2. Attach the resistance band approximately between hip and chest height to a wall bar or a door handle. Stand with your back to the wall and grab the resistance band with your arms bent at a 90-degree angle. Straighten your back and tense your body. Now press your arms straight forward. Return to the starting position in a controlled manner – avoid twisting your upper body or swaying your back. Only your arms should move.

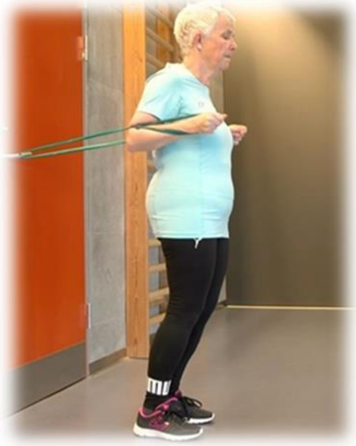

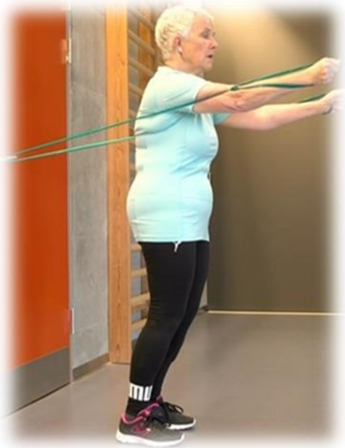

Level 3. As in level 2, but with a resistance band with greater resistance.

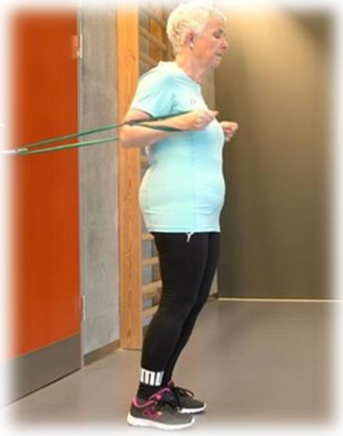

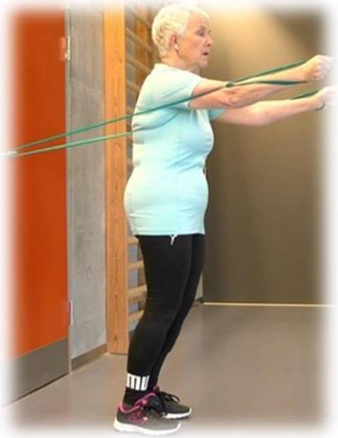

Level 4. Lie down on your knees on the floor and also place your hands on the floor. Place your hands just below your shoulders and slightly wider than shoulder width. Tense your entire body. Slowly lower your body towards the floor by bending your elbows. Slowly push yourself back up.

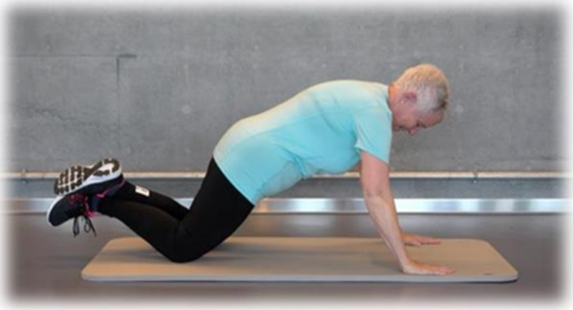

Level 5. As in level 4, but on tiptoe instead of the knees

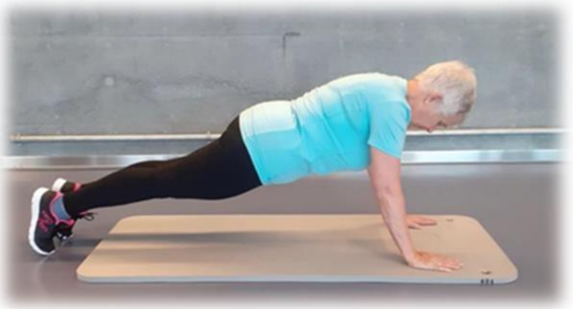

Exercise 2B. Pelvic lift

Level 1. Start in a seated position with your feet parallel and hip-width apart. Put equal weight on both legs and use the armrest of the chair to support your hands. Tense your bottom and push your hip upward. Then lower yourself in a controlled manner back to the starting position.

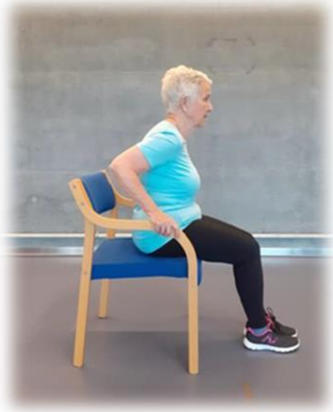
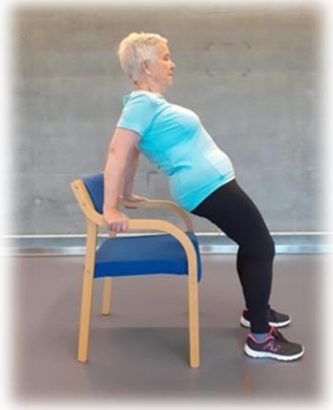

Level 2. Lie on your back with your legs bent and your arms out to the side. Tense your bottom, press your feet into the floor and lift your bottom off the floor until your body is fully stretched from chest to knees. Then lower yourself in a controlled manner back to the starting position.

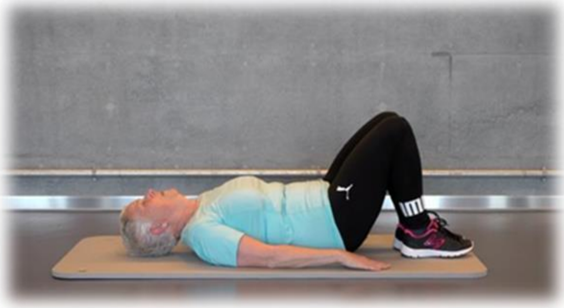
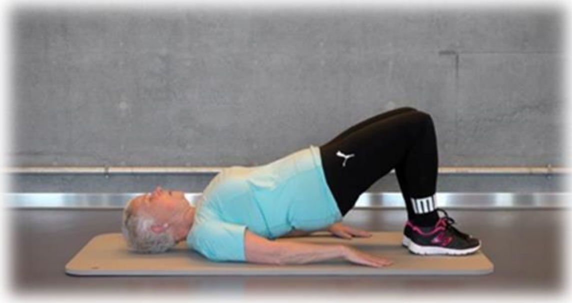

Level 3. As in level 2, but without arm support.

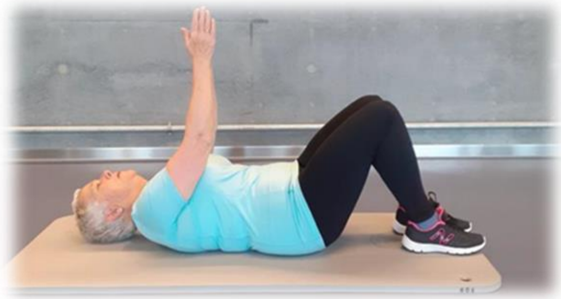

Level 4. As in level 2, but with a resistance band over the hip.

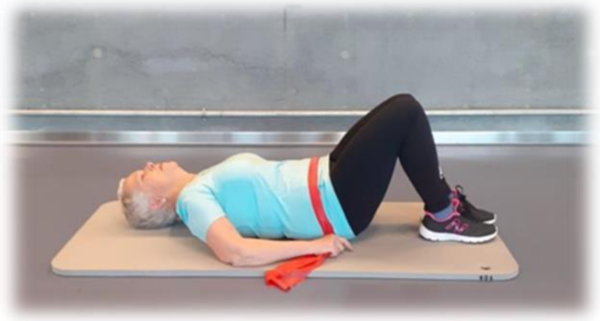

Level 5. As in level 2, but with one leg extended. Make sure to keep the pelvis straight. Repeat with the opposite leg extended.

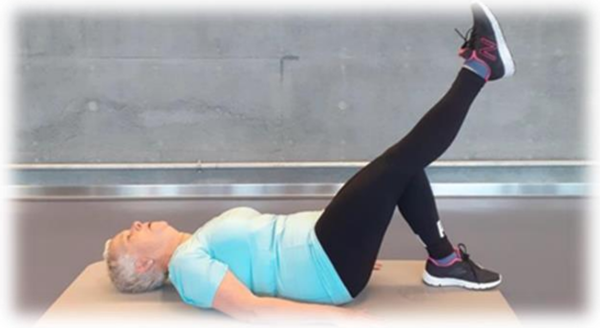

Exercise 3B. Abdominal exercise

Level 1. Attach the resistance band to a wall bar or a door handle at approximately chest height. Grab the other end of the band and take a few steps to the side. Put equal weight on both legs. Stand up straight and tense your body. Hold the end of the resistance band with both hands just below your chest and arms at a 90-degree angle. Lower your shoulders. Extend your hands forward until your entire arm is straight, then return to the starting position. Repeat with the opposite side.

Level 2. As in level 1, but take a step further out. Repeat with the opposite side.

Level 3. As in level 1, but with a resistance band with greater resistance. Repeat with the opposite side.

Level 4. As in level 2, but with a resistance band with greater resistance. Repeat with the opposite side.

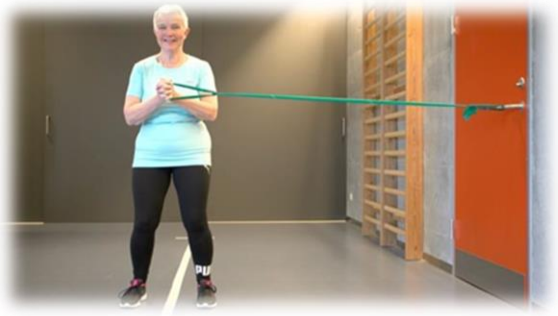

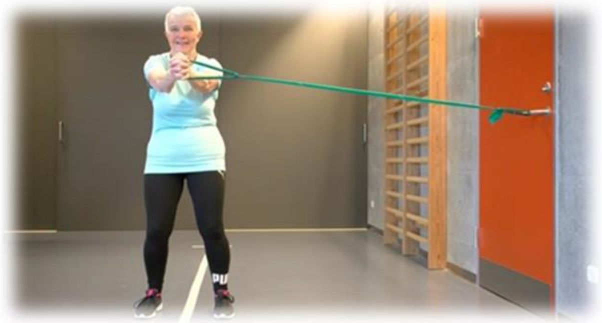

Alternative abdominal exercise
(Can replace abdominal exercise 3B)

Level 1. Lie on your back with your legs bent. Place a towel or resistance band behind your head to stabilise your neck. Tense your abdomen and raise your shoulders off the floor. Slowly lower back to the starting position.

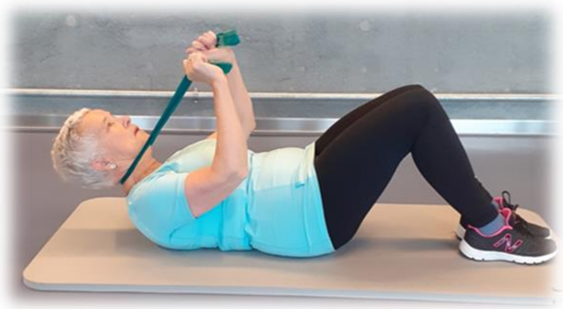

Level 2. As in level 1, but with the legs on a large training ball.

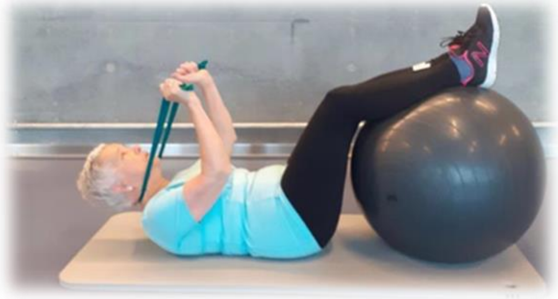

Level 3. As in level 2, but without a large training ball.

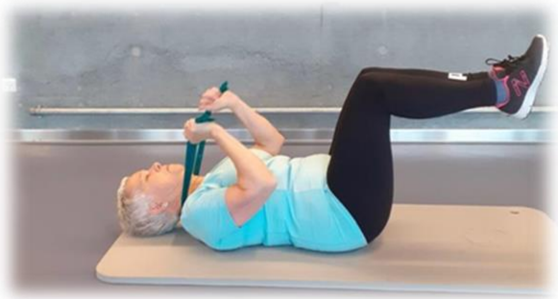

Level 4. Sit on a large exercise ball. ‘Walk’ out carefully until you are lying with your back on the ball. Tense your abdomen and lift your head and upper body so that only your lower back is in contact with the ball. Slowly lower back to the starting position. Ensure a smooth movement.

Option C, Functional exercises (20 minutes)

Exercise 1C. Stairway

Level 1. Step up and backwards down a low step bench (10cm). Stay focused on the appropriate positioning of your hip, knee and ankle joints. Switch legs and repeat the exercise.

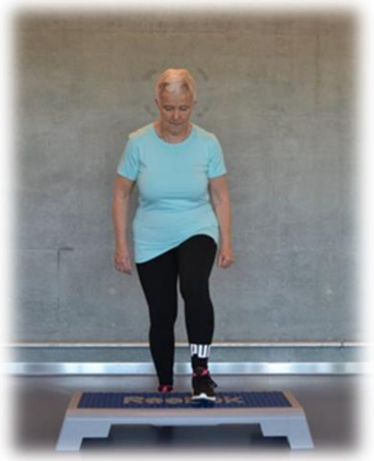
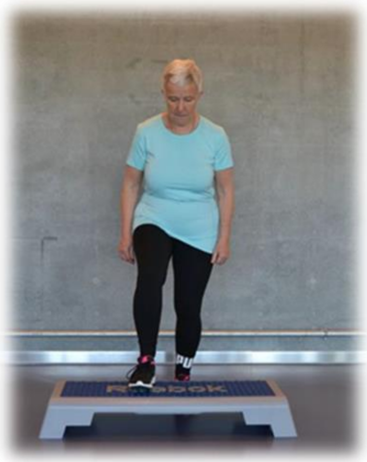

Level 2. As in level 1, but use a higher step bench (20cm). Switch legs and repeat the exercise.

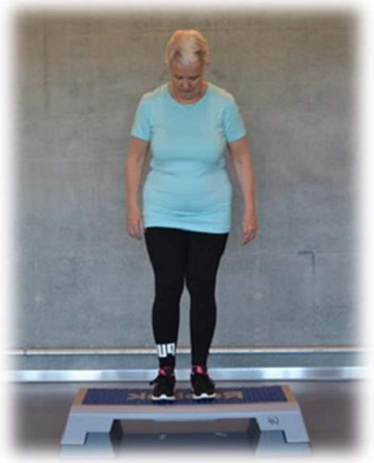
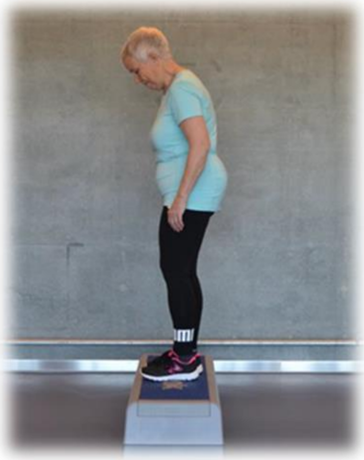
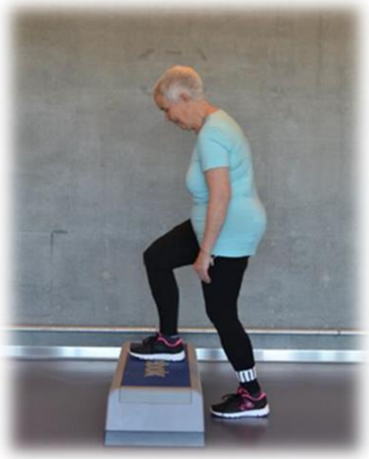

Level 3. As in level 2, but with a higher step bench (30cm). Switch legs and repeat the exercise.

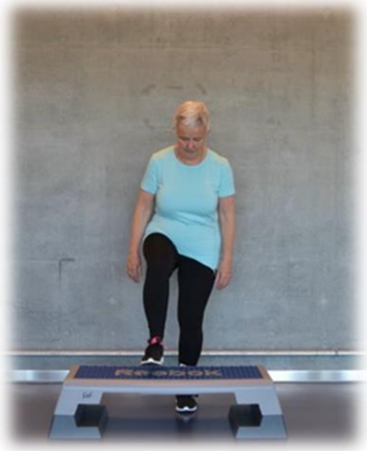
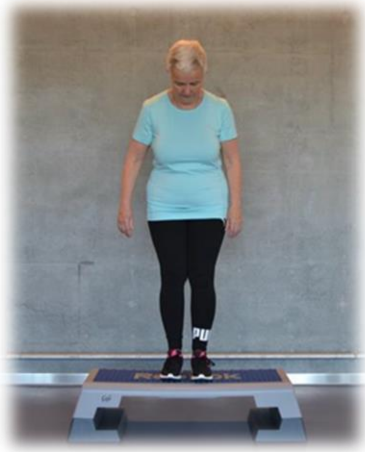
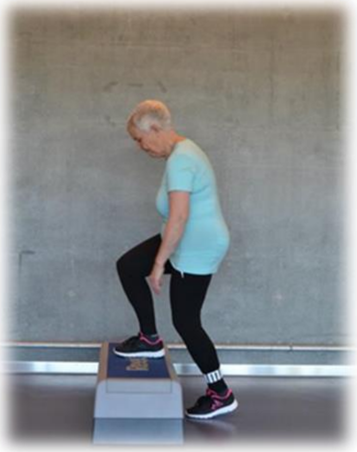

Level 4. As in level 3, but with a dumbbell in each hand. Switch legs and repeat the exercise.

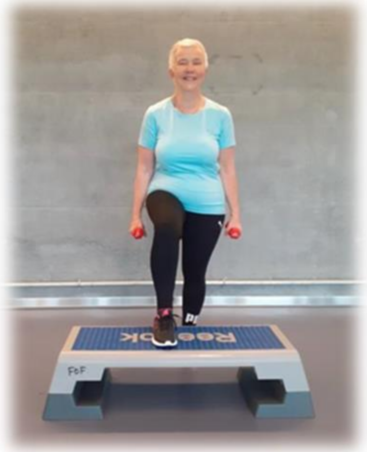
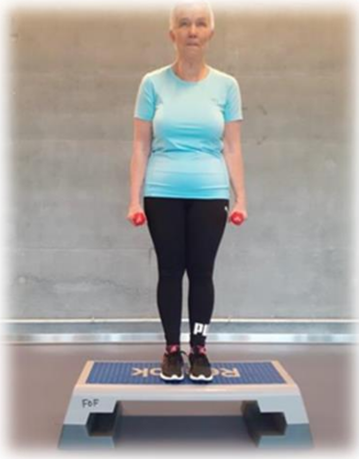

Exercise 2C. Grandchild lifting

Level 1. Take the exercise ball and lift it as high as you can. Keep your back straight and focus on the appropriate positioning of your hip, knee and ankle joints.

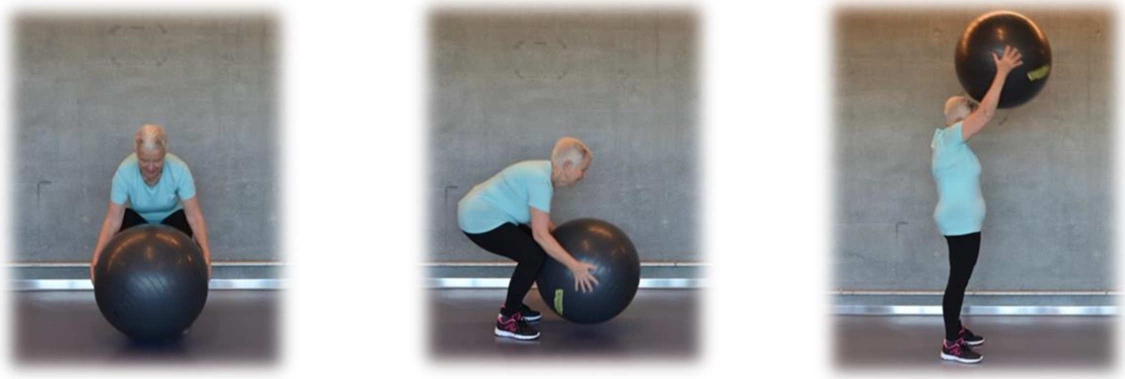

Level 2. Take a small kettlebell and lift it up to chest height. Keep your back straight and focus on the appropriate positioning of your hip, knee and ankle joints.

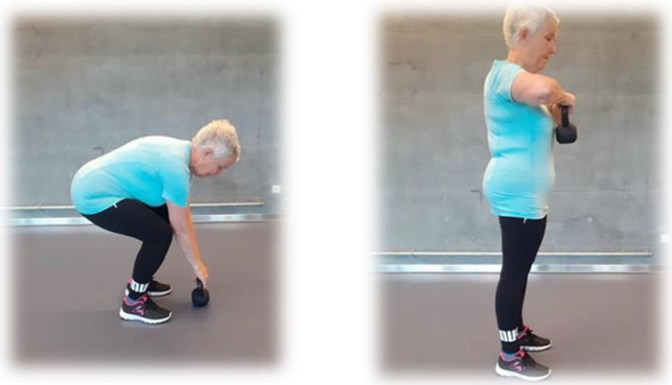

Level 3. As in level 2, but where the small kettlebell is lifted above the head.

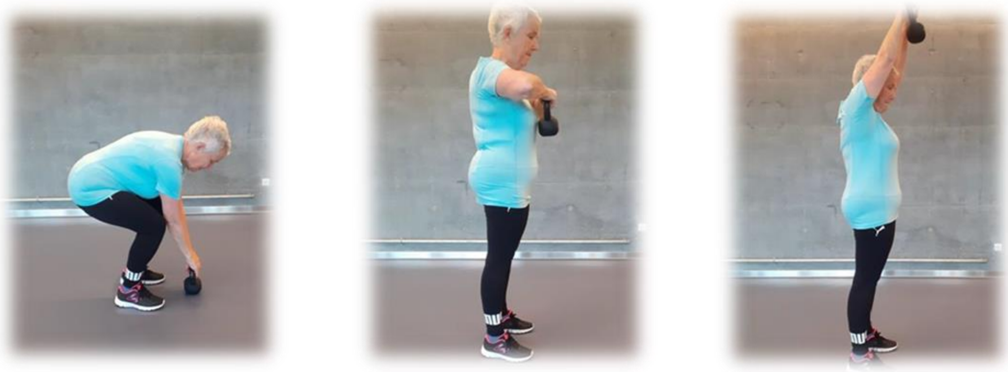

Level 4. As in level 3, but with a heavier kettlebell.

Exercise 3C. Carrying shopping bags

Level 1. Hold a dumbbell or kettlebell (1 or 2kg) in each hand. Stand with your feet slightly apart. Pull your shoulder blades back and straighten your back. Begin the movement by activating the muscles around your core. Now take a step forward and start walking with a weight in both hands. Look straight ahead. Keep your shoulders down and keep tensing tightening around your core. Divide the 'walk' into 5 metres at a time, so that, for example, 20 metres becomes 4 by 5 metres.

Level 2. As level 1, but with a heavier dumbbell or kettlebell (3 to 6kg) in each hand.

Level 3. As in level 2, but with a heavier dumbbell or kettlebell (7 to 10kg) in each hand.

Level 4. As in level 3, but with a heavier dumbbell or kettlebell (>10kg) in each hand.

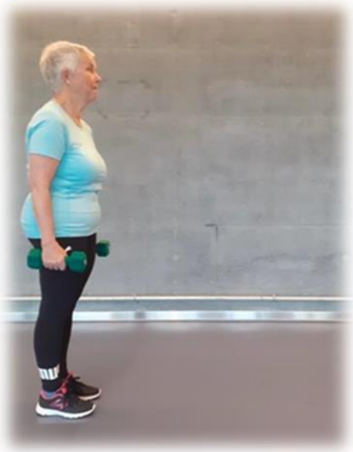
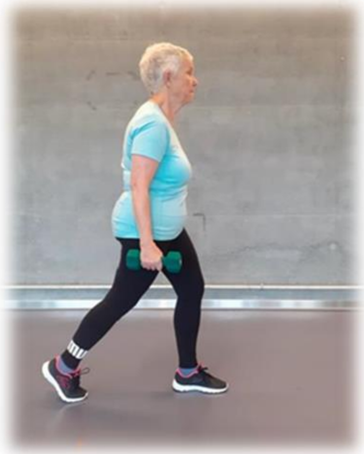
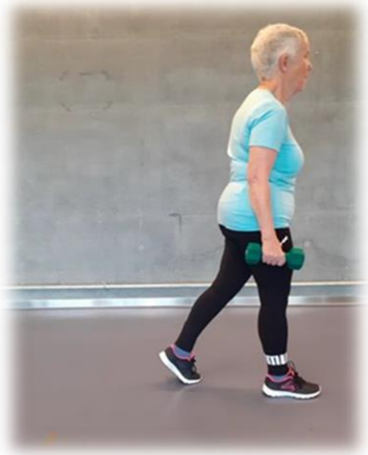

Cool-down (7 minutes)

Exercise 1. Child's position

Stand on all fours with your feet together and hands shoulder-width apart. Place your bottom onto your heels while your hands and arms rest in front of you. Take a deep breath and lean your upper body forward so that your chest rests on your thighs and your forehead rests on the mat. If necessary, place your hands under your forehead for support. As a variation, you can put your arms down by your side. Hold the stretch for 25 seconds and then return to the starting position. Repeat twice.

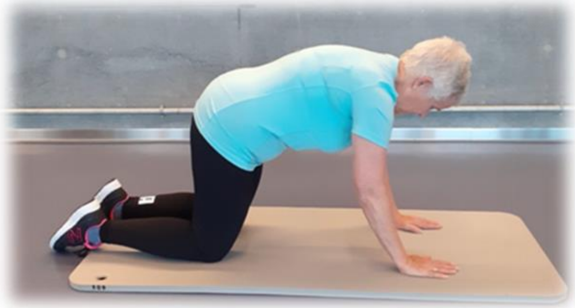

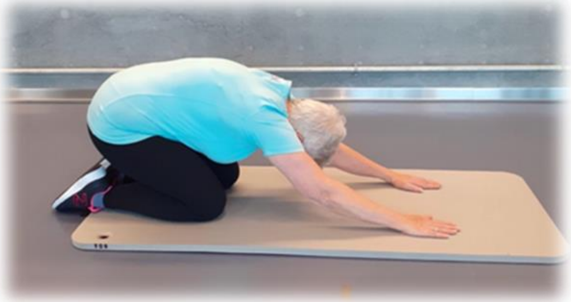

Exercise 2. Standing forward bend

Stand with your feet slightly apart and your hands folded behind your back. Exhale and bend forward at the hips and down towards the feet, while keeping your back straight. Hold the stretch for 25 seconds and then return to the starting position. Repeat twice.

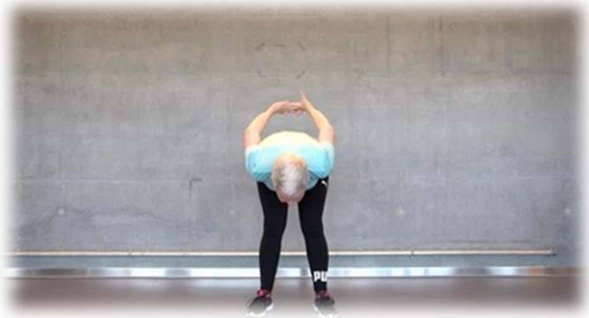

Exercise 3. Stretching upper body

Stand with your feet slightly apart and your hands folded in front of your abdomen. Raise your arms and press your hands up towards the ceiling. Hold the stretch for 25 seconds and then return to the starting position. Repeat twice.

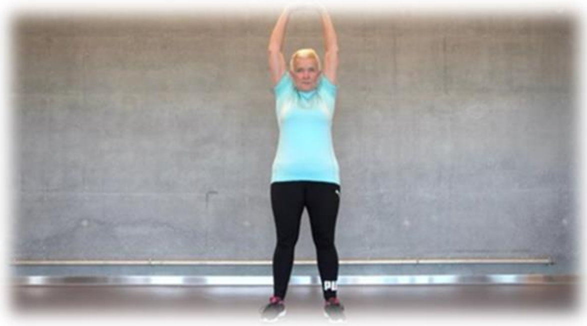

Alternative cool-down exercises

(Can replace the above cool-down exercises)

**Arm swing**<br><br>Bend your knees slightly and let your arms swing along, keeping your knees slightly bent throughout the execution.
**Shoulder roll up, back, down w/ scapula activation**<br><br>Raise your shoulders and move them backwards by bringing your shoulder blades together. Hold the position for a moment before actively lowering your shoulders straight down and letting them drop back to the starting position.
**Contraction of shoulder blades**<br><br>Stand upright, arms hanging down at your sides. Pull your shoulder blades together and slightly down.

Contraindications to exercise

Condition	Contraindications to exercise	Suggestions for physios
**Chronic heart failure and ischemic heart disease**	• Patients with level 4 NYHA<br>• Acute myocardial infarction within the last 3 months<br>• Present unstable angina, e.g. pain in the chest at rest or pain that does not respond to specific medication<br>• Pain in the chest before exercise<br>• Changes in known or new cardiac arrhythmias<br>• Present inflammation (pericarditis, myocarditis, endocarditis)<br>• Present symptomatic aortic stenosis<br>• Present dyspnoea at rest<br>• Within 10 days after a period of fever or present fever<br>• Increase in bodyweight more than 2 kg in the last 2 days (applies only to ischemic heart disease)<br>• Acute systemic infection	• If a patient experiences an increase in frequency and severity of angina/chest pain, stop the session and seek a doctor
**Depression**	• Currently meets DSM criteria for major depression or two or more episodes of major depression in the past 10 years, moderate dementia, current usage of daily anti-anxiety medication, currently meets DSM criteria for substance abuse or dependency, requires daily antipsychotic medication<br>• +3<br><br>• Current mental illness requiring psychiatric hospitalization, institutionalization, or intensive outpatient management (e.g. patients with severe or suicidal depression, acute psychosis or psychotic decompensation, severe agitation from dementia, severe substance abuse, etc.), severe dementia	• Make sure to motivate him/her constantly
**Hypertension**	• Resting systolic blood pressure of >200 mmHG or diastolic blood pressure of >115 mmHG	• Remind the patients to regularly assess their blood pressure and contact the GP if the blood pressure goes above the recommended thresholds. This is something that is part of standard care (e.g. contact the GP if the blood pressure fluctuates).
**Type 2 diabetes**	• No serious complications are known compared to people not exercising	• Foot ulcer (make sure the patient wears comfortable shoes)

		• People taking insulin or insulin secretagogues have an increased risk of hypoglycaemia (low blood sugar) with physical activity therefore keep around some sweets if this may occur<br>• People with peripheral neuropathy should be closely monitored for complications but they can exercise. Monitor numbness, loss of sensation, and sometimes pain in the feet, legs, or hands.
**COPD**	• Exceptional loss of bodyweight (10% in past ½ year or 5% in past month)<br>• Resting saturation <89%<br>• Non-adherence to COPD treatment	• The common minor adverse events reported in COPD patients are increased shortness of breath, muscle cramp and soreness especially at the beginning of the intervention. Monitor and adjust the dose of the exercise intensity accordingly. Also, COPD patients should know what to do (based on their individual recommendations from the GP what to do in case this may occur).
**Osteoarthritis**	• No major contraindications	• Pain is the most common minor adverse event. Use the VAS scale as described in the manual to monitor pain during the session.
**Osteoporosis**	• No major contraindications	• Like OA. In addition, persons with kyphosis may find it difficult/discomfort to bend forward their back, so use exercises that avoid discomfort.
**Type 1 diabetes**	• People should not start physical activity while ketones are abnormal, and the underlying cause should be found. Ketones may rise in endurance exercise, without a significant rise in serum glucose. After vigorous physical activity, hyperglycaemia may occur, so caution regarding overcorrection (potentially leading to hypoglycaemia) is required. Those with advanced neuropathy, autonomic dysfunction, end-stage renal failure or severe proliferative/non-proliferative retinopathy may require specialist advice.	• There may be increased risk of hypoglycaemia for 24 hours after exercise including risk of nocturnal hypoglycaemia, especially with afternoon activity. Keep carbohydrates close so that they can get some in case this happens during the session
**Parkinson's disease**	• Hoehn & Yahr scale ≥ 4	
**Chronic kidney disease**	• Electrolyte abnormalities – especially hypo/hyperkalaemia<br>• Recent changes to the ECG, especially symptomatic tachyarrhythmias or brady-arrhythmias<br>• Excess inter-dialytic weight gain >4 kg since last dialysis or exercise session	

	• Unstable on dialysis treatment and changing (titrating) medication regime<br>• Pulmonary congestion<br>• Peripheral oedema	
**Organ transplantation (heart, kidney, liver, lung)**	• If stable they can participate, otherwise, the contraindications are the same as for patients with heart failure	
**Multiple sclerosis**	• It seems there are no specific contraindications for multiple sclerosis	• Fatigue is often reported by people with multiple sclerosis.
**Bone cancer And cancer in general (including people with metastases)**		
**COVID-19**	• Cannot exercise if a patient has COVID-19 until 10 days after testing positive to a PCR test	• Return to exercise or sporting activity should only occur after an asymptomatic period of at least seven days<br>• Week 1 and 2 after returning to training the intensity should be low (RPE 11-12 on the BORG-20 scale)<br>• Week 3. RPE of 12-14 (moderate intensity, not out of breath and could hold a conversation)<br>• Week 4. Would involve more complex movement that challenges coordination, strength, and balance, such as running but with changes in direction, side-steps, shuffles, and circuits of body weight exercises, but again without it feeling hard. After completing phase 4, people should then feel able to return to their baseline (pre-covid) level of activity or more<br>• Week 5. MOBILIZE training as before COVID-19. However, people should stay at the phase they feel comfortable with for as long as necessary. They should monitor for any inability to feel recovered at 1 hour after exercise and on the day after, abnormal breathlessness, abnormal heart rate, excessive fatigue or lethargy, and markers of mental ill health. If these occur, or the person fails to progress as expected, they should step back to an earlier phase of activity and seek medical advice when unsure.

MOBILIZE THEMATIC MODULES MANUAL

”Most people who live with chronic disease suffer from multimorbidity. Unfortunately, the healthcare system and most research focus on one medical condition at a time. Treating one condition at a time is inconvenient, inefficient and unsatisfactory for the person with the chronic conditions as well as his or her healthcare provider. Research on effective treatment of multimorbidity is lacking.

The MOBILIZE project is going to change that and offers patients with multiple chronic conditions an individualized program tailored to their conditions, circumstances, and needs, thereby improving their quality of life and physical function and helping to prevent the development of additional chronic diseases.

Because exercise and self-management are at the core of the treatment and because physiotherapists have broad knowledge and competencies across diseases, they are well-suited to deliver this treatment and support the patient in better managing their symptoms and leading a more active life with higher quality of life and physical function.”

- Søren T. Skou, Professor and Head of Research

How to use the manual

The purpose of the self-management program is to help participants acquire skills that enable them to better manage their chronic diseases. Research indicates that it is in the patients' best interest to build competencies for managing their own illness. Additionally, from a health policy perspective, it is important that individuals with chronic diseases are more involved in their treatment than before.

The self-management program consists of 24 PowerPoint presentations – one for each thematic module. Since participants are enrolled on an ongoing basis, all presentations are designed to be delivered to participants regardless of when they enter the program. The order in which the thematic modules should be presented is structured to ensure variation in themes, teaching methods, and activities from session to session. Thus, the intervention site starts in the given order and then runs the 24 thematic modules in a loop, with participants joining at different times. There will be some repetition in some of the presentations, partly to enhance understanding and partly so that the module can be presented independently. An overview of the sequence is available in SharePoint: Documents -> Patient Education -> Various Documents.

The manual is exclusively for use by intervention therapists in connection with the self-management component of MOBILIZE. The manual is a clinical working tool and must not be distributed to others.

Each PowerPoint presentation is structured around the same template with an introductory slide presenting the day's topic, and notes indicating what the therapist should bring to the session. This is followed by 5-6 slides covering the day's theme. Each slide has accompanying notes to support the presentation. The final slide always consists of brief 'take-home messages' for the participants.

In the accompanying notes for each presentation, the following applies:

- **Bold** text indicates instructions for the physiotherapist
- Non-bold text is what should be conveyed with each slide
- Point x) in the notes corresponds to a bullet point (•) on the slide
- Paragraph x) in the notes corresponds to a paragraph on the slide
- *Italicized* text indicates references and citations

The self-management program also includes a patient handbook that compiles key points and links for each of the 24 thematic modules. The therapist will hand out a printed copy of the patient handbook to participants during the individual introduction to the program. Additionally, an

electronic version can be emailed to participants, making it easier for them to access links, videos, etc.

Tips and tricks for delivering the MOBILIZE self-management program

The role of the facilitator

- Enable participants to take greater control of their own illness.
- Help participants assess their own needs and make independent decisions.
- Build participants' confidence in their own abilities.
- Encourage each participant to contribute to the best of their ability, as everyone has valuable knowledge to share.
- Help build trust and respect among participants to promote dialogue and learning that benefits the entire group.
- Create a pleasant and friendly atmosphere where there is also room for humor.
- Meet participants where they are.
- Avoid persuasion and arguments.
- Provide support, recognition, and positive reinforcement.

Characteristics of a good facilitator

A good instructor is patient, listens, and understands the needs of their participants. They provide feedback and meaningful explanations. They interact with participants and allow them to have a voice. They are careful not to pressure, criticize, or make participants feel guilty.

Verbal skills

- Speak slowly and clearly: Use simple phrases to explain complex topics and use personal examples to facilitate understanding.
- Encourage active participation: Ask questions that prompt participants to speak, such as: "What do you think about…", "Why…", "How…", "What if…", etc. If a participant answers with just "Yes" or "No," ask them to elaborate.
- Paraphrase: Occasionally, explain participants' thoughts and feelings in your own words. Remember to check with participants to ensure you have understood them correctly.
- Share your stories: Share a bit about yourself (appropriately) to build a level of trust between the group and you. Participants are more likely to share their stories if you share yours.
- Provide examples: Use examples to help participants visualize and relate to the topic.

- Regularly check for understanding and interest: Ask questions like "Does this make sense to you?" or "Do you have any questions or comments?" Help quieter participants to speak up and point out if someone is talking too much.

Nonverbal skills

- Look over the entire group: Avoid focusing too much on a single participant.
- Stand up when you speak, especially at the beginning of a session: Show that you are relaxed but in control by smiling and keeping your hands still.
- Move around the room without being disruptive.

How to handle difficult questions

Participants sometimes ask complicated questions that can be difficult to answer. Facilitators often feel they should have all the answers. It's OK to say you don't know the answer! Instead, say that you will look into it and get back to them.

It's important that you familiarize yourself with the program and the materials provided by the MOBILIZE team. Read the material before you teach, and make sure you understand the meaning and language used. Consider which topics might need further explanation for the participants

How to handle conflicts

It can happen that some participants have strong, opposing opinions on a topic. If there is a bad atmosphere among some participants, this can affect how the group functions as a whole. The facilitator should be aware of potential disagreements and tensions and encourage participants to stay focused on common goals and interests.

How to keep track of time

It can sometimes be difficult to keep track of time when a discussion becomes very engaging or when participants are highly involved and delve into details. We have indicated the duration in minutes for the various activities to make it easier to manage time. If time runs short, tasks can be simplified (e.g., remove certain steps in an activity or very detailed examples) or the number of discussions can be reduced.

How to involve participants

Be careful not to let a single participant (who feels they have all the right answers) dominate the conversation. A good facilitator ensures that all participants have a chance to speak.

Involving relatives

Research shows that involving relatives has benefits for the patient's physical, mental, and emotional well-being. MOBILIZE recommends that relatives be invited to participate in the thematic modules on:

- Carers/relatives
- Goal setting
- After MOBILIZE

…as this can help them better support the patient moving forward.

Below is the text for the various thematic modules. The text is also available as notes for each slide in the PowerPoint presentations.

Tematic modules

Self-management 7

Physical activity 15

Mindfulness 27

Your story – patient narratives 34

General physical symptoms 40

Healthy eating habits 49

Involving carers/relatives 60

Stress reactions 68

Breathing as a tool 76

Physical training 87

Mindfulness: body scan 96

General mental health symptoms 101

Coping strategies 110

Goal setting 118

Breaking down barriers to physical activity 128

Pain mechanisms 136

Communication 146

Physical training and chronic illness 152

Mindful eating 160

Breathing and shortness of breath 169

Self-care 179

Self-monitoring 187

Mindfulness techniques 196

After MOBILIZE 205

Self-management

Slide 1

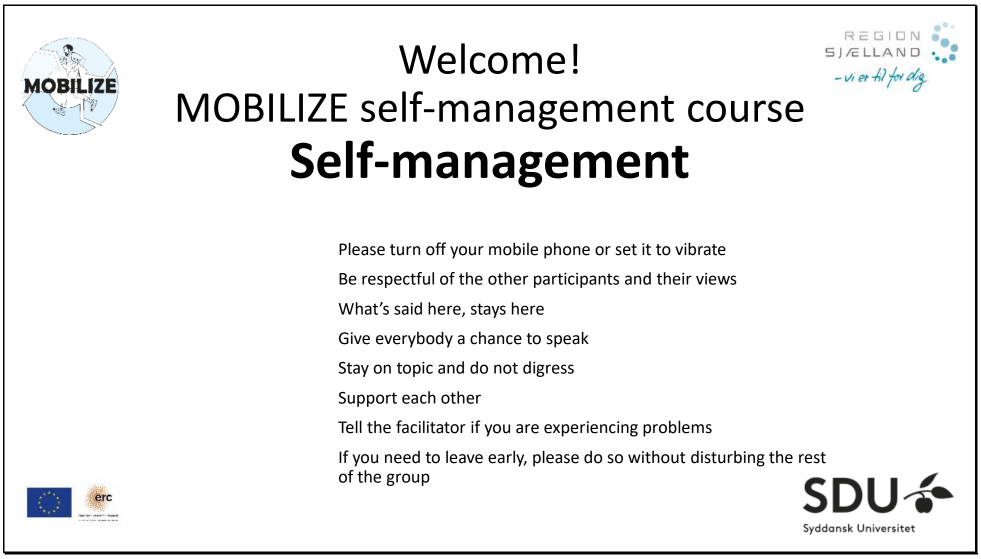

Welcome to the thematic module on self-management.

Self-management is defined as 'the tasks that individuals must undertake to live well with one or more chronic conditions'.

Self-management is what the patient education part of MOBILIZE is all about.

Today, we will look at your role in this.

Slide 2

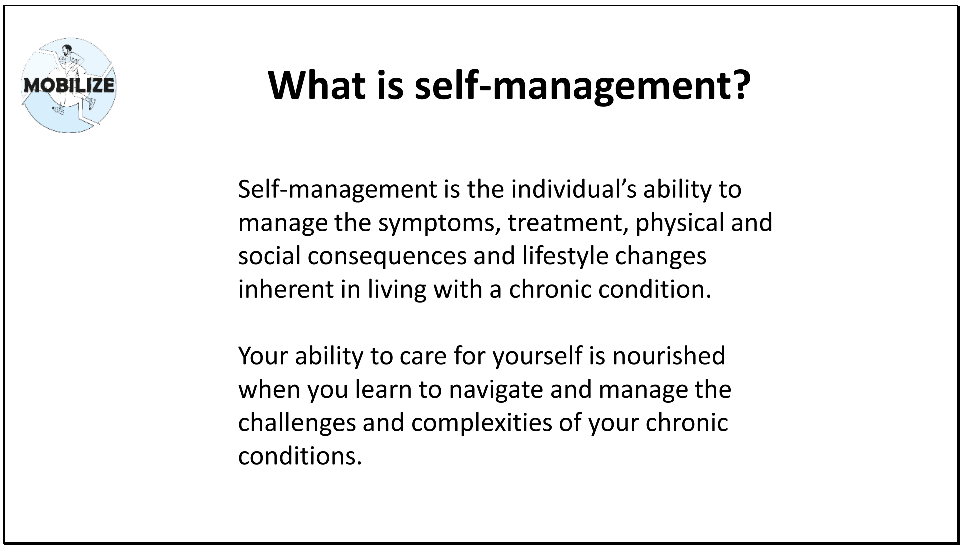

Paragraph 1) Self-management involves finding out more about your condition and learning new skills to help you manage your health.

Paragraph 2) Often in cooperation with healthcare professionals and family so you can make the right choice for you.

Slide 3

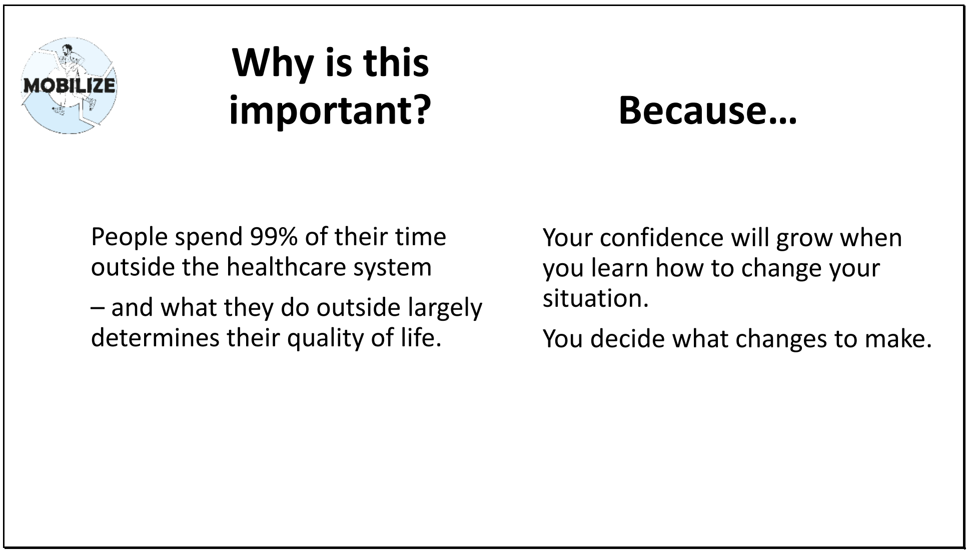

End of slide:
The more knowledge and understanding you have of your chronic conditions, the easier it is for you to influence and control the situation and your daily life.

Slide 4

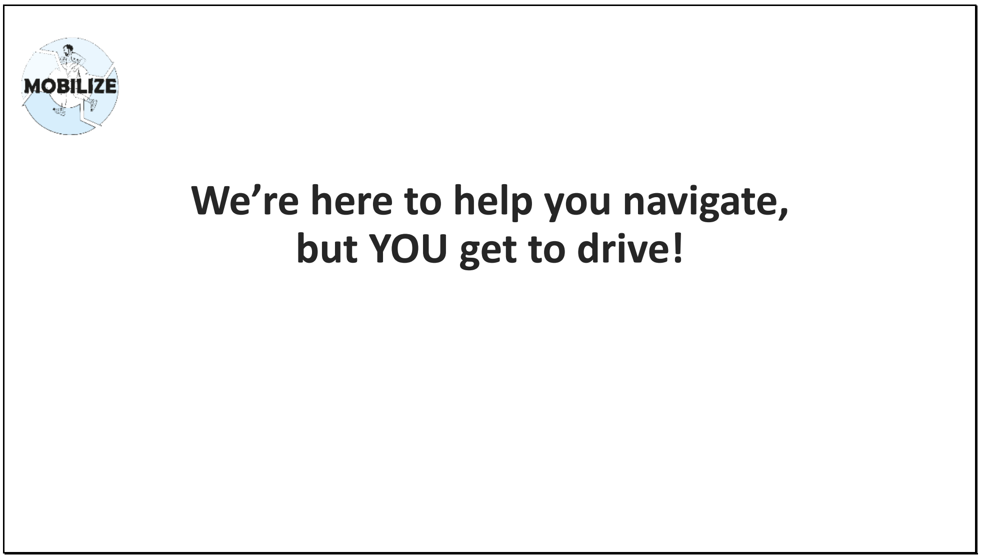

Self-management is NOT:
To be left alone
To do everything yourself
To stop 'bothering' doctors and healthcare professionals
To suffer in silence
To save money or costs

Slide 5

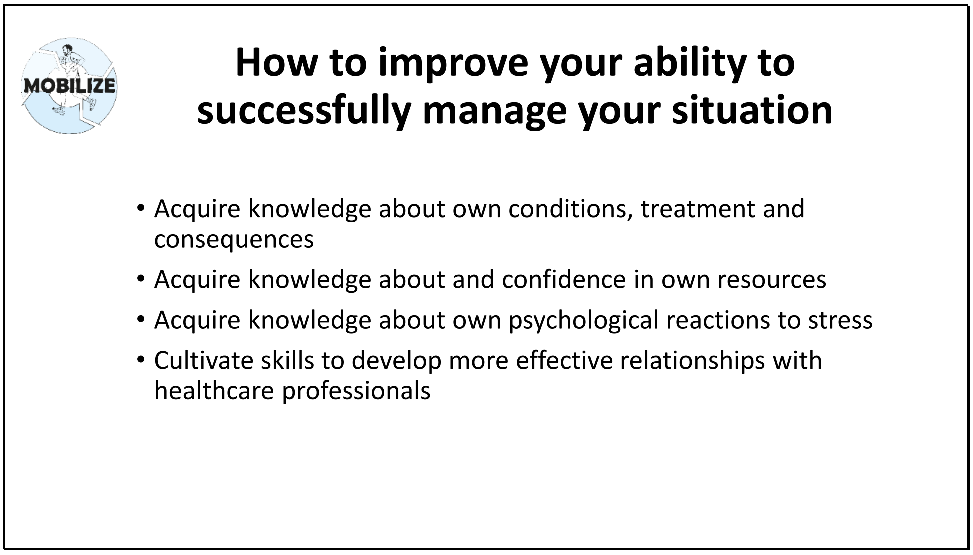

Before clicking further to the points, ask the participants: What do you need in order to improve your ability to successfully manage your situation? Is it more knowledge? More confidence when communicating with healthcare professionals? More time with the doctors? Or something completely different? **Discuss (10 minutes).**

Introduction to points: The Danish Health Authority has described a number of focus areas if you want to improve your ability to successfully manage your situation.
Point 2) Example: Know how to monitor and register blood sugar measurements, when to act on the basis of the measurements and feel confident that you can do it yourself.
Point 3) By identifying your response patterns, both the effective and ineffective ones, so that you can ask for help if you get stuck in ineffective patterns.
Point 4) By strengthening communication and confidence when discussing health matters with healthcare professionals.

End of slide:
It is possible to improve your ability to successfully manage your situation through close cooperation with relevant healthcare professionals, your GP and patient education programmes like MOBILIZE.

Good self-management skills will improve your ability to gain or regain control of your health situation.

Slide 6

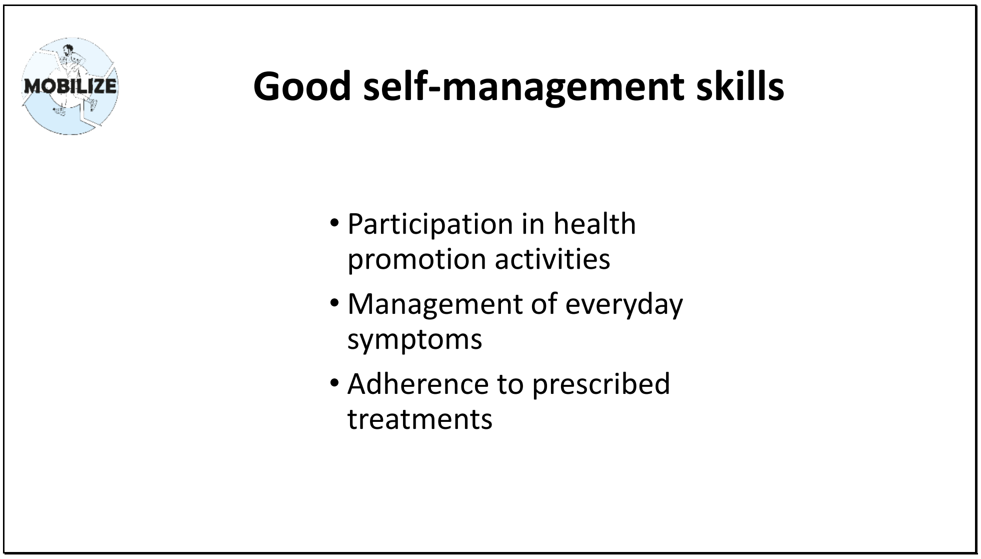

Point 1) Health promotion activities may include:
- Participation in patient education programmes like MOBILIZE
- Different kinds of exercise and physical activities such as walking and biking or exercising in a group or the local gym.

Point 2) You need knowledge about how to manage both physical and psychological symptoms related to chronic health conditions such as pain, breathing difficulties, fatigue, emotional problems etc.

Point 3) Examples: a drug or diet regiment, blood sugar monitoring, exercise or smoking cessation.

Slide 7

Benefits of good self-management skills

You will be **well informed** about your current situation.

You will **take control** of your situation (instead of being controlled by it):

- You will be able to **develop new skills** allowing you to manage your chronic conditions and how they affect your life and family etc.
- You will be **better equipped** to handle life's challenges
- You will feel that you get the right **support** and are more **aware** of the help available to you

Paragraph 1) The better informed you are about your situation, the easier it will be to ask the 'right' questions and seek more information.

Paragraph 2) Knowledge means more control over things. More control will lead to: **Points 3-5**

Slide 8

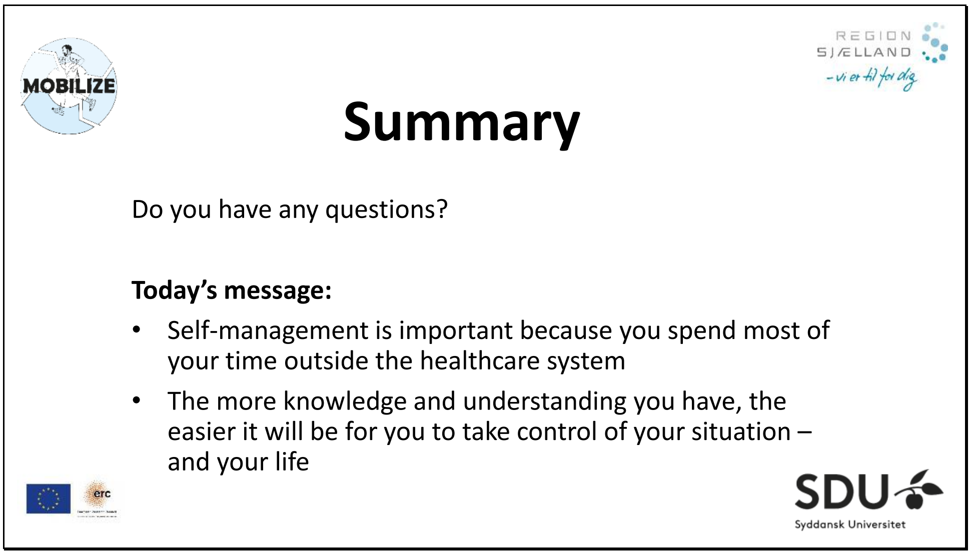

Answer any questions.

Point 1) What you do <u>outside</u> the healthcare system largely determines your quality of life.

Suggestions for home activities: At home, reflect and make notes on how to nourish YOUR ability to care for yourself. For more information, please consult your patient handbook.

Physical activity

Slide 1

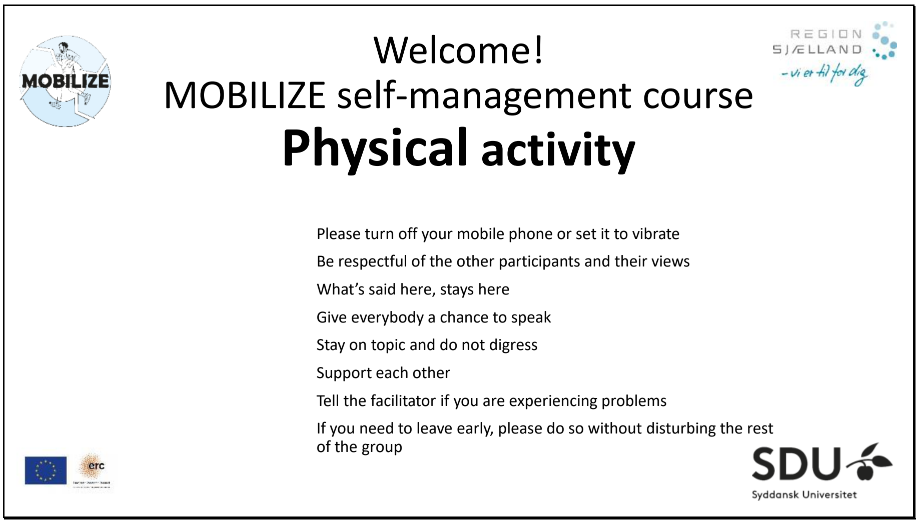

You will need a whiteboard/flipover, pencils and paper for this module.

Welcome to the thematic module on physical activity.

Today, we are <u>not</u> going to talk about the type of physical activity that is planned, structured and repetitive in nature – you will or have already talked about that in one of the other modules. Instead, we are going to focus on physical activity in a broader perspective. You will learn why it is important to stay physically active and how to sneak more physical activity into your daily routine.

Slide 2

Physical activity

WHO defines physical activity as any bodily movement produced by skeletal muscles that requires energy expenditure.

Physical activity refers to all movement including during leisure time, for transport to get to and from places, or as part of a person’s work.

To be physically active, you do not necessarily need to put on a sweat suit.

Slide 3

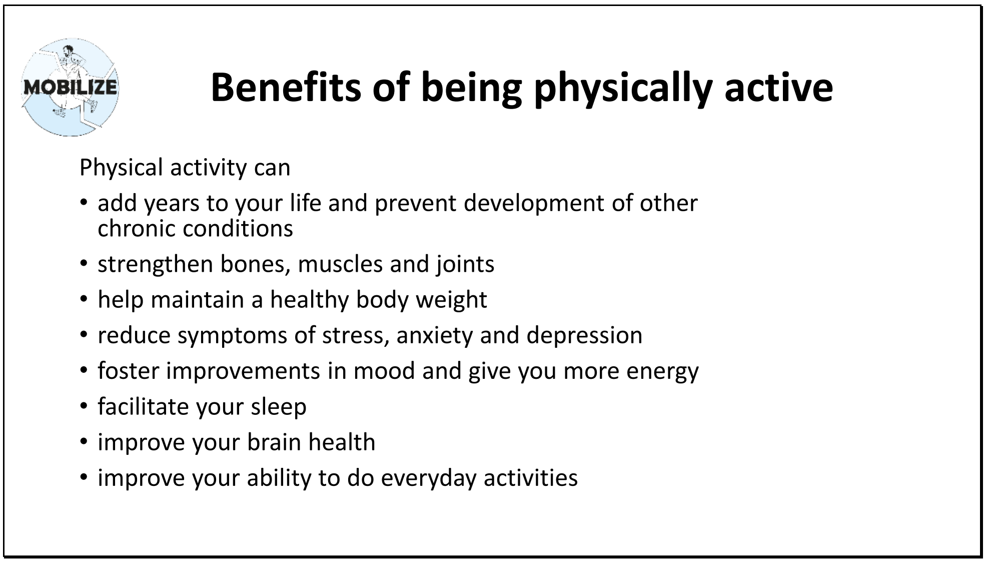

End of slide: Regular physical activity can prevent the development of lifestyle disease such as type 2 diabetes and cardiovascular diseases.

And regular physical activity is not only preventive but can also be therapeutic for people with chronic conditions – and with a low risk of side effects (compared to conventional medication)!

Slide 4

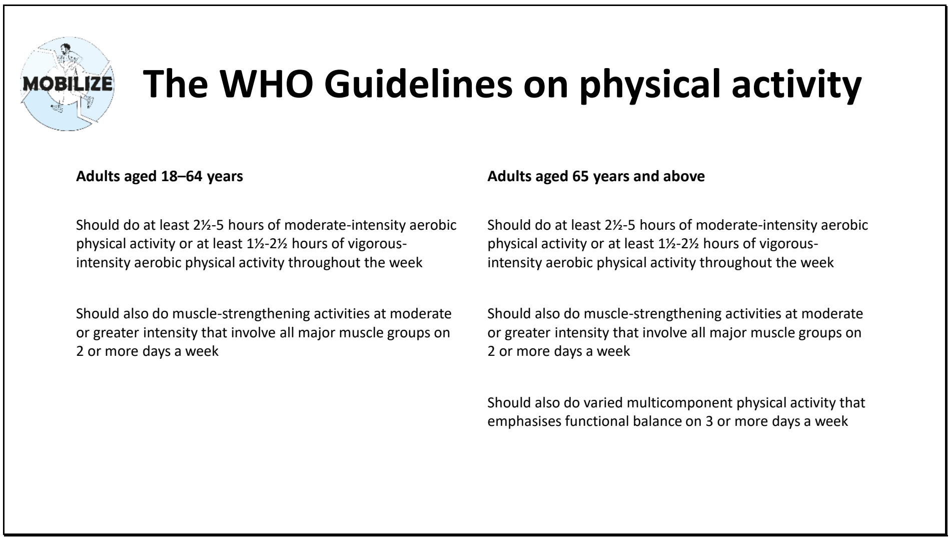

Introduction to slide:

For the first time in 10 years, WHO has updated their evidence-based public health recommendations for children and adults.

Now, there is evidence that sedentary behaviour has detrimental effects on your health. People of all ages should limit the amount of time spent being sedentary.

To help reduce the detrimental effects of high levels of sedentary behaviour on health, all adults and older adults should aim to do more than the recommended levels of moderate- to vigorous-intensity physical activity.

Some physical activity is better than doing none, and everyday activities, such as walking, cycling, cleaning and gardening etc., provide significant benefits for health. But you must always adjust your activities to your energy level and respect your limits. Start slow and then you gradually add to your routine.

As you can see, balance exercises are recommended for adults aged 65 and above. But note: Younger people can also benefit from such exercises.

Slide 5

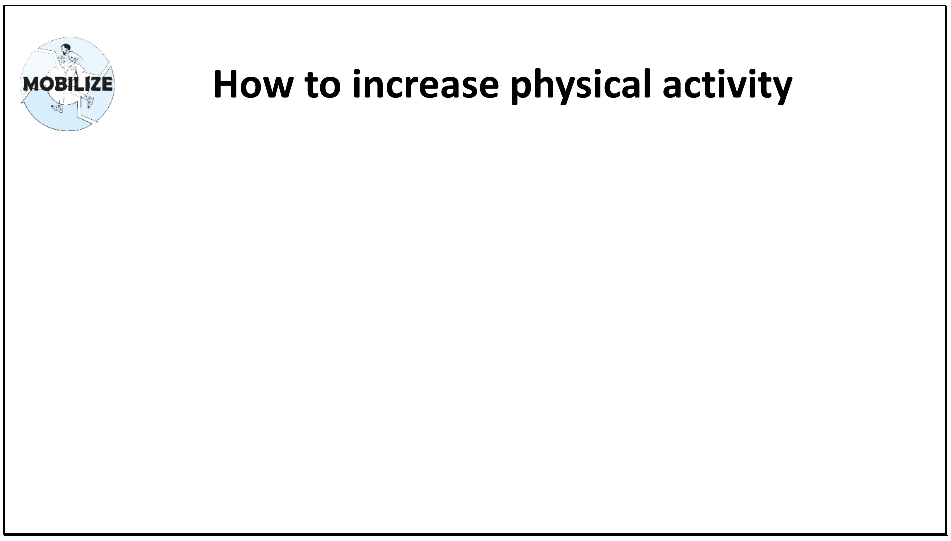

Ask the participants to share their ideas on how to incorporate physical activity into their daily routine. Write the ideas on a whiteboard/flipover. (Duration 10 minutes)

Please be aware that what is considered moderate activity by one participant may be considered vigorous by other participants.

At home:

•Take the stairs instead of the lift

•Walk around when you talk on the phone

•Find an active hobby (gardening, bowling)

•Plan a family activity after dinner (go for a walk)

•Dance while you listen to music

•Play with your (grand)children or pets

•*Make housework even more active*

•*Complete 5-10 chair stands before you sit down to watch television*

At work:

•*Go for a walk during your coffee break or lunch*

•*Park your car further away from the entrance*

•*Walk down the hall to speak with a colleague instead of sending an e-mail*

•*Use your adjustable standing desk to switch between sitting and standing*

•*Use a gym ball as an office chair*

•*Hold walk and talk meetings*

•*Always take the stairs when possible*

Exercise: Ask the participants to sit in the middle of the chair (away from the table). Tell the participants that something is better than nothing and introduce them to a 30-second chair stand exercise where they rise to a full standing position, then sit back down again. Start the exercise and begin timing.

This is an exercise that you can easily do at home before you sit down to watch television.

Slide 6

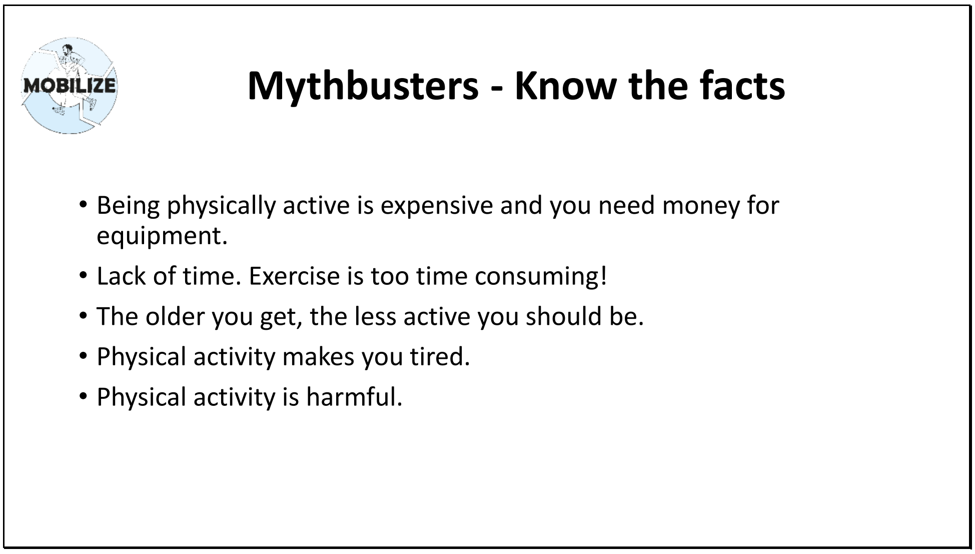

Point 1) Physical activity can be performed virtually everywhere, and you do not need expensive equipment. Walking is probably the most popular and most recommended type of physical activity – and it is completely free!

Point 2) Take a few days and monitor how you spend your time. Then identify 20 or 30-minute time slots that you could use for physical activity. Small things like taking the stairs, riding the bike to work or getting off the bus one stop earlier and walking the rest of the way can all add up and be incorporated into your daily routine.

Point 3) Most people become less physically active as they get older, but it is important to stay active your whole life – especially as you get older. Regular physical activity increases your ability to perform daily activities and stay independent longer.

Point 4) You may feel a bit tired when you exercise, but afterwards you will normally feel energised. Regular exercise boosts energy and will help you manage your daily tasks.

Point 5) No! Physical activity is safe. The human body is designed for physical activity and movement – even with wear and tear. You will learn more about that in the module on exercise and chronic conditions.

Slide 7

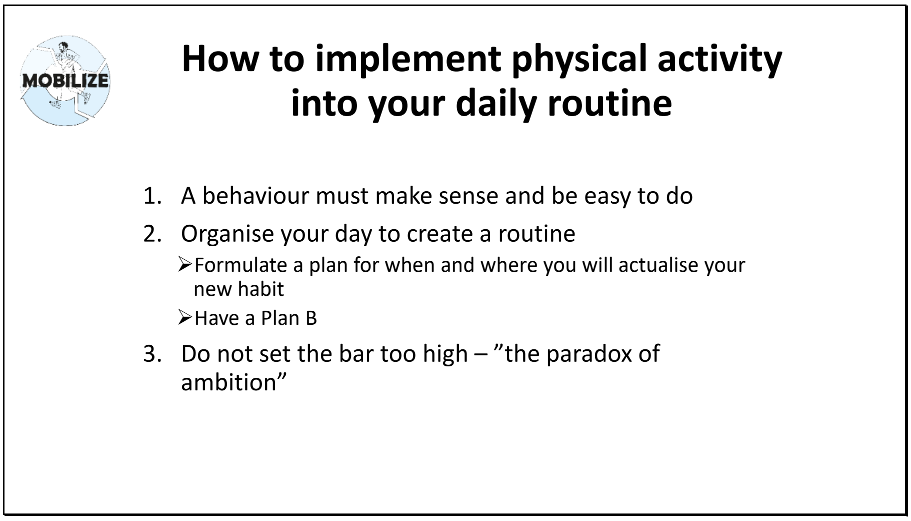

Point 1) The likelihood of you achieving your goal – i.e. carry out a behaviour, depends on how motivated you are and how easy it is for you to do. In addition, you need a trigger to activate the behaviour (a specific time, a certain situation).

Point 2) Brushing your teeth is a routine that we all do. You do it every day at roughly the same time. If that was not the case, you would probably forget to do it.

Point 2a) How to plan a behaviour management plan:

Behaviour*: Starting tomorrow, I will do 10 squats and 10 toe lifts by the kitchen table every time I take my medication.*

Or:

Behaviour: *Starting tomorrow, from Monday to Friday, I will ride my stationary bike for 15 minutes before lunch.*

Point 2b) Sometimes you cannot carry out a planned behaviour. When that happens, it is a good idea to have a Plan B in place, an if-then strategy to mitigate setbacks.

<u>Examples of a Plan B</u>

Plan B: ***If** I do not do any exercises when I take my medication, **then** I will do 10 pelvic lifts and 10 crunches when I lie in my bed.*

Plan B: ***If** I do not ride my stationary bike before lunch, **then** I will go for a walk before I turn on the TV.*

Point 3) Try not to be over-ambitious based on former achievements or the idea of ”no pain, no gain”. The higher the ambitions, the lower the chances of you getting your exercises done.

<u>You do not make up for inactivity by setting high ambitions, you make up for inactivity by moving more.</u>

Activity: Ask the participants to choose at least one physical activity that they would like to implement in their daily routine and then ask them to make a plan on where, when and how to do it. After that, ask them to make a Plan B.

Bring paper and pencils (Duration 10 minutes)

Slide 8

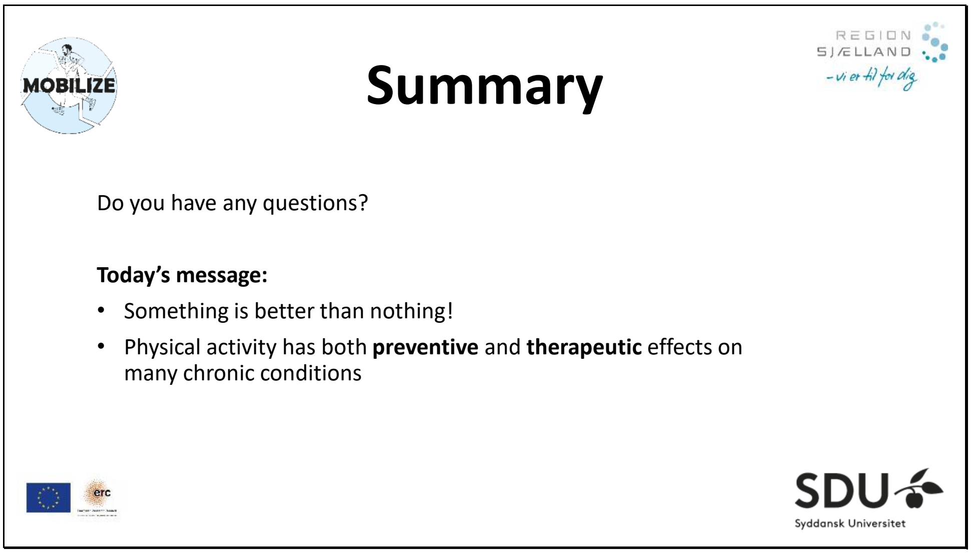

Answer any questions.

The most effective exercise is the one you'll actually do.

The best way to implement physical activity into your daily routine is to plan **when**, **where** and **how** to do it.

Try your plan to add more physical activity to your life.

Mindfulness

Slide 1

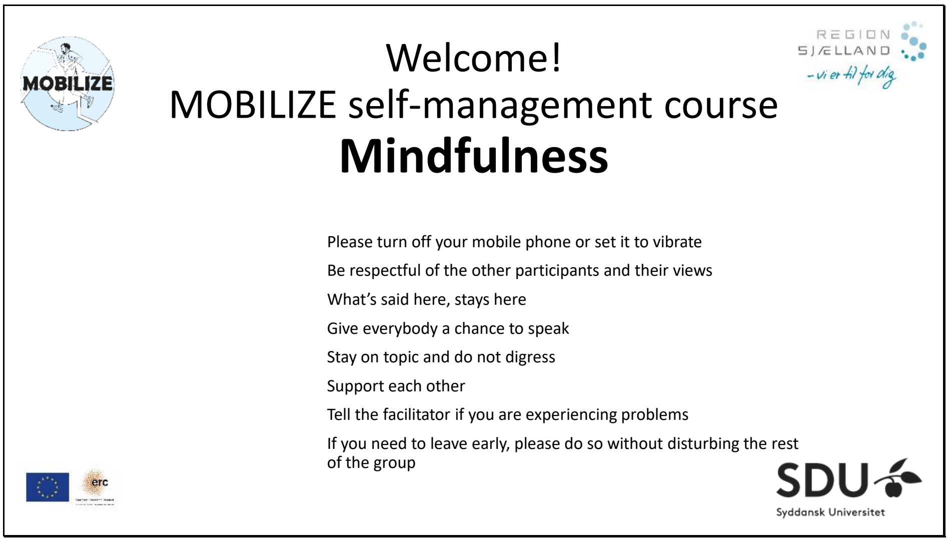

Welcome to the thematic module on mindfulness.

Today, we are going to talk about how you can use mindfulness as a tool to cope with your chronic conditions, similar to the way you exercise your muscles.

This is important to remember:
<u>Mindfulness is training for your mind and mental state</u>, and just like training your body it requires a lot of practice and consistency to get the most out of it. In addition, just like with physical training, sometimes it goes very well and other times it can be difficult to feel the effect. The same is true when working with mindfulness.

Slide 2

MOBILIZE

What is Mindfulness?

Mindfulness means living in the present moment and not in the past or future, while calmly acknowledging and accepting one's feelings, thoughts, and bodily sensations without judgement or filter.

Observe your thoughts and feelings (including the negative ones) without judging them.

Slide 3

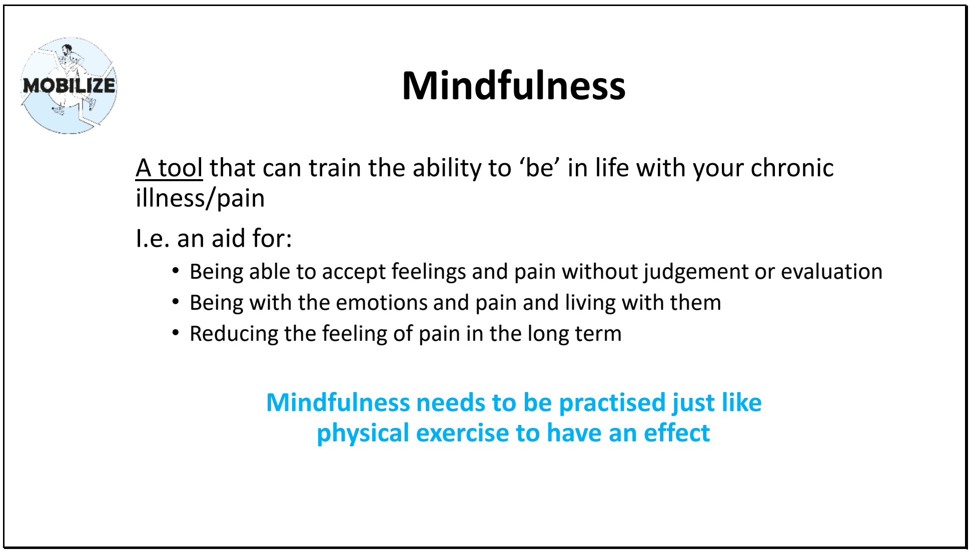

End of slide: When you relate to how you feel right now – not how you should feel or how you wish you felt – you are being mindful!

This allows you to become consciously present from moment to moment. By observing your thoughts, feelings and pain, rather than reacting to them and starting to act on them, you can let go of the past, the future and the desire for things to be different than they are.

This shift in attitude – welcoming whatever comes instead of fighting or endeavouring to remove it – creates a change in how you perceive and experience the present moment.

Mindfulness differs from psychotherapy by acting directly on the areas of the brain that regulate our emotions. It inhibits activity in these areas, making us think more objectively and clearly.

Slide 4

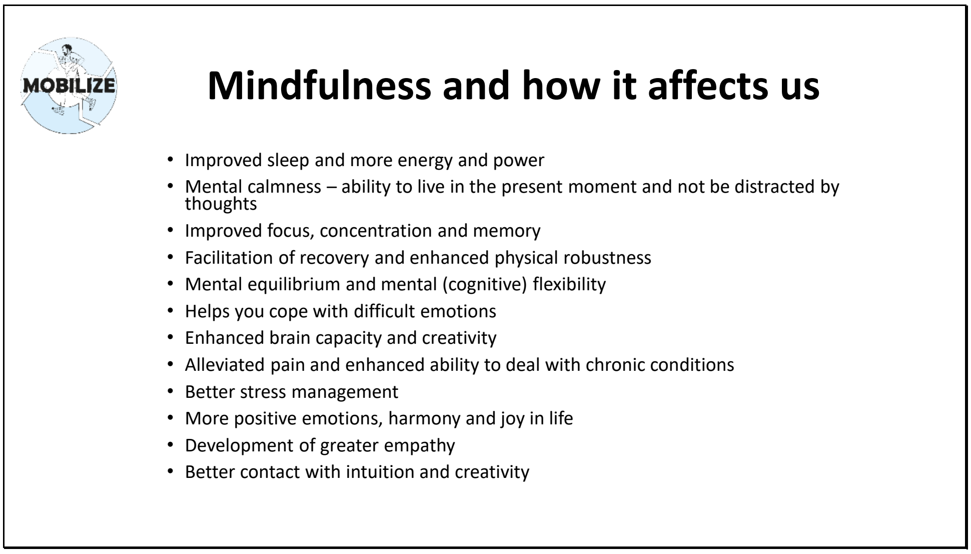

Introduction to slide: These are the findings of different research projects. Find the links in your patient handbook.

Slide 5

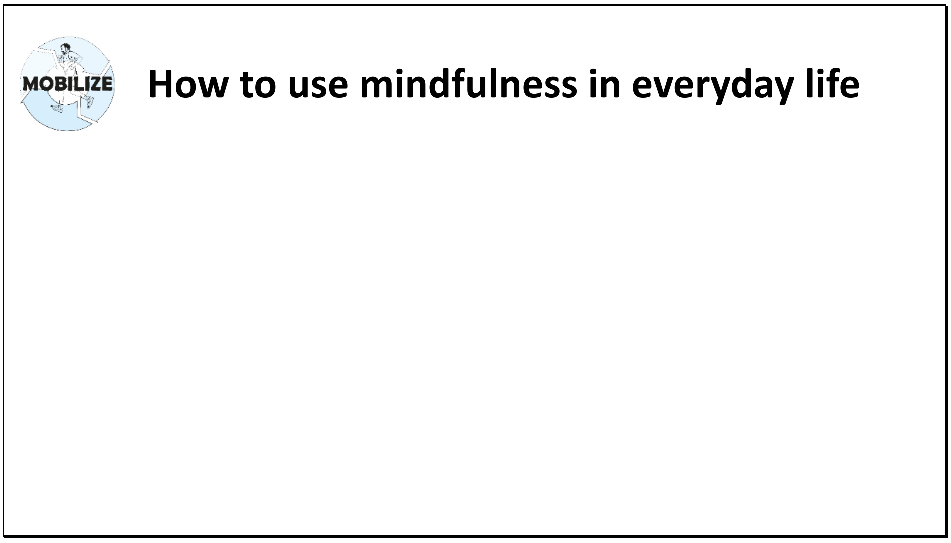

Mindful walking: During mindful walking, our journey is less about the destination and, while avoiding 'distracted autopilot,' more about bringing awareness to your surroundings and how your body and mind feel while moving forward – if you are not able to walk, you can still use this practice while in your wheelchair etc.

Mindful eating: Eating mindfully means that you are using all of your physical and emotional senses to experience and enjoy the food choices you make. Sit, slow down, savor and simplify - these are the basic tenets of mindful eating.

Mindfulness in nature: Allow the world around you to filter through each of your senses with openness and curiosity. Let your eyes, ears, nose, and sense of touch take it all in - fresh snowfall, the colour or crunch of autumn leaves or bird songs. If you live in the city, sit on a bench at your local park or outdoor shopping center and just notice people and objects coming and going.

Showing kindness to others is good for your health. It could be by giving up our seat on a bus to someone who might need it more or offering to make a cup of tea for someone at work.

Mindful communication involves applying principles of mindfulness to the way we correspond with others. These principles include setting an intention, being fully present, remaining open and non-judgmental, and relating to others with compassion.

Ask the participants: Have you tried anything like this before or can you think of situations where you could implement this in your everyday life? **(Dicuss. max. 10 minutes).**

Slide 6

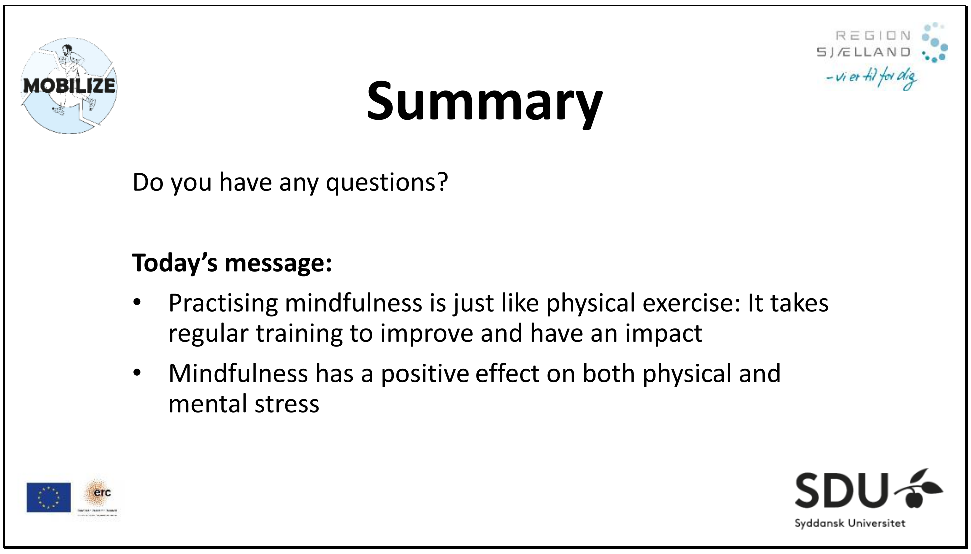

Answer any questions.

Encourage the participants to try mindfulness when they get the chance, even if it's only for five minutes.

Your story – patient narratives

Slide 1

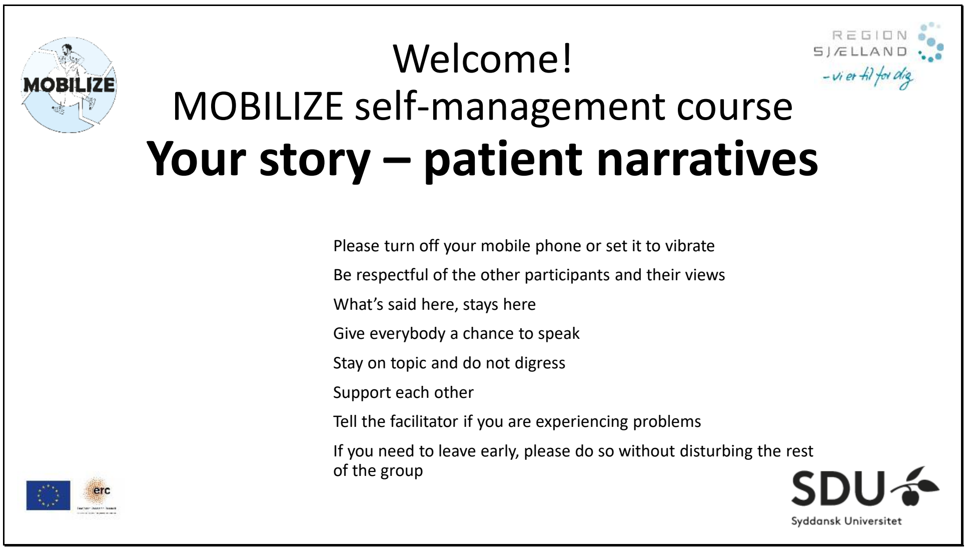

A video will be shown as part of this teaching session. Get the link ready:
https://www.youtube.com/watch?v=N3_oJ09aXu8&feature=youtu.be

Welcome to the thematic module on storytelling.

To share your story with others is a tool to manage your chronic conditions. Today, we are going to talk about how personal stories can serve to organise the otherwise raw and overwhelming sensations, disturbing feelings and thoughts that surround difficult life situations.

Slide 2

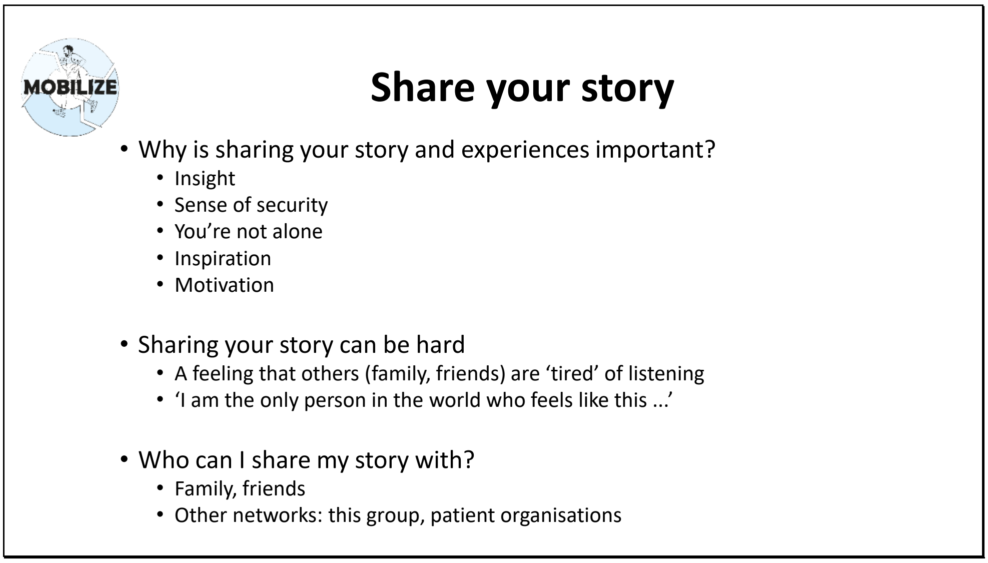

Share your story where you feel safe and heard.

Slide 3

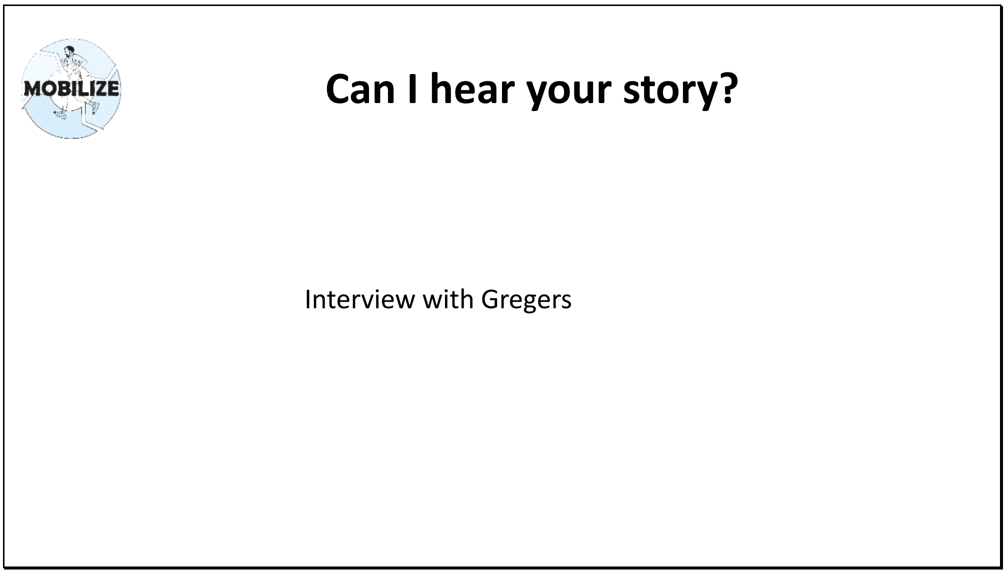

Introduction to slide: Gregers, who is one of our patient partners in MOBILIZE, has agreed to share his story of living with multiple chronic conditions.
Show the video to the participants:
https://www.youtube.com/watch?v=N3_oJ09aXu8&feature=youtu.be **(7 minutes)**

Slide 4

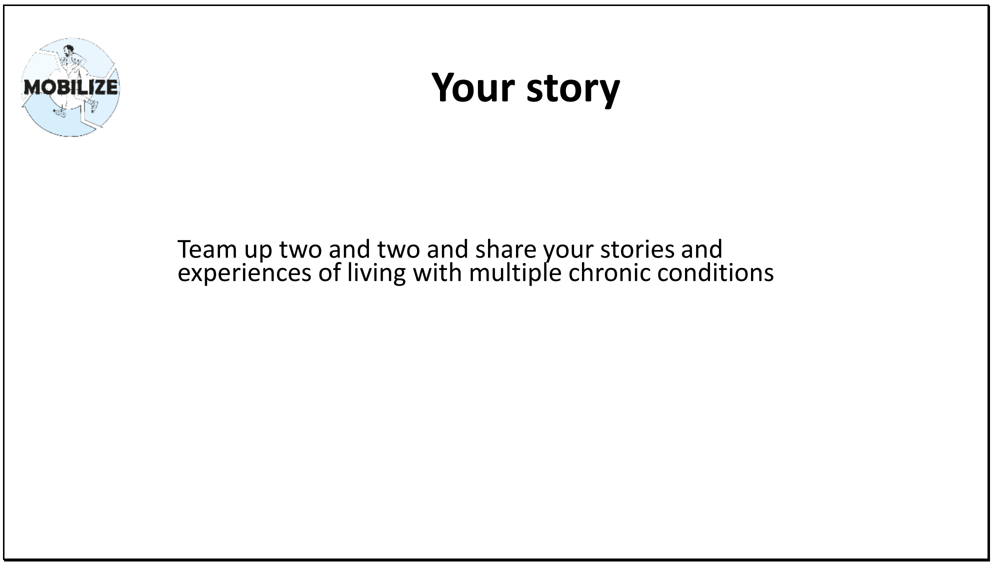

Sharing of experiences: After you have seen the video, ask the participants to team up two and two and share their stories: Take 5 minutes each and tell your fellow participant what it does to you to live with your chronic conditons. One speaks and the other one listens and then vice versa. **In case of an uneven number of participants, the facilitator can step in as the listener. The participant can afterwards join another group as the listener. (Duration: 5 minutes each).**

Joint recap in plenary (Duration: 5 minutes). What was it like to share your story? Was it a good experience? Taboo-breaking? Inspiring?

Slide 5

Patient organisations

Patient organisations can provide valuable information about your condition, treatment options and your rights as a patient.

For example:

- Hjerteforeningen
- Gigtforeningen
- Lungeforeningen
- Depressionsforeningen
- Psykiatrifonden
- Diabetesforeningen

Some patient organisations have patient partners involved and have forums where you can communicate with representatives of the organisations or other patients.
A membership fee is usually payable if you want full access to their services and resources, but you can also find a lot of free information on their websites.
Some of the patient organisations have local or regional groups that you can join.

Find useful links in the patient handbook.

Slide 6

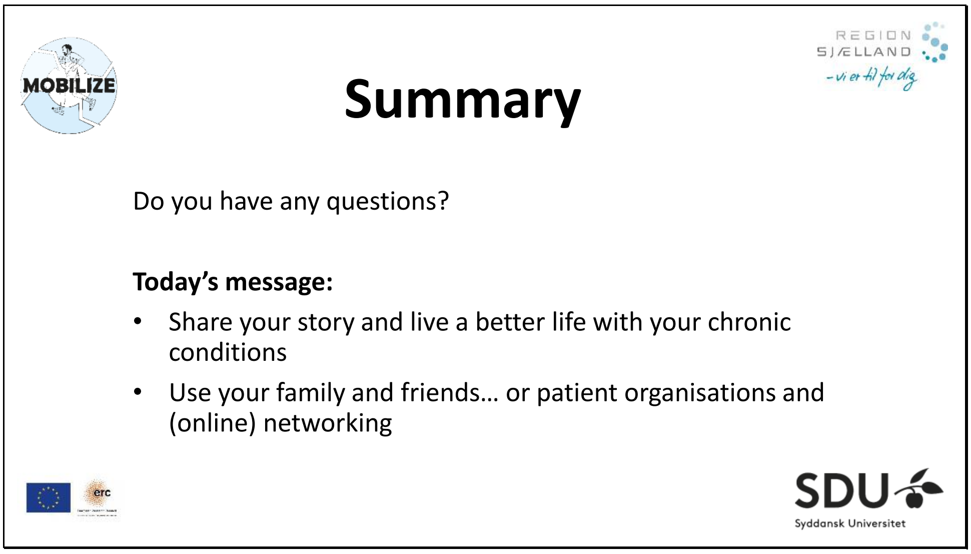

Answer any questions.
Remind the participants about the good things about <u>sharing your story</u> and being open to listening and <u>learning from others</u>.

General physical symptoms

Slide 1

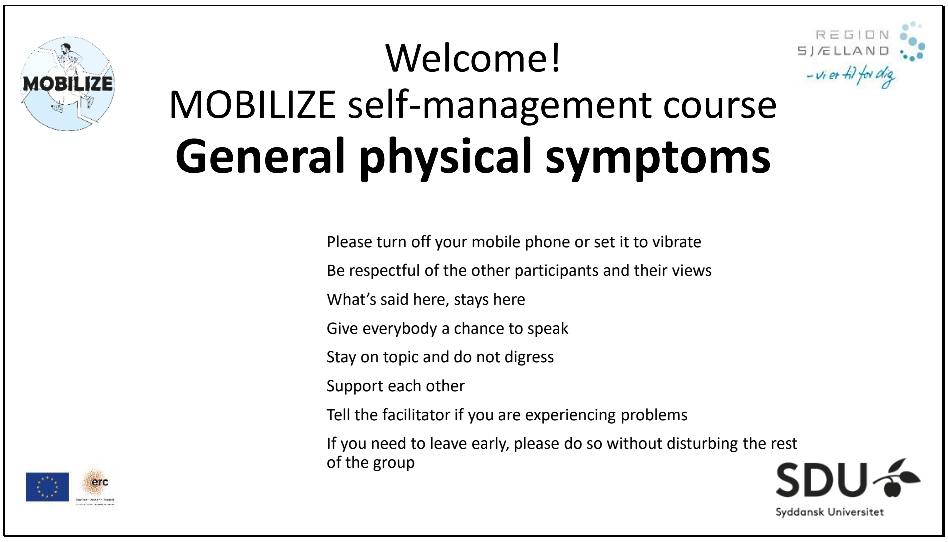

Welcome to the thematic module on general physical symptoms.

To live well with a chronic condition, you need to learn how to manage the changes and symptoms that may occur either due to the condition itself or as a result of your reactions to it.

Today, we are going to look at some of the general physical symptoms that may accompany chronic conditions and provide you with tools to manage such symptoms.

Slide 2

Shortness of breath

Shortness of breath can have many causes:

- ➢ Lung disease
- ➢ Heart disease
- ➢ Obesity
- ➢ Anxiety

Point 1) E.g. damage to the alveoli (tiny air sacs) of the lungs (emphysema). Tightness in your chest and excess mucus production (chronic bronchitis).

Point 2) If the heart muscle is unable to pump enough blood around the body properly.

Point 3) Obese people may experience shortness of breath because the excess weight puts excess pressure on their bodies.

Point 4) Anxiety may make you feel like you are losing control over your breathing, and this may result in hyperventilation.

Slide 3

MOBILIZE

How to relieve shortness of breath

- Gradually increase your level of activity
- Use relevant breathing techniques
- Check that you are receiving the right medication and dose
- Avoid smoke

Introduction to slide: Ask the participants what they do to ease shortness of breath before clicking further to the points. Do you use breathing techniques? Do you reduce your level of activity?

Point 1) Regular physical activity will ease the feeling of shortness of breath. WHO recommends at least 2½ - 5 hours of moderate physical activity or at least 1½ -2½ hours of vigorous physical activity in a week.

Point 2) You can practice pursed lip breathing. This technique helps to keep airways open longer so that you can remove the air that is trapped in your lungs by slowing down your breathing rate and relieving shortness of breath.

Point 4) Avoid smoking yourself or secondhand smoke from other smokers/sources.

Slide 4

MOBILIZE

Pain

Pain can have many causes:

- Inflammation/damage to joints or tissue
- Sleep deprivation
- Psychological factors
- Prescription medications

Introduction to slide: Pain is a common problem for people with chronic conditions.

Point 1) By inflammation we mean your body's response to harmful stimuli and not an infection from a virus or bacteria. Pain may also come from insufficient blood flow to the heart muscle or compression of nerves in the spinal canal or tissue damage.

Point 2) Insufficient sleep or poor quality of sleep can also be a factor.

Point 3) E.g. headache, if you feel stressed or anxious, or stomach pain due to worries and fears.

Point 4) Prescription medications may cause different side effects/discomfort.

End of slide: Pain can have many causes and the degree of pain may not be commensurate with the severity of the condition.

There is more information on pain in the module 'Pain mechanisms'.

Slide 5

MOBILIZE

How to relieve pain

- Physical activity and movement
- ‘Pacing'/cut up activity into manageable chunks
- Breathing exercises
- Write a pain diary
- Connect with people who are dealing with similar pain issues

Introduction to slide: Ask the participants how they handle pain before clicking further to the points. Do you take pain medication? Do you stop doing things that you used to do? Do you try to sleep away the pain?

A person with chronic pain should focus on working towards reducing the pain to a manageable level (so that it doesn’t control everything) rather than eradicating it entirely.

Point 1) Evidence shows that <u>physical acitivity can reduce pain</u>. The body produces its own natural pain-relieving hormones.

Point 2) A <u>pain diary</u> can help you see any connections between your pain and certain activities. If you can see the connection, you will be able to adjust your activities and reduce your pain. It is **<u>not</u>** recommended to complete a pain diary on a daily basis though as this may end up putting more focus on your pain.

Point 3) Find the balance between activity and rest. If you maintain a good balance, you will have a better day and more energy later in the day.

Point 4) Deep breaths will keep you calm and take focus away from negative thoughts and pain.

Point 5) Chronic pain is an invisible disability that can be difficult to understand. It can bring great relief to simply meet other people with chronic pain as this can make you feel understood and feel 'normal' around others.

Slide 6

Sleep disorders

- Difficulty getting to sleep
- Difficulty staying asleep
- Waking up too early and not feeling refreshed the next morning

Introduction to slide:
Sleep is essential to every process in the body, affecting our physical and mental functioning. Most people know what it is like if you get too little sleep or a poor quality of sleep. It affects your mood, you may find it difficult to remember things and concentrate. When you're tired, you're also less patient and less pleasant to be around. The purpose of a good night's sleep is to relax your body and shut off your mind, rejuvenating yourself for another day.

End of slide:
A single night of lost or poor sleep can be recovered.
But inability to initiate or maintain sleep can make daily life feel more stressful and less productive, and it can also make your pain worse as sleep problems will often affect the release of natural painkillers in the nervous system.

Chronic sleep deprivation is linked to numerous physical and mental health issues. It may weaken your immune system and increase the risk of diabetes, obesity, mental illness, heart disease, accelerate the development of Alzheimer's and ultimately lead to premature death.

If you struggle with sleep problems for more than 6 months, you should ask for help.

Slide 7

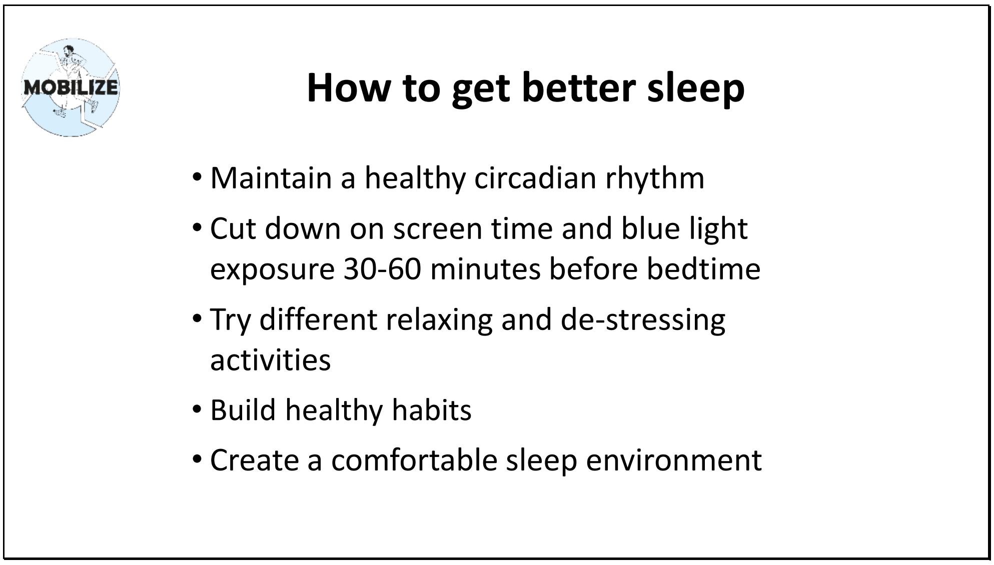

Introduction to slide: Ask the participants what they do to get a good night's sleep before clicking further to the points.

Research shows that good sleeping habits are essential for maintaining your health and wellbeing.

Point 1) Keep a regular sleeping schedule. Try to wake up at the same time every day, weekday or weekend.

Point 2) Computers, tablets and television.

Point 3) Meditation, mindfulness, breathing exercises.

Point 4) Be physically active, avoid alcohol and caffeine after 4pm.

Point 5) Have a good mattress and a dark, quiet and cool bedroom.

Slide 8

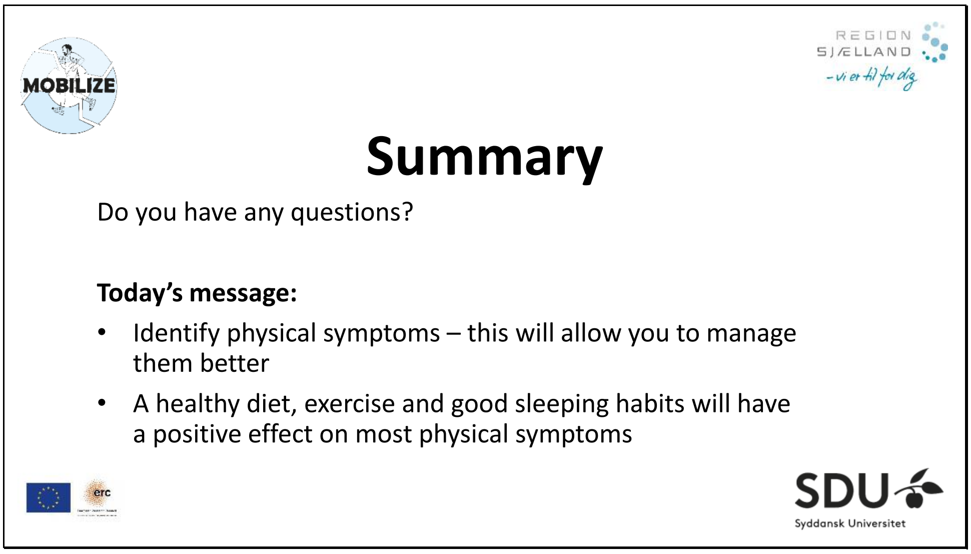

Answer any questions.

Remind the participants that they can consult healthcare professionals and patient organisations and get help.

Healthy eating habits

Slide 1

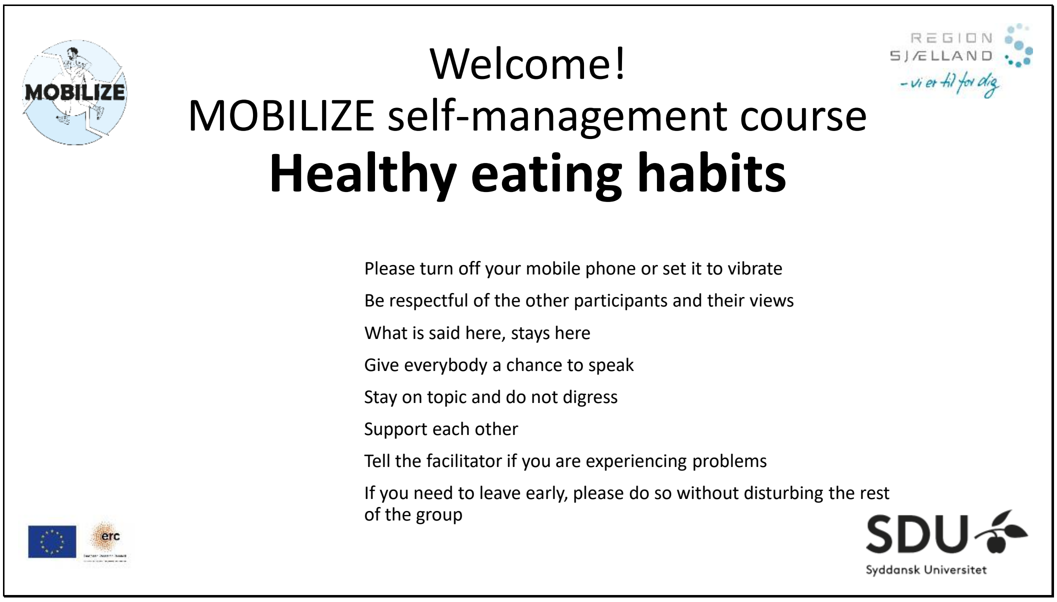

General considerations before teaching:
Before the lesson starts, it may be a good idea to find out whether there are participants who need to lose or gain weight, so that you can address these needs when the various macronutrients (carbohydrate, fat and protein) are presented.

The aim of the session is to make the participants aware of what food and meals consist of (fat, protein and carbohydrate), so the participants gain knowledge about which parts of the meal they might benefit from eating more or less of, depending on whether they need to eat more 'healthily' or maintain/increase/reduce weight.

Remember that food is much more than nutrients – taste, enjoyment and the social aspect are also important. Feel free to ask the participants what they like and use that as a starting point.

Bring a glass and 25 sugar cubes to illustrate slide 7.

Welcome to the thematic module on healthy eating habits – today we'll focus on what our food and drink consists of.

Despite the many different recommendations, we go through today, don't think that you have to live up to all recommendations every day. Think of it more as inspiration for how perhaps by changing a few things you can achieve a healthy balance in your diet, regardless of whether you are seeking to lose, gain or maintain weight.

Slide 2

What does our food consist of?
- fat, protein and carbohydrate

- Example 1: Salmon with hollandaise sauce, potatoes and asparagus.
- Example 2: Meatballs with potatoes, gravy and mixed vegetables.
- Example 3: salad with fetacheese.

<u>Examples of main meals.</u>
Our food consists of fat, protein and carbohydrate. Here are three meals in which fat, protein and carbohydrate are represented.
Example 1) is salmon with hollandaise sauce, potatoes and asparagus.
Example 2) is meatballs with potatoes, gravy and mixed vegetables.
example 3) is a salad with feta cheese.

Ask the participants: Do you know what is fat, protein and carbohydrate in the three examples? - Fat is the sauce, the gravy and the feta cheese in the examples; protein is the salmon and meatballs; and carbohydrates are the potatoes and vegetables.
Fat, protein and carbohydrates are vital nutrients, as they supply the body with energy and ensure the building and maintenance of the body's tissue.

Questions for the participants:
What does your main meal look like? Where do you get fat, protein and carbohydrates from? Are all three nutrients represented? Are there some parts of the meal that taste better than others? What is the most important part of your main meal? **(Duration max. 5 min)**

Slide 3

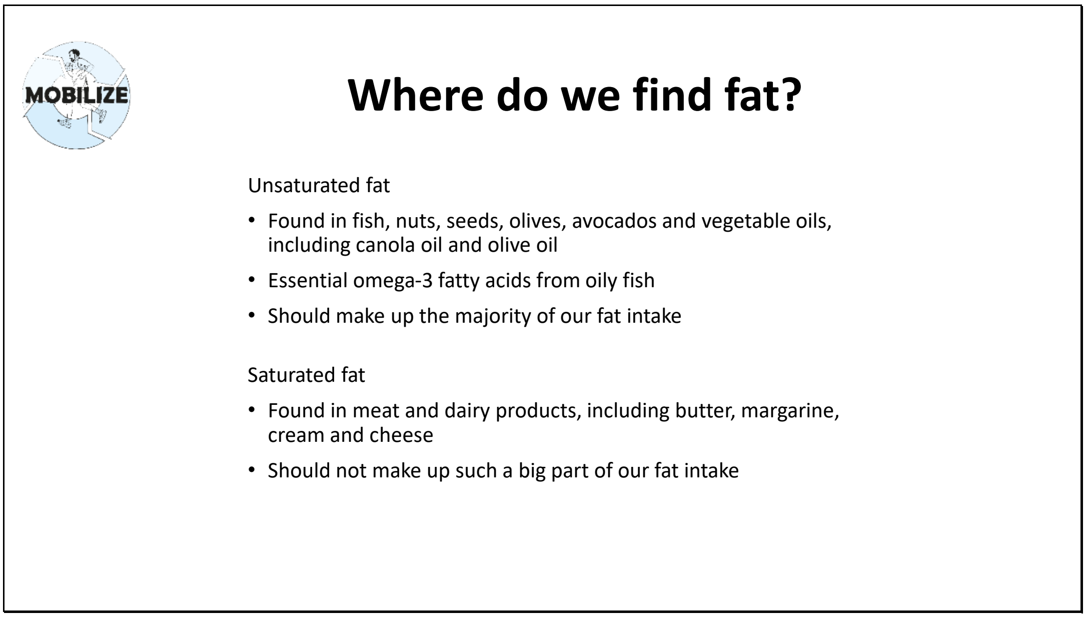

Ask the participants (before clicking on to the sub-points): Which foods are high in fat?

Introduction to slide: It is important that we eat fat, as it ensures the building of hormones and cells in the body. In addition, it ensures that the fat-soluble vitamins A, D, E and K are absorbed in the intestines.
Fat can be divided into unsaturated and saturated fat.

Point 1) <u>Unsaturated fat</u> should make up the majority of the fat we consume, as it lowers cholesterol and prevents hardening of the arteries (atherosclerosis). Unsaturated fats are found in fish, nuts, seeds, olives, avocados and vegetable oils such as canola oil and olive oil.

Point 2) <u>Saturated fat</u> should not represent such a large part of the fat we consume, as it increases cholesterol and promotes arteriosclerosis. Saturated fat is found in meat and meat products as well as in dairy products, including butter, margarine, cream and cheese.

However, it is important to point out that you do not have to omit meat and dairy products from your diet but choose the lean versions (e.g. chicken or minced meat with the lowest fat

percentage), as they contain less saturated fat and at the same time contribute protein and important vitamins and minerals to the diet.

Fat is also the part of our food that contains the most energy per unit of weight, so if you want to lose weight it might be an idea to reduce the amount of fat in your food (e.g. save on butter and cream) – and vice versa: if you want to gain weight, it can be a good idea to add more fat to your food (e.g. cream, butter and olive oil).

Questions for the participants:
What kind of fat do you eat? Why have you chosen this? (the taste, it's easy, the price, what I know...)

Slide 4

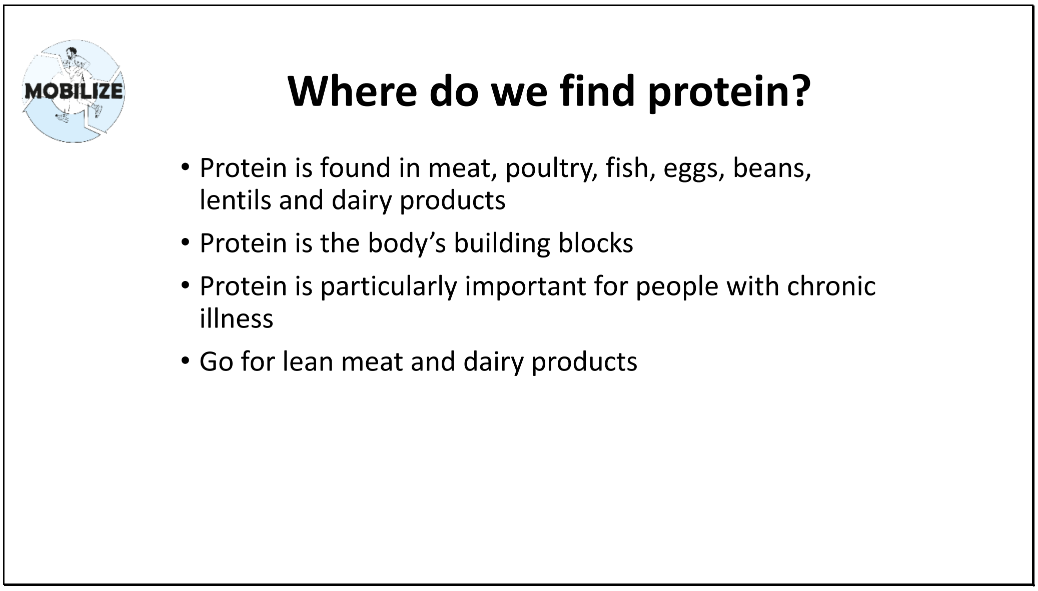

Ask the participants (before clicking on to the sub-points): Which foods contain protein?

Point 2): It is important that we eat <u>protein</u>, as it is used to build the body’s cells, tissue, hormones, antibodies and enzymes.
Protein therefore plays a vital role in building and maintaining our muscles. Protein is found in meat, poultry, fish, eggs, beans, lentils and dairy products, including cheese, milk, yoghurt and crème fraîche. The dairy products skyr and Protino (from Arla) have a particularly high protein content.

Point 3): When you have one or more <u>chronic conditions</u>, you have an increased need for protein compared to healthy people. That is why it is particularly important to consume protein every day if you have a chronic condition.
If you have a <u>small appetite</u> and don’t eat very much, it is especially important to make sure you get enough protein. For instance, you may have to cut back on the amount of vegetables and potatoes in favour of meat, as too large amounts of vegetables/potatoes/pasta may mean that you do not have enough appetite to eat sufficient protein.

Questions for the participants:
Where in your diet do you get protein? Why have you chosen this? (the taste, it’s easy, the price, what I know...)

Slide 5

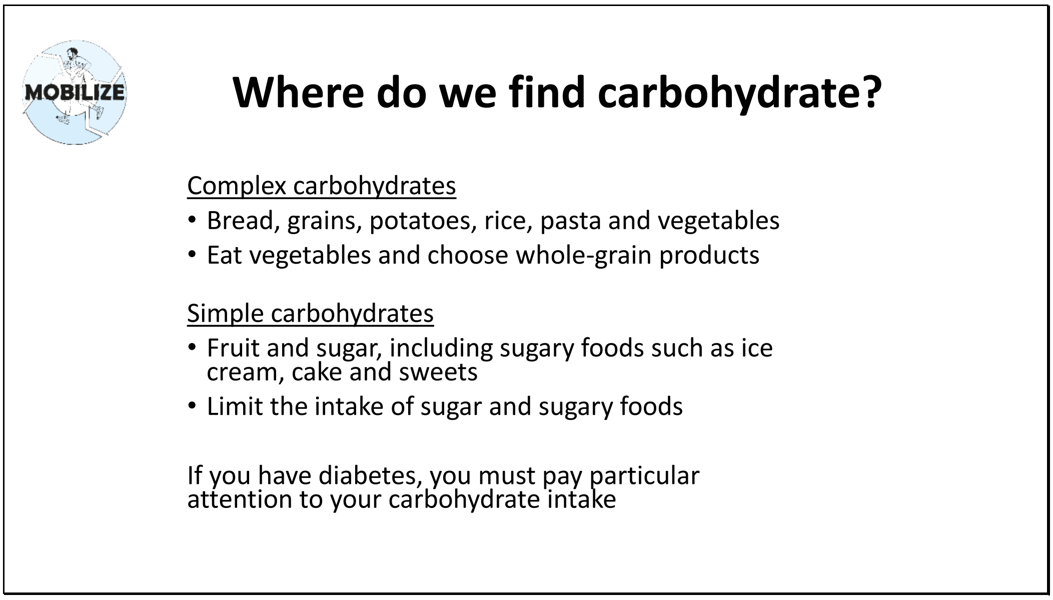

Ask the participants (before clicking on to the sub-points): Which foods contain carbohydrates?

Introduction to slide: It is important that we eat carbohydrate, as it is the body's most important source of energy and thus crucial for our body to function.
Carbohydrates can be divided into complex and simple carbohydrates.

Point 1) <u>Complex carbohydrates</u> are found in bread, grains, potatoes, rice, pasta and vegetables. Vegetables contain many vitamins and minerals and dietary fibre and should therefore make up a large part of the carbohydrate we consume. Potatoes should be included in several meals a week, as they provide good satiety and are also rich in vitamins. It is recommended to choose whole grain varieties when eating bread, rice and pasta, as they are more filling and contain more dietary fibre than white bread, pasta or rice. Oatmeal is a whole-grain product in itself, and it is beneficial when included as part of a healthy diet.

Point 2) <u>Simple carbohydrates</u> are found in fruit and sugar, including sugary foods such as ice cream, cake and sweets. Like vegetables, fruit contains lots of vitamins, minerals and dietary fibre, and can be consumed daily. Sugar and sugary foods should not make up such a large part of the carbohydrate we consume, as it contributes many calories and takes the place of healthy food, which makes it difficult to get the vitamins and minerals the body needs.

End of slide: When you have diabetes, it is a good idea to pay particular attention to your carbohydrate intake, as carbohydrates cause blood sugar to rise. However, there is a difference in how the different foods affect the rise in blood sugar. Complex carbohydrates give a slower and more controlled rise in blood sugar, while simple carbohydrates give a faster and more spontaneous rise in blood sugar. When you have diabetes, it can therefore be an advantage to go mostly for complex carbohydrates, as this can make it easier to avoid excessive fluctuations in blood sugar.

Be aware that if you have difficulty eating enough/maintaining weight, it may be appropriate to cut back on coarse vegetables and whole-grain products, as they can be very filling, which is not appropriate if you have a reduced appetite or difficulty maintaining weight. At the same time, it may also be better for those who need to gain weight to eat a piece of cake or a protein-enriched ice cream rather than an apple for a snack.

Questions for the participants:
Where in your diet do you get carbohydrates? Do you get both complex carbohydrates and simple carbohydrates?

Slide 6

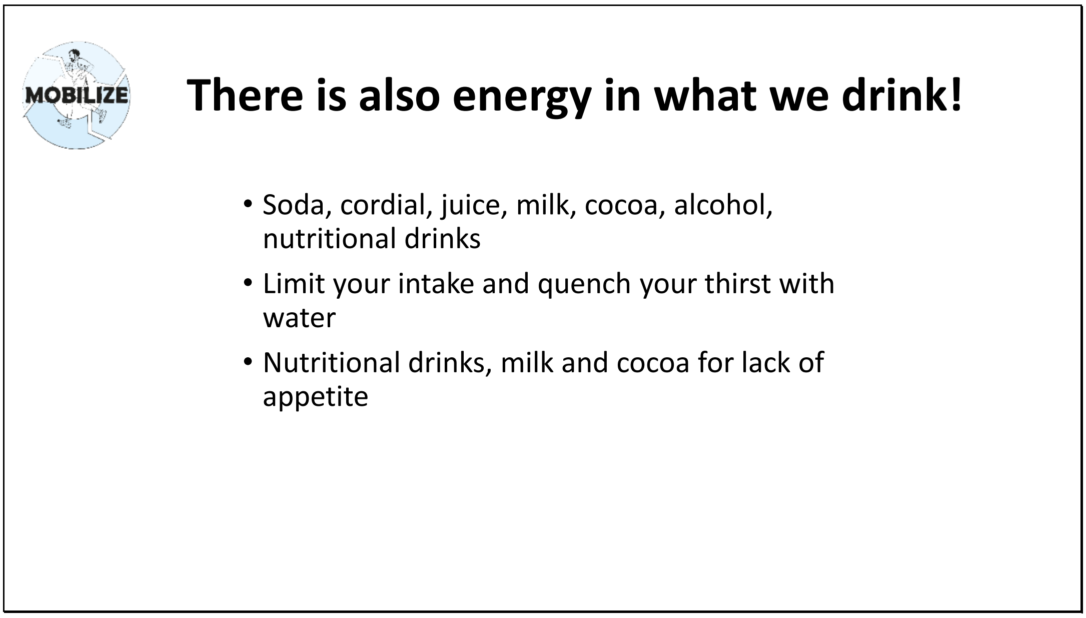

Points 1–2): As well as from our food, we can also get energy, carbohydrates, fat and protein from our drinks. This could be soda, cordial, juice, milk, cocoa, alcohol and nutritional drinks. It is recommended to limit the intake of energy drinks, as they contribute many calories and take the place of healthy food. Instead, thirst can be quenched with water.

Alcohol does nothing good for your health, and according to the Danish Health Authority, adults (men and women) should not drink more than 10 units per week and no more than 4 units on the same day.
1 unit corresponds to approx. 1 beer, 1 glass of wine or 1 cocktail.

Point 3): If you have a decreased appetite, it can often be difficult to achieve sufficient energy intake through food alone, so in this case you can benefit from supplementing your diet with nutritional drinks, milk and cocoa, as these contain both protein and fat, which contributes a lot of energy to the body.

Slide 7

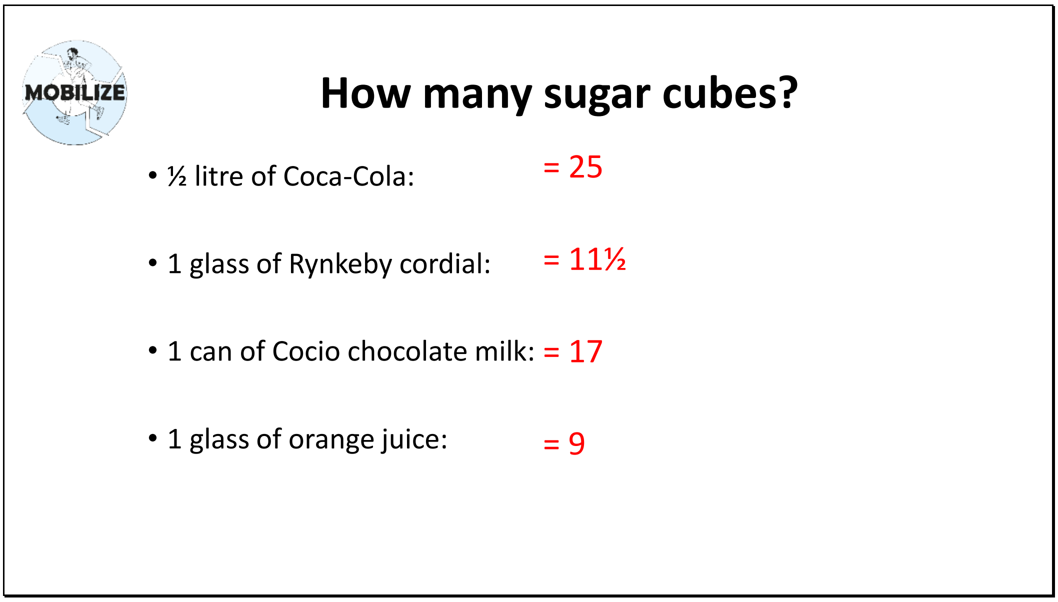

Ask the participants how much sugar they think is in soda, cordial, cocoa milk and juice before you click on to the next point.
½ litre of Coca-Cola: 25 sugar cubes
1 glass of Rynkeby cordial: 11 ½ sugar cubes
1 can of Cocio chocolate milk: 17 sugar cubes
1 glass of orange juice: 9 sugar cubes

Visualise this for the participants by showing them a glass filled with 25 sugar cubes.
The point is that even though they are 'liquid', drinks can also contribute a lot of calories and a lot of energy.

Slide 8

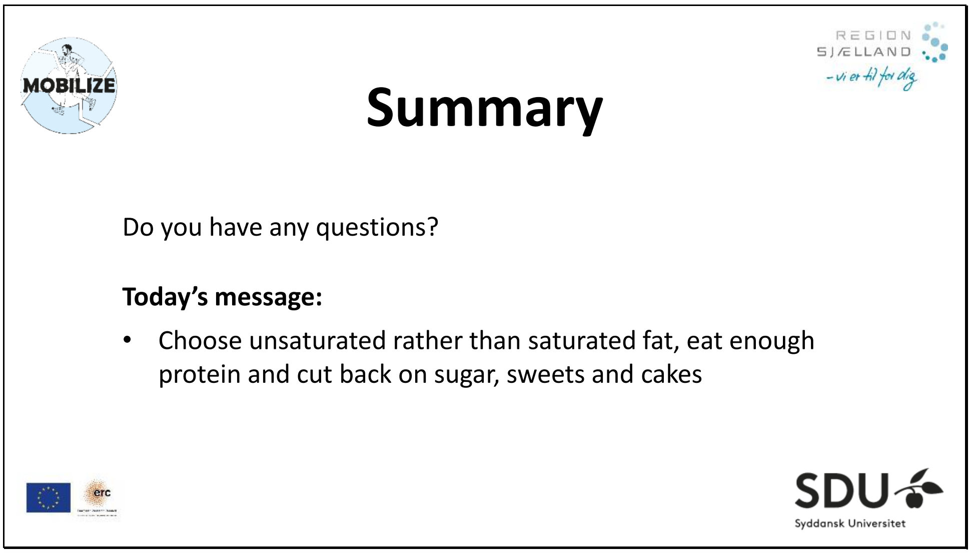

Answer any questions.

Suggestions for home activities:
Pay attention to the fat you eat during the day – is it saturated or unsaturated?
Are there changes you can make naturally to switch from saturated fat in favour of unsaturated fat?
See more in your patient handbook.

Finish by reminding the participants that they are welcome to bring their relatives to the next module on Carers/Relatives.

Involving carers/relatives

Slide 1

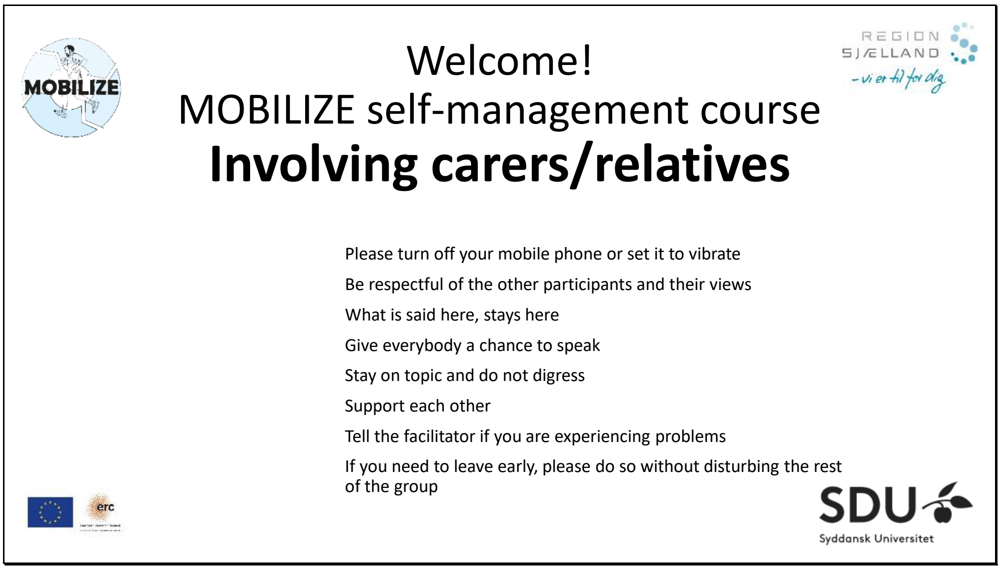

This is one of the modules where MOBILIZE recommends that carers/relatives are invited to participate.

Welcome to the thematic module on carer/relative involvement.

The theme of the day is carers/relatives and their roles: first, as a resource in the process; second, as a human being and 'co-sufferer' with their own support needs.

Traditionally, caregivers were people with family ties, but in recent times the concept has been opened up to include other close acquaintances such as friends, neighbours, colleagues, etc.

The Danish Health Authority defines carers as follows: 'Carers can be close relatives but can also be a good friend. The decisive factor is the actual connection.'
The Danish Health Authority. Guidelines on healthcare professionals' duty of confidentiality – dialogue and collaboration with patients' carers. Copenhagen: The Danish Health Authority; 2002; www.retsinformation.dk

First and foremost, carers are a valuable human support for the patient. In addition, they may be involved in a wide range of different tasks in the patient's course of illness. Carers may notice any progress or decline in the patient's condition, and they may detect errors or irregularities in treatment and care, thereby also having an impact on patient safety.

Due to healthcare professionals' duty of confidentiality, patients must always give their consent for relatives to be involved or receive information. Thus, by giving their consent the patient formally invites their carers into the partnership on illness and treatment along with the relevant healthcare professionals.

Slide 2

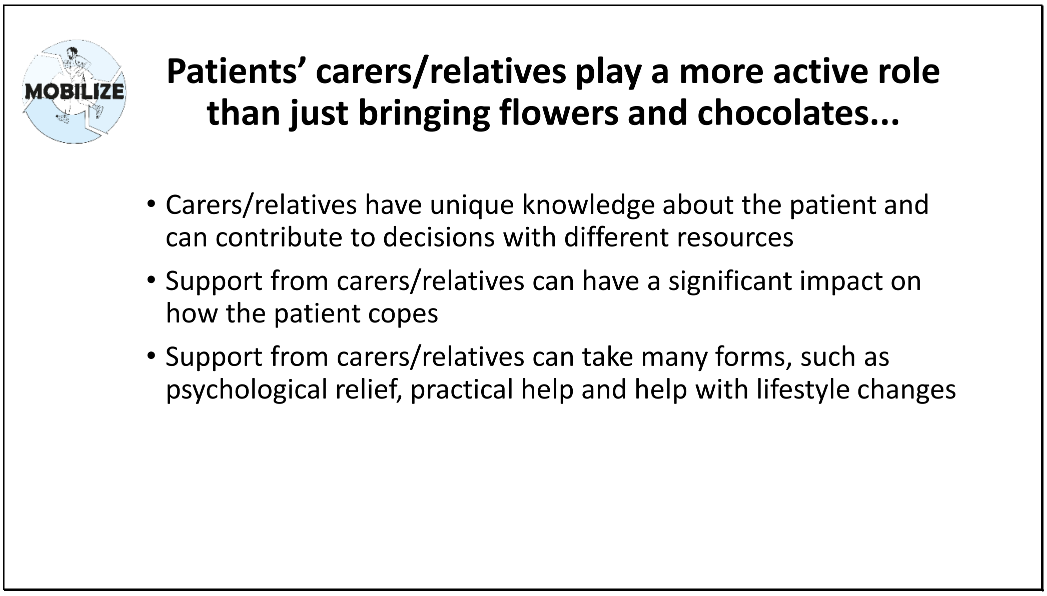

Introduction to slide: Two out of three Danes want to actively participate in treatment and want to help their partner with daily life if their loved one falls ill.

Points 1 and 2) A number of scientific studies indicate that carer involvement matters:

- better treatment outcomes for the patient
- fewer mistakes are made
- both patient and carer are better able to cope emotionally with critical situations

Point 3) Support from carers can take different forms:
Psychological relief includes listening, comforting and helping to reduce stress in everyday life
Practical help includes shopping, cooking and driving to doctor's appointments
Help with lifestyle changes can include help with dietary changes, as this is potentially something that affects family and social relationships (food is often consumed in social communities). Family support is important here.
In relation to creating new exercise habits, carers can play an active role in supporting the patient in maintaining new exercise habits.

Slide 3

How are you?

Living with or caring for someone with chronic conditions can be hard work. Here are some lessons learned by carers/relatives of patients with heart disease:

- Communicate
- Feel free to ask questions - it's also important to listen
- Make sure to see each other or call each other
- Be mindful to discuss things that might be difficult
- Try to look forward to the positive aspects

A good piece of advice is to ask "How are you?" and then, based on the response, decide whether to offer help or simply listen.

Exchange of experiences among the participants:
Talk about your experiences of where and how you have sought help when things get tough.
(Duration: approx. 10 minutes)

Slide 4

Supportive goal setting

Ask your carers/relatives to help you by setting a supportive goal...

'I want to acknowledge and praise my wife every time she comes home and completes a training session...

– instead of complaining and making her feel guilty every time she leaves.'

Introduction to slide: Just as you can set goals for yourself, your carer can do the same to support you.
You can set a goal together at home to support a lifestyle change, for example.
The goal should be encouraging but realistic.
Talk about what can be a facilitator or a barrier to achieving the goal.
Small steps can make a big difference.

Click on the example of a supporting goal.

Then encourage the participants and their carers to go home and think of one concrete thing that the carer can do to support the participant in their everyday life.

See tips in the patient handbook you have been given.

Slide 5

Being a carer/relative

Please keep in mind:

- Being a carer/relative of someone who is ill can be a very demanding role
- Carers/relatives (also) need to regain energy before they can revitalise their support and help
- Help is available from your own doctor, patient organisations, volunteers, carers'/relatives' groups, the municipality, etc.

It's important to talk – and look after yourself and each other

Point 1) It can be a great help for the chronically ill patient to get support and help from carers, BUT being a carer can also be hard work.
They have to deal with many things: their own needs and boundaries, their relationship with the patient and the encounter with the healthcare system.
That is why it is important to match expectations.

Point 2) Relatives often hold back their own thoughts and feelings due to a 'it's not me who's ill' mentality and are instead more concerned with talking about the patient.

Every second carer is emotionally burdened when a family member is affected by illness. According to studies from several patient organisations, many caregivers suffer from stress, post-traumatic stress, assess themselves as having poor health in general and they often have to leave the labour market early because they have become ill from their caregiving role.

It is crucial that caregivers are acknowledged and involved on their own terms if they are to live good lives while being a stable resource for their loved ones.

Your relationship may change and it may suddenly become difficult for the carer to look at their partner without seeing a patient, which can have consequences for the relationship (including intimacy and sexuality). It is important to be able to talk as openly as possible about what your

individual needs are in the new situation and how you can best manage everyday life. Be aware that your needs may change during the course of the illness.

Point 3) If carers feel overburdened, there are various support measures that can improve their well-being and help them cope with their everyday life to prevent them from becoming ill themselves – and no longer having the energy to provide support.

Find links to where carers can get help in the patient handbook provided.

Slide 6

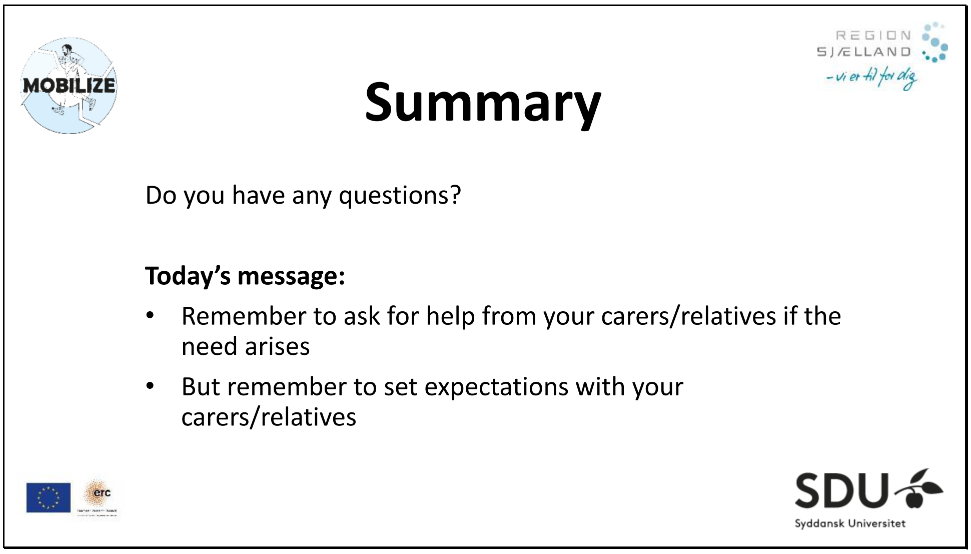

Answer any questions.

Remind participants about striking a balance between remembering to ask for help and remembering to care for loved ones.

Stress reactions

Slide 1

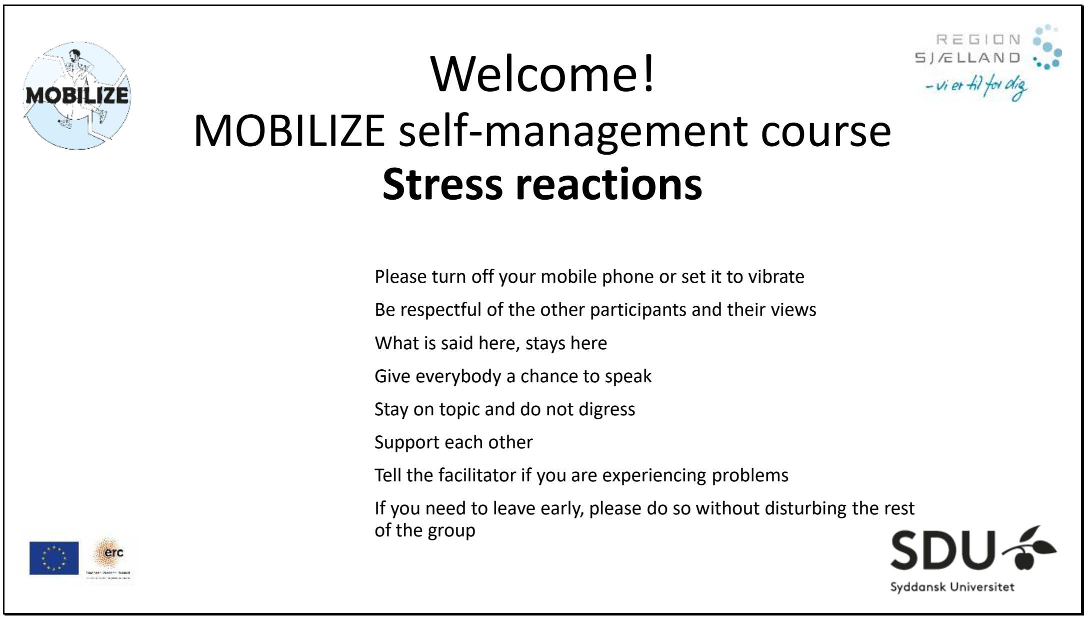

A whiteboard/flipchart should be used for this lesson.

Welcome to the thematic module on stress reactions to chronic illness.

Today's programme focuses on the reactions that can arise in connection with chronic illness.

Chronic illness or chronic pain can often affect our lives to the extent that we have to find new ways to live with the consequences of the illness or pain.

It can be very difficult to overcome chronic pain or live with a chronic condition.

It can often manifest in emotional and physical reactions that make one's chronic condition or pain worse.

Slide 2

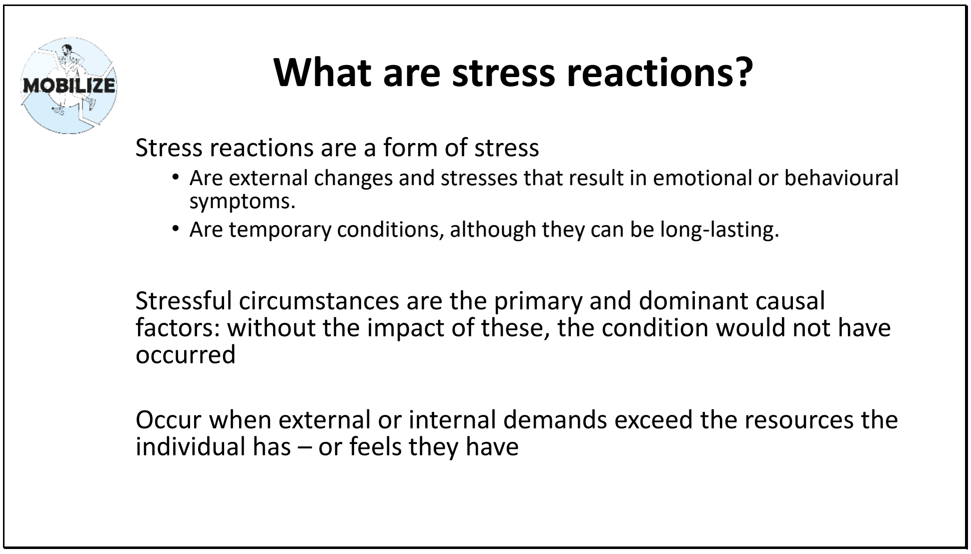

Paragraph 1) *Definition from sundhed.dk.*

Paragraph 2) Working conditions, life crises (including chronic illness), family problems, social circumstances can cause temporary crisis-triggered depression. This not to be confused with known recurrent/chronic depression.

End of slide:
In this session, we focus on the possible stress reactions that can occur as a result of your chronic condition and pain.

Slide 3

Reactions to living with chronic illness or pain:

- Anger, anxiety, depression, sadness, giving up and meaninglessness
- Difficulty being social
- Mood and temperament are challenged
- Self-esteem and identity are affected
- Physical limitations

Introduction to slide: Now, I will mention a whole lot of reactions that can be both shameful and difficult to deal with, but they are all perfectly normal reactions to a difficult situation.

Point 1)
You may experience one or more of these feelings.

Point 2)
Difficulty being with other people or making sure that the family is functioning.

Points 3–4)
It can be difficult to accept that you are no longer able to do the same things as before – and you can easily push yourself too hard or exceed your own limits.
It can be difficult to deal with others treating you differently because of your illness.
Many people feel pressured to move on quickly and accept that life now looks different.

Point 5)
Stamina and physical energy are not the same. There may also be restrictions to physical movement and the need for aids, which may make some people feel stigmatised.

Slide 4

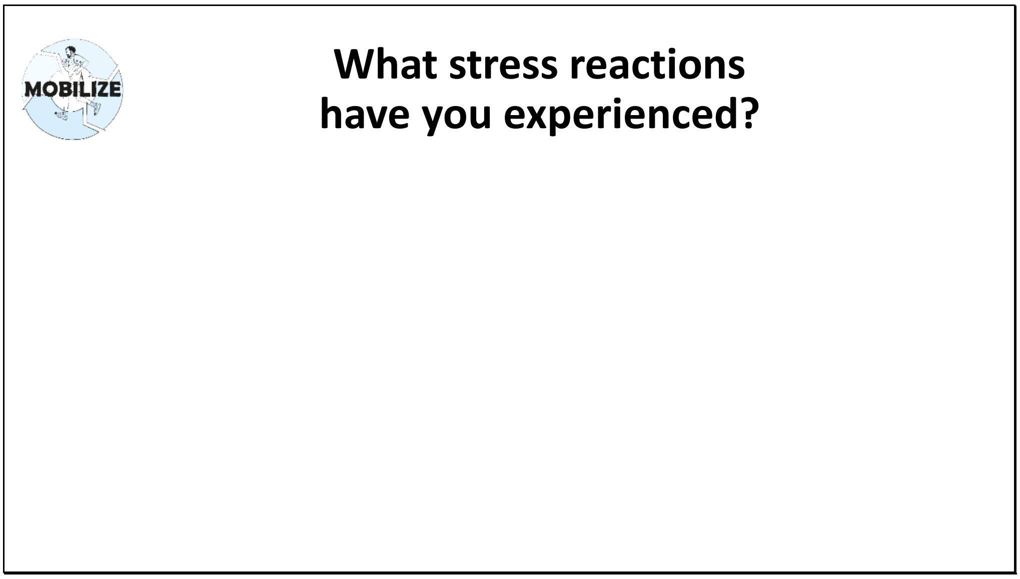

Ask the participants: What stress reactions have you experienced?
Write their answers on whiteboard/flipchart (Duration 5 minutes).

Slide 5

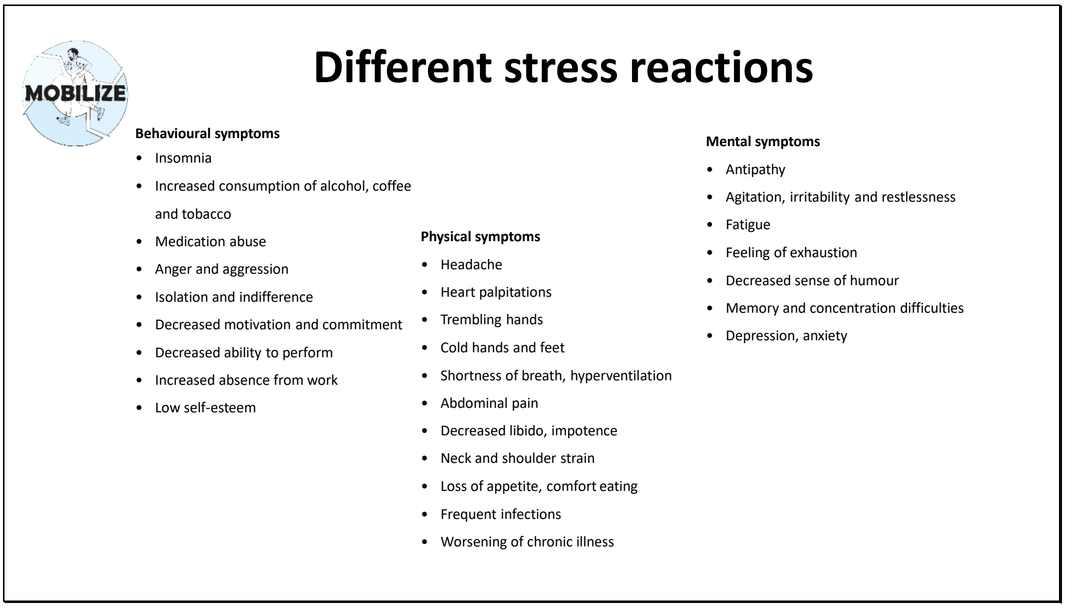

Here is a list of the stress reactions that various research studies have identified.

Highlight any that the participants themselves have identified.
Ask them if they recognise any stress reactions, they haven't mentioned themselves.

Slide 6

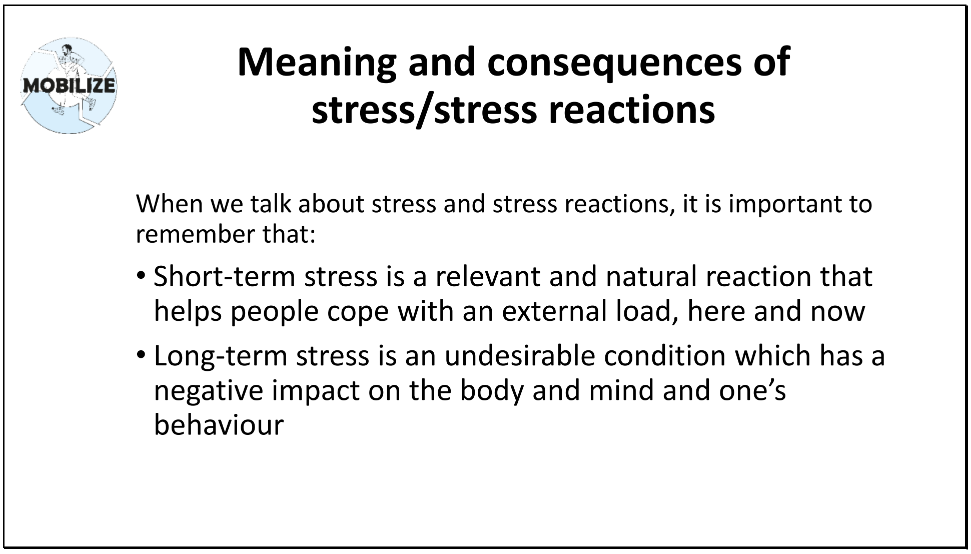

Point 1) Short-term stress can enable us to perform at our best and is originally a ‘fight or flight’ response. For example, an exam or a sporting event.

Point 2) It is a risk factor for the progression or worsening of the illness.

Slide 7

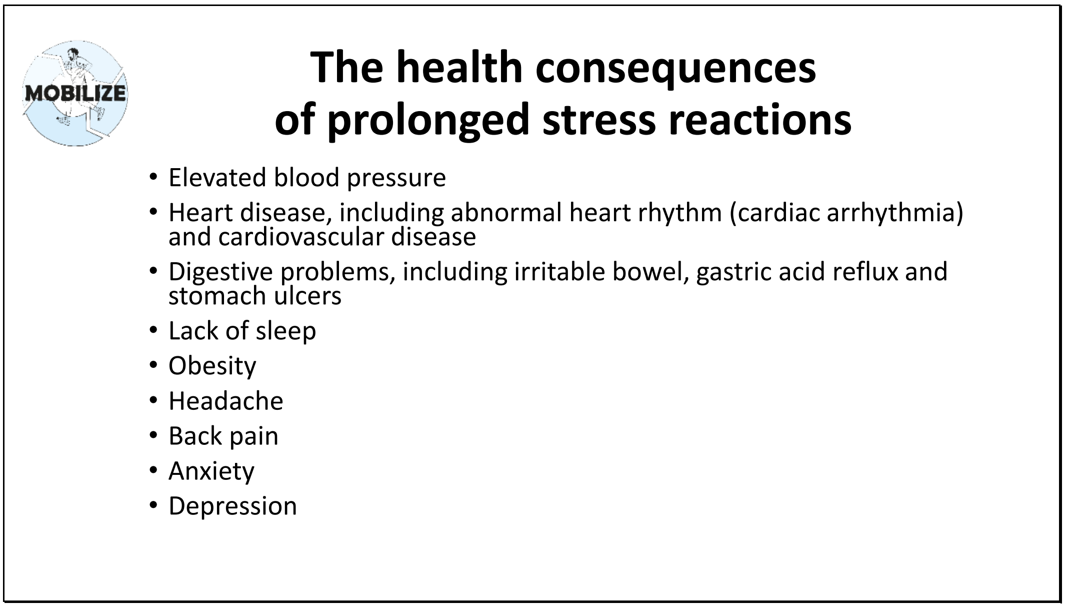

Introduction to slide: Prolonged stress reactions, which are after all a state of stress, are a burden on our body. In fact, more and more research show that stress affects our health.

End of slide: The problematic aspect of these stress responses is that they can both cause chronic illnesses and worsen a pre-existing chronic condition.

It can therefore be difficult for you to know if what you are feeling is a worsening of your chronic illness or a stress reaction.

It is always a good idea to get help and talk to a healthcare professional about your condition so that you can get the right help

Slide 8

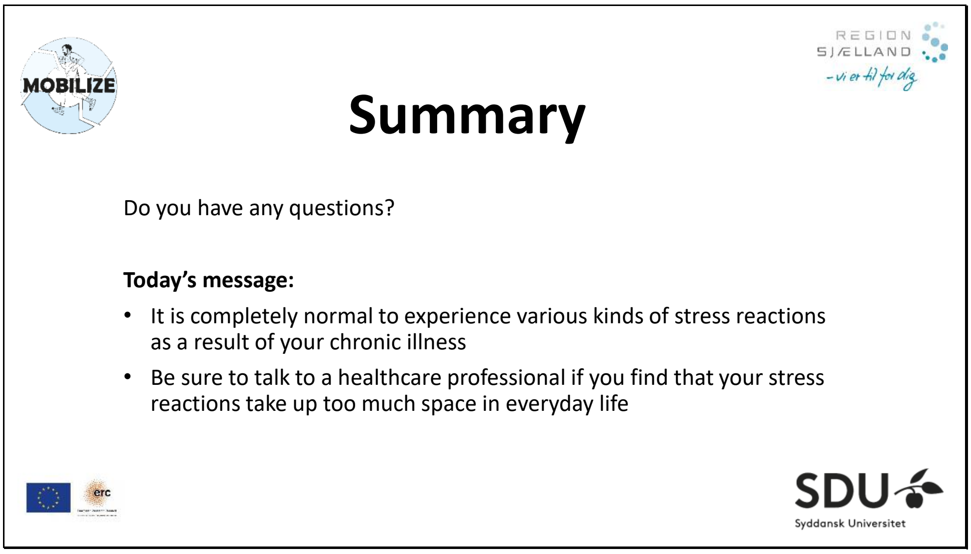

Answer any questions.

Breathing as a tool

Slide 1

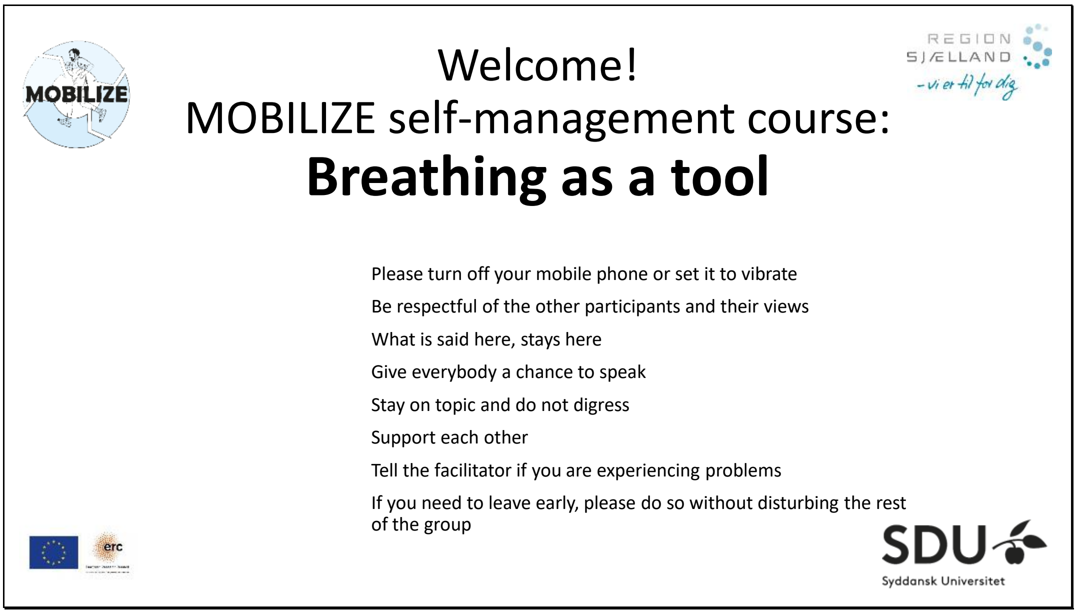

Welcome to the thematic module on breathing as a tool for managing general symptoms that may arise from your chronic illnesses.

No matter who you are and where you are in life, you can benefit from paying more attention to your breathing. You have probably heard the doctor or dentist tell you to take a deep breath before he or she anaesthetises you. Or seen that when footballers get ready for an important free kick, they always take a deep breath before they kick..

In today’s teaching session, we want to delve a little deeper into the mechanisms that influence our breathing – and vice versa.

Slide 2

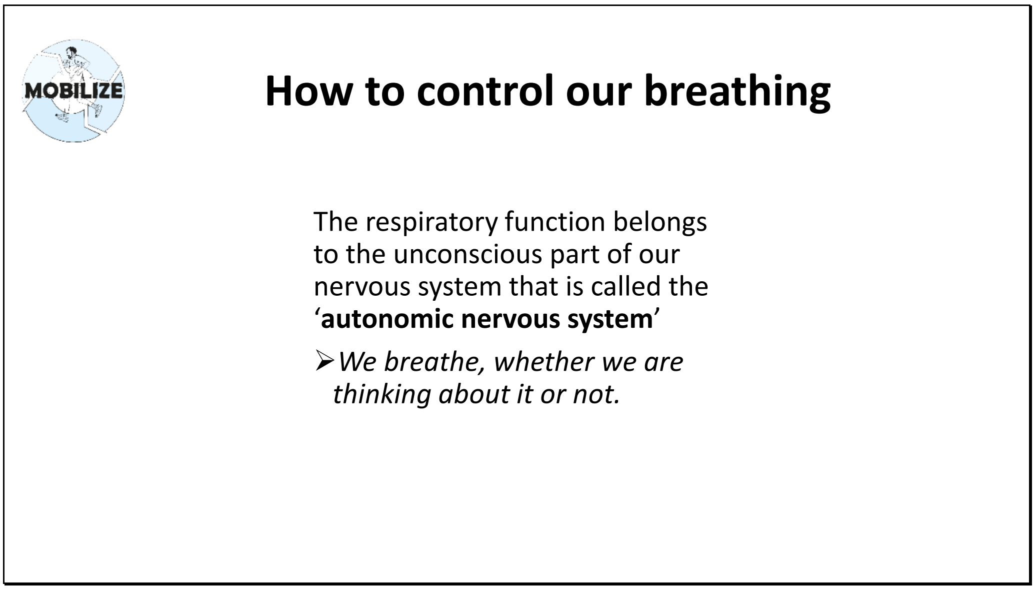

For example, very few people can hold their breath for more than a couple of minutes... Then the autonomic nervous system simply breathes for us – whether we like it or not.

Slide 3

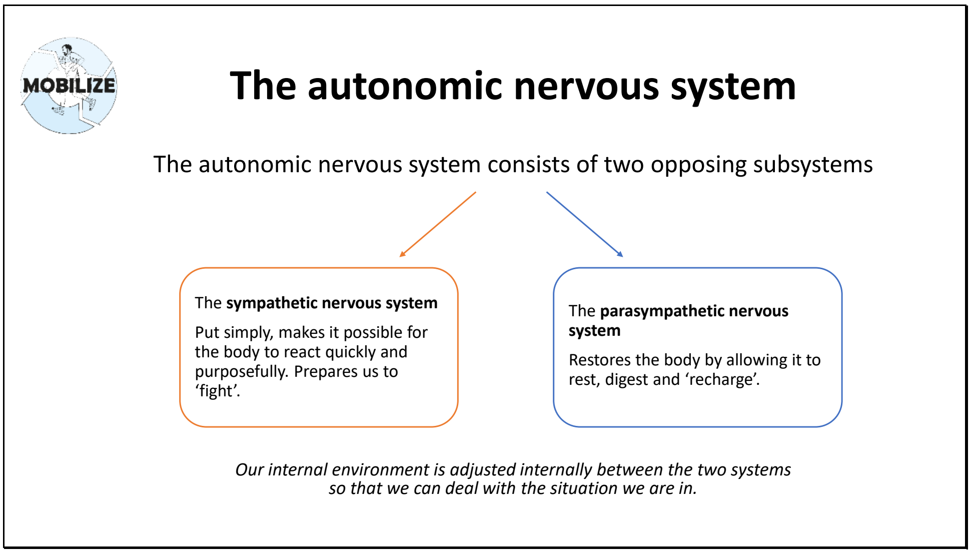

Introduction to slide: The autonomic nervous system involves everything we do not have conscious control over, such as the distribution of blood in the body, cold/heat regulation, digestion, heart rate and blood pressure.

Box 1) The sympathetic nervous system:
Ask the participants: What bodily reactions do you experience, for example, before you go to a job interview or to an important examination at the hospital?
For example, become nervous, heart rate increases, breathing becomes faster, begin to sweat, are very 'on', sharpened sense of sight and hearing...

Box 2) The parasympathetic nervous system:
Ask the participants: What bodily reactions do you experience when you have just eaten a big meal?
For example, become tired, relaxed, 'sluggish', heart rate drops, breathing is calm...

End of slide: For example, when you need to digest your food, a dominant parasympathetic system is needed – and if you subsequently need to solve a demanding task, a dominant sympathetic system is needed.
Imbalance in the autonomic nervous system with overactivity in the sympathetic branch can cause high blood pressure, stress and digestive problems.

Slide 4

MOBILIZE

The influence of breathing on the autonomic nervous system

- Breathing is the **only** part of our autonomic nervous system that we can actually influence ourselves!
- Conscious use of the breath with deep and calm breaths stimulates **increased** activity in the **parasympathetic** nervous system

Point 1) The autonomic nervous system influences and regulates breathing, but we can also use our breathing to influence the autonomic nervous system.

Point 2) If we become stressed, the breathing rhythm may increase on its own, even if we are sitting still. But when we take a deep breath, we actively reset the rhythm so that the breathing returns to a more natural level, the heart rate drops and we become more relaxed again.

Examples of how we CAN influence the parasympathetic nervous system using learned breathing techniques:

Danish freediver Stig Severinsen has trained himself to hold his breath underwater for 22 minutes.

The Dutchman Wim Hof, also called the Iceman, has developed a technique called the Wim Hof method, in which he is able to gain control of the autonomic nervous system using specific breathing techniques and cold training, which is reflected in his rather extreme exploits, such as when he

- sat for almost 2 hours with his whole body under ice (water temperature 1°C) **without** his core temperature dropping.
- Resisted an injection of an endotoxin, where the normal reaction would have been fever, tremor, nausea, etc.

- Climbed Kilimanjaro wearing only shorts.
- Ran a marathon in Namibia’s desert without water.

Slide 5

Breathing exercise: Wave

Begin in a seated position – the therapist will guide you

This exercise can be done in a lying or a sitting position.
If there are participants who prefer to lie down, they are welcome to do so.

The breathing exercise is guided by the therapist!

Exercise 1: Duration approx. 10 minutes.

In this exercise, imagine the breath as a 3-dimensional wave rolling in over the body on the inbreath and back out again on the outbreath.

- Sit back on the chair with your back relaxing against the backrest and place both hands on your stomach – one on the navel and one just below the navel. Sit comfortably with both feet on the floor. Relax your shoulders, face, jaw, tongue, throat and neck when doing the exercise.
- Take a deep breath in through your nose. Notice that the stomach naturally puffs out towards your hands. Continue breathing in and feel how the inhalation spreads further up to the ribs like a wave rolling over your body. Allow your hands to feel if anything is happening.
- On the exhale, just relax, let the air out **slowly without pressing,** so that the stomach naturally falls back into place.
- The exhalation may very well last longer than the inhalation.
- Repeat 5–7 times at your own pace.

During the exercise, say: The movement should feel like a wave moving through your body. Don't force the inbreath and make sure that the outbreath flows out slowly without forcing it.

Ask the participants: Could you feel the inhalation as a wave from your stomach up to your chest? Was it difficult – uncomfortable – nice? And what next?
COPD patients may have some shortness of breath afterwards.

Slide 6

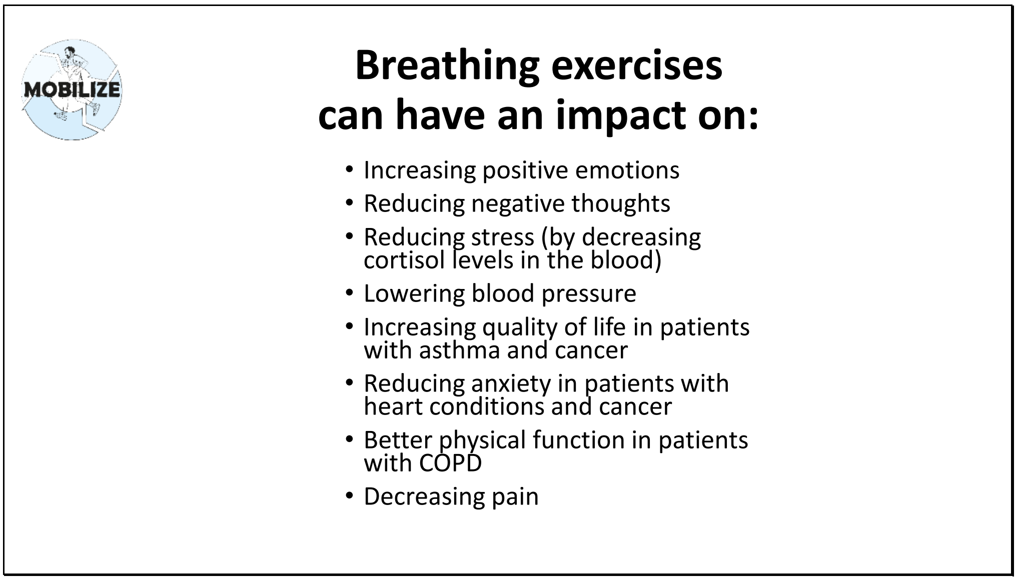

Introduction to slide: A great deal of research has been done on how we can affect our physical and mental well-being simply by working consciously with breathing.
Find the references in your patient handbook.

Slide 7

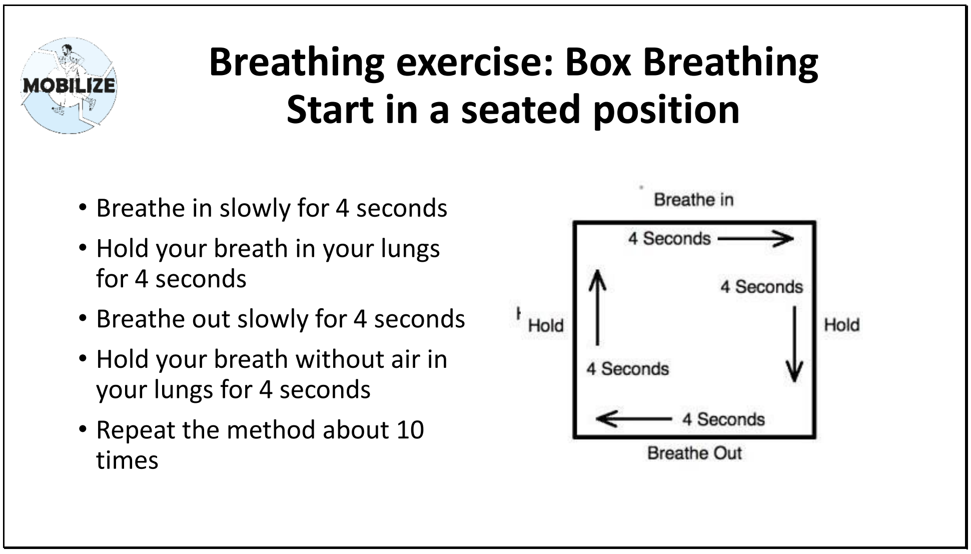

Introduction to slide: We will now test another type of breathing exercise, which is called 'box breathing'.
Box breathing can be done when you want to calm down. You can use it before going to bed or if you are a little nervous. You can also use it if you suffer from anxiety or stress.
If you felt sufficiently challenged in Exercise 1, 'the wave', then you can benefit from working a little more with it.

The breathing exercise is guided by the therapist!

Exercise 2: Duration 5 minutes.

In this exercise, imagine you are breathing inside a box, where you continuously breathe in a fixed rhythm, starting with a slow inhalation of 4 seconds, then hold your breath for 4 seconds before slowly exhaling for 4 seconds, and then hold your breath again with no air in your lungs for 4 seconds.

Sit back on the chair with both feet on the floor. Relax your shoulders, face, jaw, tongue, throat and neck when doing the exercise.

Are you ready...?

- Breathe in – 2 – 3 – 4
- Hold your breath – 2 – 3 – 4

- Breathe out – 2 – 3 – 4
- Hold your breath – 2 – 3 – 4

- Breathe in – 2 – 3 – 4
- Hold your breath – 2 – 3 – 4
- Breathe out – 2 – 3 – 4
- Hold your breath – 2 – 3 – 4

- Breathe in – 2 – 3 – 4
- Hold your breath – 2 – 3 – 4
- Breathe out – 2 – 3 – 4
- Hold your breath – 2 – 3 – 4

Repeat 4–5 times

Ask the participants: How does it feel? Was it difficult – uncomfortable – nice? And what next?

Slide 8

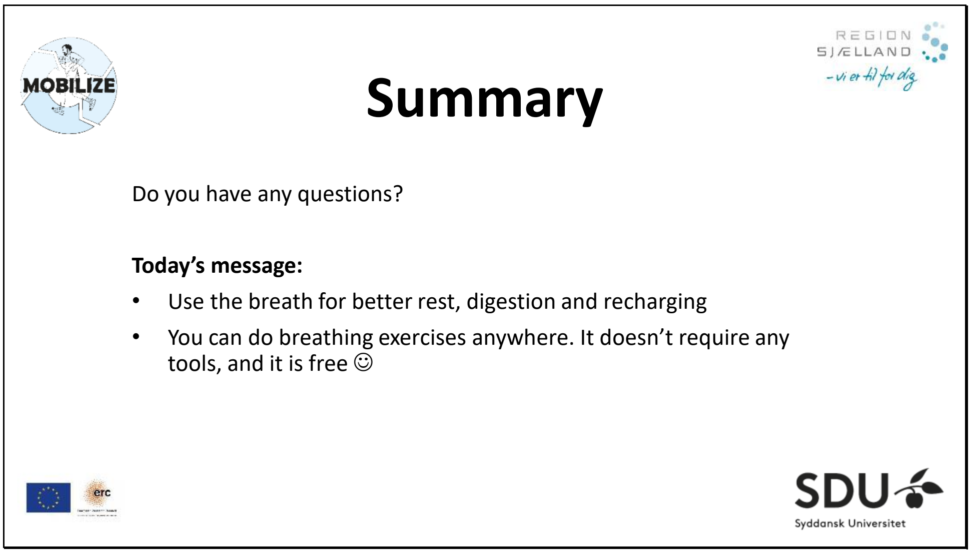

Answer any questions and remind participants to try to incorporate deep and calm breathing training into everyday situations and activities.

Once you have practiced the technique of good breathing and are able to do it at will, you can use it anywhere. For example, when you need to reduce pain in your body, are nervous, sad, in a bad mood, need to sleep, have headaches or other tensions, are stressed, your thoughts are racing, etc.

Even in a conversation with another person, at a meeting or in conflicts at home, breathing more consciously has an immediate effect.

Physical training

Slide 1

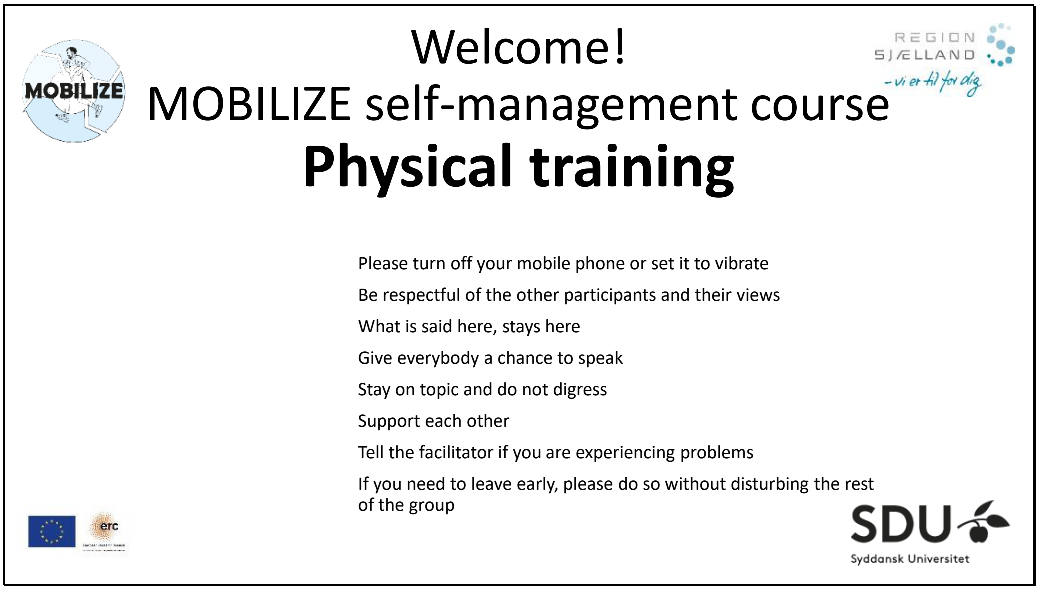

Welcome to the thematic module on physical training.

Today, we will take a closer look at the effects of strength and cardio training on the age-related changes our bodies undergo over time and why it's beneficial to exercise throughout your life.

Slide 2

Loss of muscle mass (sarcopenia)

Sarcopenia

- Between 5% and 13% of older people aged 60–70 years will have sarcopenia to a greater or lesser extent.
- For people over 80 years old, the proportion with sarcopenia has increased to somewhere between 11% and 50%.

Consequences:

- Getting up from a chair and climbing stairs will become more difficult. You walk more slowly, and your balance gets worse. The risk of falling increases.
- In more severe cases, even simple daily tasks such as getting dressed and undressed can become a problem.

Introduction to slide: Even in healthy older people, over time there is a gradual reduction in both the number of muscle fibres in the body and the size of the individual fibres, resulting in a loss of muscle mass, muscle strength and muscle power (the ability to produce high muscle force during rapid movements). In technical terms, this age-related loss of muscle mass is known as sarcopenia.

Slide 3

Strength training – why?

Training studies have shown:

- Increase in strength by 14% to 47% in people with chronic conditions.
- Overall, older +60-year-olds can achieve the same *relative* increase in muscle mass (10% to 45%) as younger people, typically after 8–12 weeks of regular strength training.
- Among the oldest (+85-year-olds), the relative strength gain can be even greater than in younger-older people.
- 75-year-olds who engage in strength training have maximal muscle power similar to that of 50-year-olds who do not engage in strength training.

Introduction to slide: Many people view the physical limitations ‘that come with age’ as a natural thing and simply accept the process. And while the process that leads to sarcopenia is inevitable with age, it is possible to slow down the process and the reduction of the body’s functionality. Research has shown that even as older people’s muscles decline, they are still responsive to growth signals.

Slide 4

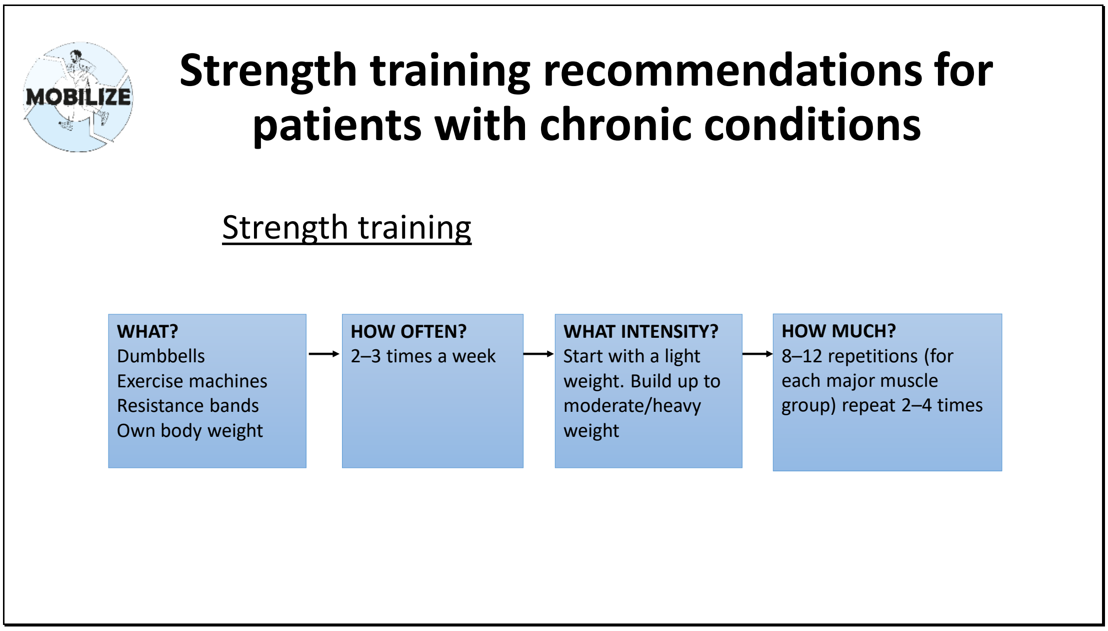

Introduction to slide: These are recommendations for strength training for people with high blood pressure, depression, diabetes, COPD, heart disease and osteoarthritis.
Ask participants to suggest what could be written in each box before clicking through.

Slide 5

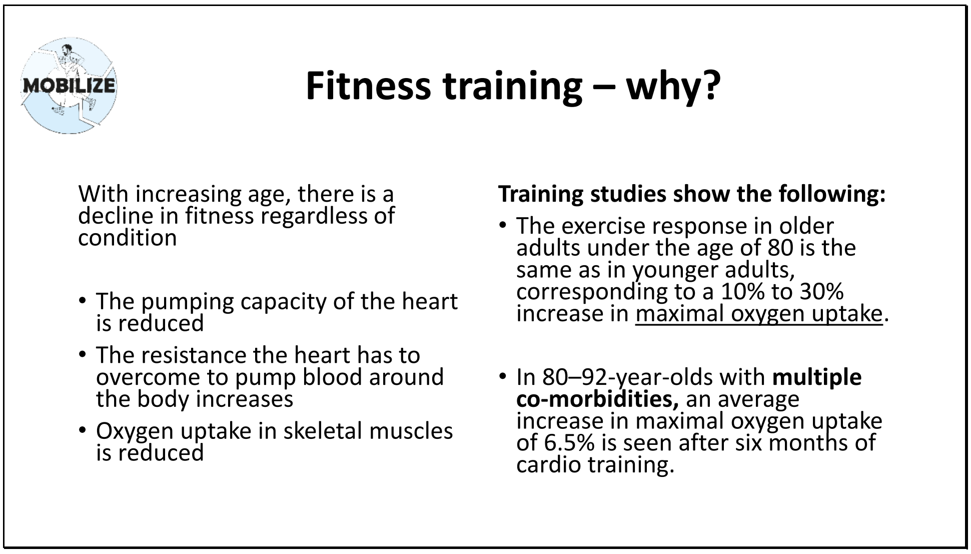

Point 1) Our maximal heart rate becomes lower and the heart's ability to contract is reduced.

Point 2) The diameter of the blood vessels is reduced, and the vessels become less elastic.

Point 3) Muscle mass and the number of small blood vessels are reduced.

Point 4) Maximal oxygen uptake is a measure of how much oxygen you can take in per minute. It gives an indication of how well your body is able to absorb, transport and utilise the oxygen you draw into your lungs.

End of slide: Ask the participants: Do you know the term 'fitness score'?
A fitness score is a commonly used measure of a person's level of fitness. The only difference between maximal oxygen uptake and fitness is that fitness also takes your weight into account. The bigger you are, the more oxygen you can transport. The fitness score is therefore based on oxygen uptake per kg body weight.

There are two ways to improve your fitness level: either by increasing your maximal oxygen uptake or by losing weight. A high oxygen uptake and low weight will therefore result in a high fitness score.

The age-related decline in fitness cannot be prevented by physical activity, but research shows that regardless of age, fitness levels are higher in those who are physically active than in those who are physically inactive.

Training intensity has an impact on the effect, and low-intensity cardio training leads to a significantly smaller increase in fitness than high-intensity training.

Slide 6

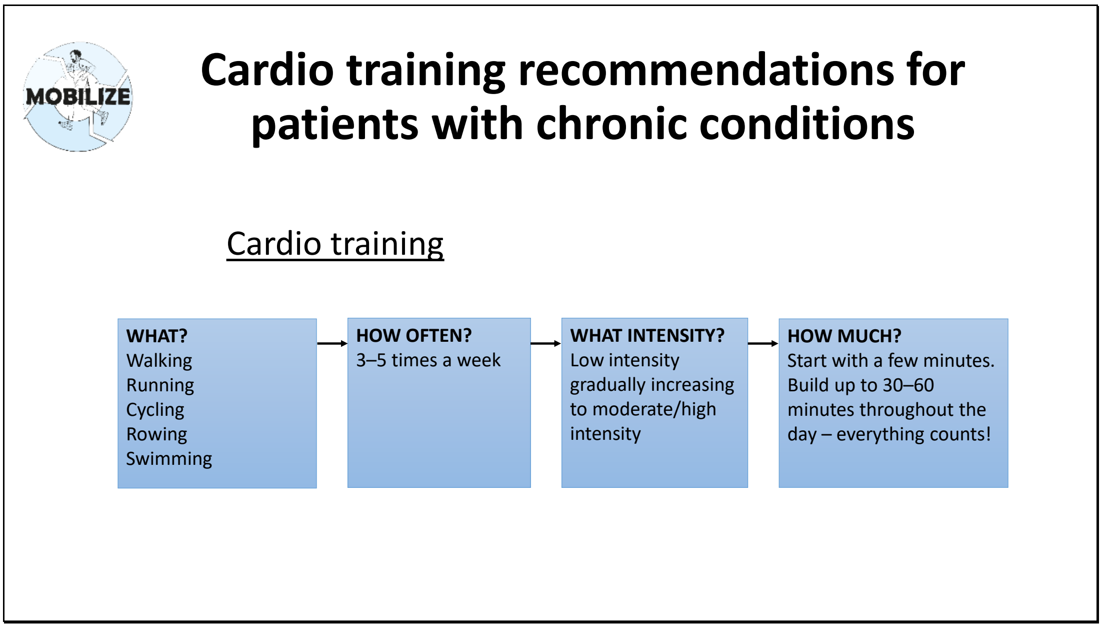

Introduction to slide: These are recommendations for cardio exercise for people with high blood pressure, depression, diabetes, COPD, heart disease and osteoarthritis.

Ask the participants to suggest what could be written in each box before clicking through.

Slide 7

Functional training

Functional training is based on 'everyday movements' such as pulling, lifting, pushing, throwing and walking to purposefully optimise and improve the body's natural movement patterns.

Functional training incorporates a mix of exercises:

- Endurance
- Strength
- Speed
- Balance
- Coordination

End of slide: Research studies have found that functional training can be a better approach than strength training if the goal is to improve the functional level of older people.

Ask the participants to come up with examples of exercises that can be related to functional training.

Examples of exercises:

Walk with a load → Carry shopping bags

Step-up exercise → walking on stairs

Lifting weight off the ground → lifting grandchildren

Get up/sit down exercise → get up from the chair

Avoiding obstacles → shopping

Slide 8

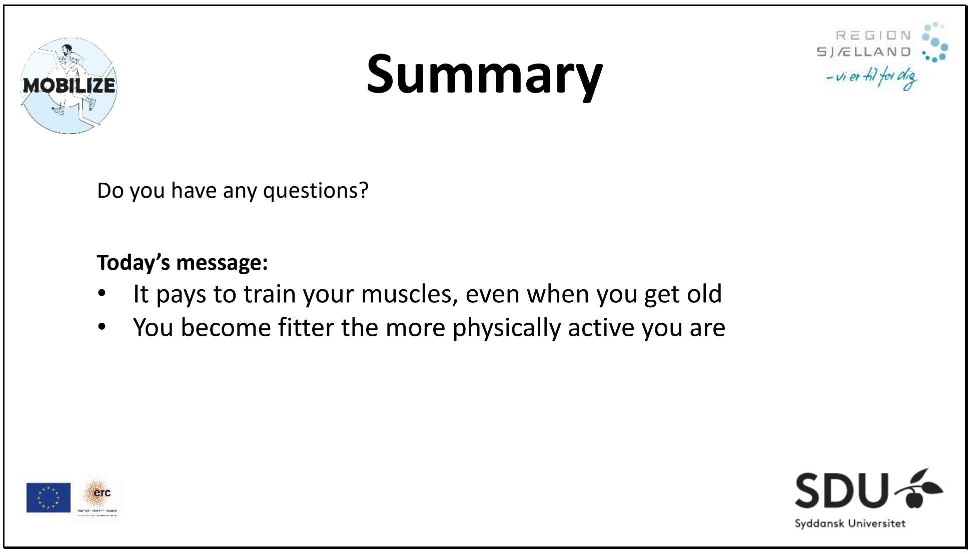

Answer any questions.

Point 1) Even though older people's muscles are atrophying, they are still responsive to growth signals.

Point 2) Regardless of age, those who are physically active have a higher level of fitness than those who are physically inactive.

Mindfulness: body scan

Slide 1

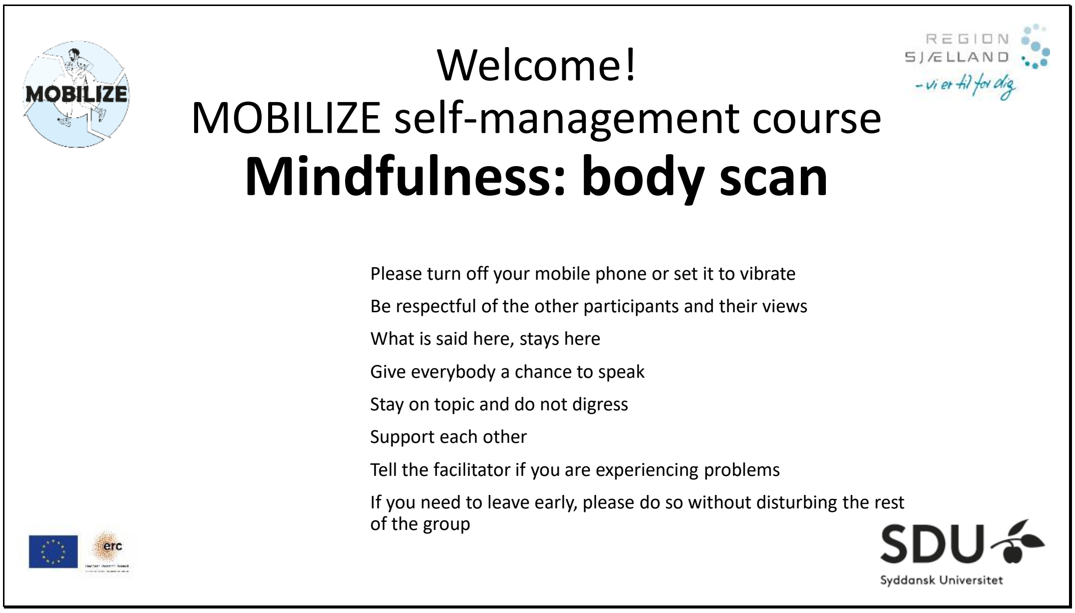

If you have pillows, blankets, mats or blocks available, these can be offered to participants during the body scan.

Welcome to the thematic module on body scanning.

Today, we will try out a longer mindfulness exercise – a so-called body scan lasting approx. 15 minutes.

Mindfulness, like physical training of the body, can be a good and relevant tool for coping with chronic illness.

This is important to remember:
Mindfulness is training for your mind and mental state, and just like training your body it requires a lot of practice and consistency to get the most out of it. In addition, just like with physical training, sometimes it goes very well and other times it can be difficult to feel the effect. The same is true when working with mindfulness.

Slide 2

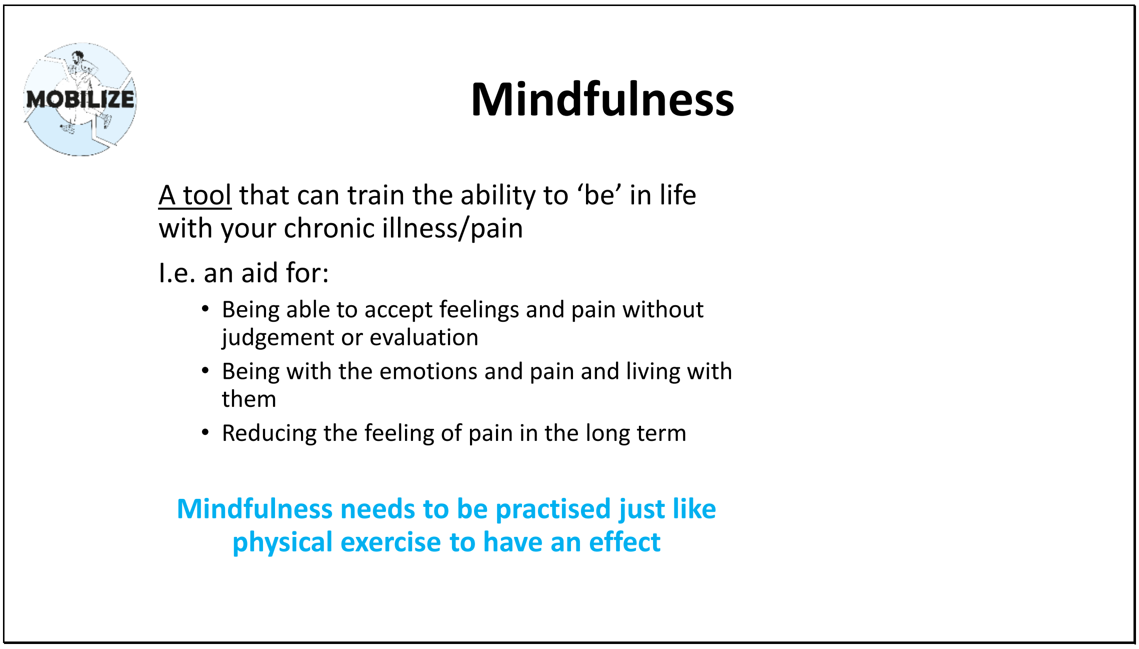

End of slide: When you relate to how you feel right now – not how you should feel or how you wish you felt – you are being mindful!

This allows you to become consciously present from moment to moment. By observing your thoughts, feelings and pain, rather than reacting to them and starting to act on them, you can let go of the past, the future and the desire for things to be different than they are.

This shift in attitude – welcoming whatever comes instead of fighting or endeavouring to remove it – creates a change in how you perceive and experience the present moment.

Mindfulness differs from psychotherapy by acting directly on the areas of the brain that regulate our emotions. It inhibits activity in these areas, making us think more objectively and clearly.

Slide 3

Body scan

During a body scan, focus your attention on each part of your body with an open mind – without judgement or trying to change anything.

If you choose to do the exercise lying down, place a pillow under your knees to keep you comfortable. You can also sit in a chair.

Take a deep breath and shift your attention to the area you're focusing on.

Be curious. What does each sensation actually feel like? You are interested in experiencing every sensation in its pure form, as it really is.

Slide 4

Body scan

You will now be guided through a body scan

Have the participants sit or lie down and relax as much as possible. Offer extra cushions or a couch if available/needed. For the next 15 minutes guide the participants:
Begin by making yourself comfortable. Take several long, slow, deep breaths. Begin to let go of noises around you. Begin to shift your attention from outside to inside yourself. If you are distracted by sounds in the room, simply notice this and bring your focus back to your breathing. Now slowly bring your attention down to your feet. Begin observing sensations in your feet. When you are ready, allow your feet to dissolve in your mind's eye and move your attention up to your ankles, calves, knees and thighs. Observe the sensations you are experiencing throughout your legs.
Then guide participants to the back and pelvis + mid and upper back + chest + shoulders + arms and hands + scalp, head and face and finally:
And now, let your attention to expand out to include the entire body as a whole. Bring into your awareness the top of your head down to the bottom of your toes. Feel the gentle rhythm of the breath as it moves through the body. Now, open your eyes and return your attention to the present moment.

After the body scan, ask the participants: Would you like to share your body scan experience?

Slide 5

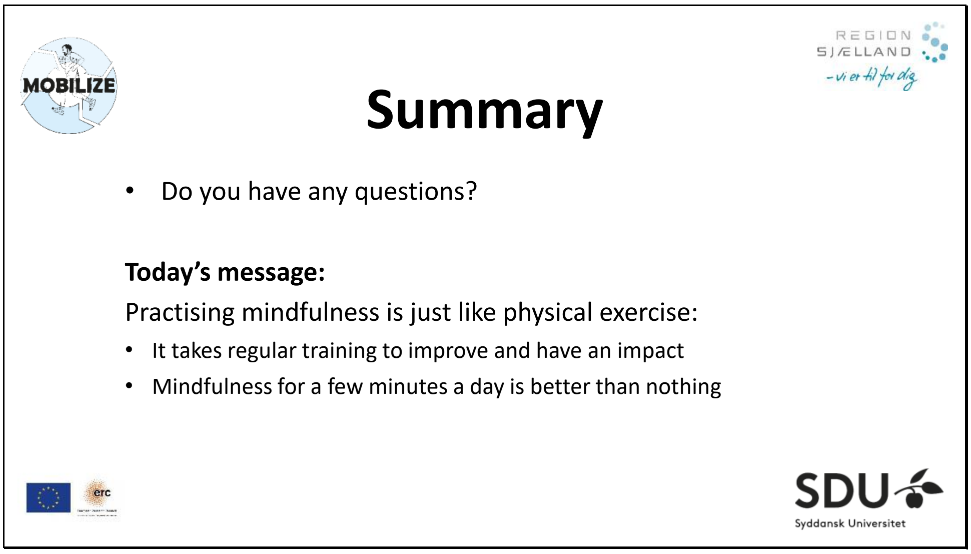

Answer any questions.

Remind participants that it’s OK if they can’t complete a full body scan every time. Shorter sessions of 5–10 minutes are also good.

General mental health symptoms

Slide 1

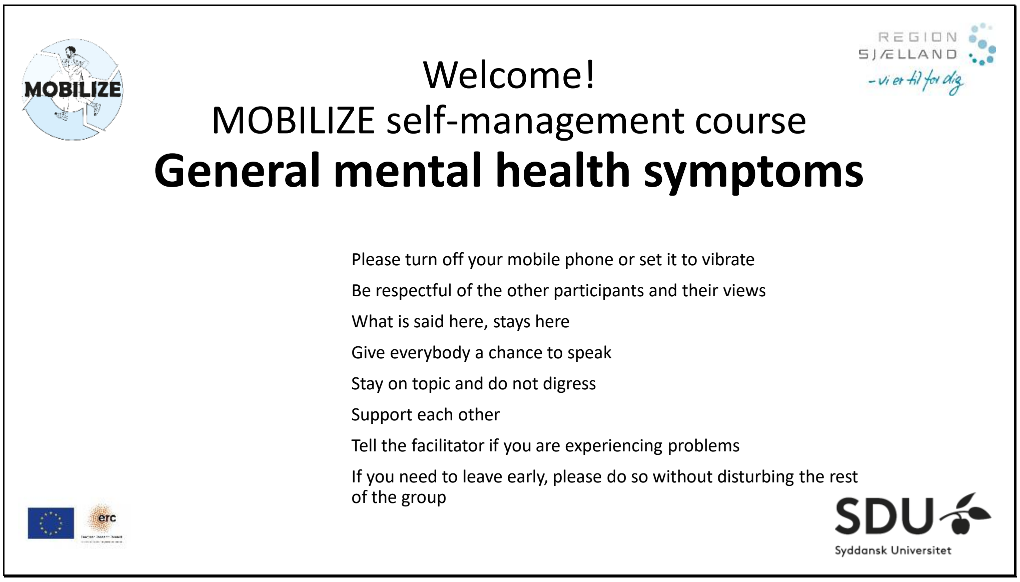

Welcome to the thematic module on general mental health symptoms.

Today, we will go through some of the common mental health symptoms that can arise from chronic illness and provide an introduction to what you can do to manage them.

Part of living well with a chronic condition is learning to manage the changes and symptoms that can occur, either because of the condition itself or as a result of the way you react to it.

Slide 2

MOBILIZE

Fatigue

- Fatigue is a specific type of tiredness characterised by a lack of energy, exhaustion or tiredness
- Chronic illness can drain your energy

Point 1) Fatigue does not disappear with rest or sleep.

Point 2) *That is why fatigue is a big problem for many people.* Fatigue can stop you from doing what you want to do.

To manage fatigue, you must first understand that it can have various causes: the illness itself, inactivity, poor nutrition, insufficient rest, emotions, medication.

Slide 3

Managing tiredness/fatigue

- Talk to others about your fatigue
- Prioritise your day so you have energy for the activities that are most important to you
- Find a good balance between activity and rest

Introduction to slide: Ask the participants how they deal with tiredness/fatigue before clicking through to the bullet points. Are you trying to ignore it? Giving in to it and trying to sleep it off? Do you let it limit you?

Point 1) Be open about fatigue. It is important to tell your loved ones about your fatigue and how fatigue differs from regular tiredness.
Fatigue affects most people's mood, so being open about how you are feeling can clear up some misconceptions.
Talk to your doctor, as it can also be caused by other conditions.

Point 2) You should also remember to prioritise social interaction, as it positively benefits both mood and pain – and remember that you do not have to be full of energy to spend time with your loved ones.

Point 3) Both your body and your mind will recover more quickly if you don't keep going until you are completely exhausted before resting or taking a break. As you find your balance, you can increase periods of activity and shorten periods of rest.

Slide 4

Stress

Stress is the body's response to an imbalance between the number of demands facing us and our ability to meet those demands

Common stress reactions:
- Physical
- Emotional

Paragraph 1) The causes of stress can vary, and there can be differences between what stresses different people. Stress is often the result of being burdened by several of these factors at the same time – with no sense that you can change the stressful circumstances or do anything about what is affecting you.

Paragraph 2)
Point 1) physical in the form of a faster heartbeat, increased blood pressure, neck and shoulder muscle tension, faster breathing, slowed digestion, dry mouth.
Point 2) mental in the form of memory and concentration problems, irritability, sadness.

Slide 5

Managing stress

- Identify what triggers your stress
- Be realistic
- Stop before you react
- Be proactive with problems
- Choose your battles
- Give yourself permission to say **NO!**

Introduction to slide: Ask the participants how they deal with stress before clicking through to the bullet points. Are you trying to make to-do lists, 'pull the plug' or run even faster...?

Point 1) Are there patterns in what triggers your stress? For example, fear of change or the unknown, family life or relationship problems? Is there anything you can avoid or change? Are there areas where you create your own stress, such as unrealistic expectations, too little or too much time, a negative attitude or way of seeing things?

Point 2) Drop the preconceived ideas of what you 'should' be doing. If you find yourself overwhelmed with too many things to do, prioritise and see what you can cross off the list. Ask for help when you need it.

Point 3) Take a deep breath and ask yourself how important it really is. Will it have a big impact and still be important tomorrow or in a few weeks? Is it even worth responding to or should you just let it be?

Point 4) Avoid procrastination. When you have to deal with the things you find unpleasant, you will only prolong your stress by putting things off.

Point 5) Avoid worrying about the little things. Not only does this mean fewer potential stressors, but it also means that when there is something worth fighting for, it can get the attention it requires.

Point 6) Knowing your limitations and then exceeding them anyway does not make you brave and is not admirable. It is just self-defeating. Why would you choose to take on more responsibility than you can handle if you know it is detrimental to your own health? Remember, every time you say no to something, you are saying yes to taking care of yourself and having time for the more valuable things in life.

End of slide: Chronic illness can be a trigger for stress, but stress can also make the illness worse. That is why it is important to manage your stress. It is not something you learn once and for all, but something you need to practise daily.

Slide 6

Low mood and depression

- Low mood is the gloomy feeling that most of us experience from time to time
- Depression is used only when referring to the medical diagnosis

Many emotions can lead to low mood

Point 1) Feeling low is a normal reaction to adversity.

Point 2) Low mood and depression are often mentioned interchangeably, but they are two different conditions.

Signs of depression: lack of interest in friends and activities, difficulty sleeping, changes in eating habits, unintentional weight change, loss of interest in personal care and style, suicidal thoughts, frequent accidents, low self-esteem, loss of energy.

End of slide: For example, fear, anxiety and uncertainty about the future, frustration and loss of control over your life.

Slide 7

MOBILIZE

Handling of low mood and depression

- Be physically active every day
- Be social
- Volunteer with an organisation
- Make a list of things you can reward yourself with
- Seek professional help

Introduction to slide: Ask the participants how they deal with depression before clicking through to the bullet points. Do you get out and get fresh air/move your body? Do you contact someone? Are you good to yourself?

Here are some things you can do:
Point 1) It doesn’t have to be for a long time every time. Less can also be good if it is done daily. It can be anything from gardening or going for a walk to playing with grandchildren.

Point 2) Do something together with friends, family. For example, a walk, a coffee, a shopping trip, a few days away from everyday life.

Point 3) People who help others are less depressed.

Point 4) It is a way of caring for yourself.

Point 5) Seeking professional help is not a sign of weakness, it is a sign of strength. Counselling therapy, possibly combined with medical treatment, can often provide relief.

Slide 8

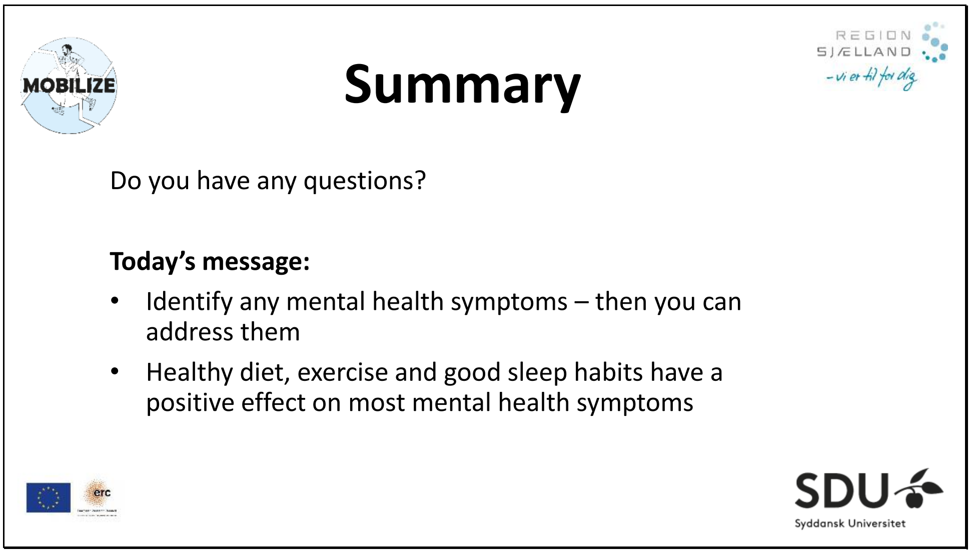

Answer any questions.

Remind participants that they can seek knowledge from healthcare professionals and patient organisations for help with symptoms.

Coping strategies

Slide 1

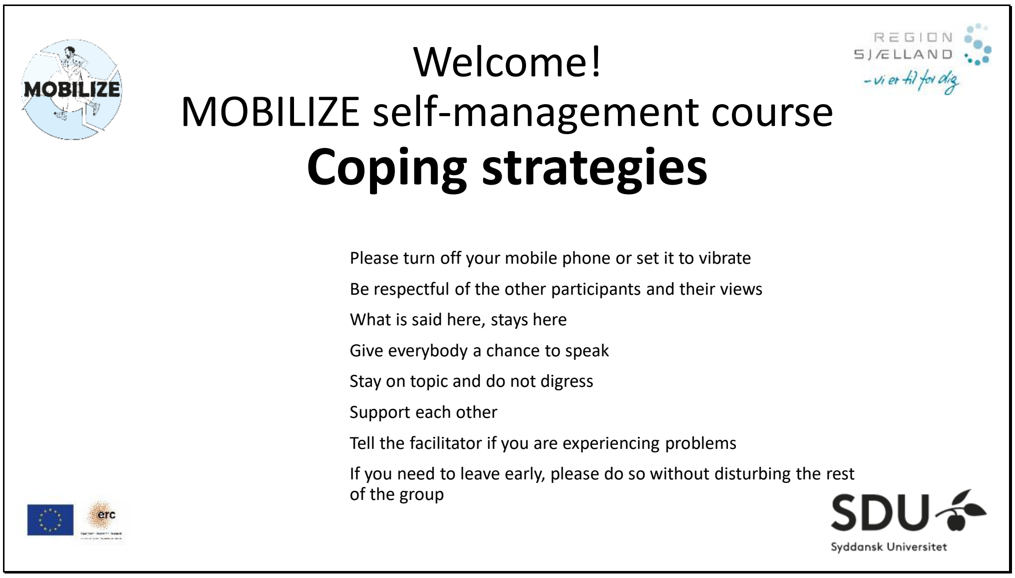

Welcome to the thematic module on coping and coping strategies.

Mastering a situation or conditions can also be referred to as ‘coping’ with a situation or conditions. Today, we will take a closer look at what strategies you can use when life presents you with challenges that can be difficult to deal with.

Slide 2

What does 'coping' mean?

Constantly changing cognitive and behavioural attempts to cope with specific external and/or internal demands that are deemed challenging or beyond the resources available to the person.

End of slide:
When our important values or goals are challenged, threatened or destroyed, we experience psychological stress. People will try to counter, avoid or cope with this stressful situation in different ways.

The purpose of coping strategies is:

- Skills and behaviours we use to create positive meaning in negative situations
- Help us deal with the emotions that arise when life is difficult
- Help us to develop as people
- Can be both emotional and problem-orientated

Slide 3

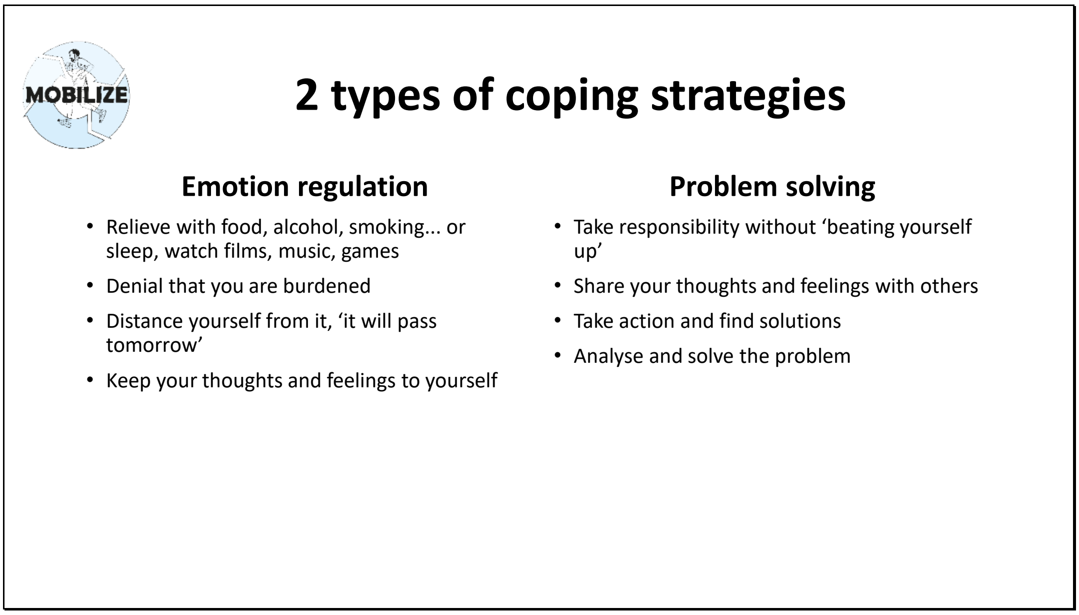

Introduction to slide: To master/cope with a crisis or stressful situation, we develop behaviours and strategies in two main ways: emotion regulation and problem solving.
Often, both are used.

Emotion regulation: Focuses on regulating emotions and discomfort and shifting the focus of thoughts or seeking comfort/relief. May provide short-term peace and relief, but in the long run it leads to additional strain and stress in relation to your chronic illness situation.

Problem solving: Focuses on solving or processing problems, increasing options, seeking information or confrontation. These strategies work in the long run and are good for both your mental and physical well-being. However, they often require more effort on your part.

Both strategy types can include strategies that focus on avoidance, where you either remove challenges, remove yourself from the situation or withdraw physically, mentally or emotionally.

Slide 4

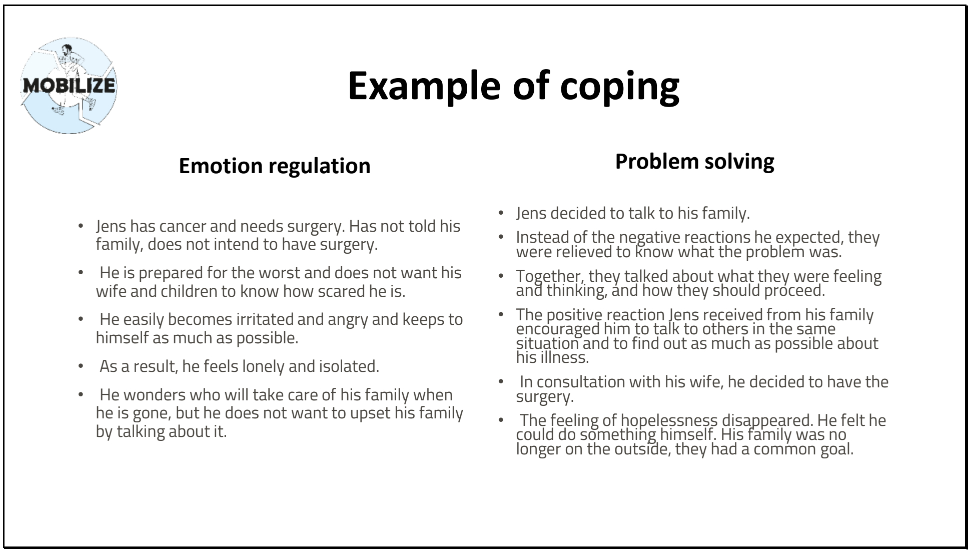

<u>Emotion regulation:</u>
Introduction: We try to calm inner turmoil, our feelings of guilt or anxiety. We enter a mental process, avoid taking action or deny/avoid the situation.
The following is an example of this: **(Click further)**

After points: The coping strategy Jens uses is to minimise discomfort, avoid taking action. Avoiding his family results in a feeling of loneliness and isolation. By preparing for the worst and keeping his thoughts to himself, he is passive and resigned, leaving everything to 'fate'. This only adds to his sense of hopelessness, which rubs off on his family. However, his wife and children have reacted to how much he has changed. They feel alone and do not know what to do.

<u>Problem solving:</u>
Introduction: We are trying to find the cause of what has happened. We will look at what we can do or can get others to do. We seek knowledge and information. We take action.
The following is an example of this: **(Click further)**

After points: The coping strategy Jens now uses is to be active and participate. He took control of the situation and talked about his feelings. His wife and children could also participate and talk about their feelings. They could all support each other.

Slide 5

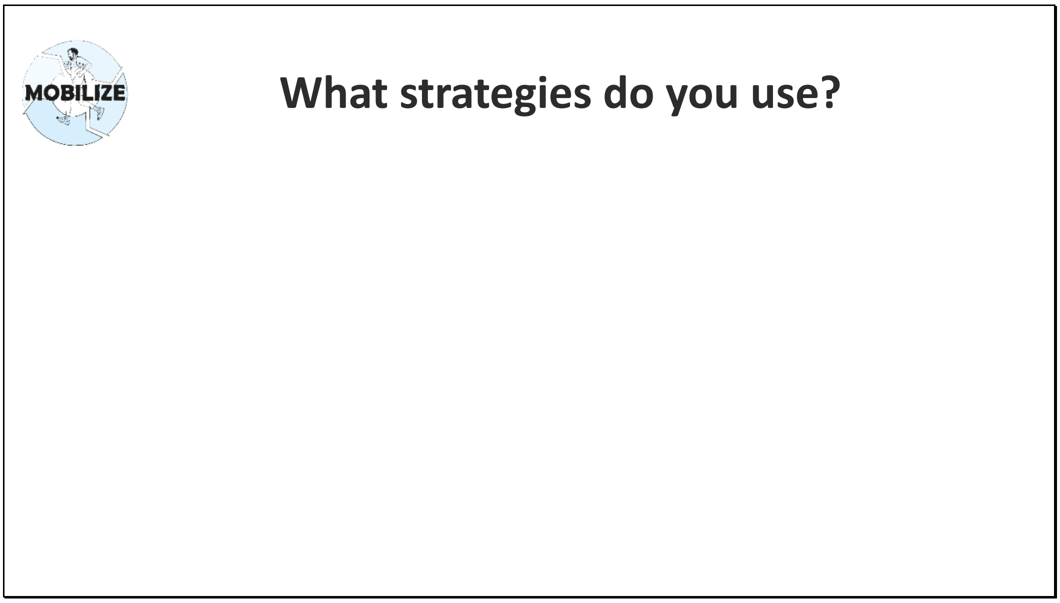

Set up chats in pairs: How do you manage the stresses of living with chronic illness in your daily life? (Are you more orientated towards solving problems or do you opt for emotion-orientated solutions? Maybe a bit of both?)

Spend 5 minutes each talking to each other about how you manage the stresses of living with chronic illness in your daily life. It is important that only one person at a time is talking and one is listening.

In the event of an odd number, the teacher steps in and is the listening party. The participant can then go to another group and become a listener. (Duration: 5 minutes each).

Plenary wrap-up (Duration: 5 minutes). How was it to share your strategies/solutions? Did you become more aware of your strategies? Was it inspiring?

Slide 6

MOBILIZE

Toolkit for healthy coping strategies

- Talk to a friend – many people find this helpful
- Exercise
- Keep a diary
- Creative activities such as painting, singing, dancing, cooking
- Take a day off
- Take 10 minutes to write down what is good and meaningful
- Do something that makes you laugh
- Do something special for someone else
- Get some small things done
- Listen to music

End of slide:
It is important to remember that appropriate coping strategies can be both problem-solving (talking to someone, seeking knowledge and information) and emotion-regulating (games, films, relaxing, laughing, listening to music).

The important thing is not to rely too much on relieving strategies such as food, alcohol or denial.

Slide 7

Relaxation techniques

Sometimes you may need to take a break to rest your body and mind.

There are physical techniques such as breathing exercises or muscle relaxation/relaxation exercises.

There are also mental exercises such as mindfulness and meditation.

Mindfulness is a great tool if you tend to get stressed easily. It is something that needs to be learned and practiced.

Relaxation techniques are also a tool you can use to help you cope with your situation. You have or will try this out in other modules.

Slide 8

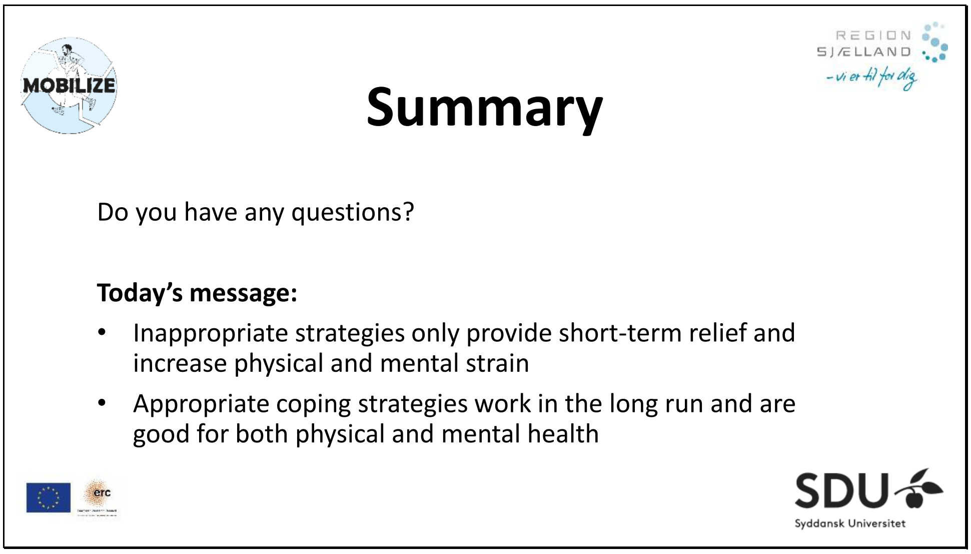

Answer any questions and remind participants to become aware of their coping strategies.

This is the first step of changing to more appropriate ways of coping with their situation.

Finally, remind the participants that they are welcome to bring their carers to the next module on Goal Setting.

Goal setting

Slide 1

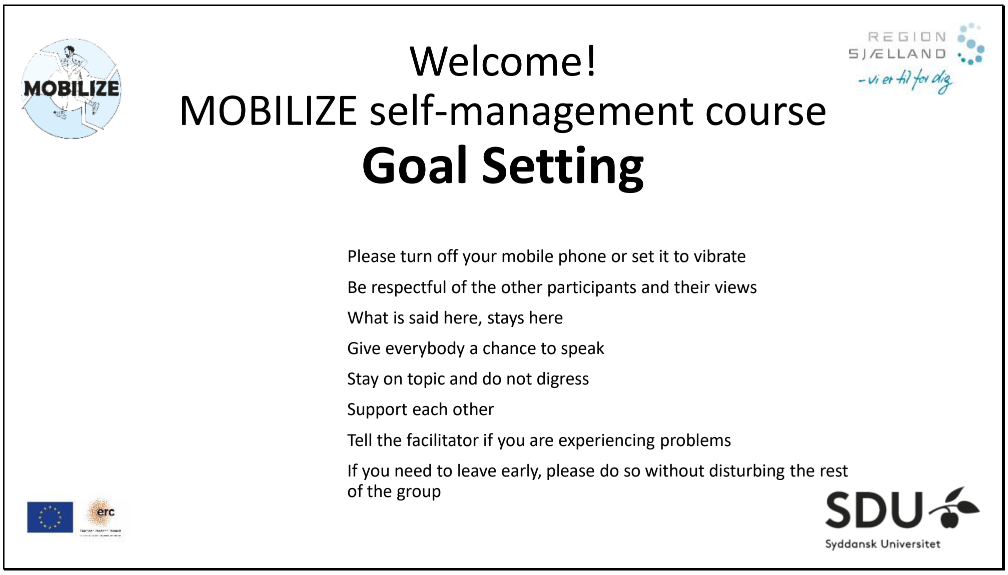

This is one of the modules where MOBILIZE recommends that carers are invited to participate, as they can support the participant in achieving their goal.

Participants' goal charts and pens are required for this module.

Welcome to the thematic module on goal setting.

Today's theme is all about goal setting.
We will talk about motivation and its importance for your goal setting.
We will look at your goal charts and talk about how to achieve your goals.

Slide 2

Motivation

Motivation can be defined as...

the sum total of the <u>internal</u> and <u>external</u> forces that influence us to make a certain choice

Remember: motivation is dynamic

Introduction to slide: First, let’s talk about motivation, as it is an important factor in achieving your goals in relation to physical activity. The likelihood of you doing what you set out to do – i.e. performing an action – depends on how motivated you are and how easy it is for you to do.

<u>Inner forces can include thoughts, emotions and physical resources:</u>
Thoughts: Our thoughts can help or hinder our motivation. For example, motivation is enhanced if it is meaningful and important. Conversely, thoughts can also ‘allow’ us to not be motivated to do physical activity by constantly postponing or making excuses for ourselves.
Emotions: The more we can associate physical activity and healthy eating with being positive, joyful and relationship-building, the easier it will be to change habits.
Physical resources: The ability to make the right choices is dependent on how we feel physically. The energy to change habits can be far less for people with a chronic illness than for healthy people. It is important to match lifestyle demands with energy levels. Avoid falling into the trap of setting your ambitions too high based on ‘what you could achieve in the past’ but respect your current energy level and start with short but frequent walks, for example.

<u>External forces can be culture, physical surroundings, relationships:</u>
Culture: Our culture rubs off on our lifestyle. For example, our perception of belonging and politeness affects whether we should say yes or no to cake at the office.
Physical environment: We know that it is important for our motivation that our environment is designed to make it easy to do the right thing. There are many ways to adjust the environment to

help you make the right choice. Cake at the office can be replaced with fruit, the office chair with an exercise ball and the exercise bike can be placed in front of the TV next to the sofa.

Relationships: The people we spend the most time with can either inspire us to live healthily or lead us astray. It is not just your family that needs to support a lifestyle change, but also your neighbours, colleagues and many others who suddenly have to get used to the fact that vegetables and not just sausages are on the barbecue or that you make time for exercise in your daily life. It can be a challenge. It can be helpful and motivating to have a friend to make appointments with, for example, to exercise or go for walks. You are more likely to show up when you have made an appointment.

Slide 3

MOBILIZE

Your goal chart

Is your **SMART** goal still smart?
Look at the goal chart you filled out at the start of the programme.

Introduction to slide: Hand out the participants' personalised goal charts from the individual introduction.
Now we will talk about your goals. In this context, it is important to remember that knowing what you should or should not do does not alone lead to action.
Remember that if healthy habits are linked to your personal values, your motivation will be greater than if they are linked to health improvements alone.

Ask participants to look at their goals again and think about whether their plan is aligned with the SMART goals below: (Duration max. 5 min.)

Specific: What do you want to achieve?
Measurable: How will you measure whether you have reached your goal?
Attractive: Why is it important to achieve this goal?
Realistic: Can it be done?
Timeline: When is your deadline?

Slide 4

Tips to achieve your goal

- Set milestones on the way to the final goal
- Evaluate your goal
- Get back on track if you have lost motivation

In the next slides, you will find tips and advice on how to achieve your personal goals.

Slide 5

Milestones

If your final goal is a long way off, you may want to set interim goals, or milestones

- Split the period into a number of sub-periods
- Decide how much you want to have achieved at certain points in time

Milestones will allow you to stop, evaluate and adjust either the goal or the method.

Points 1–2) The idea is that your milestones build on top of each other like a staircase, so they constantly bring you one step closer to your end goal.
<u>Example of milestones</u>:
If the goal is to be able to walk up all the stairs to your third floor flat instead of taking the lift, you could set a milestone of being able to walk to the first floor after one month, the second floor after two months and the third floor after three months.

End of slide: Use milestones to test whether you are on the right track or need to make adjustments. Is the goal too ambitious, have you chosen the right activity for your goal?
They can also be used to boost your morale. Every time you reach a milestone, it is a small victory.

Ask the participants: Do you need to break down your goal into smaller sub-goals (milestones)?
Talk in plenary about possible milestones based on the example above (Duration: max. 5 minutes).

Slide 6

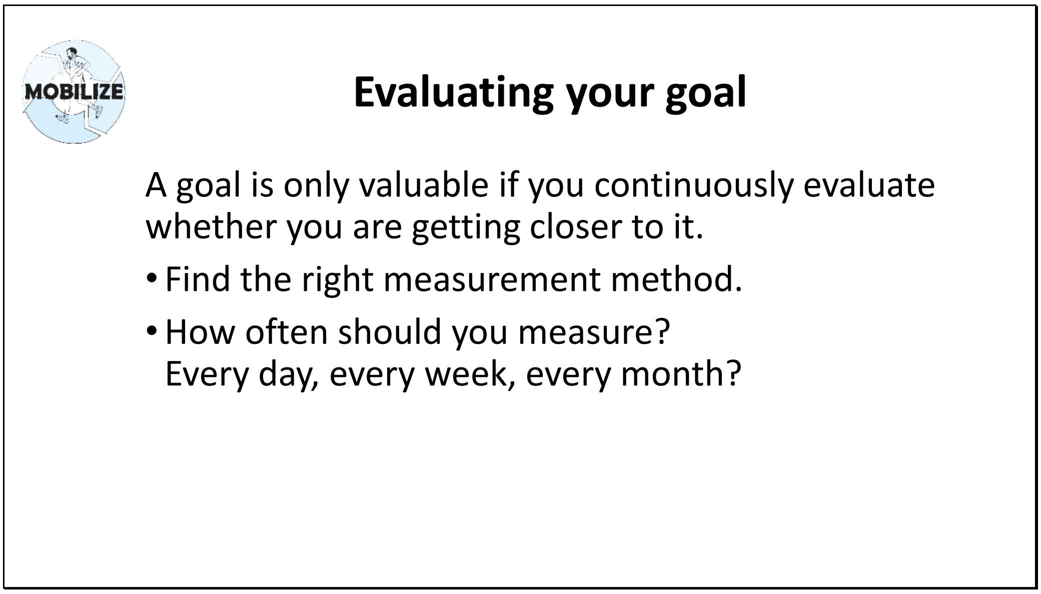

Point 2) It is all about choosing the best method to evaluate your goal.
If you want to lose weight around your stomach, measure around your stomach. If you want to be able to walk 10,000 steps a day, you need to count steps.

Point 3)
Benefit Using frequent measurements, you can quickly make adjustments
Disadvantage Using frequent measurements, there is a risk that the changes are very small, so it can be challenging to assess whether there is progress or decline.

Ask participants how they measure their specific MOBILIZE goals (which measurement methods and how often) (Duration: max. 5 minutes).

Slide 7

Relapse... back on track

You are not starting over....

You just have a new starting point!

That is how you are most likely to succeed
if you make your training a habit.
Start with small steps.

Introduction to slide: It is perfectly normal for motivation to wane along the way – there can be a myriad of reasons for this. The key here is not to give up, but to get back on track.
Be honest with yourself. Not because you need to beat yourself up or feel guilty, but because you will learn from it.

Paragraph 1) It may feel like you are starting over, but you are not. You have learned from your past victories and defeats and are more aware of your strengths and weaknesses. You know more about yourself now and what to be prepared for.

Paragraph 2) A habit is a behaviour that has been repeated so often that it has become automatic, and you no longer think about it. It would be easiest for you to live with exercise, for example, if it became a habit... an integral part of your everyday life. It is difficult to say how long you need to persevere before it becomes a habit, but studies suggest that you should do it regularly for at least three months.

End of slide: Lasting habit changes are best made in small steps. Instead of planning a difficult habit that you can only accomplish during the most optimal periods of your life, plan an easy habit that you can easily accomplish during all periods of your life. In other words: make your training so manageable that you can't say no.

Remember: it is all about getting started again! Once you get started, you may find yourself doing more than you planned. The best training sessions are the ones that actually get done!

Ask the participants: Have any of you experienced a loss of motivation but found a way to get back on track, and what did you do to get back on track? **(Duration: max. 5 minutes).**

Slide 8

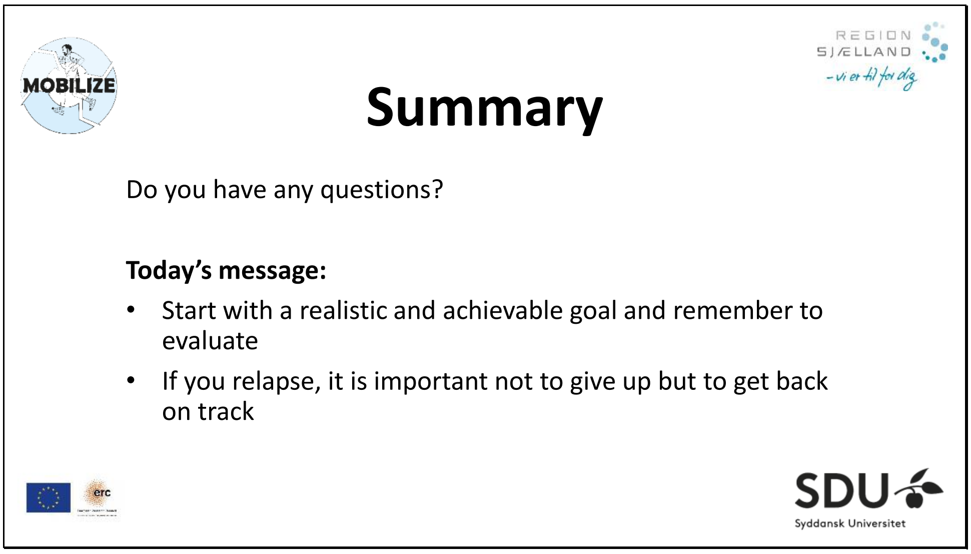

Answer any questions.

Ask participants to take their (adjusted) goal charts home and remind them to follow up on the charts regularly.

Breaking down barriers to physical activity

Slide 1

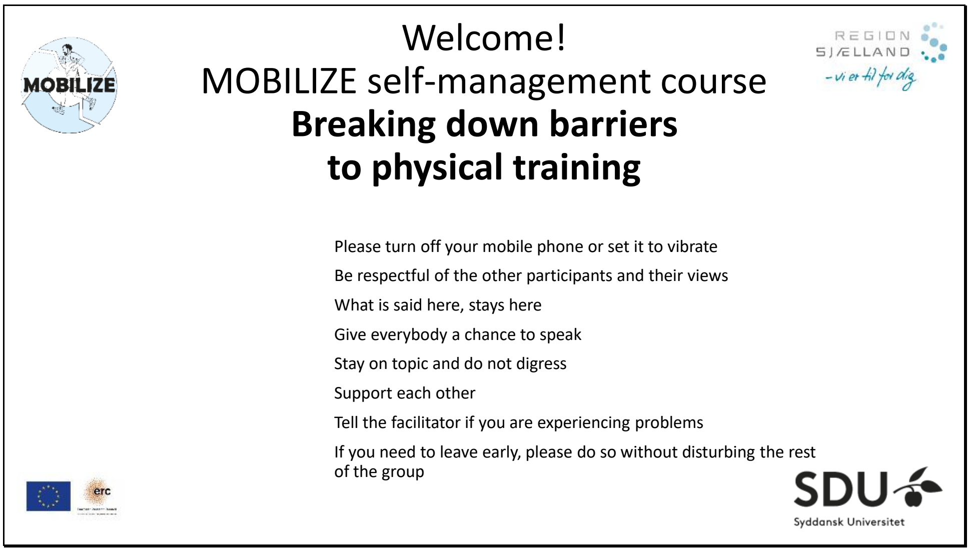

You will need a whiteboard/flipchart, post-it notes and pens for this module.

Welcome to the thematic module on breaking down barriers, where we will talk about what kinds of obstacles to exercise and physical activity can arise and how you can break them down.

Slide 2

MOBILIZE

Barriers to training

If you want to incorporate more physical activity into your daily life – not because others say you should, *<u>but because you want to</u>* – it can be a good idea to take a closer look at the barriers that stand in the way of actually getting started.

Slide 3

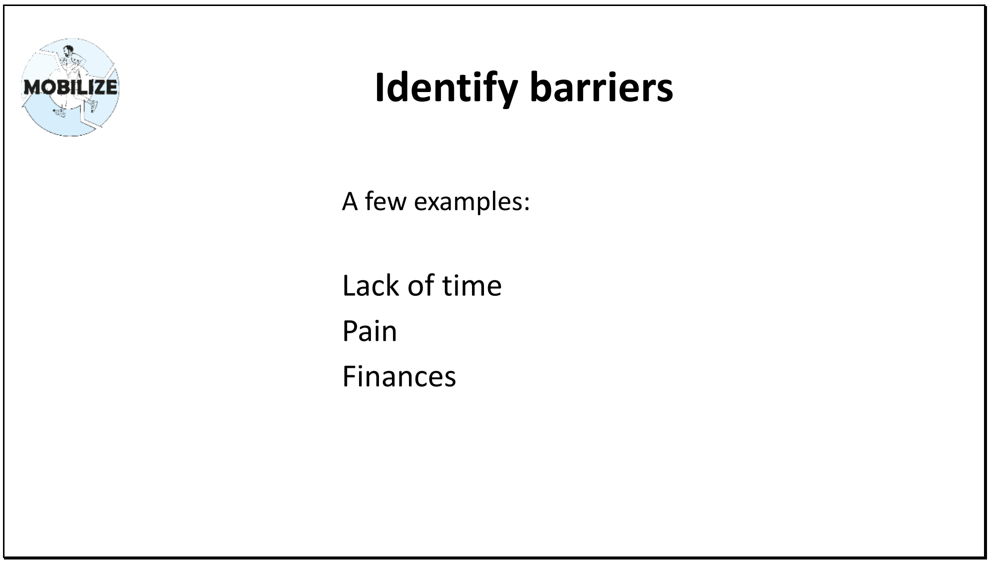

Have the participants identify barriers to training based on their personal experiences.
Write the examples on a whiteboard/flipchart with enough space to add post-it notes next to each barrier later.
(Duration: max. 5 minutes).

Slide 4

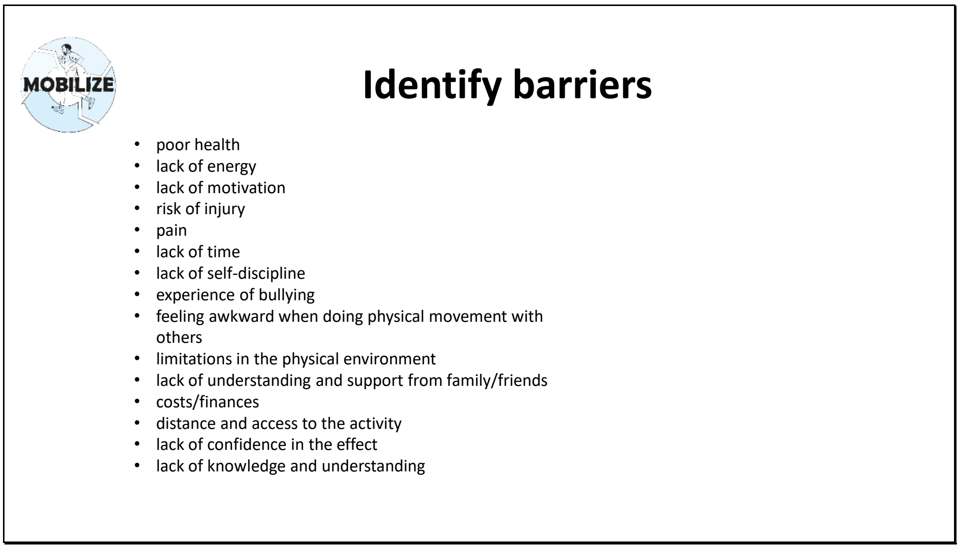

Here is a list of some of the barriers that various research studies have identified among people aged 60+.

Clarify points that may be unclear:
<u>Experienced intimidation</u> => e.g. experiencing being too slow in a team
<u>limitations in the physical environment</u> => busy roads, poor pavements, lack of lighting

Highlight any that the participants themselves have identified.
Ask them if they recognise any stress reactions they haven't mentioned themselves.

Slide 5

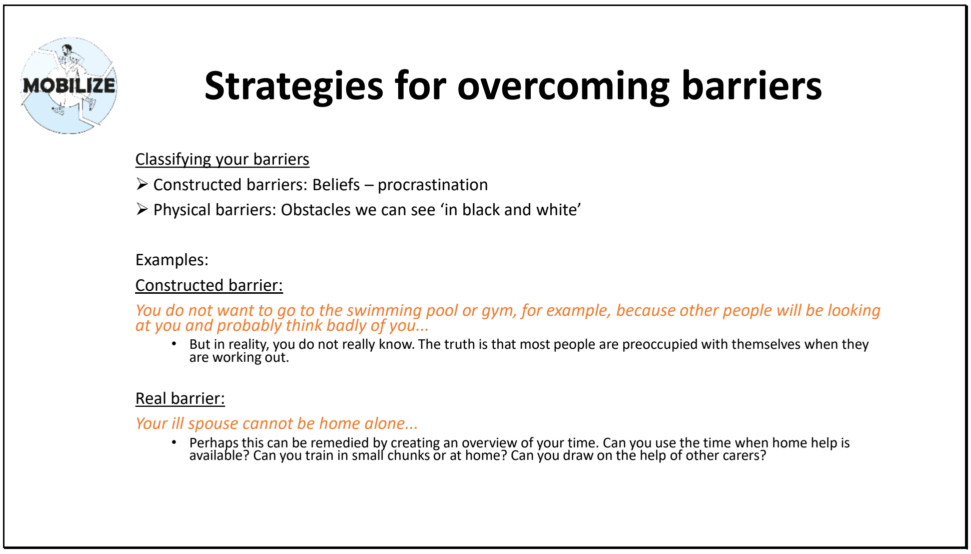

Introduction to slide:
The mental barrier is often much greater than the real barrier once you get started. The vast majority of barriers are <u>beliefs</u> that are not true or not as true as we think. Or barriers that can be overcome if you take a closer look at them, because it is <u>not</u> necessarily a question of either/or.

End of slide:
This way, you can work on classifying any barriers in terms of whether they are constructed or real.

Slide 6

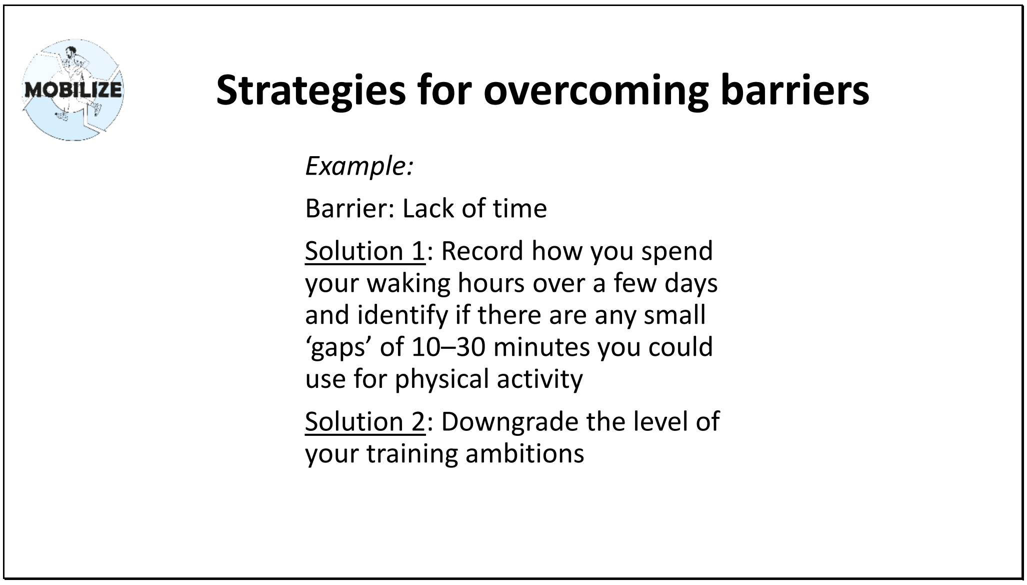

After reviewing the slide, give the participants this task (remember post-it notes and pens):
Take some time to think about solutions to and strategies for breaking down barriers to exercise. Team up in pairs and select 1–3 of the barriers you have previously identified. Try to find solutions together.
You need to find at least one solution for each barrier. Write each solution on a post-it note and place it on the board next to the barrier in question.
(Duration: 10–15 minutes)

Participants can take a picture of the proposed solutions with their mobile phones so that they can be inspired by the solutions at home.

Slide 7

The ambition paradox

The higher the ambitions we set for our training, the less likely we are to actually exercise. When we have to take on too much at once, we become overwhelmed and can't be bothered. Then our brain starts giving us a whole lot of suggestions for procrastination and all of a sudden it is late at night, and you still haven't exercised.

You do not compensate for inactivity with big ambitions.
You compensate for inactivity by moving.

Paragraph 1) As a consequence, you 'beat yourself up' and think that since I didn't work out today, I'll have to do even better tomorrow – thus raising the ambition level even further in an attempt to compensate for inactivity. This is a vicious circle...

Paragraph 2) Break the vicious circle: Forget making plans for big ambitions – just move!

Slide 8

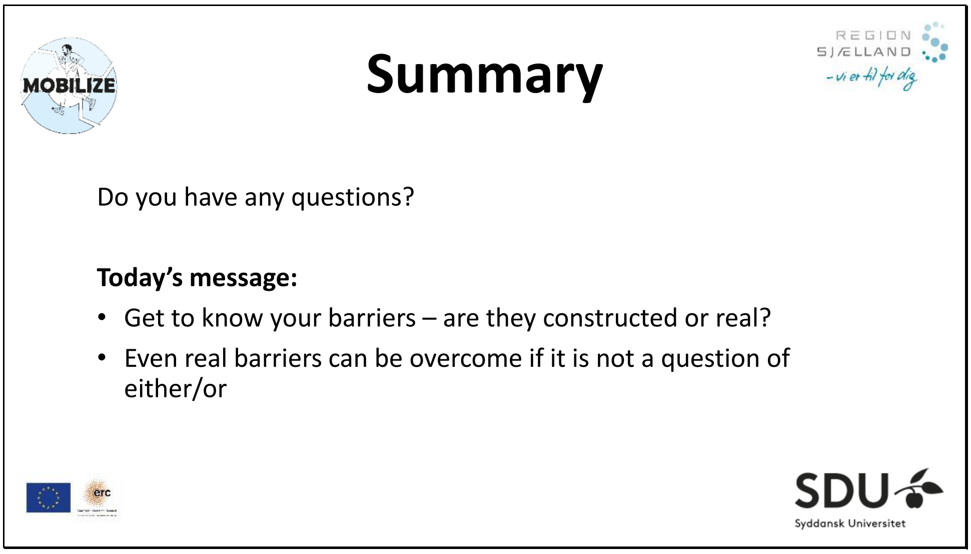

Answer any questions and remind the participants that the mental barrier is often much bigger than the real barrier and that real barriers can often be overcome by thinking outside the box.

Pain mechanisms

Slide 1

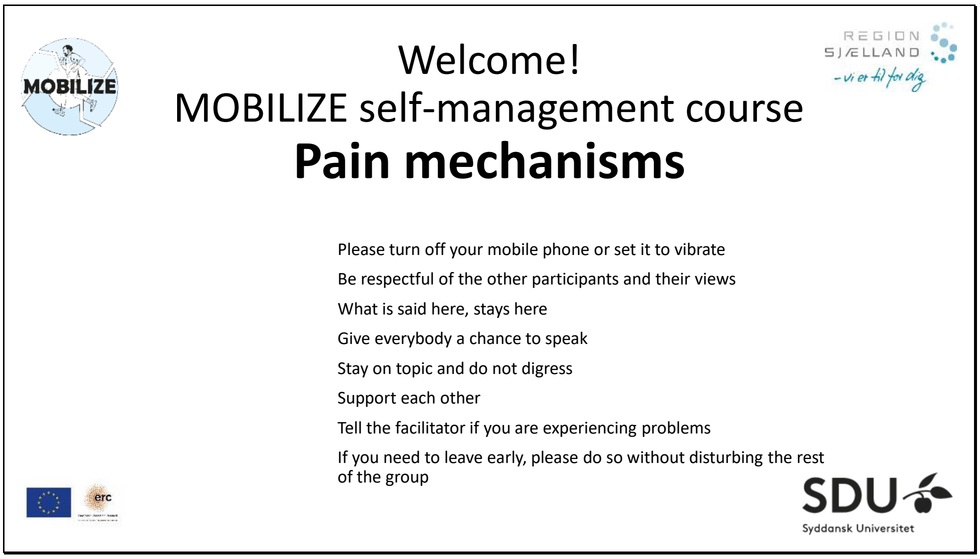

Welcome to the thematic module on pain.

Most of you who have a chronic condition have in common that you experience long-term or chronic pain. According to the Danish Health Authority, more than one in five people in Denmark live with pain.

Today we will talk about:
Pain mechanisms in acute and chronic pain.
How to influence and live with chronic pain.

Slide 2

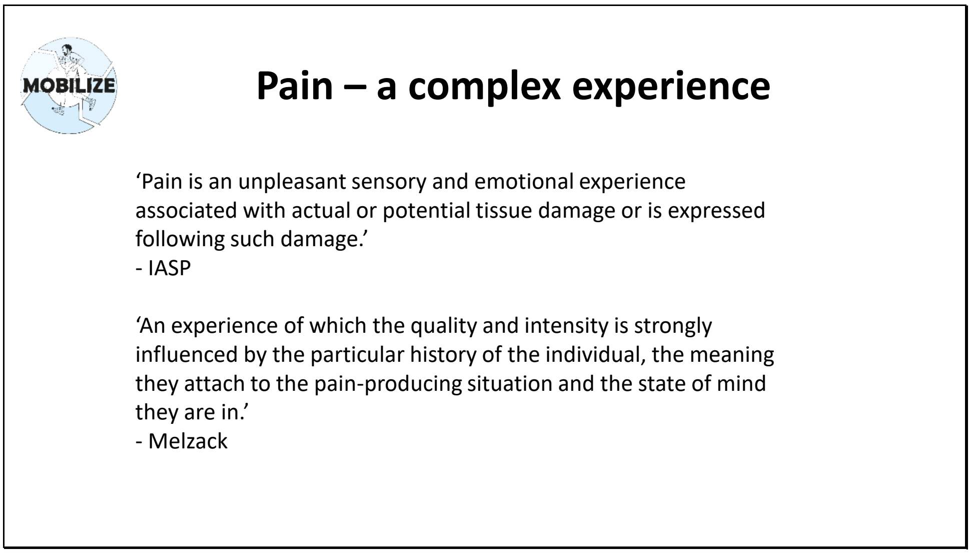

Ask the participants what pain means to them before clicking further (why do we get pain, how does it feel, what emotions does it evoke?)

Here are a few official, recognised definitions of pain:

Paragraph 1) IASP: International Association for the Study of Pain.

Paragraph 2) Ronald Melzack is a Canadian professor of psychology who has done groundbreaking research on pain.

End of slide:
Based on these definitions, it is clear that pain is not only a sensory experience. It also has an emotional impact.

Pain is therefore complex and is currently best described according to the biopsychosocial model of pain, i.e. where physical, emotional and social factors play a role. We feel pain instead of perceiving pain, and pain occurs when the brain has assessed that we are potentially in danger, have an injury or are currently unhappy in life.

If you have fallen and hurt yourself, you feel pain due to an injury. However, if you are still in pain 6 months or 6 years later, it is probably not due to tissue damage. This does not mean that you aren't in pain, but that there is another reason for the pain you are experiencing.

Slide 3

MOBILIZE

Acute pain

- Imminent or actual harm
- Stopping
- Learning what to avoid
- Has a purpose

Acute (i.e. sudden) pain is a warning signal from our nervous system to our brain that:

Point 1) From this point of view, pain has an important function in preventing damage to the body and, if damage occurs, in re-establishing the body's normal functions. The pain disappears when the danger is over, i.e. when the injury has healed. For example, when you burn yourself on the oven, pain is triggered immediately and lasts until the skin is intact again.
The acute pain mechanism has an important protective function. We would not be able to look after ourselves properly if we were born without this sense.

Point 2) Acute pain tells us to stop an action or force because it could harm us.

Point 3) It teaches us what to look out for to avoid injury and makes sure we protect an injured part of the body so that the injury does not get worse and healing can take place in the best possible way.

Point 4) Acute pain has a specific purpose and is essential for our survival.

Slide 4

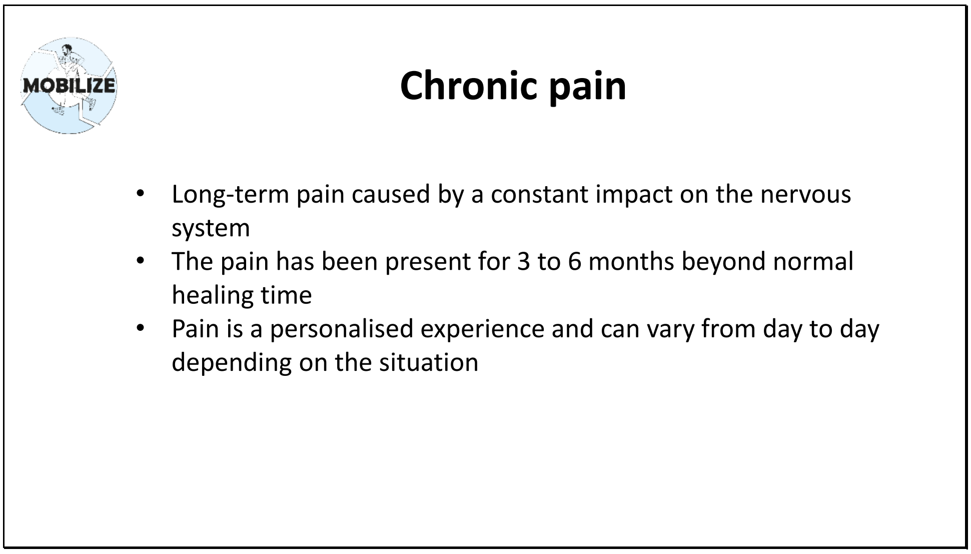

Point 1) For instance, osteoarthritis of the knee or lower back. The pain worsens with strain and sometimes at rest. Here, chronic pain can fulfil the same function as acute pain, for example, making sure we limit the load on the worn-down knee. The pain often follows a specific pattern.

Point 2) There is a smooth transition from acute pain, where clear changes can be seen in the area of injury, to chronic pain, where there may be permanent tissue damage, but where the pain can also be present even if the tissue has healed. It is said that the time factor is what determines whether it is acute pain or chronic pain.

Point 3) Pain can be inhibited or amplified by our thoughts.

Slide 5

MOBILIZE

Complex chronic pain

- Causes other than the original injury
- Has no purpose as a warning system
- Constant 'state of alert'

Point 1) In complex chronic pain, biological/physical, psychological and social factors all play a role in your pain experience. **Ask the participants if they recognise/have experienced how their pain experience can be affected by mood, surroundings, etc.**

Point 2) There is a disturbance in the pain-regulating mechanisms of the central nervous system and it is not a sign of injury. It can develop as a result of damage to a person's nervous system or changes in the system due to persistent pain. The nervous system gets better and better at sending pain signals, but loses the ability to be specific, so the pain will spread.

Point 3) The body is in a constant 'state of alert' and releases stress hormones that amplify the amount of pain impulses to the brain.
The brain registers pain much more strongly. With minimal triggers, chronic pain sufferers experience severe pain. This is also known as hyperalgesia.
A normal non-painful impulse (light touch, cold, warmth, heat, draft, noise, movement) is recognised and experienced as pain. This phenomenon is called allodynia.

Slide 6

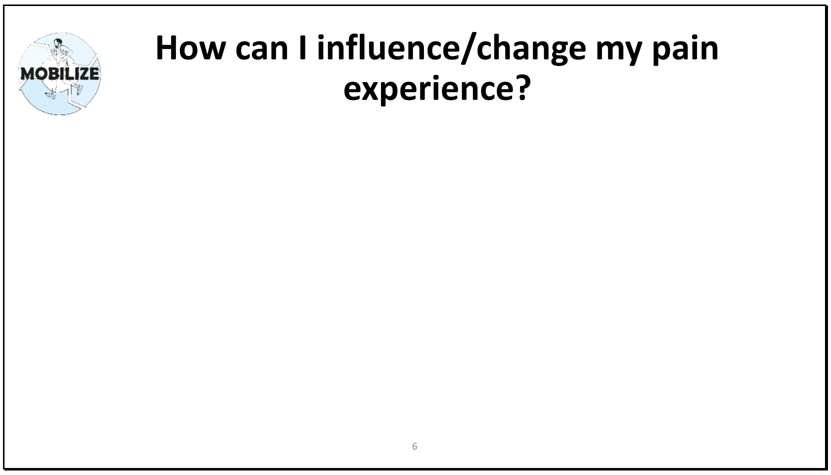

Introduction to slide: Thoughts, emotions, body and behaviour all play a role in chronic pain conditions. You can influence your pain experience by influencing these areas.

This is used in a form of therapy called cognitive behavioural therapy, which works to raise awareness of the connection between thoughts, feelings and behaviour.

This can be demonstrated by the following model:

Chronic pain can be influenced by your thoughts, emotions, bodily sensations and behaviour.

In other words, the experience of pain sometimes makes us think, feel and act in a certain way, and this can often trigger a certain process that in turn affects the pain experience in either a positive or negative direction.

Emotions, thoughts, body and behaviour affect each other.

Negative thoughts can lead to unpleasant feelings and an uncomfortable sensation in the body, which in turn can affect behaviour.

Conversely, happy thoughts can also lead to a positive reaction in body and behaviour.

An example of how this interaction can 'turn up' and 'turn down' the experience of pain:
You are walking and have pain in your back. You wonder if you might have something seriously wrong with you, you get a feeling of unease and worry. You start to tense your body more, change your behaviour and avoid things that make the pain worse. Your change in behaviour affects your

body and may make your pain worse. Your thoughts turn even more to the pain and you start having catastrophic thoughts, thinking that you might end up with impaired mobility.
On the other hand, this negative spiral can also be positively influenced by the fact that you may get clarification that there is nothing wrong during an examination. You get a sense of security and peace of mind. The body relaxes and you change behaviour and move more freely with normal movements and do normal activities. This in turn provides positive input to your body, mind and emotions.

Ask the participants if they have examples of/experiences with this themselves. (Duration: max. 5 minutes)

Slide 7

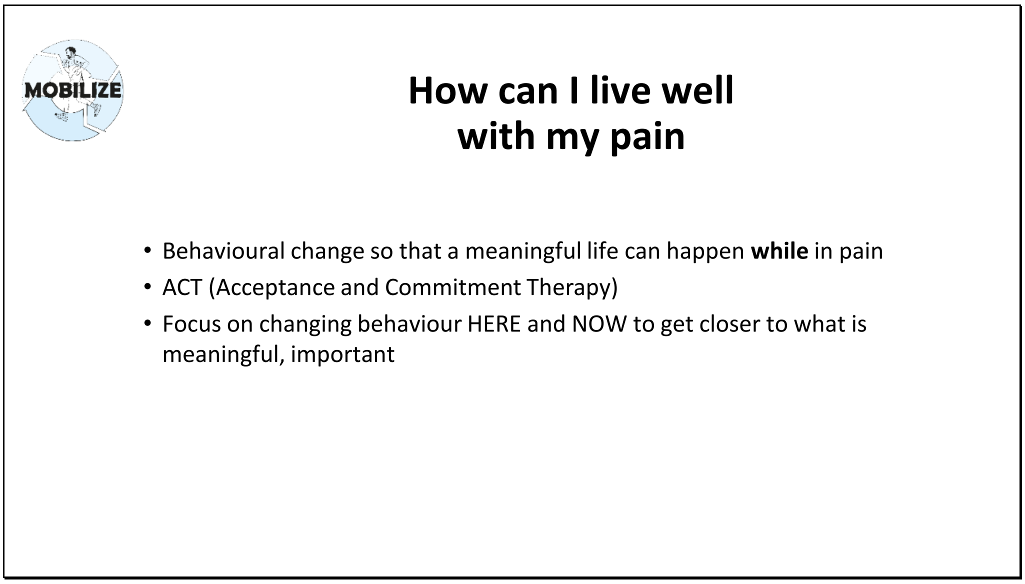

Introduction to slide: Another approach might be that instead of trying to influence and minimise your pain, you can find a way to live with your pain.

Point 1) Develop a greater ability to be flexible in your thinking and enable you to deal with pain and other discomfort in a more appropriate way and thereby live a better life that is in line with the way you want to live your life. For example, taking part in social events that make you happy even though you are in pain. Choose to go for a walk with your friend and have a good chat, even if you are in pain. However, this can sometimes be difficult to do yourself. There is a further development of cognitive behavioural therapy that focuses on this and may be of help to you.

Point 2) Is a further development of cognitive therapy, which corresponds to an 'acceptance and commitment approach' (ACA). Facilitates changes in <u>behaviour.</u>

Point 3) ACA does not generally focus on the physical pain, which may come as a surprise when it is precisely the pain that you want to disappear. With ACA, you try to figure out how, despite the pain, you can still move in the direction and perform the actions that make sense in your life.
As an added bonus, many people will find that the pain is reduced and that over time it becomes less of a burden and doesn't affect their quality of life in the same way as it did before. For most people, this is a wonderful added bonus that can come when you *don't* focus on the pain, but rather on what you want to do with your life.

ACT is not a 'fix it' approach but a 'live with it' approach. Has been shown to be particularly effective for chronic pain. Mindfulness is also part of this, as this approach is about learning to be present in the moment.

You have or will familiarise yourself with mindfulness as a tool to try in other modules.

Slide 8

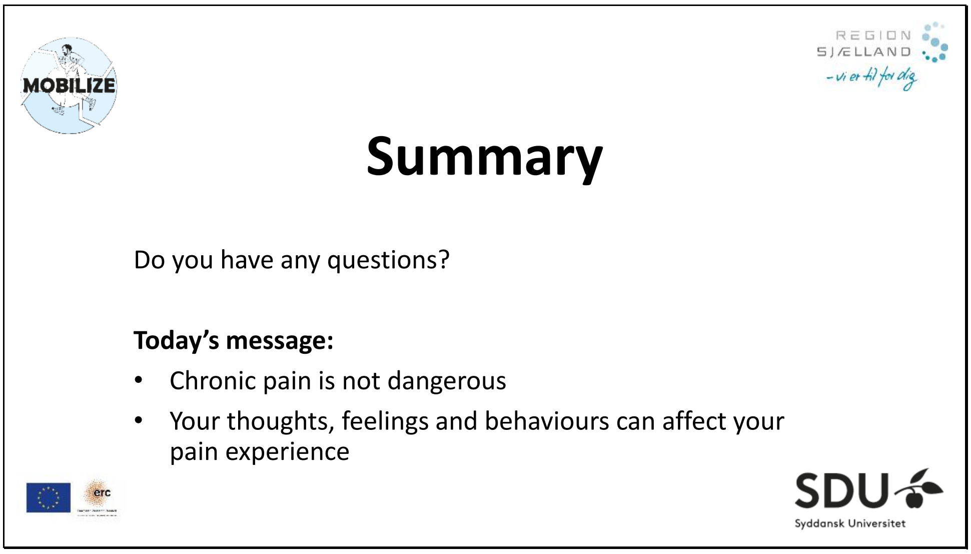

Answer any questions.

Try to work with thoughts and behaviours that positively influence your pain. But remember if you get <u>new</u> pain or <u>severe worsening</u> contact a healthcare professional.

Also in your daily life, try to focus on living a meaningful life where you, and not the pain, determine what you do.

Communication

Slide 1

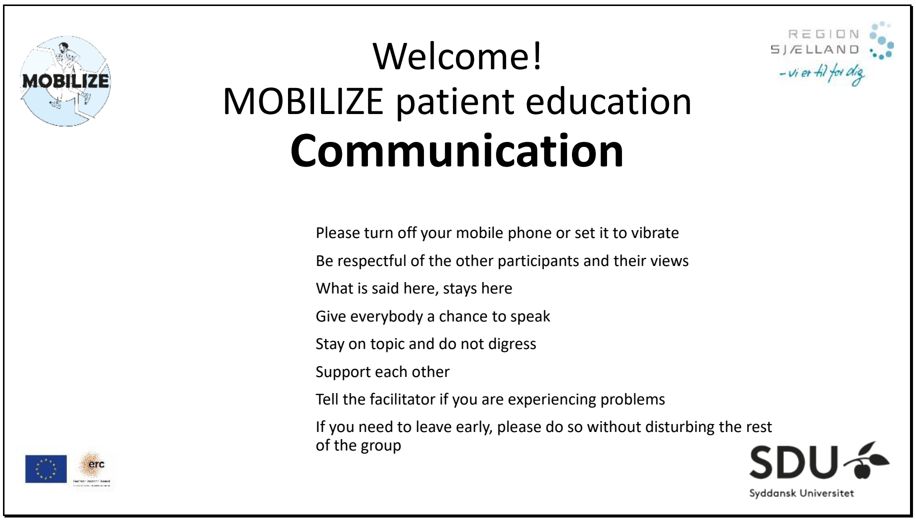

Whiteboard/flipchart and marker pen will be used.

Welcome to the thematic module on communication.

Communication between healthcare professionals and patients greatly influences the patient's experience of their treatment. Today, we will look at what you can do to contribute to good communication.

Communication can be defined as mutual information exchange and includes both verbal communication (i.e. what is being said) and nonverbal communication (i.e. body language, gestures, tone of voice, etc.) that takes place in the direct encounter between the patient and the healthcare professional.

Slide 2

What does it mean to be partners in healthcare?

Doctors (and other healthcare professionals) and patients establish a partnership that ensures an equal collaboration that will contribute to the best solutions.

The healthcare professional contributes professional knowledge, and the patient contributes **personal knowledge and experience**.

Paragraph 1) According to the Legal Rights of Patients Act of 1998, patients are entitled to:

- obtain adequate information about their state of health and treatment options
- have an informed say in the course of their treatment, which means that the information provided is also understood
- gain insight into their own journal and have the content explained if needed
- have next of kin informed and involved to the extent they themselves wish
- have information adapted to the recipient's individual circumstances in terms of age, maturity, experience, etc. This also means that the patient can insist on not having information.

Paragraph 2) Good communication between the patient and the healthcare professional is an important element in all phases of the patient journey.

In order for patients to get the most out of their treatment, it is crucial that:

- patients get the knowledge and information they need
- healthcare professionals get the necessary insight into the individual patient

Slide 3

MOBILIZE

Clear Speech – communication between doctor and patient

Slide 4

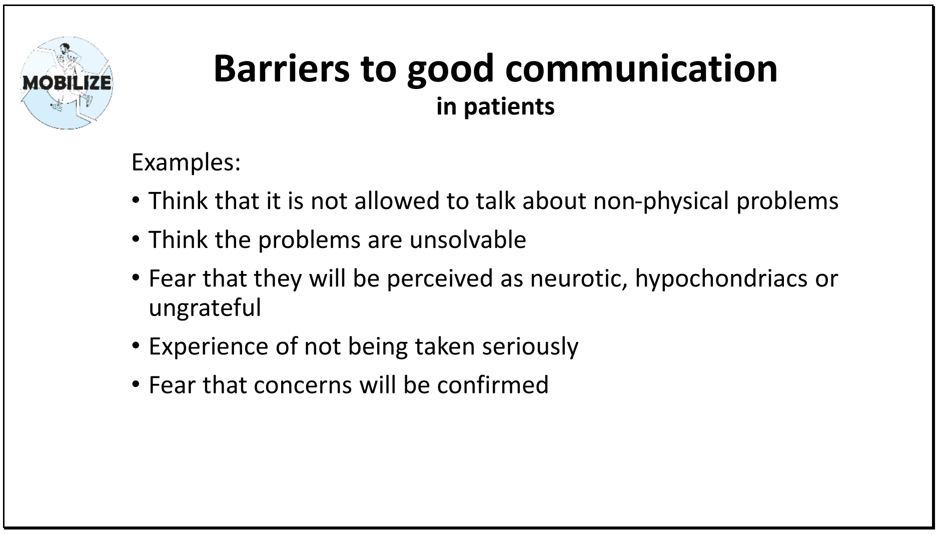

Introduction to slide: Communication in a stressful situation requires special attention, because the communication will not be direct and equal between the participants. One participant is the healthcare professional, who is at work in their own universe and in a familiar situation, and the other is the patient who is in a strange place in a vulnerable situation.
This means that there is a high chance of misunderstanding.

Before clicking further to the points, ask the participants: What barriers do you encounter when communicating with healthcare professionals? **Discuss (5–10 minutes).**
Write their examples on the flipchart/whiteboard.
Then click on the examples of what other patients have experienced in their communication with healthcare professionals.

Slide 5

What can you do yourself?

- Be prepared and specific when describing your symptoms, etc. to the doctor.
- Ask if in doubt: Can you explain it in other words?
- Take notes. They may remind you later about what the doctor said.
- Pay attention to the limited time the doctor has.
- You can also ask a family member or friend to attend your appointments with the doctor.

It is important to be aware that you are always communicating. Avoiding conversation or adopting negative body language is also communication...

Before clicking further to the points, ask the participants: What do you do to promote good communication with healthcare professionals and what has worked well?
Write their examples on the flipchart/whiteboard (Duration: max. 10 min).
Then click on the examples of what patients can do themselves.

End of slide: In addition, it is also important that a healthcare professional is a good communicator:

- Has respect for their patients
- Has the ability to share information in a form patients can understand
- Does not interrupt patients
- Does not see patients as stereotypes
- Has the ability to align their own and patients' expectations

Slide 6

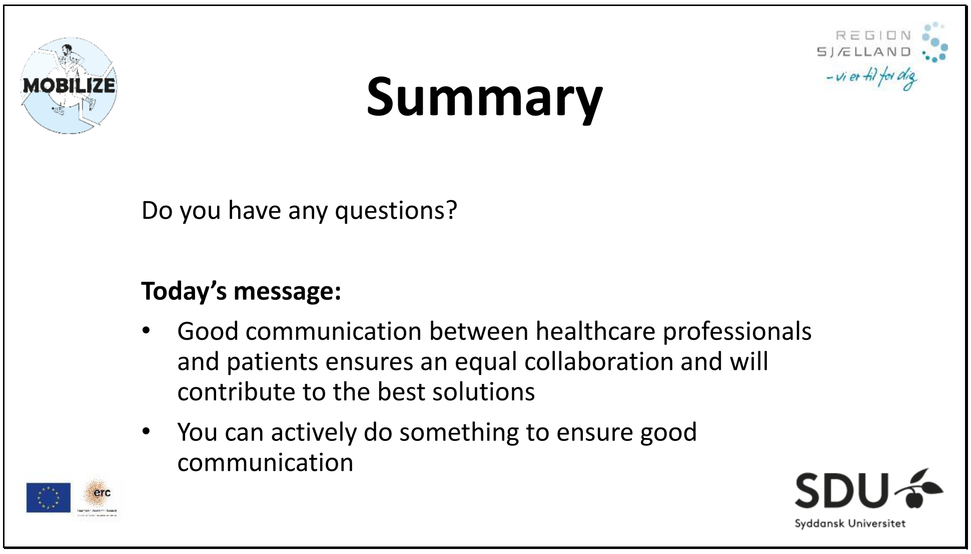

Answer any questions.

Ask if the participants feel better equipped to ensure good communication with healthcare professionals.

Physical training and chronic illness

Slide 1

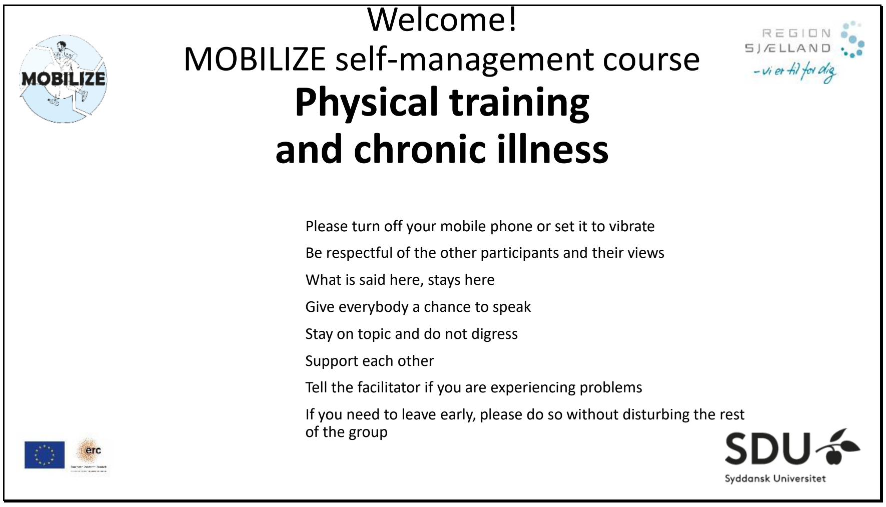

Welcome to the thematic module on physical training for patients with chronic conditions.

Today we will take a closer look at what we know about exercise as a treatment for a wide range of chronic conditions and how you can exercise safely with your conditions.

Slide 2

Physical training for people with chronic conditions

Exercise and physical activity are safe and effective as:

- Potential primary **prevention** of at least 35 chronic diseases
- **Treatment** for at least 26 chronic diseases

Start where you are, use what you have, do what you can

Exercise is safe, and a little is better than none!

Slide 3

Illnesses	Recommendations	Special focus areas
COPD	Endurance training, walking or cycling with prolonged activity at 70–85% of maximum oxygen uptake in combination with strength training.<br><br>People with mild COPD should aim to be physically active in line with the Danish Health Authority's general recommendations for physical activity.	• People with severe COPD could consider oxygen during exercise.
Heart failure	Endurance training in which the intensity and duration of training sessions are gradually increased.<br><br>Alternatively, dynamic training/strength training of smaller muscle groups can be used.	• People with angina pectoris should exercise to the level just below the ischaemic threshold, i.e. avoid provoking ischaemic pain during exercise.<br>• Heart pain or similar discomfort is a signal to slow down or, perhaps best of all, take a break.<br>• Patients with ischaemic heart disease should refrain from short-term, high-intensity interval training<br>• Patients with severe ischaemia and dyspnoea (shortness of breath) at rest should take a break from exercise with low loads
Elevated blood pressure	Endurance training, dynamic strength training (preferably with light weights and many repetitions) and combination training all reduce blood pressure.<br><br>In general, endurance training is most effective at moderate to high intensity.	• It is generally recommended to avoid heavy physical exertion if systolic BP>180 or diastolic BP>105 mmHg<br>• Caution is advised for very intensive dynamic strength training or strength training with very heavy lifting.

Introduction to slide: In the following, we will go through specific recommendations and special points of attention, i.e. situations where you should adjust or stop your training in relation to six of the most prevalent diseases among the Danish population (which are the six diseases in the MOBILIZE project).

Many of you also have other conditions than the ones we will discuss here. We therefore recommend that you familiarise yourself with the Danish Health Authority's handbook 'Physical activity – handbook on prevention and treatment on 31 disorders and associated risks'. Your other conditions may be covered there. Find the link in your patient handbook.

Slide 4

Illnesses	Recommendations	Special conditions
Osteoarthritis	There are no guidelines that recommend one form of exercise over another. Cardio, strength training, coordination training and/or functional training can all reduce pain and improve physical function.<br><br>To achieve an effect on pain and physical function, it is recommended to exercise 2–3 times a week for at least 6 weeks.	• If pain increases after exercise, you may need to take a break and adjust your training programme.<br>• In cases of obesity, weight-bearing exercise should be done with caution.<br>• In acute joint inflammation, training should be modified/stopped until the effect of medical treatment is achieved.
Diabetes	Cardio training with gradually increasing intensity in combination with strength training.<br><br>The patient should aim as a minimum to be physically active following the Danish Health Authority's general recommendations for physical activity, but greater effect is achieved by exercising more and at higher intensities.	• Physical activity should be postponed if blood sugar levels are above 17 mmol/l or below 7 mmol/l until corrected.<br>• In retinopathy (diabetic eye disease), high-impact strength training with a sharp rise in blood pressure is contraindicated due to the increased risk of damage to the eye's blood vessels.<br>• In cases of peripheral nerve damage and risk of developing foot ulcers, caution is recommended for weight-bearing activities.
Depression	There are no guidelines that recommend one form of exercise over another.<br><br>The patient should aim as a minimum to follow the Danish Health Authority's general recommendations for physical activity.	• Nothing general

Slide 5

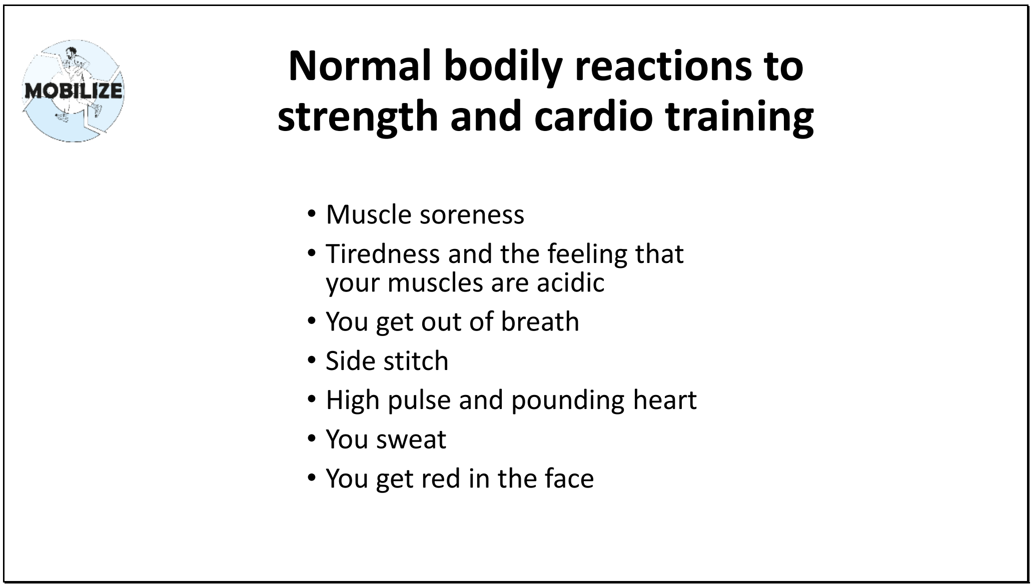

Introduction to slide: Ask the participants what bodily reactions to strength and cardio training they are familiar with before clicking further to the points.

End of slide:
It is normal to feel tired and have sore muscles during and after physical activity. This is especially true if you do unfamiliar activities or use your muscles in a different way than you are used to. It is also normal to experience muscle soreness if you haven't been physically active for a long period of time. It is a normal and harmless reaction. The soreness will disappear on its own after a few days and you will have fully recovered.

It is also perfectly normal to feel out of breath, get side stitches, sweat profusely and have a pounding heart.

If you get muscle injuries, they will often cause severe pain and mobility will be significantly reduced. It can be downright excruciating and not just sore and painful. The pain often takes a few days to subside.
With many types of muscle injuries, you will also get swelling near the injury, which never occurs with normal 'training pain'.

In addition, you should stop exercising and seek medical attention if you experience chest pain or severe shortness of breath during exercise that does not go away even when you stop exercising.

Slide 6

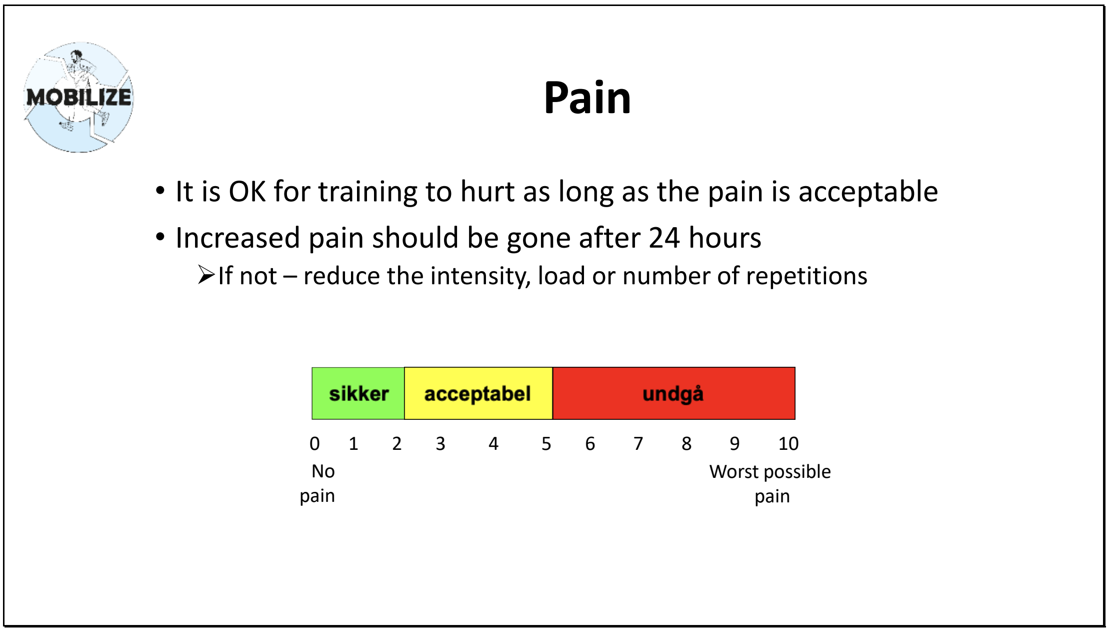

Introduction to slide: It can be difficult to customise your training sessions and find the motivation to exercise in general if you are in pain. Many people opt out of training beforehand because it seems unachievable.
But it is important, especially for people with chronic illness, to be physically active. The more active your life is, the better quality of life you will have. Exercise is necessary to maintain or increase function, and a strong body can cope with more in daily life without increasing pain.

Points 1–2) It can be difficult to know your own limits – especially if you haven't used your body for a long time. To help with this, the MOBILIZE project uses the Numerical Rating Scale to assess pain, where a score of 0–5 is considered acceptable pain in terms of continuing to exercise, but a score of 6–10 indicates that the exercise/training should be adjusted.

Slide 7

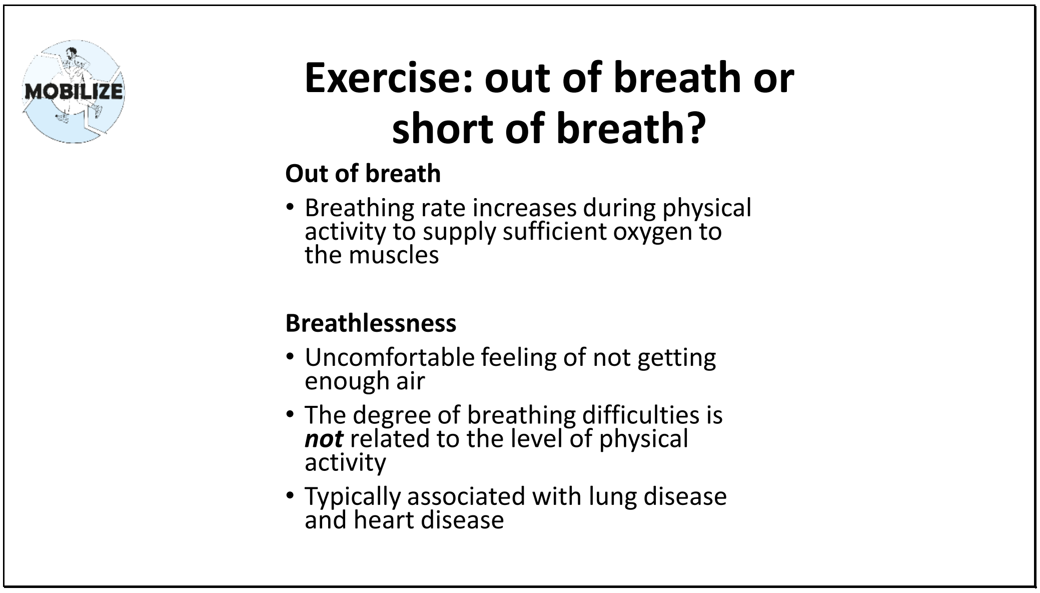

Introduction to slide: Ask the participants to suggest what the difference is between out of breath and breathlessness before clicking further to the bullet points.

Out of breath
For example, when we are running a little late for catching the bus.
Or when accompanying others (walking/running/cycling) who are more fit than you are.
Or when speaking while walking/running/dancing.

In cardio training, it is a given that you will get out of breath and it will feel 'hard', but it shouldn't feel unpleasant!

Breathlessness
Can occur at rest, when speaking and during light activity.

End of slide:
<u>You can manage breathlessness in these ways:</u>
- Puffing sound (Pursed Lip Breathing): exhaling through pursed lips
- Relieve the breathing muscles: When you are short of breath or have had a severe coughing fit, a comfortable resting position can help you breathe calmly again.

In another module, you will try (or will have tried) different breathing techniques.

Slide 8

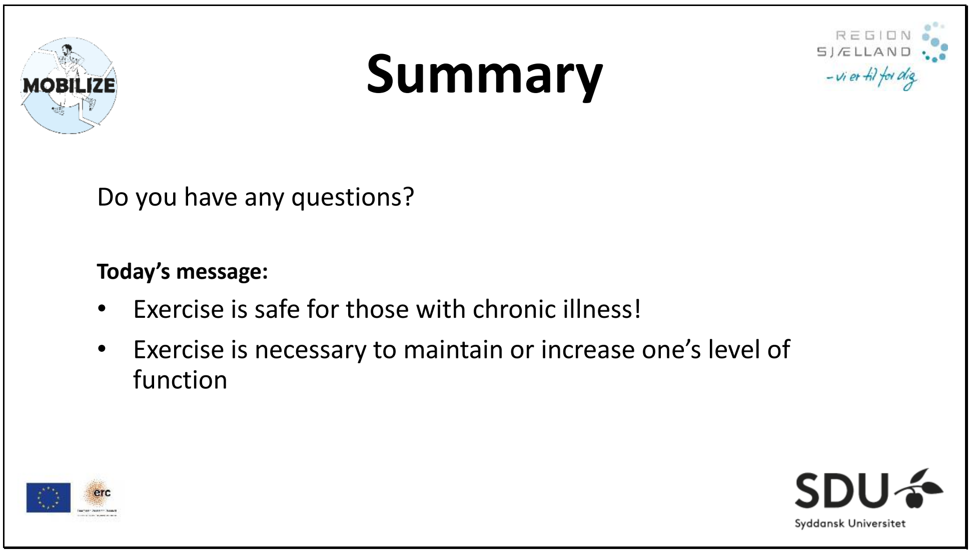

Answer any questions.

Find more information in the Danish Health Authority's handbook 'Physical activity – handbook on the prevention and treatment of 31 conditions and associated risks'. Find the link in your patient handbook.

Mindful eating

Slide 1

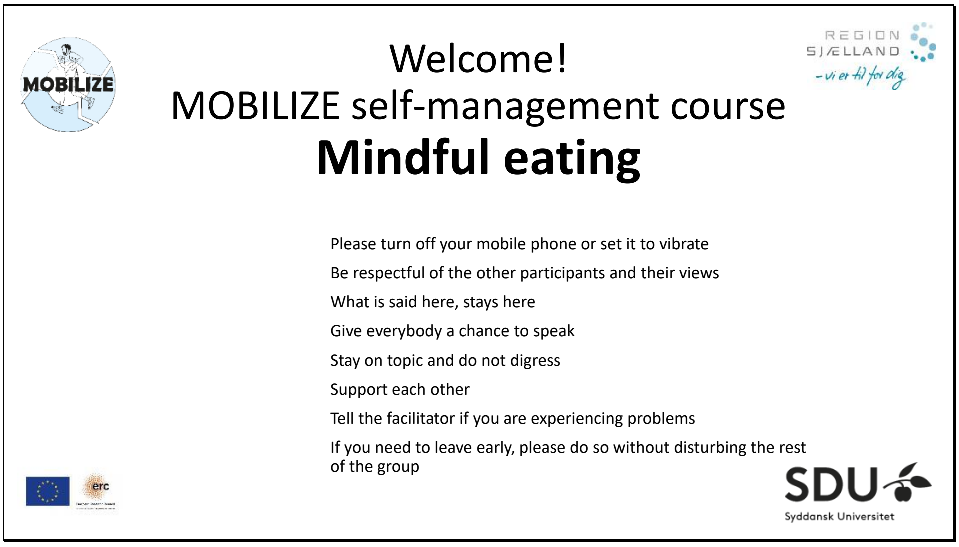

Considerations before the teaching session:
Be aware of whether there are participants who do not have an appetite and who may prefer not to lose weight, but to gain weight. They will feel full before they even start eating. In that case, focus on enjoying the food – and on making the participants aware of when and how they eat – are there times of the day when they perhaps completely forget to eat?
The session has two focal points. First, a focus on when and in which situations the participants eat – and then a focus on eating itself, which includes the exercise of eating a raisin. If for some reason a participant cannot eat/tolerate raisins, then a piece of dark chocolate or an apple can also be used for the exercise.

Bring raisins/alternative to raisins

Welcome to the thematic module on mindful eating.

Today will be a workshop in which you become more aware of your eating habits – particularly in the eating situation itself, but also in relation to when, why and how you eat. Mindful eating is about being present when you eat – and the method is particularly used in relation to weight loss/weight maintenance.

Slide 2

Become aware of your eating habits

- When do you eat?
- Where do you eat?
- How do you eat?

Eating habits are about many things. Today, it is not so much about what is on the plate, but more about becoming aware of what habits we have in relation to consuming food (meals, snacks and drinks) and becoming aware of what is happening right at the moment we eat the food. First, it will be about what habits we have in relation to consuming food.

Ask the participants whether there is a difference in what they eat in the different situations.
<u>When do you eat</u>? (e.g. fixed mealtimes (3 or more), when I'm hungry, when I'm bored, when something is served...)
<u>Where do you eat</u>? (e.g. at the dining table, in front of the TV, in the car, at a restaurant/café/with friends...)
<u>How do you eat</u>? (e.g. alone >< with others, while I'm reading/watching TV, using the iPad, talking on the phone...)

Slide 3

MOBILIZE

Mindful eating – eat with awareness

- Notice whether you are hungry or whether it is another feeling that causes you to eat
- Remove distractions
- Use your senses and notice colours, smells, sounds, textures and tastes
- Eat slowly, breathe and chew your food thoroughly
- Stop when you are full

Introduction to slide: Now it will be about becoming aware of what happens at the moment when we eat. When you want to eat more attentively/with presence (mindful eating), you can advantageously focus on these elements of eating.

Point 1) It may be relevant to become aware of physical and emotional hunger – is the body really hungry because it needs nutrients and energy or do other emotions initiate eating (you are bored, you are sad …)?

Point 2) If you eat in front of the TV or read while you eat, there may be a risk that your attention is diverted from the food – then perhaps it is the TV programme that determines when you have finished eating – instead of you stopping eating when you feel full. It may also be relevant to focus on a good atmosphere around the meal (ventilation, calmness...) – this can be particularly important if you do not have a great appetite, as bad smells, interruptions, etc. can reduce the intake of food.

Point 3) You focus on the food and become more aware of what you eat and how much – enjoyment is essential.

Point 4) Pace is important for what we are able to perceive, so when the pace is slower we are able to be aware and use our senses – we can enjoy the food. Breathing calmly and chewing your food thoroughly are ways to slow down.

Point 5) The feeling of satiety begins to occur approx. 20 minutes after we have started to eat – so it may be a good idea to eat slowly and pay attention, and don't have a second portion until 20 minutes after the beginning of the meal. We also get 'full' through our eyes, so if you eat the same portion on a small rather than a large plate, the portion will appear larger and more filling than if it is served on a large plate.

Ask the participants what is important for when they start eating and stop eating?

Slide 4

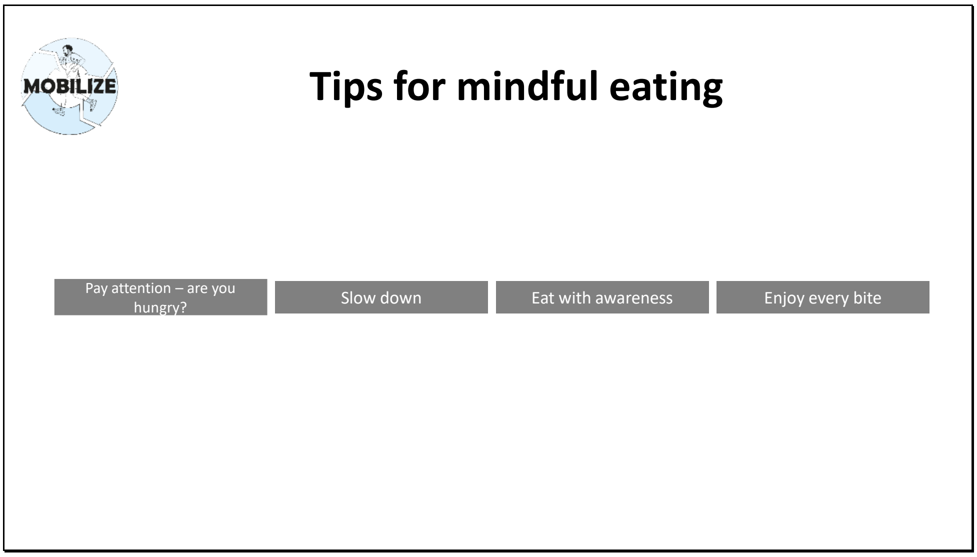

Overall, mindful eating can be summed up in these four points – and apart from the first point, you will now try mindful eating.

Slide 5

Taste a raisin

Conduct a brief mindful eating session with the participants (Duration: max. 5 minutes). You can also use other food than a raisin, e.g. a piece of dark chocolate or an apple.
In a calm voice, the therapist guides the participants through the exercise with the following instructions:

- Take a raisin and put it in your hand
- Give yourself plenty of time to focus on the raisin
- Turn the raisin around and examine its consistency
- Smell the aroma and scents of the raisin
- Slowly bring the raisin up to your lips
- When you are ready, prepare to chew and taste the raisin, noticing how and where it needs to sit in your mouth to be chewed
- When you feel ready, you can swallow the raisin. Continue to be aware of the experience
- Notice how your body and mind as a whole feel after you complete this exercise

Ask afterwards:
Now you have tried eating a raisin with mindful eating. How was the experience?

Slide 6

Mindful eating

Sit in pairs and discuss these six questions. Think about your current eating habits.

Why?
Why do I eat?

When?
When do I want to eat?

What?
What do I eat?

How?
How do I eat?

How much?
How much do I eat?

Where?
Where do I invest my energy?

Put the participants in pairs and ask them to discuss the six questions (Duration: approx. 15 minutes).

You may have covered some of the questions earlier – but let the participants think about their day yesterday or just their dinner from yesterday. Ask if it was 'a typical' day – or if there was anything special, and if so, what did it mean for the experience of the meal/day's eating habits?

Slide 7

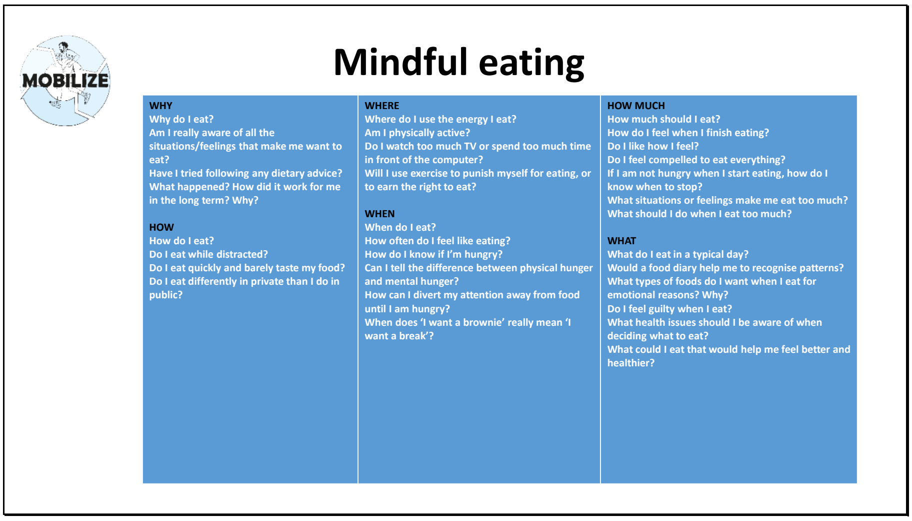

This slide is <u>not</u> to be presented – but can provide inspiration for the discussion introduced on the previous slide. That is this slide can just be on the screen while the discussion is going on.

Slide 8

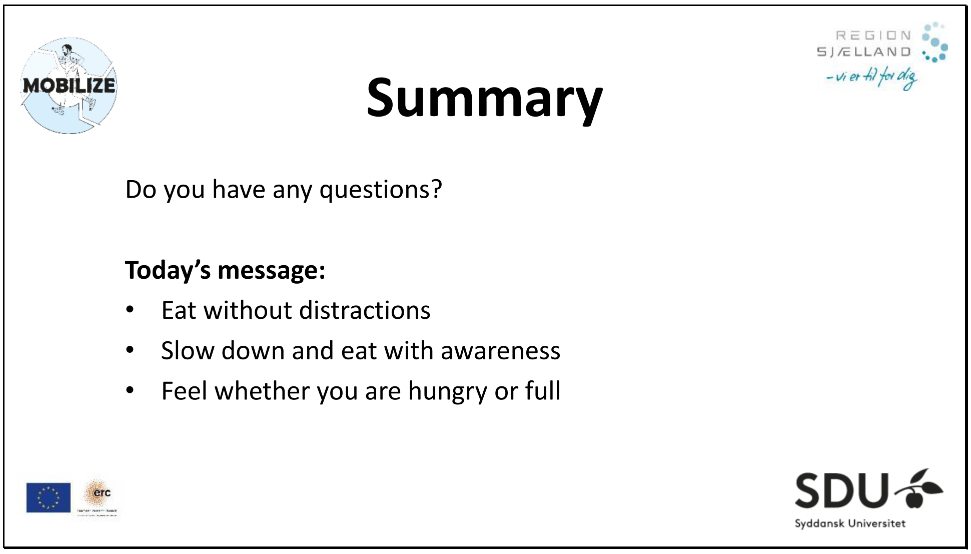

Answer any questions.

Suggestions for home activities:
Write a diary and become aware of your eating habits. See how in the patient handbook.
Try doing this for 2 weeks (or maybe just 1 week).

Breathing and shortness of breath

Slide 1

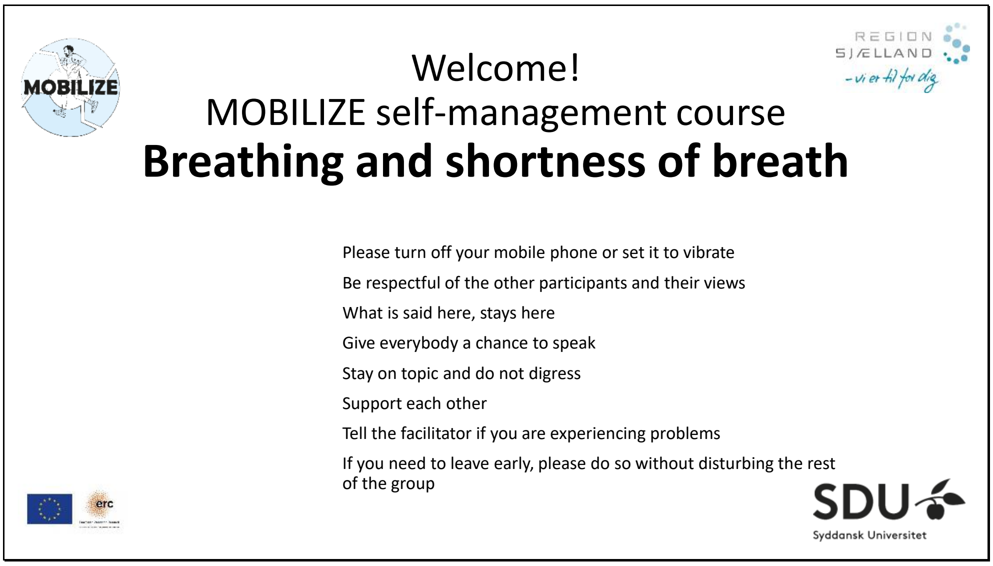

Welcome to the thematic module on breathing and shortness of breath. You might know the feeling of gasping for breath – but are you just out of breath or do you have shortness of breath? Today we will take a closer look at how we breathe and what the difference is between being out of breath and having shortness of breath.

Slide 2

The structure of the lungs

The lungs consist of three lobes on the right side and two on the left side. There is room for the heart close to the left lung.

The trachea, also known as the windpipe, connects to the two lungs, from where it branches out into many smaller airways called bronchi.

At the end of the bronchi are about a billion tiny air sacs that look like little bunches of grapes called alveoli.

The lungs are elastic tissue. Elasticity allows healthy lungs to easily fill with air and deflate. When you inhale, the lung tissue expands. When you exhale, the lung tissue contracts again.

Slide 3

Breathing – how to

The lungs are located relatively high in the chest.
Try to feel on your own body as I talk **(have participants feel the sternum and rib curvature)**. The rib cage consists of the ribs and the sternum. At rest, the lungs only reach the tip of the sternum.

The diaphragm is a thin, dome-shaped muscle attached to the lower ribs and spine.
In the following , illustrate with your hands while explaining how the diaphragm works: Interlace your fingers like a dome in front of you and then press down like a plunger, flattening the dome and spreading your fingers apart (while still interlaced).
The diaphragm is our primary breathing muscle. After exhaling, it is dome shaped. When inhaling, it flattens out and creates negative pressure, like a plunger, sucking air into the lungs. The stomach is distended. On exhalation, the diaphragm relaxes into the dome shape, creating positive pressure in the lungs which pushes the air out. The stomach returns to its normal position.
During normal breathing, only a small 'plunger' movement of the diaphragm will occur.

We have accessory muscles between the ribs, from the shoulder girdle to the ribs, and from the shoulder girdle/upper ribs to the neck.
Their job is to help the diaphragm when we are out of breath, for example, during physical activity.

COPD and asthma patients tend to use the accessory muscles a lot – and can be very tired and sore in these muscles.

Slide 4

Breathing – why

In order for cells to metabolise nutrients, they need oxygen, and breathing plays an important role in this. Oxygen is carried into the lungs via the air we breathe in. Around the lungs we have both oxygen-rich and oxygen-poor blood. The oxygen-rich blood is transported from the lungs to the cells, where it is used as fuel during muscle work. During metabolism, the cells produce the waste product carbon dioxide, which the blood transports back to the lungs.

The gas exchange between oxygen and carbon dioxide takes place in the outermost branches of the lung tissue – in the many small air sacs called alveoli.
Oxygen (O2) is transported from the alveoli to the bloodstream, while carbon dioxide (CO2) is released from the bloodstream to the alveoli.

We exhale carbon dioxide and inhale oxygen.

Slide 5

MOBILIZE

Breathing in practice

- At rest, we normally breathe at a frequency of 8–12 times per minute = 20,000 times in a day
- *A good inhale is a prerequisite for a good exhale ☺*

Point 2) Many people believe that if we just make sure we breathe in well and 'fill our lungs with air', everything will be fine. But a good inhale is a prerequisite for a good exhale.

Brief exercise to illustrate the importance of the exhale:
We are now going to do a quick exercise to help you realise the importance of a good exhalation.

- Sit up straight in your chair and then take a good deep breath, filling your lungs with air.
- Now breathe out just under half of the air again before taking another breath as deeply as possible. Repeat, blowing out just under half of the air before taking the next breath and feel how it becomes more and more difficult to take in fresh air.
- End with a good long exhale and notice the difference it makes for the next inhale.

Example with a glass of water: take an empty glass and pour in a splash of water and pour out an even smaller splash of water. Then you add a little more and pour a little less again, and so on. Eventually, this means that the glass is filled to the brim and there is no room to add more water. In exactly the same way, the lungs must be emptied before there is room for new air. The exhale should even be slightly longer than the inhale.

Slide 6

Factors that can affect our breathing

- Stress
- Thoughts and feelings
- Pain
- Body posture
- Obesity
- Lung diseases

Introduction to slide: There are a number of factors that can negatively affect our breathing.

Point 1) Fast, shallow, slightly tense breathing

Point 2) Does not allow optimal breathing

Point 3) Shallow, high and fast breathing

Point 4) No room for optimal breathing with increased rounding in the thoracic spine, head pushed forward and rounded shoulders => increased activation of the accessory muscles which become fatigued and sore and stiff.

Point 5) No room for the diaphragm to work optimally, as a fuller figure yields more resistance.

Point 6) Emphysema, COPD. Asthma => Decreases the exchange of oxygen from the lungs to the bloodstream; increases the activity of the accessory muscles.

Slide 7

Out of breath or shortness of breath?

Getting out of breath

- The breathing rate increases during physical activity to supply sufficient oxygen to the muscles

Having shortness of breath

- Uncomfortable feeling of not getting enough air
- The degree of breathing difficulties is ***not*** related to the level of physical activity
- Typically associated with lung disease and heart disease

Managing shortness of breath

- Make a puffing sound (Pursed Lip Breathing)
- Make a good long exhalation
- Relieve the breathing muscles

Paragraph 1)

Shortness of breath: For example, when we are running a little late for catching the bus.
Or accompanying others on a walk/run/cycle ride who are fitter than you are.
Or when speaking while walking/running/dancing.

In cardio training, it is a given that you will get out of breath and it feels hard, but it shouldn't feel uncomfortable!

Severe shortness of breath is a sign that the cardiopulmonary circulation does not match the physical activity. It can be a sign of reduced fitness due to inactivity, illness or bed rest.
During your training, we want you to train with a moderate load, which is why we use the BORG 'talk test'.

Paragraph 2)

Shortness of breath: Can occur at rest, when speaking and during light activity.
Shortness of breath cannot be measured with a device – e.g. as blood oxygen saturation can (pulse oximeter, typically a finger clip)
It is a subjective experience only – but it can be scary.
The body's reaction: unease and anxiety increase.

NOTE: If you experience chest pain during exercise, you should seek medical attention.

Paragraph 3)

Managing breathlessness:
Puffing sound (Pursed Lip Breathing): exhalation through puckered lips
Relieve the breathing muscles: When you are short of breath or have had a severe coughing fit, a comfortable resting position can help you breathe calmly again.

Resting positions: **Ask participants to do the first two:**
Sit up straight
Sit slightly bent forward with your elbows resting on your thighs
Stand with your back straight against a wall
Stand at a high table slightly bent forward with your elbows resting on the tabletop

Slide 8

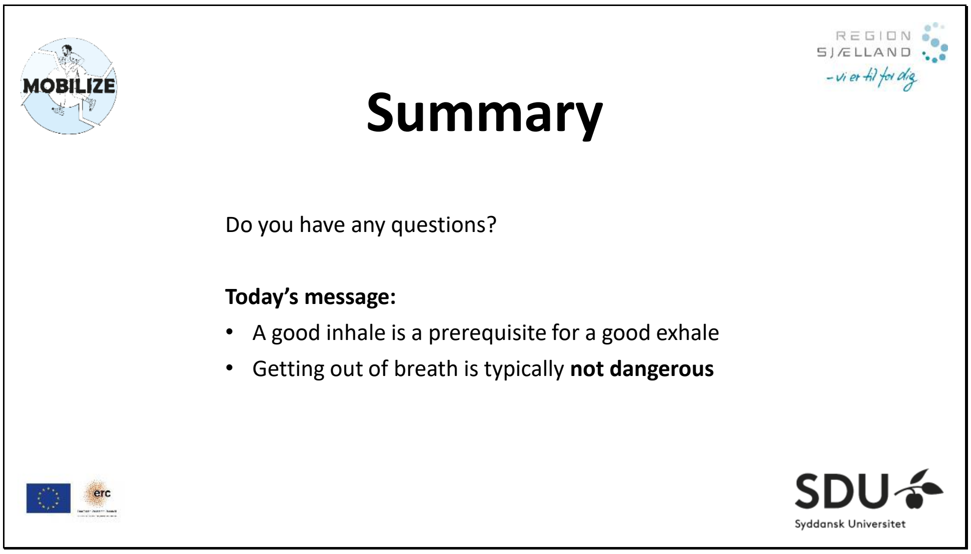

Answer any questions.

Remind participants that getting out of breath is not dangerous.

Self-care

Slide 1

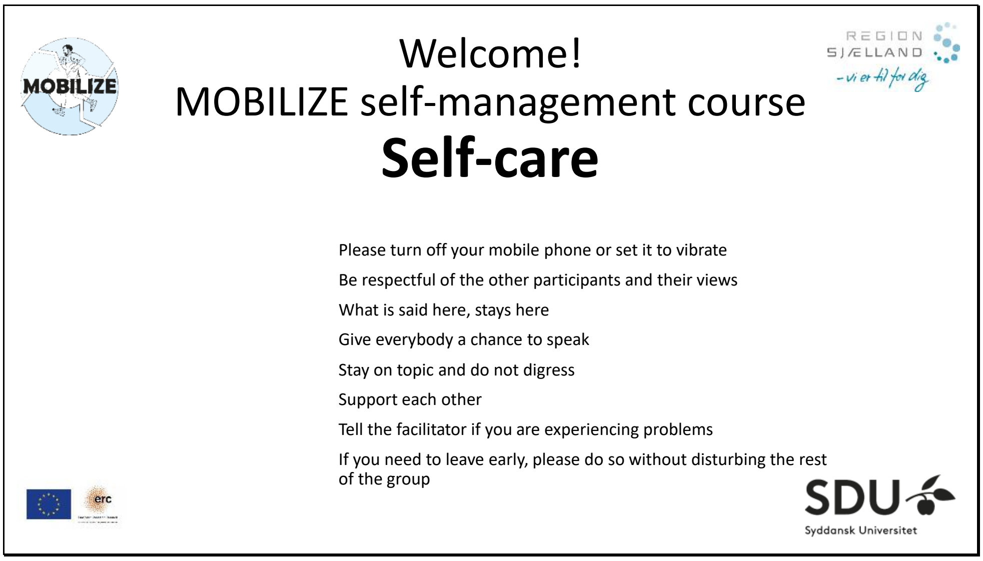

Welcome to the thematic module on self-care.

Self-care can be defined as the action pattern of a person that aims to promote or maintain physical and mental health. Today we will talk about how you can increase your ability to support and accept yourself – even when something is difficult.

Slide 2

Self-care

Self-care is the practice of individuals looking after their own health using the knowledge and information available to them.

In other words:

Participating actively in your own treatment, seeking knowledge, having healthy lifestyle habits, engaging in constructive collaborations with healthcare professionals, etc.

Or simply:

Self-care is the good things you do for yourself

End of slide: Self-care can thus be seen both from a health perspective (‘contributing to keeping yourself healthy’) and a more individual perspective (‘do things that you feel are good for you’).

Slide 3

The 7 pillars of self-care – from a health perspective

1. Health competence
2. Mental health
3. Physical activity
4. Healthy eating habits
5. Risk reduction
6. Good hygiene
7. Sensible use of resources

Introduction to slide: The health perspective on self-care is covered by the following pillars:

1. Health competence: Refers to the ability to read, understand and act on health-related information.
2. Mental health: Life satisfaction, optimism, feeling of high self-esteem, feeling of mastery and control, seeing a purpose in life and a sense of belonging and being able to get support.
3. Physical activity: Is essential for good health and can reduce the risk of many non-communicable diseases.
4. Maintaining healthy eating habits: Has repeatedly been shown to have a preventive and curative effect on many lifestyle diseases.
5. Risk reduction: Means avoiding or limiting behaviours that directly increase the risk of illness and death.
    - Vaccinations
    - Stopping smoking
    - Safe sex
    - Moderate alcohol consumption
6. Good hygiene: Refers to conditions and actions that can maintain health and prevent the spread of disease.
7. Sensible and responsible use of health-related resources: In connection with self-care, this means that the individual can handle their health problems in a safe and effective way with the relevant medicine and health services.

Slide 4

Self-care
– from an individual perspective

The ability to treat yourself with the same kindness and care you would treat a good friend when something goes wrong, is difficult or feels uncomfortable.

Can be described in 3 parts:

- Kindness to yourself (as opposed to self-criticism)
- Community humanity (as opposed to isolation)
- A mindful mindset (as opposed to judging and suppressing thoughts)

Point 1)
With self-care, we treat ourselves with kindness, warmth, understanding, support and care. We cannot always be who we want to be or get what we want to get. When we don't want to accept that fact, it creates internal pressure, stress and dissatisfaction. On the other hand, when we acknowledge our problems and imperfections without judging ourselves, we can better help ourselves and generate positive emotions that strengthen and build. Kindness towards yourself is also about explicitly training your good will, so that you want the best for yourself both in wishes and actions.

Point 2)
With self-care, we recognise that we are fundamentally like other people. We understand that the experience of being imperfect is part of being human. We recognise common humanity in ourselves and realise that all people have problems at times. It minimises feelings of being different, wrong and alone, which reduces self-blame, self-criticism and the tendency to withdraw from others.

One of the big problems with self-criticism is that it often means that we isolate ourselves from others. When we discover something about ourselves that we don't like, we almost get tunnel vision and can easily feel that everyone else is perfect. That it is only us who are finding things difficult. The same happens when something goes wrong in our external life, then we can feel that

others have an easier time and are in control of their lives. Common humanity helps us realise that life is challenging for most people and that personal mistakes are part of being human.

Point 3)
Showing compassion to ourselves and others, and being present in the moment is the prerequisite for us to give ourselves what we need. Being mindful is about observing what is happening in the moment and registering feelings and thoughts as they are, without evaluating or suppressing them. <u>Mindful thinking is also about being aware that our thoughts are not reality but rather phenomena in the moment, which you can decide to listen to or let pass.</u> Mindful thinking creates more clarity and calmness. In this way, we can respond with kindness and compassion when we feel challenged, precisely because we recognise that we are having a hard time.

End of slide:
These 3 points help build inner resilience by increasing your ability to support and accept yourself – even when something is difficult.

Research has shown that the better you are at self-care, the easier it will be to live with your chronic illnesses.

Slide 5

If you don’t take care of yourself, you can’t take care of others

Taking care of yourself is like recharging your batteries so that you can be there for others.

Self-care is not only beneficial for yourself, but it also helps you to be there for your family and friends.

On flights, we are instructed to put on our own oxygen mask first in emergencies. Because if we don’t take care of ourselves first, we can’t help others.

Slide 6

Introduction to slide: You will now try self-care in practice. You will try a relaxation technique that may increase your physical and mental well-being.
Progressive muscle relaxation is one of the easiest and most effective relaxation techniques to learn. You learn to relax mentally and can reduce your stress by gradually tensing and relaxing your muscle groups one at a time.

It is recommended that the therapists find benches for participants who cannot get on the floor. Alternatively, they can be placed in a sitting position if their breathing does not allow a lying position or there are no benches available, but this is not quite as optimal in this session.

Muscle relaxation exercise (duration: 15 minutes)
Guide the participants:
Allow your attention to focus only on your body. If you begin to notice your mind wandering, bring it back to the muscle you are working on.
Take a deep breath through your abdomen, hold for a few second, and exhale slowly.
As you exhale, imagine the tension in your body being released and flowing out of your body.
Now tighten the muscles in the soles of your feet.
Hold for about 5 seconds, and release.
Pause for about 10 seconds.
Continue the exercise up through the body.
You are now completely relaxed from the tips of your toes to the top of your head.
Please take a few more minutes to rest. Relax.

Slide 7

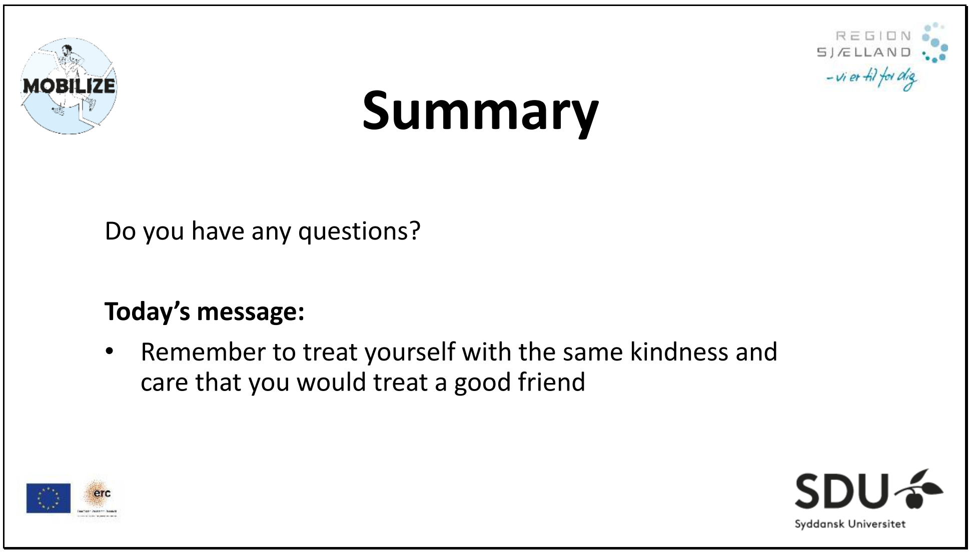

Answer any questions.

Try muscle relaxation at home. See more in your patient handbook.

Self-monitoring

Slide 1

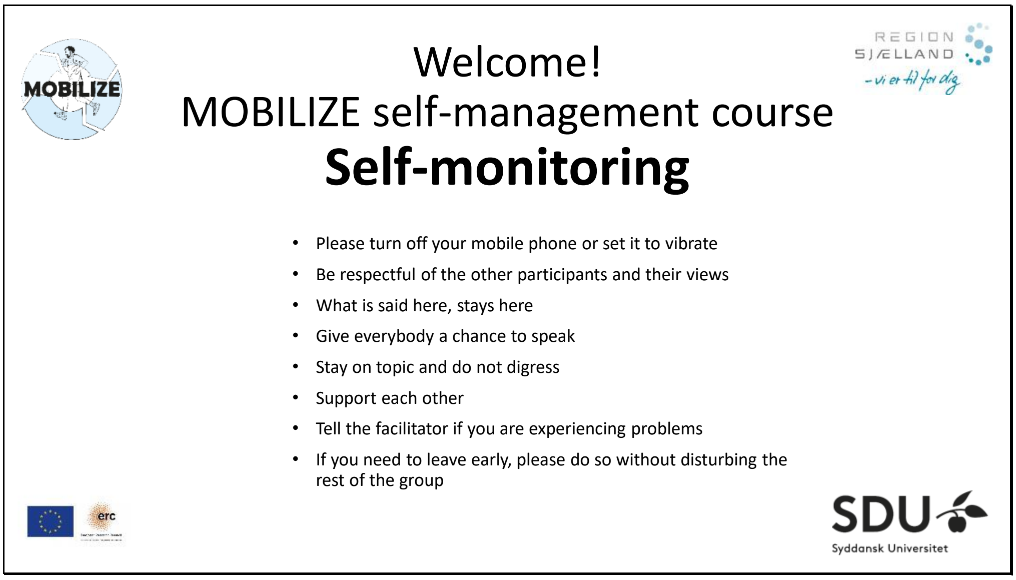

In this session, a step-counter is given to each participant (see slide 7 for details).

Welcome to the thematic module on self-monitoring.

Self-monitoring is a method where a person continuously measures one or more aspects of their body, mind or activities using tools that collect data. Today we will look at how self-monitoring can help us – and affect us.

Slide 2

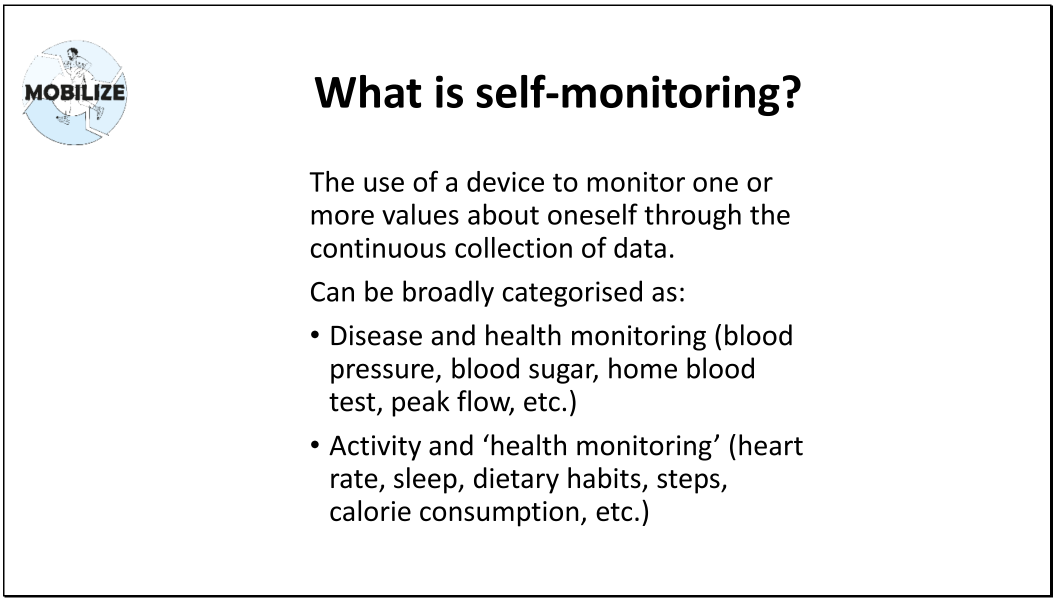

Point 1) As a patient, you are left to take care of your treatment on a day-to-day basis. Self-monitoring is smart, particularly for conditions where relatively frequent measurements are important, such as diabetes. By utilising the benefits of self-monitoring, where you measure your own values, you may be able to have fewer physical check-ups in the hospital.

Point 2) Outside of the healthcare system, there is also a lot of interest in self-monitoring. It has become easy to monitor different aspects of health using various smartphones and smartwatches. The options are endless, and it can be a bit of a jungle to navigate.

Slide 3

Accelerometer measurement

Introduction to slide: You have all worn an activity tracker – an accelerometer – on your thighs and wrists as part of the tests you had to complete at the start of the project. These small USB sticks can monitor how long you have done no, light, moderate or hard physical activity, how long you have done different types of physical activity and how many steps you have walked.
Note: Is it possible to inform participants about their own results, as these are only processed at the end of the programme.

Slide 4

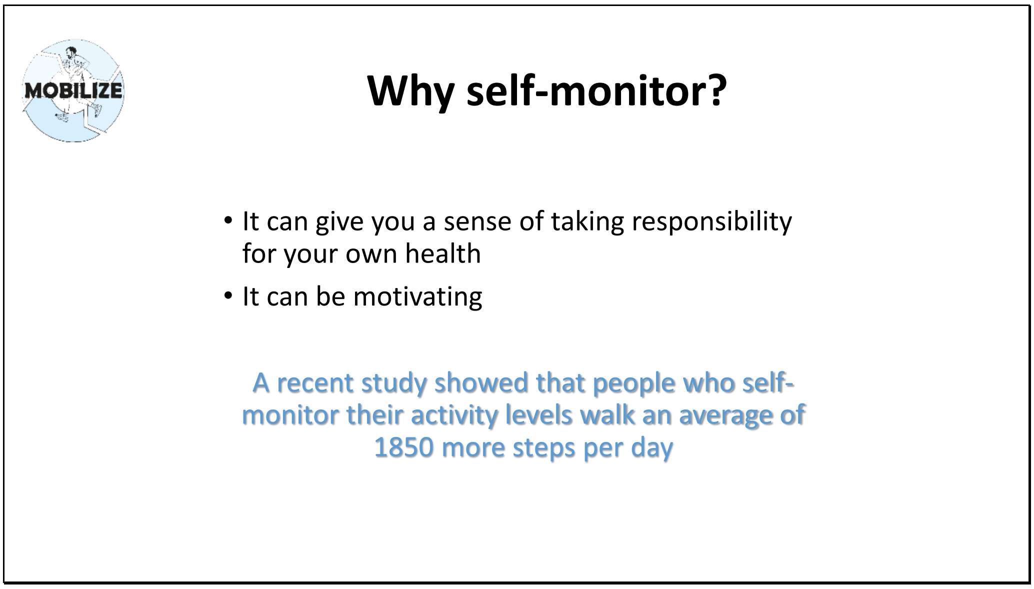

Paragraph 2) Self-monitoring in the form of counting steps, viewing calories, metabolism, measuring distances or seeing time in different heart rate zones can be a motivating factor for some people to increase their physical activity levels.

But it doesn't have to be complicated – a simple step-counter in your pocket, an activity watch or the health app on your mobile phone is also good.

If you aren't so keen on technology, you can also fill out a training diary like the one we have in the MOBILIZE project. You can easily create your own or continue with the existing one in paper format after the project.

Ask the participants: Do any of you have experience with using self-monitoring? **If as a therapist you have experience, please share it. (Duration: max. 10 minutes)**

Slide 5

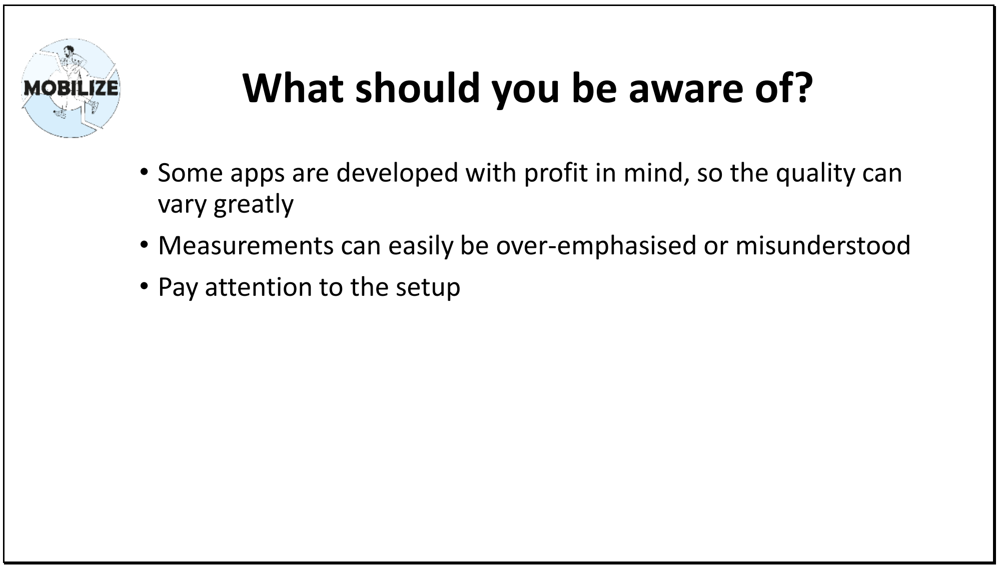

Introduction to slide: Tools that count steps or remind you of your medication can be straightforward, but there are also more complicated measurements such as home blood tests, mole scanning, etc. With these, you need to pay a little more attention.

Point 1) Some apps are developed by companies that may have completely different interests (e.g. money) in mind than the best interests of the patient. Therefore, be critical before using an app.

Point 2) Self-monitored measurements can easily be misunderstood, over-interpreted and generally more value can be attributed to them than it should be. Many of the existing health and lifestyle app measurements result in a number or some kind of score, which users then more or less accept as the truth. Measurement figures rarely take into account the user's other life factors, and the device can also measure completely incorrectly.

Point 3) Be careful not to receive too much data all the time – it can be more stressful than helpful.

Slide 6

MOBILIZE

A few examples of Danish apps

- My Walk App (Hjerteforeningen)
- TrænSmertenVæk (Sundhed.dk)
- Træn Selv (DGI)

Note: some features in a free app may cost money

Introduction to slide: Most mobile phones can count steps, and many do just fine with this. IF you are interested in more info/data or want help keeping track of your walks/exercise, etc. there are various mobile apps with a multitude of options.
The vast majority of apps are in English – in a systematic search we conducted, only 8 out of 45 health apps were in Danish (and those that were translated into Danish were often of poor linguistic quality).
Here, we highlight three free Danish apps that can help you lead a more active life. For more information about where to get them, see the patient handbook.

Point 1) With the walking app, you can track your walks, invite your friends to join you, create company teams, share your experiences with others and participate in the association's various challenges. You can view your walk on a map, see the number of steps, kilometres, total time you've walked and how many walks you have done.

Point 2) The app 'TrænSmertenVæk' enables you to exercise at home and can be used by people who have neck, shoulder, back, hip or knee pain. Once you have downloaded the app and answered questions about your pain, you will receive an exercise programme tailored to your challenges. At the same time, you can keep track of your progress, how your pain is progressing and how active you are. This will help you learn more about how exercise and movement can alleviate your pain.

Point 3) The 'Træn Selv' app helps you stay active in a busy life. All workouts can be done without equipment at home on your living room floor or in the garden. There are beginner, intermediate and advanced training programmes, with durations as short as 4 minutes and as long as 1 hour.

Slide 7

MOBILIZE

Homework

Count your steps daily for a week

Use the step-counter provided:

- Your mobile phone (health app, Google Fit)
- App (there are many different apps that count steps)
- Fitness tracker watch

Introduction to slide: Did you know that the ‘10,000 steps’ mantra comes from the marketing of a Japanese pedometer called Manpo-kei, which translates directly into 10,000 steps!?
The fact that studies have subsequently shown that this activity goal improves heart health, mental health and even lowers the risk of diabetes may go some way to explaining why this arbitrary number has stuck.

However, new research suggests that all activity has a health benefit, and even an average of 7,000 steps per day reduces the risk of premature death by 50–70% compared to those who walk less than 7,000 steps. However, the 10,000 steps campaign can still be seen as spot-on, as the study also shows that the more steps, the lower the risk of mortality up to 10,000 steps, whereas mortality does not decrease significantly for those who walk more than 10,000 steps.

Hand out step-counters to the participants and help them get started: 1) The step-counters are preset to a stride length of 70 cm 2) If you want to reset, this must be done manually – hold down the ‘triangle’ for 2 sec. 3) The step-counter does not need to be switched on – it works when you start walking.
Instructions to participants: The idea is that you each try to record your daily steps for a week. Use the step-counter you’ve been given (or a smartphone or activity watch). You can record the number of steps in your patient handbook and measure/track how many steps you take. If you have any questions about how to do this, we will take some time now to see if you can help each other, and I will help as much as I can. **(Duration: max 10 minutes).**

Slide 8

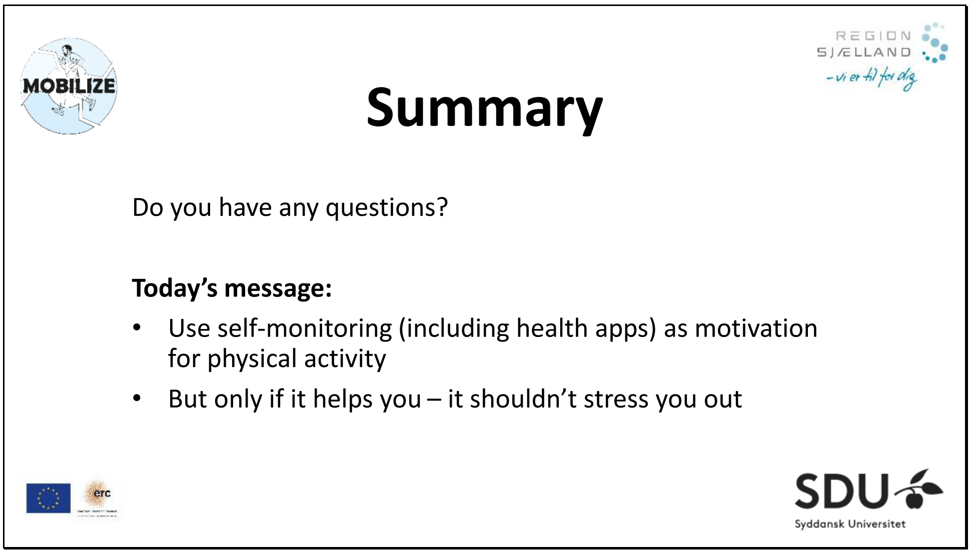

Answer any questions.

Remind participants that self-monitoring is a way to promote self-care. But dealing with this data should never become a stress factor.

Mindfulness techniques

Slide 1

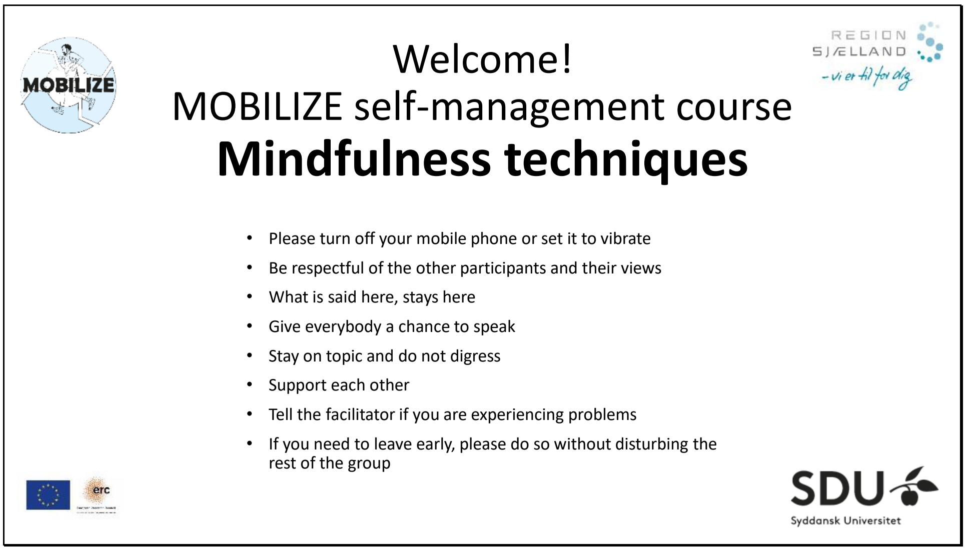

Welcome to the thematic module on mindfulness techniques.

Today we will talk about mindfulness and different techniques.
Mindfulness, like physical training of the body, can be a good and relevant tool for coping with chronic illness.

It is important to remember that:
Mindfulness is training for your mind and mental state, and just like training the body you have to practise many times and keep up the training to get something out of it. In addition, just as with physical exercise, sometimes it goes very well, and other times you can have difficulty feeling that it is having any effect. The same is true when working with mindfulness.

We will try a brief mindfulness session today that will introduce you to these techniques.
During the patient education course, you have or will try out a longer mindfulness session in the form of a body scan.

Slide 2

Mindfulness

A tool that can train the ability to ‘be’ in life with your chronic illness/pain

I.e. an aid for:

- Being able to accept feelings and pain without judgement or evaluation
- Being with the emotions and pain and living with them
- Reduce the feeling of pain in the long term

Mindfulness needs to be trained, just like physical exercise, to have an effect

End of slide: When you relate to how you feel right now – and not how you should feel or wish you felt – you are being mindful!

This allows you to become consciously present from moment to moment. By observing your thoughts, feelings and pain, rather than reacting to them and starting to act on them, you can let go of the past, the future and the desire for things to be different than they are.

This shift in attitude – welcoming whatever comes instead of fighting or striving to eliminate it – creates a change in how you sense and experience the present moment.

Mindfulness differs from psychotherapy by acting directly on the areas of the brain that regulate our emotions. It inhibits activity in these areas, making us think more objectively and clearly.

Slide 3

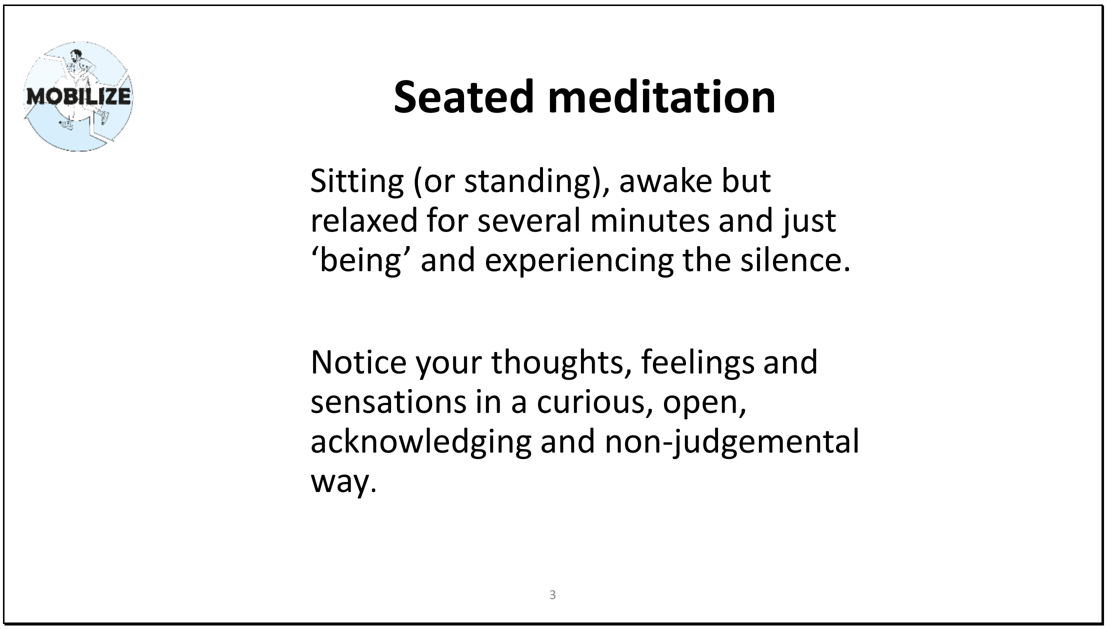

Introduction to slide: One can be mindful in many ways. Now we briefly describe different ways.

End of slide: Seated meditation is a way to practice mindfulness in your everyday life that doesn't require much space or time.

Slide 4

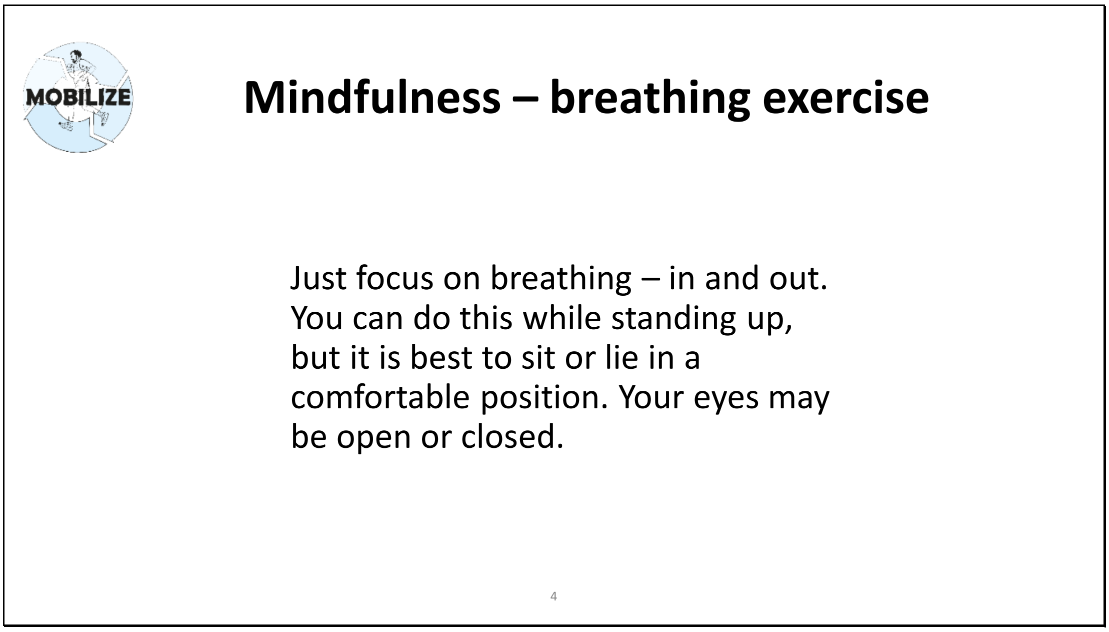

End of slide: The advantage of focusing on the breath when practicing being mindful is that it helps you let go of your thoughts because you have the breath to focus on.

You can do this while standing up, but it is best to sit or lie in a comfortable position.
Your eyes may be open or closed, but it is usually easier to stay focused if you close your eyes...

Slide 5

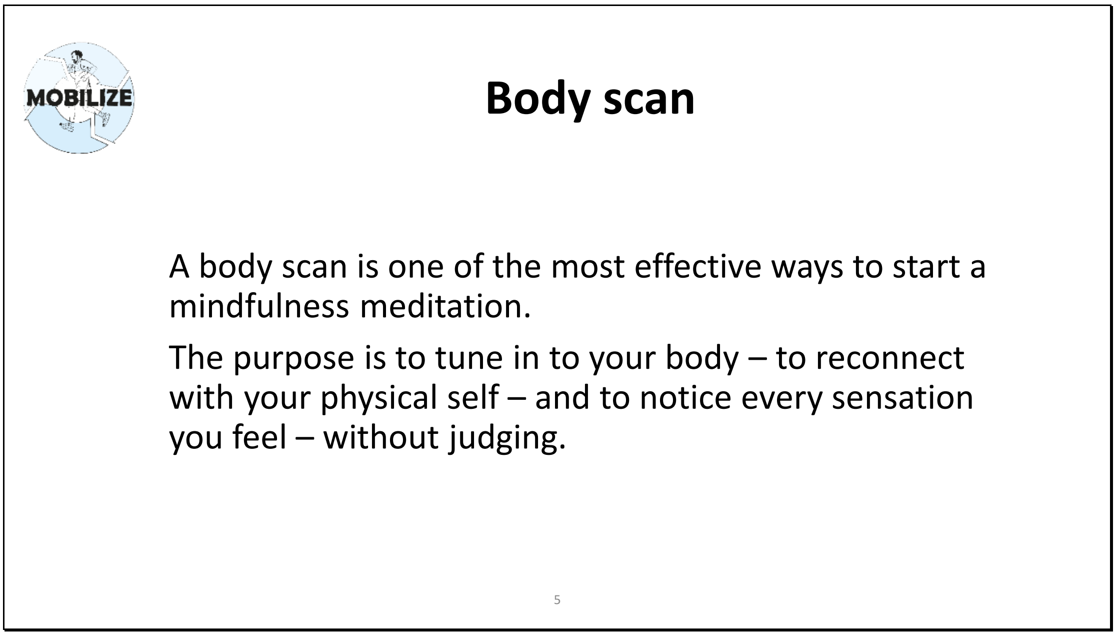

End of slide:
For example, a body scan may mean that you lie down, relax and focus on one body part at a time. For example, you can start by focusing on your feet and noticing whether they are relaxed. Then, move on to the lower legs, knees, thighs, etc.
The advantage is that it is an effective way to practice mindfulness but requires more time and a calm environment.

Slide 6

Brief mindfulness exercise

Ask participants to position themselves so that they are sitting comfortably and are relaxed.
Guide the participants (Duration: 5 minutes):
Begin by taking several long slow deep breaths breathing in fully and exhaling fully.
Breathe in through your nose and out through your nose or mouth. Allow your breath to find its own natural rhythm. As you turn more deeply inward, begin to let go of noises around you. If you are distracted by sounds in the room, simply notice them and then bring your intention back to your breath.
If your mind wanders to thoughts, plans or problems, simply notice your mind wandering. Watch the thought as it enters your awareness as neutrally as possible. Then practice letting go of the thought as if it were a leaf floating down a stream. In your mind, place each thought that arises on a leaf and watch as it floats out of sight down the stream. Then bring your attention back to your breath. Your breath is an anchor you can return to over and over again when you become distracted by thoughts.
As this practice comes to an end, slowly allow your attention to expand and notice your entire body and then beyond your body to the room you are in. When you're ready, open your eyes and come back fully alert and awake.

Afterwards, give the participants the opportunity to briefly comment on the experience. (Duration: 5 minutes).
Ask the participants: Were you able to let go of your thoughts and be in the moment?

Remind the participants that even if they found that it didn't make sense the first time, it needs to be practised a few times, just like with physical exercise. It is also OK if some participants don't think it is for them.

Slide 7

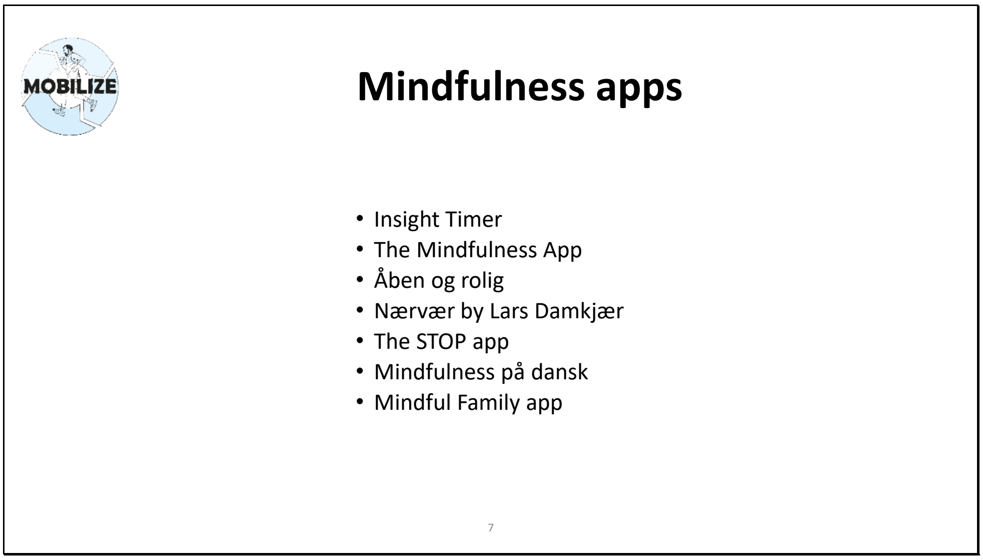

These are suggestions for some of the mindfulness apps that are available which you can try. There are no general recommendations from the health service.

Slide 8

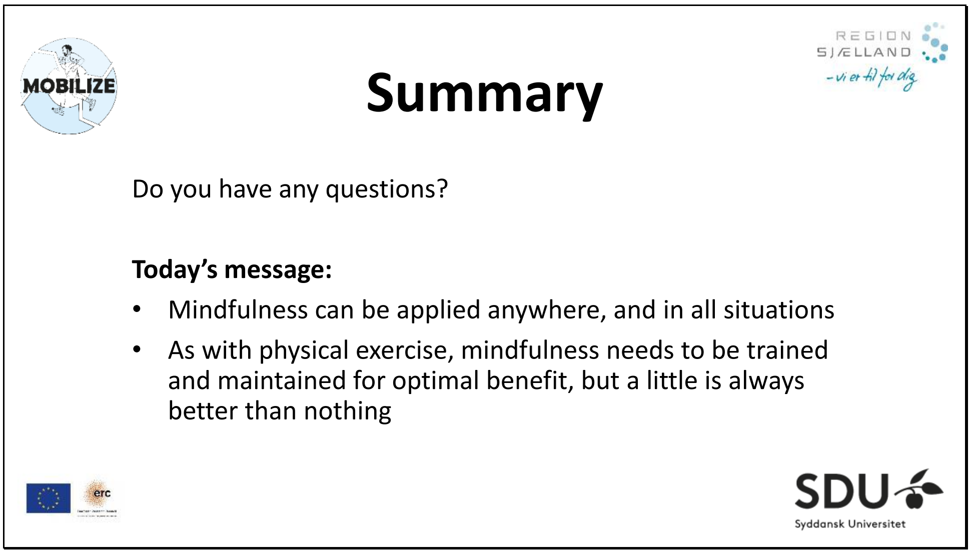

Answer any questions.

Suggestions for follow-up at home:

- Practise mindfulness techniques.
- Find links to videos with mindfulness exercises in your patient handbook.

Finally, remind the participants that they are welcome to bring their carers/relatives to the next module on After MOBILIZE.

After MOBILIZE

Slide 1

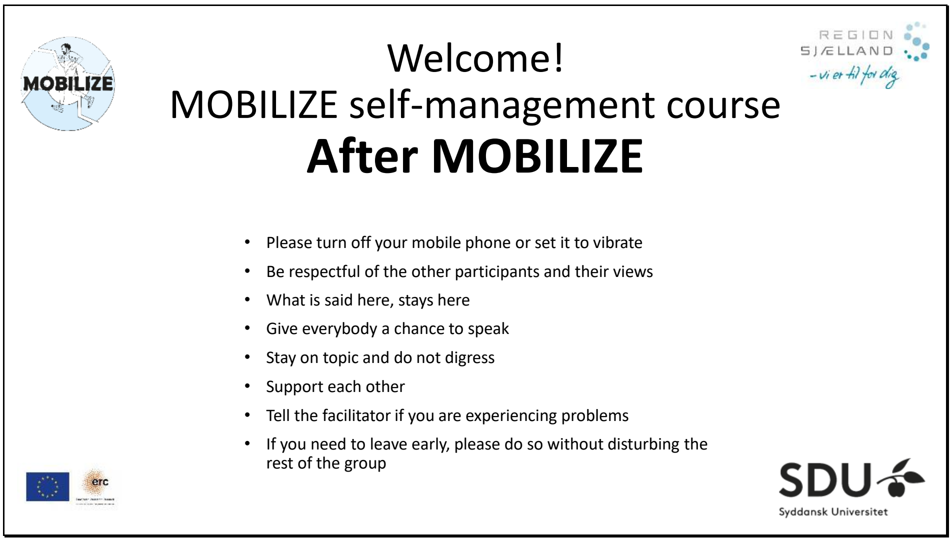

This is one of the modules where MOBILIZE recommends that carers/relatives are invited to participate.

Paper and pen are required as part of this training.

Welcome to the thematic module on how to proceed after MOBILIZE.

We know from research that it can be difficult to maintain new lifestyle habits once the programme ends. In this module, we will help you become aware of what supports and motivates you to move more in everyday life and give you tips on how to maintain a more active life after MOBILIZE.

Slide 2

Keeping going can be difficult

- Unfortunately, when a training project ends, many of the participants also stop being physically active...
- But if you stop being physically active, the positive effects you gain from exercise will quickly disappear...
- And in the long run, it can mean losing your independence

Point 1) It is a challenge for most people to take control of themselves.

Point 2) Research shows that exercise works as a treatment for as many as 26 different illnesses – including the illnesses that got you involved in MOBILIZE.

Point 3) As you get older, it is important to maintain as much muscle strength as possible – if you lose strength, you lose the ability to move around on your own and you become dependent on others for help.

Slide 3

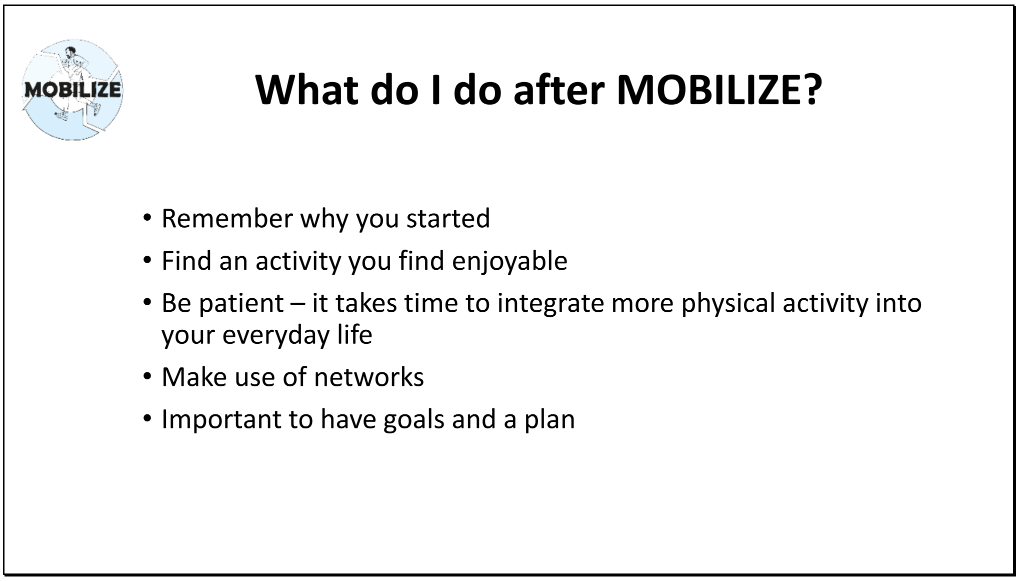

Introduction to slide: What do you do to integrate good habits and more physical activity into your everyday life when MOBILIZE ends? We will look at that now.

Point 1) Involve the participants and ask what got them started. Was it to be able to take the dog on longer walks? Be able to lift and play with your grandchild? Take the stairs up to your flat? Or something else entirely...
It is important to hold on to what made you want to change in the first place.

Point 2) Find the exercise/activity that you find fun or that motivates you. This helps to ensure that you stick with it in the long run. Although interval training on a bike is great, it won't help if you hate sitting on a bike.

Point 3) It takes time to make exercise a habit, an integrated part of your everyday life, so be patient and don't set your ambitions too high at first.

Point 4) Having an agreement to train together with others can be an obligation (and motivating). Notice if you have good chemistry with some of the other participants – perhaps you can form a small group that can continue together after MOBILIZE? Remember to agree on the framework if you meet after MOBILIZE (how often you will meet, who takes the initiative, who to call if you can't make it, etc.) Patient organisations and other associations may also have outdoor and indoor programmes that could suit you.

Point 5) You have already created a goal chart with a specific goal for your participation in MOBILIZE. Let this motivate you. By the time you go home today, you will also have given some thought to what happens after MOBILIZE.

Slide 4

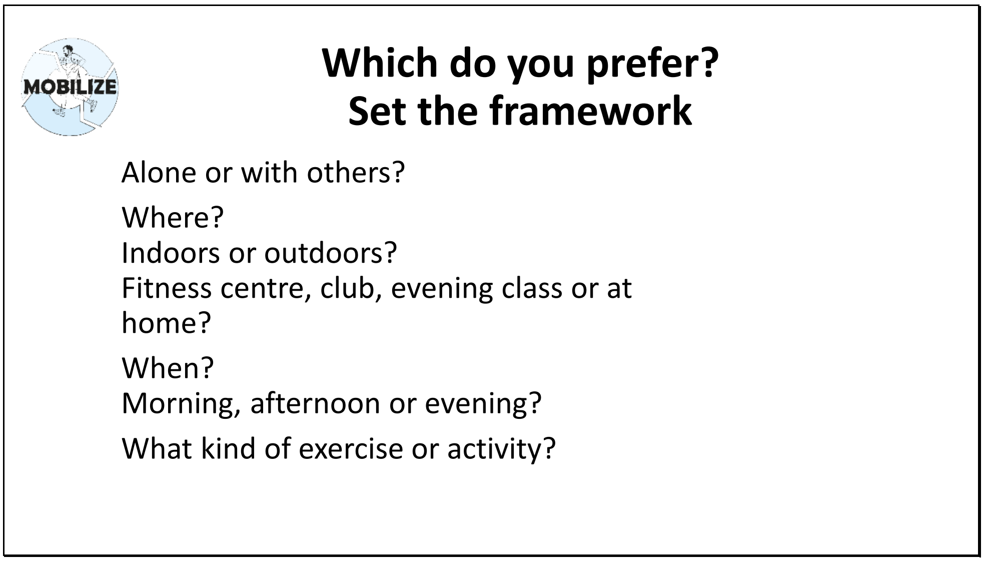

Hand out a piece of paper and pen to participants.

Paragraph1) Click the first point and ask the participants to discuss the pros and cons of movement, alone or with others. Then ask each of them to write down what they prefer.

- Do you prefer to go for a walk alone when it suits you?
- Perhaps you would benefit from a training partner who partly obliges you/partly entertains you as you walk, so you forget that it's hard?
- Maybe you enjoy the social aspect of being part of a team?

Paragraph 2) Click the next item and ask participants to discuss the different options for where training can take place. Then ask each of them to write down what they prefer.

- Are you a nature lover? And do you have a raincoat and a good pair of hiking shoes?
- Or are you more comfortable doing it at home? And do you have the equipment you need (training exercises, stairs, exercise bands, exercise ball, mat)?
- Or would you like to join a fitness centre or a club? And do you have their phone number or website so you can contact them?
- Are you willing to drive, or would you prefer to exercise close to where you live? And what is available near where you live?

Paragraph 3) Click the next item and ask participants to discuss the pros and cons of different times. Then ask them to note which time works best for each of them.

- Are you the type of person who prefers to get your training session done first thing in the morning or do you prefer to exercise later in the day?

- Or are you still in the labour market and dependent on working hours?

Paragraph 4) Click on the next point and ask the participants to think about what kind of activity they find fun and would like to do after MOBILIZE. Then ask them to write this down on paper.

- What motivates you?
- Do you prefer activities that require strength or something that requires endurance? Or maybe it should be something with a ball?

Now ask the participants to look at the four answers they have written down and then have a plenary discussion with suggestions and tips for each participant based on what they would like to do (not what they should do). Perhaps one participant knows of a local organisation or activity that could be interesting for someone else? Perhaps a participant has tried training at home before? Or maybe there are some participants who can come together and form a community after MOBILIZE (since their answers match)?

(Duration: 15–20 minutes)

Slide 5

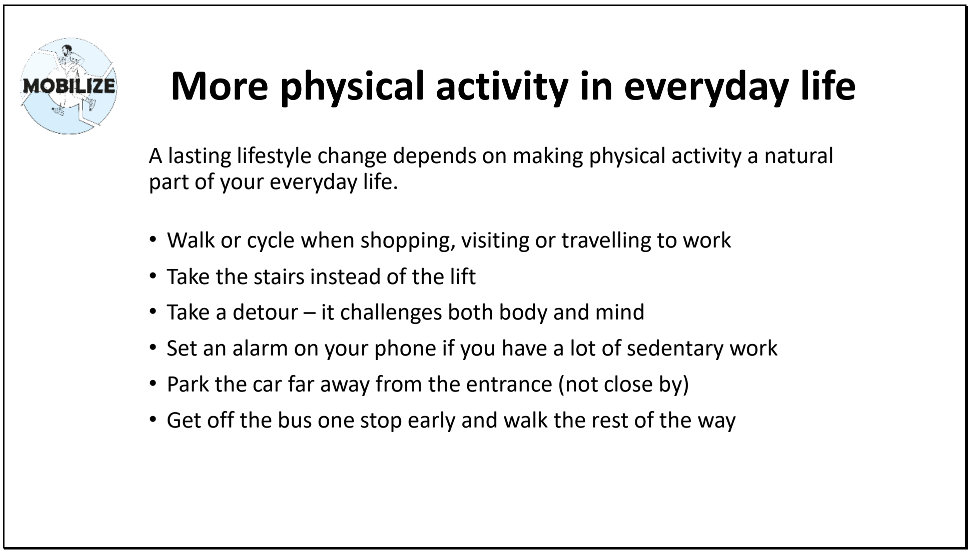

Introduction to slide: Maybe you are not interested in group training or fixed training times at certain locations. However, you can still increase your activity level by incorporating more physical activity into your everyday life and making it a natural part of your daily routine. You already move during the day, and often only small adjustments are needed to optimise activity levels and strengthen the body.

Ask the participants to come up with clear suggestions for how they could each introduce more activity into their everyday life (before clicking further to the points).

Slide 6

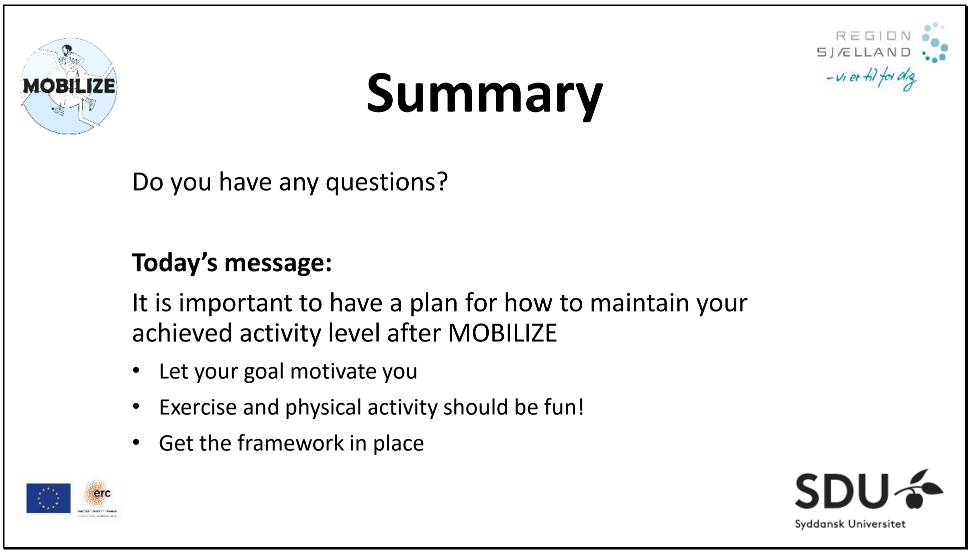

Answer any questions.

Are you now more aware of what each of you can do to stick to your new exercise habits and/or increase your everyday activity levels?
Please keep in mind: it isn't very difficult to 'sneak' more activity into your everyday life.

Supplementary Appendix 9: Patient and public involvement reported according to the GRIPP2 reporting checklist

Section and topic	Item
**1: Aim**<br>Report the aim of the study	To investigate the effectiveness of a personalized exercise therapy and self-management support program alongside usual care, or usual care alone on health-related quality of life (primary outcome) and a range of self-reported, objectively measured and physiological outcomes in people with multimorbidity (i.e. at least two of the following conditions: knee or hip osteoarthritis, chronic obstructive pulmonary disease, heart disease (heart failure or coronary heart disease), hypertension, type 2 diabetes mellitus, depression).<br><br>To collaboratively involve patients as research partners at all stages and in all aspects of the research project to ensure the patient and public involvement (PPI) perspective in the process and outcomes.
**2: Methods**<br>Provide a clear description of the methods used for PPI in the study	Initially, two patients and one family member were recruited to the research team, and we co-created an exercise therapy and self-management intervention using integrated knowledge translation as defined by the Canadian Institutes of Health Research. We strived to involve the patient partners on the collaborate level of the IAP2 spectrum of public participation, and they participated in monthly research meetings and on an ad hoc basis (online, written, in person) and helped to:<br><br>• Discuss the outcomes of the project<br>• Identify topics for and format of the self-management program<br>• Assist in the development of interview guides for focus group interviews<br>• Pilot the exercise therapy and self-management program<br>• Co-develop, feature in and check the comprehension of information communicated from the project (e.g. recruitment flyers, videos, the MOBILIZE website)<br>• Clarify materials for dissemination to patients (e.g. patient information sheets and questionnaires)<br>• Co-develop a patient handbook<br>• Contribute to dissemination strategies<br>During the randomized controlled trial (RCT), four more patients and two more family members were recruited to the research team. They helped to:<br><br>• Facilitate the recruitment process through snowballing techniques and participation at MOBILIZE events for patients

	• Explain their role as patient partners at conferences and events<br>During/after the RCT, two patient partners stopped due to progression of disease and two family members due to time issues, but the rest of the patient partners and family members assisted in the public dissemination and implementation of the results. They<br><br>• Facilitated dissemination of study findings to past research participants, stakeholders and the general public<br>• Will help draft and produce online support tools based on the exercise therapy and self-management program<br>• Participated in conferences and other dissemination activities. together with the research team<br>• Will helped organize and host a workshop informing stakeholders of the application of the results
**3: Results**<br>Outcomes—Report the results of PPI in the study, including both positive and negative outcomes	PPI contributed to the study in several ways, including:<br><br>• It increased the reach and impact of our research by actively ensuring the lay perspective in information and results communicated from the project<br>• Patient priorities and perspectives were designed into the topics of the self-management program<br>• After the feasibility testing of the exercise therapy and self-management program, patient partners helped adjust the program before proceeding with the RCT<br>• A patient handbook was co-developed<br>• The patient partners facilitated the recruitment process. Among other things, they participated in three MOBILIZE events for patients, where at least 15 patients were recruited for the RCT<br>• Patient partners helped sharing patient stories. One of our patient partners published a Patient Voice article about his life with multiple chronic conditions which was published in a peer-reviewed journal (British Journal of Sports Medicine)<br>• Patient partners will facilitate the implementation in clinical practice. Among other things, they will participate at workshops and meetings with municipalities
**4: Discussion**<br>Outcomes—Comment on the extent to which PPI influenced the study overall. Describe	PPI had high priority in this study and influenced important aspects of it, based on the impacts mentioned in section 3. This might have been related to several factors. Firstly, the patient partners had received thorough introduction to the study and participated at the monthly project meetings throughout the first 15 months of the project. Also, they participated in many different aspects of the project from co-production of videos to recruitment events at hospitals and therefore worked closely with the entire project team. Finally, the project ran for more than five years, allowing the project team to establish close relations with the patient partners and mitigating power imbalances.

positive and negative effects	However, there were limitations. The patient partners were not involved from the very beginning of the project, and so they did not contribute to the formulation of the research question or applications for funding. However, they were involved immediately after funding was secured allowing them to help shape the program, recruit patients and ensure that patient priorities and perspectives were reflected throughout the project. In similar future studies, involving patient partners from the very beginning would potentially broaden the pool of ideas about what is important to be researched.<br><br>At the beginning, the project team members were not experienced at involving patient partners in their research and therefore we could have done more to provide clarity regarding roles and expectations. However, we learned as we went along, and a supportive attitude of patient involvement from everybody assisted in the positive impact that PPI had on this study. In similar future studies, to streamline the process, we would use tools such as an 'involvement matrix'[1] that uses a participatory research methodology in which meaningful conversations between patients and researchers are made possible.
**5: Reflections** Critical perspective—Comment critically on the study, reflecting on the things that went well and those that did	If this was repeated, the patient partners should be involved at an even earlier stage to be able to influence the start-up and the design phase.<br><br>Our group of patient partners were volunteers of similar socioeconomic and cultural status and so they did not represent traditionally marginalized voices. Next time, we will do more to broaden the diversity of our volunteer group.

[1] de Wit M, Beurskens A, Piškur B, Stoffers E, Moser A. Preparing researchers for patient and public involvement in scientific research: Development of a hands-on learning approach through action research. Health Expect. 2018 Aug;21(4):752-763. doi: 10.1111/hex.12671. Epub 2018 Feb 8. PMID: 29418053; PMCID: PMC6117481.

not, so others can learn from this experience	
