# Supplementary material for: Exercise therapy and self-management support for individuals with multimorbidity: a randomized and controlled trial
Source: Nat Med. 2025 Jun 30;31(9):3176–82. doi: 10.1038/s41591-025-03779-4 (PMC12443619; doi:10.1038/s41591-025-03779-4)
Supplement: Supplementary file 2 — Reporting Summary [file 41591_2025_3779_MOESM2_ESM.pdf]

nature portfolio

Corresponding author(s): Søren T. Skou

Last updated by author(s): Mar 10, 2025

Reporting Summary

Nature Portfolio wishes to improve the reproducibility of the work that we publish. This form provides structure for consistency and transparency in reporting. For further information on Nature Portfolio policies, see our Editorial Policies and the Editorial Policy Checklist.

Statistics

For all statistical analyses, confirm that the following items are present in the figure legend, table legend, main text, or Methods section.

n/a	Confirmed	
☐	☒	The exact sample size (*n*) for each experimental group/condition, given as a discrete number and unit of measurement
☐	☒	A statement on whether measurements were taken from distinct samples or whether the same sample was measured repeatedly
☐	☒	The statistical test(s) used AND whether they are one- or two-sided<br>*Only common tests should be described solely by name; describe more complex techniques in the Methods section.*
☐	☒	A description of all covariates tested
☐	☒	A description of any assumptions or corrections, such as tests of normality and adjustment for multiple comparisons
☐	☒	A full description of the statistical parameters including central tendency (e.g. means) or other basic estimates (e.g. regression coefficient) AND variation (e.g. standard deviation) or associated estimates of uncertainty (e.g. confidence intervals)
☐	☒	For null hypothesis testing, the test statistic (e.g. *F*, *t*, *r*) with confidence intervals, effect sizes, degrees of freedom and *P* value noted<br>*Give P values as exact values whenever suitable.*
☒	☐	For Bayesian analysis, information on the choice of priors and Markov chain Monte Carlo settings
☐	☒	For hierarchical and complex designs, identification of the appropriate level for tests and full reporting of outcomes
☒	☐	Estimates of effect sizes (e.g. Cohen's *d*, Pearson's *r*), indicating how they were calculated

Our web collection on statistics for biologists contains articles on many of the points above.

Software and code

Policy information about availability of computer code

Data collection	EasyTrial v. 1.0.1.1 (EasyTrial ApS, Aalborg, Denmark)
Data analysis	SAS v. 9.4 (SAS Institute Inc., Cary, NC, USA)

For manuscripts utilizing custom algorithms or software that are central to the research but not yet described in published literature, software must be made available to editors and reviewers. We strongly encourage code deposition in a community repository (e.g. GitHub). See the Nature Portfolio guidelines for submitting code & software for further information.

Data

Policy information about availability of data

All manuscripts must include a data availability statement. This statement should provide the following information, where applicable:

- Accession codes, unique identifiers, or web links for publicly available datasets
- A description of any restrictions on data availability
- For clinical datasets or third party data, please ensure that the statement adheres to our policy

Deidentified data and data dictionaries from the MOBILIZE study are available from the principal investigator (Prof. Søren T. Skou, stskou@health.sdu.dk) after publication of the primary publications and until 5 years after the publication of this manuscript. However, restrictions apply to the availability of the deidentified data due to GDPR and study-specific regulation and access requires a data sharing agreement and a research proposal that will be evaluated by the study group. Requests to access data can expect to be answered within 3 months.

Human research participants

Policy information about studies involving human research participants and Sex and Gender in Research.

Reporting on sex and gender	All patients adhering to the eligibility criteria, regardless of sex and gender, were included. We report on the prevalence (n(%)) of male and female sex (determined by the civil registration number in Denmark) in Table 1, but did not plan or conduct a formal analyses related to sex or gender.
Population characteristics	See Table 1. Patients (n=227) had a mean age of 69.8 years (SD 8.4), mean BMI of 30.9 (SD 5.7), (n=98) 43% were female, and patients had on average 7 chronic conditions (SD 3, range 2-19). One of the original 228 patients withdrew written consent and permission to use data.
Recruitment	Participants were recruited from four general practitioners, two psychiatric facilities, and six hospital departments in the Region of Zealand, Denmark, as well as by self-referral. Recruitment methods included direct consultations, Facebook ads, local newspaper articles, and other forms of advertising such as posters and handouts. Recruitment by self-referral may lead to the inclusion of individuals who might not have sought care for their conditions elsewhere, as well as those with greater motivation to participate in a study, thereby affecting generalizability.<br>Individuals visiting one of the recruitment sites who met the eligibility criteria were invited to participate in the RCT. Patient records were also reviewed to identify eligible participants, who were then contacted by phone. Interested individuals were referred to the MOBILIZE project team, and a team member followed up to finalize their inclusion. For self-referrals, a project team member provided detailed information about the study and assessed their eligibility for enrollment by phone. A MOBILIZE-affiliated medical specialist evaluated self-referrals to ensure they complied with the eligibility criteria on being diagnosed with the listed conditions, not having unstable health conditions or were at risk of serious adverse events.<br>Once the patients verbally agreed to participate, written informed consent was obtained by study personnel before they were enrolled in the study. Participants received reimbursement for transportation to the outcome assessments and study treatment.
Ethics oversight	The study was approved by the Regional Committees on Health Research Ethics for Region Zealand (SJ-857).

Note that full information on the approval of the study protocol must also be provided in the manuscript.

Field-specific reporting

Please select the one below that is the best fit for your research. If you are not sure, read the appropriate sections before making your selection.

☒ Life sciences ☐ Behavioural & social sciences ☐ Ecological, evolutionary & environmental sciences

For a reference copy of the document with all sections, see nature.com/documents/nr-reporting-summary-flat.pdf

Life sciences study design

All studies must disclose on these points even when the disclosure is negative.

Sample size	The RCT was powered to detect a difference of 0.074 points between the two groups in the primary outcome (EQ-5D) from baseline to the 12-month follow-up. A priori, the R-function blockrand was used for sample size calculation. To detect the 0.074 difference in change, 95 participants per group were required according to the sample size calcuation, assuming a common standard deviation of 0.156, with 90% power and an alpha level of 0.05. A total of 228 participants were recruited to account for a potential 20% loss to follow-up.
Data exclusions	No data was excluded.
Replication	The two statisticians double-coded and conducted the primary and secondary endpoint intention-to-treat analyses independently (baseline to 12 months) as defined in the statistical analysis plan. They then compared results and finalized the analyses. No other replication was conduction.
Randomization	Participants who met the eligibility criteria and signed the informed consent form were randomized in a 1:1 allocation ratio following baseline assessment. The statistician had previously prepared a computer-generated randomization schedule using permuted blocks of four or six individuals, stratified by the number of chronic conditions (2 or 3+) and by recruitment centers. Allocation numbers were concealed in opaque sealed envelopes, which were only accessible to a study coordinator after informed consent and baseline assessment were completed.
Blinding	The outcome assessors, the research assistant handling the data, and the statisticians were blinded to the randomization.

Reporting for specific materials, systems and methods

We require information from authors about some types of materials, experimental systems and methods used in many studies. Here, indicate whether each material, system or method listed is relevant to your study. If you are not sure if a list item applies to your research, read the appropriate section before selecting a response.

Materials & experimental systems

n/a	Involved in the study
☒	☐ Antibodies
☒	☐ Eukaryotic cell lines
☒	☐ Palaeontology and archaeology
☒	☐ Animals and other organisms
☐	☒ Clinical data
☒	☐ Dual use research of concern

Methods

n/a	Involved in the study
☒	☐ ChIP-seq
☒	☐ Flow cytometry
☒	☐ MRI-based neuroimaging

Clinical data

Policy information about clinical studies

All manuscripts should comply with the ICMJE guidelines for publication of clinical research and a completed CONSORT checklist must be included with all submissions.

Clinical trial registration

ClinicalTrials.gov (NCT04645732).

Study protocol

https://journals.sagepub.com/doi/10.1177/26335565231154447

Data collection

Self-reported outcomes were collected using electronic or paper-based self-reported questionnaires completed at home (EasyTrial ApS, Aalborg, Denmark) at baseline, 4 months (approximately 16 weeks, immediately after the treatment program), 6 months, and 12 months. If a participant was either unable to access the questionnaire electronically or did not wish to complete it electronically, he or she would receive a paper version by mail along with a prepaid return envelope and completed it at home. Objectively measured outcomes were collected at baseline, 4 months, and 12 months at the intervention sites by blinded assessors who had undergone specific training in the test protocol during a one-day course. From January 18, 2022, through to May 30, 2023, we assessed 663 patients with multimorbidity. Ultimately, 228 patients were randomized (36 %, recruitment rate), 115 to the exercise therapy and self-management support intervention group and 113 to the usual care group (Figure 1).

Outcomes

Self-reported outcomes were collected using electronic or paper-based self-reported questionnaires completed at home (EasyTrial ApS, Aalborg, Denmark) at baseline, 4 months (approximately 16 weeks, immediately after the treatment program), 6 months, and 12 months. If a participant was either unable to access the questionnaire electronically or did not wish to complete it electronically, he or she would receive a paper version by mail along with a prepaid return envelope and completed it at home. Objectively measured outcomes were collected at baseline, 4 months, and 12 months at the intervention sites by blinded assessors who had undergone specific training in the test protocol during a one-day course. The outcomes were selected to reflect the anticipated impact of the intervention and to include most of the recommended core outcomes for multimorbidity trials.38
Primary outcome measure
The primary outcome was the descriptive index of the self-reported, EQ-5D-5L questionnaire (5-level version, ranging from -0.758 to 1, higher is better) at 12 months. The EQ-5D-5L is a reliable and valid measure of health-related quality of life.39The descriptive index includes five dimensions (mobility, self-care, usual activities, pain/discomfort and anxiety/depression) which each has five levels. The participants self-reported their problems for each of the dimension, which was then calculated into an overall index value using the Danish EQ-5D-5L value set.40
Secondary outcome measures
All secondary outcomes were evaluated in all participants.
Functional performance was assessed at baseline and at follow-up at 4 and 12 months using the 6-minute walk test and the 30-second chair-stand test, which are commonly used, valid and reliable measures of functional capacity, lower extremity strength and endurance in older adults.41,42 Steps per day and minutes/day of at least light intensity were measured at the same time points using two Axivity® AX3 accelerometers (Axivity Ltd, Newcastle, UK) worn on the right thigh and the wrist of the non-dominant hand. Participants wore them for seven consecutive days, and valid data required at least 22 hours of wear per day on 3 weekdays and 1 weekend day. The measurement followed a protocol previously found valid and reliable.43,44
Self-reported outcomes included the Bayliss burden of illness measure (on a 1-5 scale for each individual condition, summed to a total score for all conditions, higher is more severe disease burden),31 the Personal Health Questionnaire Depression Scale (PHQ-8, ranging from 0-24 points, higher is more severe depression),45,46 the General Anxiety Disorder-7 (GAD-7, ranging from 0-21, higher is more severe anxiety),45 the Self-Efficacy for Managing Chronic Disease scale (ranging from 1 to 10, higher scores indicating higher self-efficacy),47 the 12-item WHO Disability Assessment Schedule (WHODAS 2.0; ranging from 0 (no disability) to 100 (full disability),48,49 and the EQ-VAS of the EQ-5D-5L questionnaire (ranging from 0-100, higher is better self-rated health).39 Finally, self-reported patient acceptable symptom state for quality of life was assessed (yes/no),50 and in those responding no, treatment failure was assessed (yes/no).51,52 The self-reported outcomes instruments have previously been found valid and reliable. The Bayliss burden of illness measure was translated into Danish for this study.
Furthermore, the number of adverse events (AEs) and serious adverse events (SAEs) were self-reported or identified by reviewing medical records during follow-up. AEs and SAEs were defined as any undesirable experience during follow-up leading to contact with the health-care system. They were categorized according to body system or mortality, and assessed for severity by an adjudication committee (UB and PG) experienced in evaluating AEs (e.g., such as pain, falls and fatigue) and SAEs (e.g., hospitalization, disability or permanent damage) based on definitions of SAEs from the U.S. Food and Drug Administration.53
The outcomes were selected to reflect the anticipated impact of the intervention and to include most of the recommended core outcomes for multimorbidity trials and pre-defined in the clinicaltrials.gov registration.
